# Hyperandrogenemia Induces Trophoblast Ferroptosis and Early Pregnancy Loss in Patients With PCOS via CMA‐Dependent FTH1 Degradation

**DOI:** 10.1002/advs.202506091

**Published:** 2025-12-16

**Authors:** Hanjing Zhou, Weijie Yang, Na Liu, Qing Liu, Jiamin Jin, Chenqiong Zhao, Xiaoying Jin, Miao Gui, Haiyan Zhu, Songying Zhang, Yinli Zhang

**Affiliations:** ^1^ Assisted Reproduction Unit Department of Obstetrics and Gynecology Sir Run Run Shaw Hospital School of Medicine Zhejiang University Hangzhou 310016 China; ^2^ Zhejiang Key Laboratory of Precise Protection and Promotion of Fertility Hangzhou 310016 China; ^3^ Zhejiang Provincial Clinical Research Center for Reproductive Health and Disease Hangzhou 310016 China; ^4^ Liangzhu Laboratory Zhejiang University School of Medicine Hangzhou 311121 China

**Keywords:** androgen, early pregnancy loss, ferroptosis, FTH1, metformin, polycystic ovary syndrome, trophoblast stem cell

## Abstract

Polycystic ovary syndrome (PCOS) patients with hyperandrogenemia exhibit an increased risk of early pregnancy loss; however, the underlying mechanisms remain poorly understood. Ferroptosis, an iron‐dependent form of cell death driven by phospholipid peroxidation, has been implicated in various diseases. This study identifies significant iron homeostasis disorders and ferroptosis in PCOS patients with hyperandrogenemia, which is mediated by androgen‐induced reduction of ferritin heavy chain 1 (FTH1) protein levels in trophoblasts. Specifically, androgens upregulate FTH1 mRNA and protein synthesis by binding to androgen response elements on the FTH1 promoter via the androgen receptor (AR). Simultaneously, elevated androgen levels enhance chaperone‐mediated autophagy (CMA) through upregulating LAMP2A (lysosomal‐associated membrane protein 2), thereby promoting FTH1 protein degradation. When androgen levels are excessive or AR is overactivated, this CMA‐driven degradation exceeds FTH1 protein synthesis, leading to a reduction in FTH1 level. Furthermore, metformin was found to compete with androgens for AR binding, thereby stabilizing FTH1 and protecting trophoblasts from ferroptosis. In PCOS‐model mice, metformin significantly reduced early embryonic absorption. These findings reveal androgen‐induced ferroptosis as a key mechanism in placental dysfunction and highlight a potential application of metformin for treatment of early pregnancy loss associated with PCOS.

## Graphical abstract

In PCOS patients with hyperandrogenemia, decreased ferritin heavy chain 1 (FTH1) causes Fe^2^⁺ overload and ferroptosis in trophoblasts. Androgens induce FTH1 protein degradation via AR‐LAMP2A‐mediated chaperone‐mediated autophagy pathway, leading to placental development disruption and early pregnancy loss. Metformin mitigates androgen‐induced placental ferroptosis and early pregnancy loss in PCOS mice partially through antagonizing the androgen‐AR pathway.

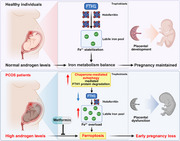

## 1 Introduction

Polycystic ovary syndrome (PCOS) is the most common endocrine disorder among women of reproductive age and is characterized by oligo‐ovulation or anovulation, hyperandrogenemia, insulin resistance, and polycystic ovaries.^[^ ^1^ ^]^ Previous studies have demonstrated that women with PCOS have a greater prevalence of early pregnancy loss (EPL), specifically defined as an empty gestational sac or the termination of embryo development confirmed by ultrasound at 6 to 8 weeks of gestation. Notably, PCOS patients with the hyperandrogenic phenotype had a higher risk of EPL.^[^ ^2^, ^3^ ^]^ Chronic exposure to elevated androgen levels has a well‐documented detrimental effect on endometrial receptivity and embryonic development during early gestation.^[^ ^4^, ^5^, ^6^, ^7^ ^]^ Trophoblasts, critical components of the placenta, play central roles in material exchange and hormone secretion.^[^ ^8^ ^]^ Recent studies have shown that maternal hyperandrogenism causes dysregulation of the proliferation and differentiation of trophoblast precursor cells, consequently impairing trophoblast fusion and invasion in both mouse placentas and human trophoblast organoids.^[^ ^5^, ^7^ ^]^ However, how hyperandrogenemia affects trophoblast development and ultimately induces EPL in patients with PCOS remains unclear.

Ferroptosis is a newly identified type of programmed cell death driven by iron‐dependent phospholipid peroxidation^[^ ^9^ ^]^ in which ferrous ions (Fe^2+^) act as catalysts for the Fenton reaction and promote the accumulation of reactive oxygen species.^[^ ^10^ ^]^ Cellular iron metabolism encompasses the uptake, transport, storage, release, and efflux of iron ions. Ferric ions (Fe^3+^) are taken up by transferrin receptor (TfR1) and converted into Fe^2+^ by the metalloreductase STEAP3, after which they are released into the cytoplasm to form a labile iron pool. Fe^2+^ also originates from the degradation of heme catalyzed by heme oxygenase 1 (HO‐1).^[^ ^11^ ^]^ A portion of the free Fe^2+^ is subsequently exported to the extracellular space, chelates with ferritin for storage, or participates in intracellular biological processes.^[^ ^12^, ^13^ ^]^ The balance of iron metabolism is vital for maintaining cellular iron homeostasis and ferroptosis. Ferritin is composed mainly of two subunits, ferritin heavy chain (FTH1) and ferritin light chain (FTL). FTL participates in the formation of the iron core and the maintenance of ferritin stability. FTH1 has ferrous oxidase activity and promotes Fe^2+^ oxidation and chelation, which plays a central role in maintaining intracellular iron homeostasis. Suppression or knockdown of *FTH1* expression increases the LIP, enhances lipid peroxidation, and promotes ferroptosis,^[^ ^14^, ^15^, ^16^ ^]^ whereas *FTH1* overexpression exerts a protective effect by stabilizing iron metabolism and attenuating oxidative stress.^[^ ^17^, ^18^ ^]^

Previous studies have indicated a potential link between ferroptosis and the occurrence of pregnancy loss.^[^ ^19^ ^]^ Increased placental ferroptosis has been observed in a dihydrotestosterone (DHT)‐induced PCOS‐like rat model with fetal loss, suggesting that ferroptosis may mediate hyperandrogenemia‐induced pregnancy loss.^[^ ^20^ ^]^ Androgens exert their effects mainly through the androgen receptor (AR), a transcription factor that promotes and recruits transcriptional coregulators to remodel chromatin and alter gene expression profiles.^[^ ^21^, ^22^ ^]^ It has been reported that androgens suppress ferroptosis in cancer cells by upregulating their surveillance mechanisms or attenuating their induction mechanisms.^[^ ^23^, ^24^, ^25^, ^26^ ^]^ In contrast, in the ovaries and uterus, androgens promote ferroptosis by suppressing their surveillance mechanisms.^[^ ^27^, ^28^, ^29^ ^]^ Specifically, hyperandrogenism has been shown to cause ferritinophagy and ferroptosis in granulosa cells through the upregulation of nuclear receptor coactivator 4 (NCOA4).^[^ ^30^ ^]^ Nevertheless, the effects of androgens on ferroptosis in trophoblasts remain unclear. Given the evident overload of Fe^2+^ in trophoblasts under hyperandrogenic conditions in our study, investigating how iron metabolism is disrupted is crucial for preventing EPL occurrence in patients with PCOS.

Metformin, a biguanide insulin‐sensitizing drug, is used for glycemic regulation and weight control during pregnancy but does not significantly increase the short‐term risks to the mother or fetus; thus, metformin has promising application prospects.^[^ ^31^, ^32^, ^33^ ^]^ Randomized controlled trials with PCOS patients suggest that the use of metformin in early pregnancy may reduce the risk of EPL and improve pregnancy outcomes, possibly through the amelioration of insulin sensitivity and antiandrogenic effects.^[^ ^34^, ^35^ ^]^ However, whether metformin ameliorates pregnancy loss by antagonizing androgens to protect trophoblasts from ferroptosis remains unknown.

In this study, we provide evidence that excessive androgens activate chaperone‐mediated autophagy (CMA) to induce FTH1 protein degradation, resulting in Fe^2+^ overload and ferroptosis in trophoblasts. We also investigated the role of metformin in antagonizing AR, which protects trophoblasts from androgen‐induced ferroptosis, thus ameliorating placental development and reducing embryo resorption in PCOS mice.

## 2 Results

### 2.1 Androgens Induce Trophoblast Ferroptosis in Patients with PCOS

**Figure 1:**
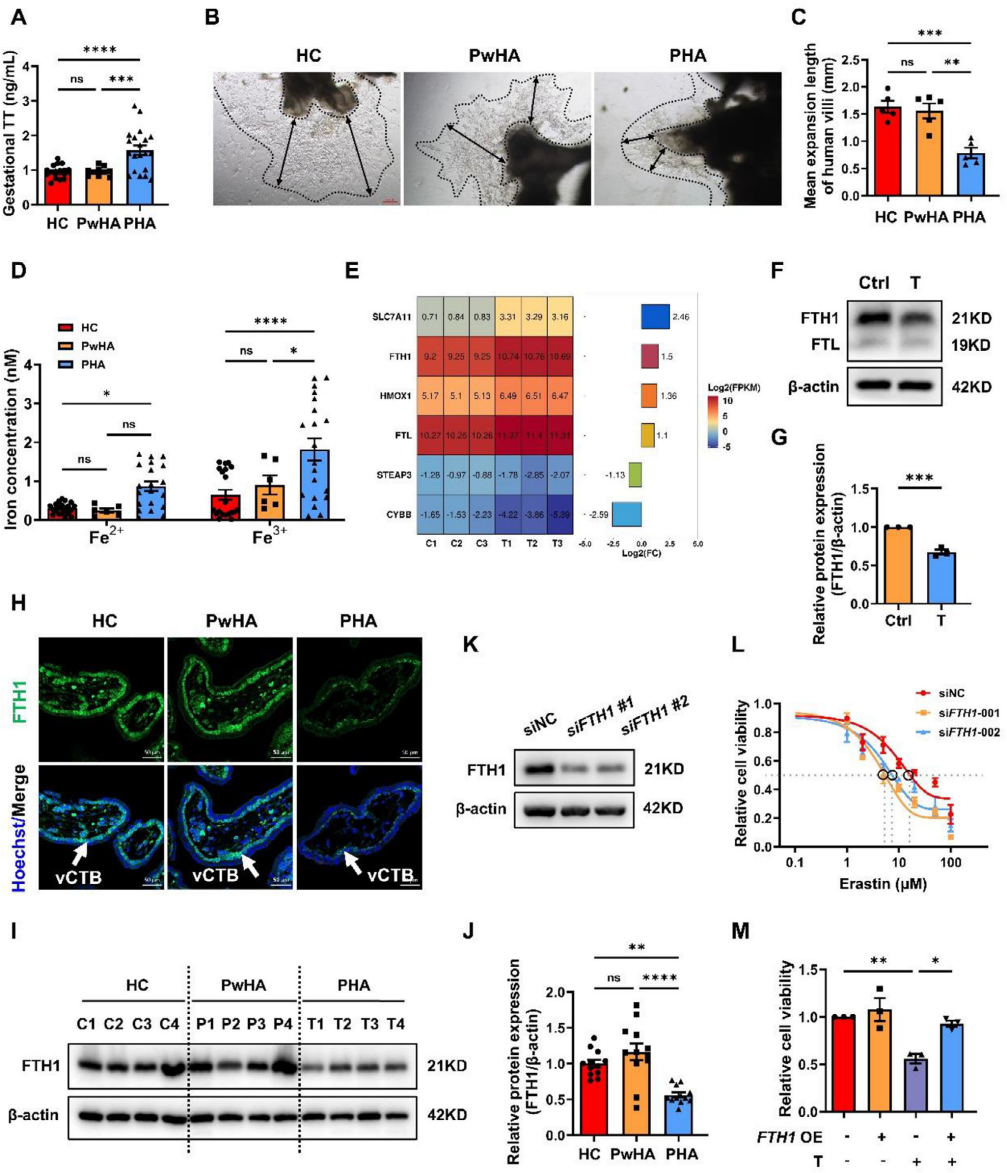
FTH1 protein degradation is involved in androgen‐induced ferroptosis in trophoblasts. A) Early gestational serum TT levels in the HC (n = 20), PwHA (n = 10), and PHA (n = 20) groups. B) Representative images of villus explants from each group cultured for 4 days. The black double‐sided arrows indicated the expansion distance of villus explants. Scale bars, 200 µm. C) The distance that the villus explants in each group grew was quantified. Villus explants for each group derived from five individuals were measured. D) The levels of Fe^2+^ and Fe^3+^ from villi collected between the sixth and eighth weeks in the HC (n = 20), PwHA (n = 6), and PHA (n = 20) groups. E) Heatmap bar plot showing transcriptional divergence (Log_2_ (FPKM)) in six ferroptosis‐related DEGs between the control and T‐treated groups of TS^CT^ cells (*p* < 0.05 and |Log_2_ (FC) | > 1). F) Western blot analysis showing FTH1 and FTL protein levels in TS^CT^ cells treated with T as indicated for 24 h. G) Statistical quantification of FTH1 protein levels in each group normalized to that of β‐actin. The results of three independent experiments were analyzed. H) Representative immunofluorescence images of FTH1 (green) from three independent experiments suggested that FTH1 was expressed mainly on the cytotrophoblast layer (vCTB: white arrow). Hoechst 33 342 indicated the nucleus. Scale bars, 50 µm. I) Representative Western blot analysis of FTH1 expression in villi collected between the sixth and eighth weeks from the HC, PwHA, and PHA groups. n = 4 independent biological samples in each group. J) Statistical quantification of the FTH1 protein level in each villus sample normalized to its β‐actin level. n = 12 independent biological samples were included in each group. K) TS^CT^ cells were transfected with siRNAs (two different sequences, #1 and #2) targeting *FTH1* for 72 h. FTH1 protein levels were analyzed by Western blotting. n = 3 independent experiments were performed. L) Analysis of the viability of TS^CT^ cells transfected with siNC and si*FTH1* (sequence, #1 and #2) for 48 h, followed by treatment with 1, 2, 5, 10, 20, 50, or 100 µM erastin for another 24 h. The results of three independent experiments were analyzed. M) Viability of TS^CT^ cells transfected with FLAG‐FTH1 plasmids for 24 h, followed by T treatment for another 24 h. The results of three independent experiments were analyzed. The data are presented as the mean ± SEM. Statistical analysis was performed using one‐way ANOVA in (A), (C), (D), (J) and (M), unpaired *t*‐test in (G), and a standard curve was interpolated with sigmoidal, 4PL, where X is the concentration model in (L). ns, not significant; *p < 0.05, **p < 0.01, ***p < 0.001, ****p < 0.0001. HC, healthy control; PwHA, patients with PCOS without hyperandrogenemia; PHA, patients with PCOS with hyperandrogenemia; T, testosterone; TT, total testosterone; DEG, differentially expressed gene; FC, fold change; OE, overexpression. T: 20 µM.

To investigate the potential mechanism involved in EPL of patients with PCOS, we collected villi from healthy controls (HC) and patients with PCOS with or without hyperandrogenemia at 6 to 8 weeks of gestation. Elevated circulating testosterone levels were measured in a subset of EPL patients, specifically those with PCOS and hyperandrogenemia, referred to as the PHA group (**Figure** **1**A). The remaining PCOS patients without hyperandrogenemia were classified into the PwHA group. Villus explant culture experiments demonstrated a significantly slower villus expansion rate in the PHA group than in both the HC and PwHA groups (Figure 1B,C). Our results revealed a marked increase in the levels of malondialdehyde (MDA), a final product of lipid peroxidation, in the villi from the PHA group (Figure S1A, Supporting Information), suggesting an imbalance between oxidation and antioxidation processes. Additionally, we observed significant increases in both Fe^2+^ and Fe^3+^ levels in the villi of the PHA group (Figure 1D), indicating iron overload in the placenta of PCOS patients with hyperandrogenemia.

To elucidate the molecular mechanism underlying the androgen‐induced effects in trophoblasts, we used proliferative cytotrophoblast (CTB)‐derived trophoblast stem (TS^CT^) cells as an in vitro model, given that their transcriptome and DNA methylome are similar to those of primary CTBs, and that these cells can differentiate into extravillous cytotrophoblast (EVT)‐like and syncytiotrophoblast (ST)‐like cells upon induction with specific exogenous agents.^[^ ^36^ ^]^ TS^CT^ cells were treated with testosterone at various concentrations and for various durations. The results of the cell proliferation assay revealed dose‐ and time‐dependent cell death upon testosterone treatment (Figure S1B, Supporting Information). In subsequent experiments, TS^CT^ cells were treated with 20 µM testosterone for 24 h. Morphological observations revealed that testosterone exposure caused TS^CT^ cell rounding, shrinkage and plasma membrane rupture, along with mitochondrial swelling, blebbing and intracristal dilatation (Figure S1C, Supporting Information). The levels of MDA were significantly elevated after testosterone stimulation (Figure S1D, Supporting Information). Testosterone treatment also resulted in a decrease in PGSK fluorescence intensity, indicating increased free Fe^2+^ levels (Figure S1E,F, Supporting Information). Cell proliferation assays revealed that pretreatment with the ferroptosis inhibitor ferrostatin‐1 (Fer‐1) or deferiprone (DFP) significantly reversed testosterone‐induced cell death (Figure S1G, Supporting Information). Importantly, testosterone did not significantly increase the percentage of apoptotic cells (Figure S1H,I, Supporting Information). Both the cell proliferation assays and colony formation assays revealed that neither inhibition of apoptosis with Z‐VAD‐FMK nor inhibition of necroptosis with necrostatin‐1 (Nec‐1) reversed testosterone‐induced cell death (Figure S1J–L, Supporting Information), further excluding apoptosis and necrosis as the primary mechanisms of androgen‐induced TS^CT^ cell death. These results collectively suggest that androgens induce ferroptosis in trophoblasts.

### 2.2 Decreased FTH1 Protein Expression Mediates Androgen‐Induced Ferroptosis in Trophoblasts

To further elucidate the underlying molecular mechanism, RNA sequencing was performed to assess the gene expression profiles in TS^CT^ cells treated with or without testosterone. Principal component analysis (PCA) demonstrated clear differences between the control and testosterone‐treated groups (Figure S2A and Table S1, Supporting Information). Kyoto Encyclopedia of Genes and Genomes (KEGG) pathway analysis revealed enrichment of ferroptosis in the testosterone‐treated group (Figure S2B and Table S2, Supporting Information). Among the differentially expressed genes (DEGs), we identified six ferroptosis‐related genes (*SLC7A11*, *CYBB*, *HMOX1*, *STEAP3*, *FTL*, and *FTH1*) and constructed a heatmap showing their changes in expression (Figure 1E). The expression of iron metabolism‐related genes, including *FTH1*, *FTL*, *HMOX1*, and *STEAP3*, was confirmed by qPCR, and that of their encoded proteins was confirmed by Westen blotting (Figure S2C–E, Supporting Information). The expression of both *HMOX1* mRNA and its encoded protein (HO‐1) were upregulated, whereas the STEAP3 protein level did not change despite its decreased mRNA expression. Interestingly, the protein levels of both FTH1 and FTL were notably downregulated, in contrast to their mRNA expression patterns following testosterone treatment (Figure 1F,G). The increase in HO‐1 expression and the decrease in FTH1 and FTL expression collectively led to an increase in intracellular Fe^2+^ levels; however, the baseline level of *HMOX1* mRNA was much lower than those of FTH1 and FTL in TS^CT^ cells (Figure 1E and Table S1, Supporting Information). FTH1 was specifically expressed in the cytotrophoblast layer of the villi (Figure 1H), and its expression was significantly decreased in the PHA group (Figure 1I,J), suggesting that FTH1 may play a vital role in hyperandrogenemia‐induced villus ferroptosis. Notably, knockdown of *FTH1* expression significantly increased the susceptibility of TS^CT^ cells to ferroptosis (Figure 1K,L). Conversely, the overexpression of *FTH1* substantially attenuated intracellular levels of free Fe^2+^ (Figure S2F, Supporting Information), rescued cell viability (Figure 1M), and enhanced trophoblast differentiation, as indicated by a marked increase in hCG protein levels (Figure S2G, Supporting Information). These results support the idea that FTH1 plays a vital protective role against ferroptosis induced by androgens.

### 2.3 Androgens Disrupt the Balance between FTH1 Synthesis And Degradation via AR

**Figure 2:**
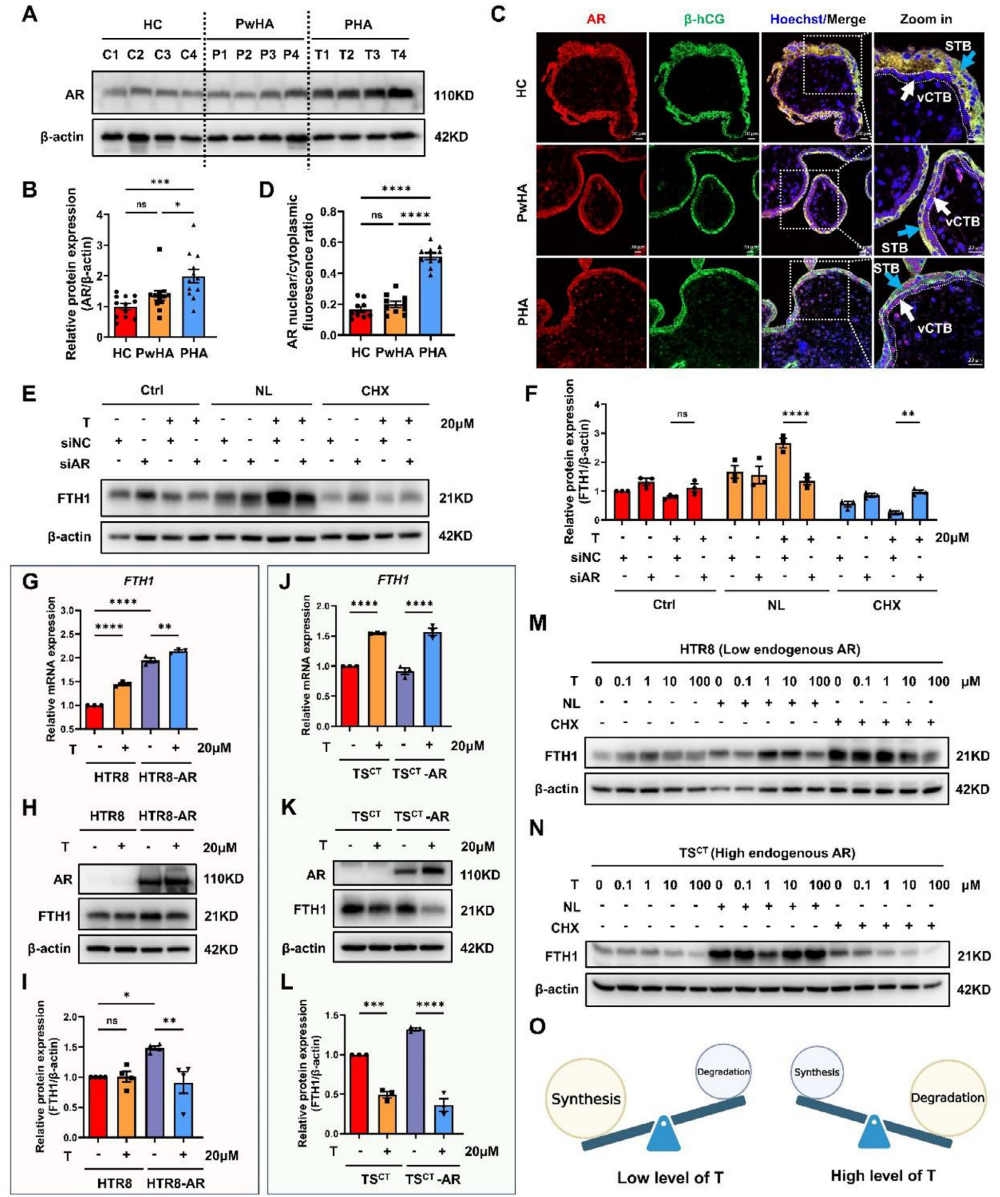
Overactivation of the androgen‐AR axis disrupts the balance between FTH1 synthesis and degradation. A) Representative Western blot analysis of AR expression in 6–8‐week‐old villi from the HC, PwHA, and PHA groups. n = 4 independent biological samples in each group. B) Statistical quantification of the AR protein level for each villus sample normalized to its β‐actin level. n = 12 independent biological samples were included in each group. C) Representative immunofluorescence images of AR (red) and β‐hCG (green) from three independent experiments suggested the localization of AR on the cytotrophoblast layer (vCTB: white arrow) and syncytiotrophoblast layer (STB: blue arrow). Hoechst 33 342 indicated the nucleus. Scale bars, 20 µm. D) Statistical quantification of the nuclear/cytoplasmic AR fluorescence ratio of CTBs was performed. Ten independent fields from five individuals were included in each group. E) Western blot analysis showing FTH1 expression in TS^CT^ cells transfected with siNC or si*AR* for 48 h, followed by T and NL or CHX treatment for another 24 h. F) Statistical quantification of the FTH1 protein levels in each group normalized to those of β‐actin. The results of three independent experiments were analyzed. G) RT‒qPCR analysis of *FTH1* in control and *AR*‐overexpressing HTR8 cells treated with or without T for 24 h. n = 3 biological replicates were included in each group. H) Western blot analysis showing AR and FTH1 expression in control and *AR*‐overexpressing HTR8 cells treated with or without T for 24 h. I) Statistical quantification of the FTH1 protein levels in each group normalized to those of β‐actin. The results of three independent experiments were analyzed. J) RT‒qPCR analysis of *FTH1* in control and *AR*‐overexpressing TS^CT^ cells treated with or without T for 24 h. n = 3 biological replicates were included in each group. K) Western blot analysis showing AR and FTH1 expression in control and *AR*‐overexpressing TS^CT^ cells treated with or without T for 24 h. L) Statistical quantification of the FTH1 protein levels in each group normalized to those of β‐actin. The results of three independent experiments were analyzed. M,N) Western blot analysis showing FTH1 expression in HTR8 (M) or TS^CT^ cells (N) pretreated with NL or CHX for 6 h, followed by treatment with 0.1, 1, 10, or 100 µM T for another 18 h. n = 3 independent experiments were performed. O) Schematic diagram of FTH1 protein synthesis and degradation under low‐ or high‐androgen conditions. The data are presented as the mean ± SEM. Statistical analysis was performed using one‐way ANOVA in (B), (D), (F), (G), (I), (J) and (L). not significant; *p < 0.05, **p < 0.01, ****p < 0.0001. AR, androgen receptor; β‐hCG, human chorionic gonadotropin beta; T, testosterone; CHX, cycloheximide; NL, NH_4_Cl + leupeptin; siNC, negative control siRNA; OE, overexpression. T: 20 µM; CHX: 50 µg mL^−1^; NH_4_Cl: 10 mM; Leupeptin: 20 µM.

Given that androgens exert biological effects by binding to AR,^[^ ^37^, ^38^ ^]^ we further examined the expression of AR in trophoblasts. AR protein levels were significantly higher in the PHA group than in the HC and PwHA groups (**Figure** **2**A,B). Moreover, AR localization to the nucleus was notably increased in both the cytotrophoblast layer and the syncytial layer of villi from PHA patients (Figure 2C,D). In TS^CT^ cells, knockdown of *AR* reversed the testosterone‐induced decrease in FTH1 protein expression after protein synthesis was inhibited by cycloheximide (CHX) (Figure 2E,F), suggesting that testosterone promoted the protein degradation of FTH1 via AR. In contrast, inhibition of lysosome‐mediated protein degradation using NH4Cl + leupeptin (NL) led to the accumulation of the FTH1 protein, whereas the knockdown of *AR* expression reduced the FTH1 protein level in the presence of testosterone (Figure 2E,F), suggesting that testosterone promoted the synthesis and degradation of FTH1 via AR simultaneously. To further assess the role of AR, we analyzed a panel of trophoblast cell lines along with HEK293T cells as negative controls. AR was highly expressed in TS^CT^ and JEG3 cells but was expressed at low levels in HTR8 and HEK293T cells (Figure S3A, Supporting Information). In HTR8 cells (with low endogenous AR expression), *AR* overexpression alone increased the mRNA and protein levels of FTH1 (Figure 2G–I). However, testosterone stimulation further increased *FTH1* mRNA levels in *AR*‐overexpressing HTR8 cells but significantly reduced FTH1 protein levels (Figure 2G–I). These findings indicate that testosterone‐induced AR activation promotes *FTH1* mRNA synthesis and FTH1 protein degradation. In TS^CT^ cells (with high endogenous AR expression), FTH1 mRNA levels increased but FTH1 protein levels decreased upon testosterone exposure regardless of *AR* overexpression (Figure 2J–L). These results indicate that in the presence of high AR levels, testosterone has a stronger promotion effect on the degradation of FTH1 than on its synthesis.

We next determined the bidirectional effects of different concentrations of androgen on FTH1 synthesis and degradation using two kinds of trophoblasts: HTR8 (with low endogenous AR levels) and TS^CT^ (with high endogenous AR levels). At low androgen concentrations (less than 1 µM), FTH1 synthesis outweighed degradation in HTR8 cells (Figure 2M). In contrast, at higher androgen concentrations (more than 1 µM), degradation exceeded synthesis in both HTR8 and TS^CT^ cells, regardless of the baseline AR level (Figure 2M,N). Together, these results indicate that the balance of FTH1 synthesis and degradation is dependent on androgen levels. Specifically, FTH1 synthesis is predominant at low androgen levels, whereas FTH1 degradation is predominant under high androgen conditions (Figure 2O).

### 2.4 AR Directly Binds to the FTH1 Promoter to Promote its Transcription

Enzalutamide is an FDA‐approved AR antagonist that binds to the ligand‐binding domain of AR, preventing AR nuclear translocation and thus suppressing its transcriptional activity.^[^ ^39^ ^]^ Interestingly, enzalutamide not only reduced the baseline expression of FTH1 but also abolished the AR‐mediated upregulation of FTH1 at both the mRNA and protein levels (Figure S3B–D, Supporting Information), suggesting that androgens promote FTH1 synthesis via AR. To investigate the underlying mechanism, we analyzed the *FTH1* gene promoter using the JASPAR database and identified two high‐confidence putative androgen response elements (AREs) (Figure S3E, Supporting Information). CUT&Tag assays and RT‒qPCR of HTR8 cells confirmed that AR directly binds to these AREs of the *FTH1* gene (Figure S3F, Supporting Information). Moreover, the binding of AR to these AREs was significantly enhanced by testosterone and inhibited by enzalutamide (Figure S3G, Supporting Information), suggesting that androgen‐stimulated AR directly transactivates *FTH1* by binding to its promoter.

### 2.5 FTH1 is Preferentially Degraded via the Chaperone‐Mediated Autophagy Pathway in the Presence of Androgens

**Figure 3:**
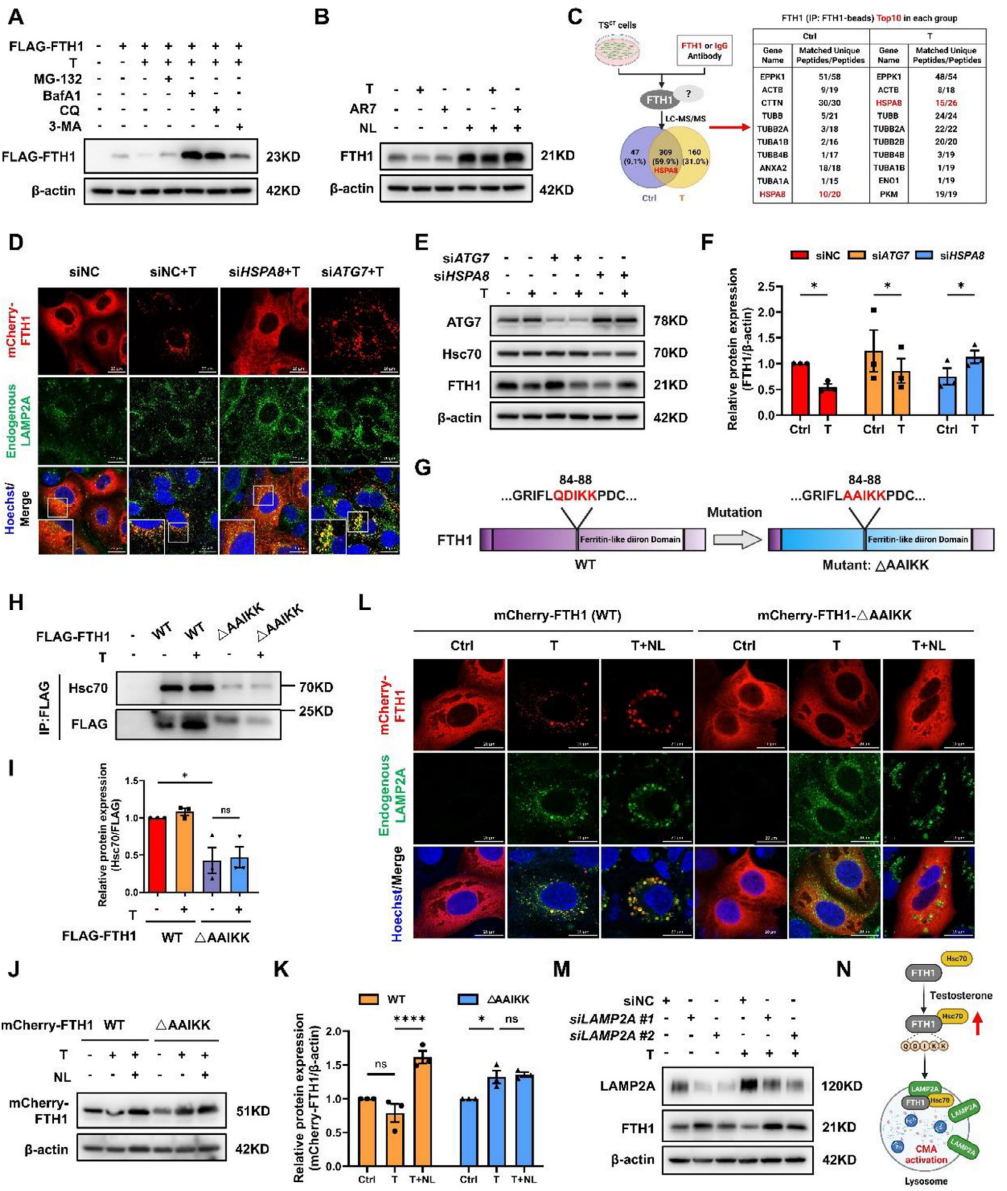
FTH1 is preferentially degraded via the CMA pathway in the presence of testosterone. A) FLAG‐FTH1 protein levels were detected with an anti‐FLAG antibody in TS^CT^ cells pretreated with a proteasome inhibitor (MG‐132), a macroautophagy inhibitor (3‐MA), or a lysosome inhibitor (BafA1 or CQ) for 12 h, followed by T treatment for another 12 h. n = 4 independent experiments were performed. B) Western blot showing endogenous FTH1 expression in TS^CT^ cells treated with T or AR7 for 12 h, followed by NL (lysosomal inhibitors) treatment for another 12 h. n = 3 independent experiments were performed. C) A mass spectrometer was used to detect the proteins that interact with endogenous FTH1 in TS^CT^ cells from the control and T‐treated groups. D) Representative immunofluorescence images showing the colocalization of endogenous LAMP2A (green) and mCherry‐FTH1 (red) in TS^CT^ cells transfected with si*Hsc70* or si*ATG7* for 48 h, followed by T treatment for another 24 h. Hoechst 33 342 (blue) indicated the nucleus. Scale bars, 20 µm. E) Western blot analysis showing ATG7, Hsc70, and FTH1 protein levels in TS^CT^ cells transfected with si*ATG7* or si*HSPA8* for 48 h, followed by T treatment for another 24 h. F) Statistical quantification of the FTH1 protein levels in each group normalized to those of β‐actin. The results of three independent experiments were analyzed. G) Schematic representation of the KFERQ‐like motif on the human FTH1 amino acid sequence from the 84^th^ to 88^th^ amino acids and the mutation of the QDIKK motif. H) TS^CT^ cells were transfected with wild‐type (WT) FLAG‐FTH1 or FLAG‐FTH1 containing mutant ferritin‐like diiron domain (AAIKK) plasmids for 48 h, followed by treatment with or without T for another 24 h. The binding of exogenous FTH1 and endogenous Hsc70 was confirmed by co‐IP. I) Statistical quantification of Hsc70 protein levels in the WT FLAG‐FTH1 and mutated FLAG‐FTH1 groups treated with or without T and normalized to those in the FLAG‐FTH1 group. The results of three independent experiments were analyzed. J) WT mCherry‐FTH1 or mutated mCherry‐FTH1 plasmids were transfected into TS^CT^ cells, followed by T and/or NL treatment for 24 h. mCherry‐FTH1 protein levels were detected with an anti‐mCherry antibody using Western blotting. K) Statistical quantification of mCherry‐FTH1 protein levels in each group normalized to that of β‐actin. The results of three independent experiments were analyzed. L) Immunofluorescence colocalization of endogenous LAMP2A (green) and mCherry‐FTH1 (red) in WT or mutated‐*FTH1*‐overexpressing TS^CT^ cells, with or without T and/or NL treatment for 24 h. Hoechst 33 342 (blue) indicated the nucleus. Scale bars, 20 µm. M) TS^CT^ cells were transfected with siRNAs (two different sequences, #1 and #2) targeting *LAMP2A* for 48 h and then treated with T for another 24 h. LAMP2A and FTH1 protein levels were analyzed by Western blotting. n = 3 independent experiments were performed. N) Schematic illustration showing that CMA is involved in the degradation of the FTH1 protein under androgen stimulation. The data are presented as the mean ± SEM. Statistical analysis was performed using one‐way ANOVA in (F), (I) and (K). ns, not significant; *p < 0.05, ****p < 0.0001. IP, immunoprecipitated; T, testosterone; BafA1, bafilomycin; CQ, chloroquine; 3‐MA, 3‐methyladenine; NL, NH_4_Cl + Leupeptin. T: 20 µM; MG‐132: 10 µM; BafA1: 100 nM; CQ: 5 µM; 3‐MA: 10 mM; AR7: 10 µM; NH_4_Cl: 10 mM; Leupeptin: 20 µM.

Protein degradation predominantly occurs via two pathways: a proteasome‐dependent pathway and a lysosome‐dependent pathway. Next, we investigated the mechanism underlying testosterone‐mediated FTH1 degradation. TS^CT^ cells were treated with testosterone in combination with inhibitors targeting different degradation pathways: the proteasome inhibitor MG‐132, the lysosomal inhibitor chloroquine (CQ), the autophagosome‒lysosome fusion inhibitor bafilomycin A1 (BafA1), or the autophagosome formation inhibitor 3‐methyladenine (3‐MA). Unlike treatment with MG‐132 or 3‐MA, treatment with either CQ or BafA1 markedly suppressed FTH1 degradation (**Figure** **3**A). This inhibition was accompanied by increased accumulation of FTH1 within lysosomes marked by LAMP1 (Figure S4A, Supporting Information). Notably, transmission electron microscopy analysis revealed an increased number of lysosomes after testosterone treatment, with BafA1 used as a positive control (Figure S4B,C, Supporting Information). These results indicate that testosterone facilitates FTH1 degradation via a lysosome‐dependent mechanism.

To assess whether macroautophagy is involved in the observed lysosomal degradation, we inhibited FTH1 synthesis with CHX and silenced *ATG5* and *ATG7* to block autophagosome formation. Notably, suppression of macroautophagy failed to prevent FTH1 degradation upon testosterone treatment (Figure S4D,E, Supporting Information), indicating that FTH1 lysosomal degradation is independent of canonical macroautophagy. We therefore focused on CMA, a selective lysosomal process inducible by stresses such as starvation, oxidation or genotoxicity.^[^ ^40^ ^]^ Chemical stimulation of CMA by the atypical RARA/RARα receptor (retinoic acid receptor alpha) antagonist AR7 markedly reduced FTH1 levels in the absence of testosterone induction (Figure 3B). This reaction was reversed by cotreatment with NL, indicating that CMA is involved in FTH1 degradation.

CMA substrates are recognized and delivered to lysosomes by the heat shock cognate protein Hsc70 (encoded by *HSPA8*) via a KFERQ‐like motif.^[^ ^41^ ^]^ Coimmunoprecipitation with anti‐FTH1 antibodies followed by mass spectrometry analysis confirmed that FTH1 interacts with Hsc70 in both control and testosterone‐treated TS^CT^ cells (Figure 3C and Table S3, Supporting Information). Moreover, testosterone enhanced the interaction between FTH1 and Hsc70 (Figure S5A,B, Supporting Information). Consistent with these findings, knockdown of *HSPA8*, but not *ATG7*, attenuated the lysosomal accumulation of FTH1 (Figure 3D) and rescued FTH1 protein levels under testosterone stimulation (Figure 3E,F), underscoring the specificity of the CMA pathway.

Sequence analysis revealed a conserved KFERQ‐like motif (QDIKK) in FTH1. To verify its role, we generated a mutant form of FTH1 by mutation of QDIKK to AAIKK (referred as ΔAAIKK) (Figure 3G). Importantly, mutation of the motif significantly weakened the FTH1‐Hsc70 interaction (Figure 3H,I), abolished testosterone‐induced degradation of the FTH1 protein, and rendered it unresponsive to lysosomal inhibition (Figure 3J,K). Immunofluorescence further demonstrated that upon testosterone treatment, wild‐type mCherry‐FTH1, but not the KFERQ‐like motif mutant, formed punctate structures that colocalized with lysosomal markers (Figure 3L). Moreover, lysosomal inhibition by NL did not further increase the accumulation of mutated FTH1 in lysosomes in the presence of testosterone (Figure 3L).

Since LAMP2A serves as a receptor and channel for transporting cytosolic proteins into lysosomes during CMA process, we confirmed the essential role of LAMP2A in FTH1 CMA‐degradation. The knockdown of *LAMP2A* in TS^CT^ cells significantly suppressed testosterone‐induced FTH1 degradation without affecting its mRNA level (Figure 3M and Figure S5C, Supporting Information). Conversely, *LAMP2* overexpression did not alter basal FTH1 expression but did affect its testosterone‐induced degradation and lysosomal colocalization (Figure S5D–H, Supporting Information). Collectively, these findings demonstrate that androgens promote FTH1 degradation via the CMA pathway, a process dependent on Hsc70 recognition and LAMP2A‐mediated lysosomal translocation (Figure 3N).

### 2.6 AR Transcriptionally Activates LAMP2A to Promote CMA Activation and FTH1 Degradation

**Figure 4:**
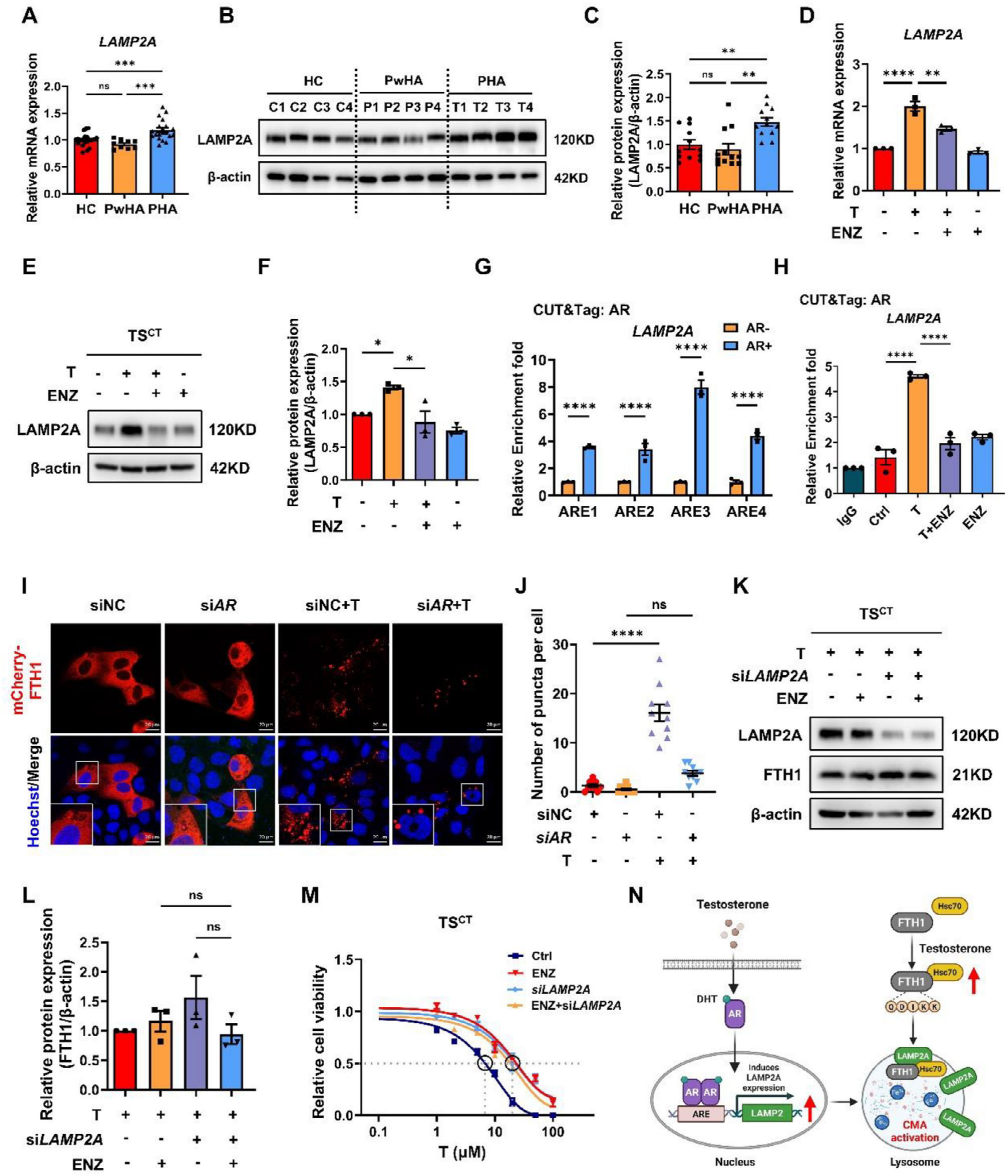
Androgen treatment induces FTH1 degradation through AR‐mediated LAMP2A upregulation. A) RT–qPCR analysis of *LAMP2A* gene expression in human villi from the HC, PwHA, and PHA groups. n = 12 independent villus samples were included in the HC and PHA groups, and nine independent villus samples were included in the PwHA group. B) Representative Western blot analysis of LAMP2A in villi taken between the sixth and eighth weeks from the HC, PwHA, and PHA groups. n = 4 independent biological samples in each group. C) Statistical quantification of the LAMP2A protein level of each villus sample normalized to its β‐actin level. n = 12 independent biological samples were included in each group. D) RT–qPCR analysis of *LAMP2A* mRNA levels in TS^CT^ cells treated with T and/or ENZ for 24 h. n = 3 biological replicates were included in each group. E) Western blot analysis showing LAMP2A protein level in TS^CT^ cells treated with T and/or ENZ for 24 h. F) Statistical quantification of the LAMP2A protein level in each group normalized to that of β‐actin. The results of three independent experiments were analyzed. G) Quantitative PCR analysis followed by a CUT&Tag assay revealed that the AR protein could interact with numerous AREs on the *LAMP2* promoter in AR‐overexpressing HEK293T cells. n = 3 biological replicates were included in each group. H) CUT&Tag‒qPCR results showing the occupancy of AR on the human *LAMP2* ARE1 region in TS^CT^ cells treated with T and/or ENZ for 24 h. n = 3 biological replicates were included in each group. I) Representative immunofluorescence images of mCherry‐FTH1 (red) in TS^CT^‐siNC and TS^CT^‐si*AR* cells treated with or without T for 24 h. Hoechst 33 342 indicated the nucleus. Scale bars, 20 µm. J) Statistical quantification of mCherry‐FTH1 puncta per cell. Ten cells in each group were analyzed. K) Western blot analysis of siNC and si*LAMP2A*‐TS^CT^ cells pretreated with DMSO or ENZ for 24 h, followed by T treatment for another 24 h. L) Statistical quantification of the FTH1 protein levels in each group normalized to those of β‐actin. The results of three independent experiments were analyzed. M) Analysis of the viability of siNC‐ and si*LAMP2A*‐transfected TS^CT^ cells pretreated with DMSO or ENZ for 24 h, followed by treatment with the indicated concentration of T for another 24 h. The results of three independent experiments were analyzed. (N) Schematic depiction of how androgen‐AR activates CMA by upregulating *LAMP2A* mRNA expression. The data are presented as the mean ± SEM. Statistical analysis was performed using one‐way ANOVA in (A), (C), (D), (F) to (H), (J) and (L), and a standard curve was interpolated with Sigmoidal, 4PL, where X is the concentration model in (M). ns, not significant; *p < 0.05, **p < 0.01, ***p < 0.001, ****p < 0.0001. HC, healthy control; PwHA, patients with PCOS without hyperandrogenemia; PHA, patients with PCOS with hyperandrogenemia; AR, androgen receptor; ARE, androgen response element. T, testosterone; ENZ, enzalutamide; siNC, negative control siRNA. T: 20 µM; ENZ: 5 µM.

We observed that testosterone exposure increased LAMP2A protein levels (Figure 3L), in contrast to the decrease in FTH1 expression. Similarly, compared with those from the HC and PwHA groups, the villi from the PHA group presented higher LAMP2A mRNA and protein levels (**Figure** **4**A–C). Furthermore, LAMP2A expression correlated with AR levels across trophoblast cell lines: it was higher in TS^CT^ and JEG3 cells (high AR expression) but lower in HTR8 and HEK293T cells (low AR expression) (Figure S6A, Supporting Information). These observations prompted us to investigate whether androgens induce LAMP2A expression to activate CMA and promote FTH1 degradation.

Testosterone treatment upregulated LAMP2A expression in TS^CT^ cells, whereas the AR antagonist enzalutamide suppressed both its basal expression and testosterone‐induced expression (Figure 4D–F and Figure S6A, Supporting Information). Correspondingly, *AR* knockdown also attenuated FTH1 upregulation (Figure S6B–D, Supporting Information). Conversely, transient *AR* overexpression in HTR8 cells was sufficient to increase LAMP2A expression upon testosterone stimulation (Figure S6E–G, Supporting Information). Bioinformatic analysis of the LAMP2 promoter identified four putative AREs (Figure S6H, Supporting Information). CUT&Tag assays confirmed that AR could bind to all four AREs, which was enhanced by testosterone and inhibited by enzalutamide (Figure 4G,H). These results demonstrate that AR directly transactivates LAMP2A expression.

Next, we examined whether enhanced CMA activity is mediated by AR. To validate this, we monitored the lysosomal delivery of mCherry‐FTH1. Testosterone significantly increased the number of mCherry‐positive puncta in control TS^CT^ cells, indicating enhanced CMA activity, whereas this effect was abolished upon *AR* knockdown (Figure 4I–J). Additionally, enzalutamide failed to increase endogenous FTH1 expression in *LAMP2A*‐knockdown cells treated with testosterone (Figure 4K,L). Furthermore, *LAMP2A* knockdown significantly inhibited testosterone‐induced ferroptosis, which could not be further suppressed by enzalutamide (Figure 4M). Collectively, these results suggest that testosterone enhances CMA‐mediated FTH1 protein degradation, which is driven by the AR‐dependent transcriptional upregulation of LAMP2A (Figure 4N).

### 2.7 Metformin Directly Binds to the AR Ligand‐Binding Domain to Antagonize Dihydrotestosterone‐Induced Stabilization

**Figure 5:**
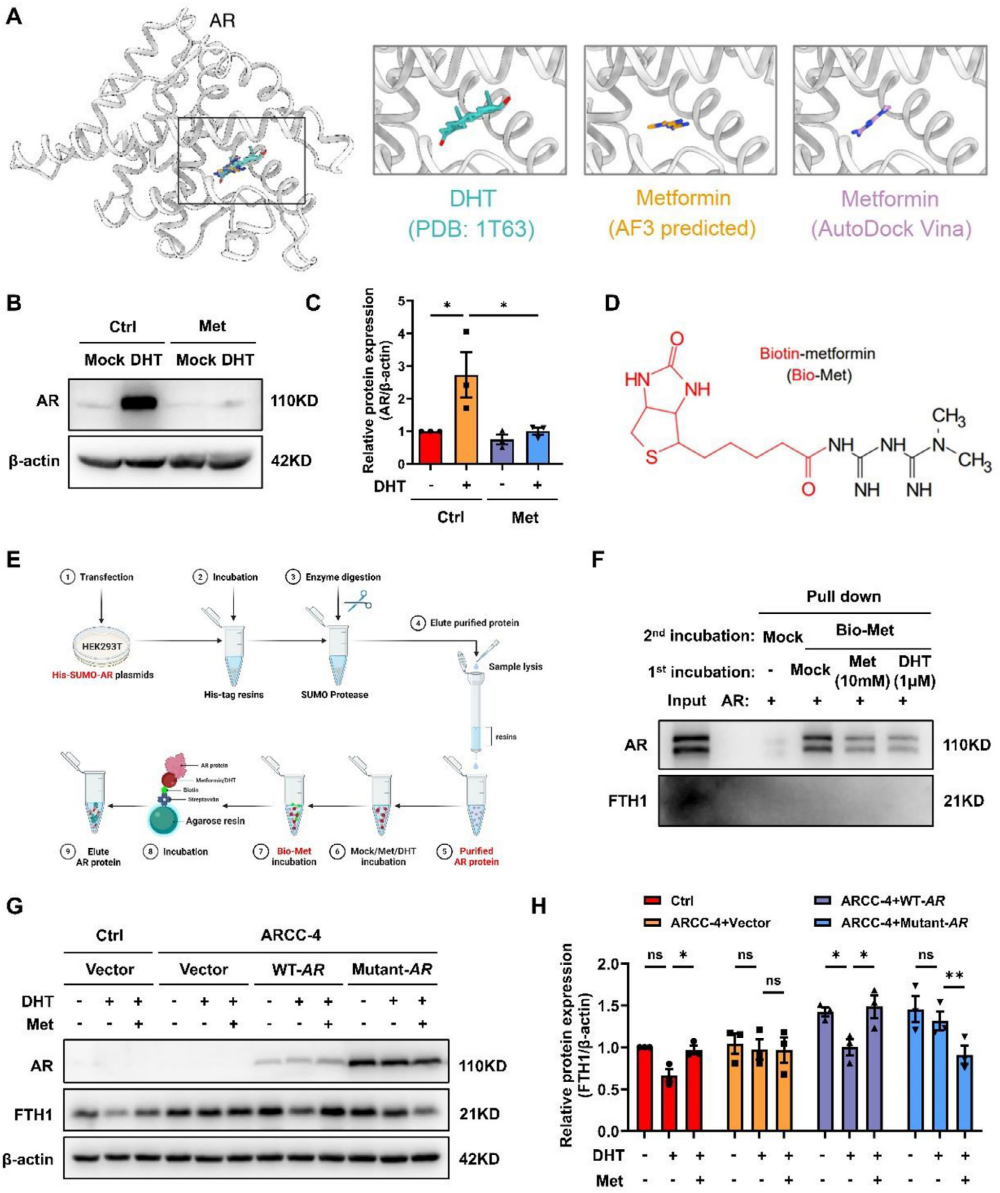
Metformin reduces the stability of AR through competitive binding to AR with DHT. A) Molecular docking of human AR protein and DHT/metformin. B) Western blot showing AR expression in TS^CT^ cells pretreated with or without Met for 12 h, followed by DHT incubation for another 12 h. C) Statistical quantification of AR protein levels in each group normalized to that of β‐actin. The results of three independent experiments were analyzed. D) Chemical structure of biotin‐labeled metformin (bio‐Met). E) Schematic of the purification and bio‐Met capture assay for the recombinant AR protein. F) The purified AR protein was preincubated with ddH_2_O, 10 mM free Met, or 1 µM DHT, followed by incubation with 2 mM bio‐Met. Western blot analysis of AR protein expression was conducted to detect the interaction between AR and bio‐Met which was captured by streptavidin‐coated agarose. n = 3 independent experiments were performed. G) TS^CT^ cells were pretreated with ARCC‐4 for 24 h, followed by reconstitution with WT or mutant AR (deletion of LSSLNELG between residues 702 and 709) plasmids. The designated cells were then incubated with or without Met for 12 h, followed by DHT treatment for another 12 h. The protein levels of AR and FTH1 were measured by Western blotting. H) Statistical quantification of the FTH1 protein levels in each group normalized to those of β‐actin. The results of three independent experiments were analyzed. The data are presented as the mean ± SEM. Statistical analysis was performed using one‐way ANOVA in (C) and (H). ns, not significant; *p < 0.05, **p < 0.01. DHT, dihydrotestosterone; Met, metformin; Bio‐Met, biotin‐metformin; Del, deletion. DHT: 100 nM; ARCC‐4: 5 µM; Met: 5 mM.

Androgens enhance AR stability by inducing AR dimerization and nuclear translocation.^[^ ^42^ ^]^ Since testosterone is less effective than DHT in stabilizing the AR and has a faster dissociation rate,^[^ ^43^ ^]^ we chose to use DHT in the following experiments. Molecular docking revealed that metformin occupies the known hormone‐binding pocket within the ligand binding domain of AR, overlapping with the DHT binding site (**Figure** **5**A). Pretreatment with metformin attenuated DHT‐induced AR stabilization at the protein level but not at the mRNA level in TS^CT^ cells (Figure 5B,C and Figure S7A, Supporting Information). To assess direct binding, we performed a pull‐down assay using biotin‐conjugated metformin (bio‐Met) (Figure 5D,E). Bio‐Met exhibited binding affinity for the AR protein but not for FTH1, and this interaction was disrupted by preincubation with free metformin or DHT (as positive controls) (Figure 5F). Furthermore, a thermal stability assay further indicated that metformin binding altered the AR conformation, thereby reducing its thermal stability, providing additional evidence for the direct interaction between metformin and AR (Figure S7B, Supporting Information).

We next performed protein structure‐based docking calculations with a high‐resolution structure of AR (PDB entry: 8E1A) to characterize the binding mode. H‐bonds formed between metformin and several amino acids, such as Leu705 and Asn706 (Figure S7C and Table S4, Supporting Information). Notably, the LSSLNELG residues (702–709 aa) in AR are highly conserved across different species (Figure S7D, Supporting Information). To verify the metformin binding site of AR, we generated *AR* mutant plasmids lacking LSSLNELG residues, which may be involved in AR–metformin binding. To evaluate the functional relevance of the LSSLNELG residues in increased FTH1 expression, we first degraded the endogenous AR protein using ARCC‐4, a PROTAC that selectively targets AR, and reconstituted cells overexpressing WT‐*AR* or mutant‐*AR*. Compared with the WT‐*AR‐*overexpressing group, metformin failed to rescue the decrease in FTH1 protein levels induced by DHT in the mutant*‐AR*‐ overexpressing group (Figure 5G,H). Consistently, DHT upregulated *FTH1* mRNA in cells overexpressing either WT or mutant *AR*, whereas metformin suppressed this effect only in WT *AR*‐reconstituted cells (Figure S7E, Supporting Information), indicating that the LSSLNELG residues are essential for metformin–AR binding. In summary, metformin may act as a competitive AR antagonist, binding to the ligand‐binding domain of AR to disrupt its DHT‐induced stabilization.

### 2.8 Metformin Alleviates Placental Ferroptosis and Prevents Early Pregnancy Loss in PCOS Model Mice

**Figure 6:**
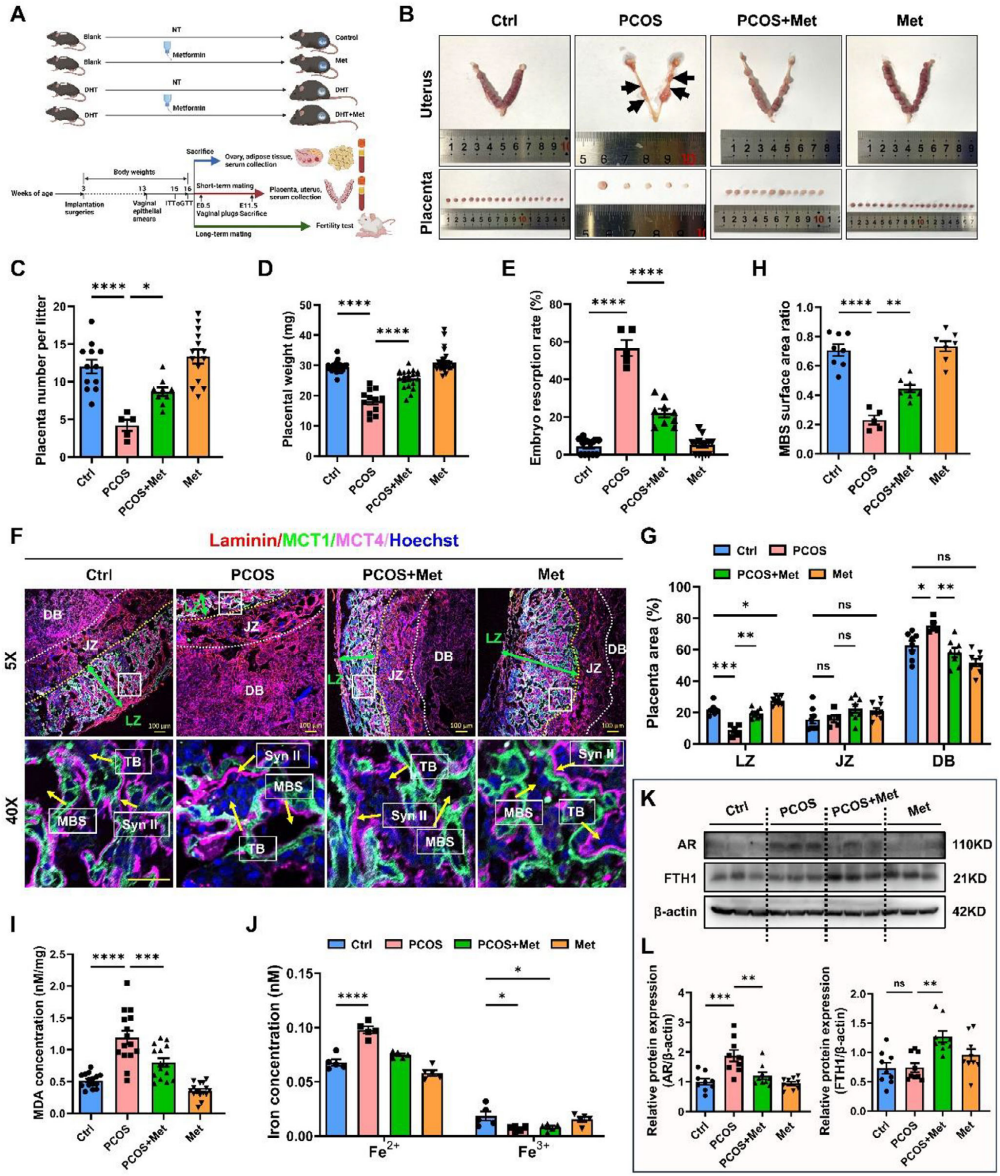
Metformin ameliorates ferroptosis and reduces early pregnancy loss rates in a mouse model of PCOS. A) Schematic depiction of the experimental setup. The mice were randomly assigned to either the blank (n = 60) or the PCOS (n = 60) group on postnatal day 21. Half of the dams in each group were fed 500 mg kg^−1^ metformin daily from 3 to 16 weeks of age. Vaginal smears were examined daily for 2 weeks beginning at 13 weeks of age. ITTs and oGTTs were performed at 15 weeks of age. The mice were sacrificed at 16 weeks of age. The body weights of the mice were recorded, and blood samples and tissues were collected. B) Representative morphological images of the uteri and placentas from mice from the control, Met, PCOS, and PCOS + Met groups at E11.5. The black arrows indicated absorbed embryos. C) Placenta number per litter in different groups at E11.5. n = 12 litters in Ctrl group, n = 5 litters in PCOS group, n = 9 litters in PCOS + Met group, n = 14 litters in Met group. D) Placental weights in different groups at E11.5. n = 30 placentas were included in Ctrl group, n = 12 placentas were included in PCOS group, n = 21 placentas were included in PCOS + Met group, n = 30 placentas were included in Met group. E) Embryo resorption rate in different groups at E11.5. n = 12 litters in Ctrl group, n = 5 litters in PCOS group, n = 9 litters in PCOS + Met group, n = 14 litters in Met group. F) Representative immunofluorescence images of E11.5 mouse placentas from different groups stained for SynT I and SynT II with MCT1, MCT4, and laminin. Hoechst 33 342 indicated the nucleus. The white and yellow dotted lines indicated the boundaries of the DB/JZ and JZ/LZ layers, respectively. The green double‐sided arrows indicated the thickness of the labyrinth layer. Yellow arrows indicated the MBS, TB, and SynT II in the labyrinth layer. The top images are at 5× magnification, and the bottom images are at 40× magnification. Scale bars, 100 µm. G) Measurement of the ratio of placental areas (different placental zone areas normalized to the total placental area) in different groups at E11.5. n = 8 placentas were included in Ctrl, PCOS + Met, and Met groups, n = 5 placentas were included in PCOS group. H) Measurement of the ratio of the MBS surface area (MBS surface area normalized to the labyrinth zone area). n = 8 placentas were included in Ctrl, PCOS + Met, and Met groups, n = 5 placentas were included in PCOS group. I) Measurement of MDA levels in viable placentas at E11.5 from different groups. n = 15 placentas were included per group. J) Measurement of iron concentrations in viable placentas at E11.5 from different groups. n = 5 placentas were included per group. K) Western blot analysis and statistical results of AR and FTH1 expression in placentas from different groups at E11.5. n = 3 placentas were included in each group. L) Statistical quantification of AR and FTH1 protein levels in each placenta normalized to its β‐actin level. n = 9 placentas were included in each group. The data are presented as the mean ± SEM. Statistical analysis was performed using one‐way ANOVA in (C) to (E), (G) to (J) and (L). ns, not significant; *p < 0.05, **p < 0.01, ***p < 0.001, ****p < 0.0001. LZ, labyrinth zone; JZ, junctional zone; DB, decidua basal zone; MBS, maternal blood space; TB, trophoblast; SynT I, syncytiotrophoblast I; SynT II, syncytiotrophoblast II.

To verify the therapeutic potential of metformin in influencing placental development and ferroptosis in vivo, we established a PCOS mouse model with hyperandrogenemia via continuous DHT exposure for 3 months starting on postnatal day 21 (**Figure** **6**A). These mice recapitulated key PCOS‐like features, including abnormal estrous cycles (Figure S8A,B, Supporting Information), PCO‐like ovaries (Figure S8C,D, Supporting Information), increased body weight, elevated serum steroid levels, and impaired glucose tolerance (Figure S8H–K and Table S5, Supporting Information). Compared with those in control mice, serum levels of testosterone (T), DHT, and cholesterol (CHOL) clearly increased and high‐density lipoprotein (HDL) levels significantly decreased in PCOS‐model mice, whereas circulating 17β‐estradiol (E2), fasting blood glucose, triglyceride (TG), and low‐density lipoprotein (LDL) levels remained unchanged (Figure S8G and Table S5, Supporting Information). Metformin treatment partially reversed these metabolic disturbances in PCOS mice, reducing body weight and ameliorating dyslipidemia and hormonal imbalances (Figure S8A–O, Supporting Information).

DHT‐exposed dams exhibited poor pregnancy outcomes, reflected by smaller, pale fetuses, reduced litter sizes, and lower pup weights, although the sex ratio of the offspring was unaffected (Table S6 and Figure S8P–R, Supporting Information). Healthy fetuses were a healthy pink color, whereas growth‐restricted fetuses were usually small, hemorrhagic, and/or emaciated and pale. We found that offspring subjected to continuous DHT treatment were small, presented pale skin, had lower newborn weights and were in smaller litters on average (Figure S8P–R, Supporting Information); however, DHT did not significantly alter the sex ratio of the offspring. Metformin significantly reduced the body weights of DHT‐treated mice and somewhat normalized their abnormal estrous cycles and steroid hormone levels (Figure S8A–M, Supporting Information). Additionally, metformin efficiently reduced serum CHOL levels and increased HDL levels (Figure S8N,O, Supporting Information).

Notably, metformin significantly improved short‐term fertility outcomes. The placental morphology transitioned from ischemic to hyperemic (Figure 6B), a change that was accompanied by increases in placental number and weight (Figure 6C,D), and concurrently led to a decrease in the embryo resorption rate (Figure 6E). Immunofluorescence staining revealed the expansion of the labyrinth area (labeled by MCT1 and MCT4) and maternal blood spaces in metformin‐treated placentas (Figure 6F–H), indicating improvements in placental circulation and the efficiency of maternal–fetal exchange across the placenta. Metformin also restored fetal morphology, litter size, and newborn weight in PCOS mice (Figure S8P–R, Supporting Information) and enhanced the long‐term fertility of PCOS mice by shortening the interval between litters and increasing pup yield per litter (Figure S8S, Supporting Information).

We further evaluated whether metformin counteracts placental ferroptosis in this model. Metformin significantly reduced lipid peroxidation and iron accumulation in the placentas of PCOS mice (Figure 6I,J), suggesting that metformin attenuated ferroptosis in vivo. Mechanistically, metformin significantly decreased AR protein levels and restored FTH1 protein levels in DHT‐exposed placentas (Figure 6K,L). In summary, metformin ameliorates hyperandrogenemia‐related placental defects and pregnancy loss by suppressing AR‐driven ferroptosis, supporting its therapeutic potential for EPL in patients with PCOS.

## 3 Discussion

The high incidence of EPL among patients with PCOS with hyperandrogenemia has long been a perplexing challenge in reproductive medicine. Although the clinical association has been well established, the underlying pathogenic mechanisms and potential therapeutic targets remain largely elusive. This study provides compelling evidence that excessive androgens contribute to placental dysfunction via a previously unrecognized mechanism—the induction of ferroptosis in trophoblasts. Our results reveal FTH1, an iron storage protein, as a critical mediator of androgen‐induced ferroptosis in trophoblasts. Androgens exert dual effects on regulating FTH1 protein levels through the AR. Androgens directly increase FTH1 protein synthesis while concurrently inducing FTH1 protein degradation via the Hsc70–LAMP2A‐mediated CMA pathway. Collectively, these findings expand our mechanistic understanding of PCOS‐associated EPL and reveal that the AR–CMA–FTH1–ferroptosis axis is a promising target for therapeutic intervention.

This study revealed that androgen‐induced ferroptosis arises primarily from disrupted iron metabolism, a mechanism distinct from the previously reported characterized disturbances in lipid and amino acid metabolism pathways. Mechanically, our pharmacological and genetic loss‐ or gain‐of‐function studies demonstrated that AR directly promotes FTH1 protein synthesis to counteract ferroptosis, while simultaneously activating the CMA pathway to accelerate FTH1 degradation in the presence of testosterone to facilitate ferroptosis. These findings suggest a dual regulatory function of AR in ferroptosis. NCOA4‐mediated ferritinophagy involves canonical selective autophagy‐dependent degradation of ferritin.^[^ ^44^, ^45^ ^]^ Conceptually, these findings broaden the current understanding of ferritinophagy by revealing an alternative ferritin degradation route that is mechanistically distinct from the classical NCOA4‐dependent macroautophagy pathway.

Metformin enhances fertility in patients with PCOS by improving oocyte quality, promoting ovulation, and facilitating embryo implantation.^[^ ^46^, ^47^, ^48^, ^49^, ^50^ ^]^ Our study reveals a previously unrecognized mechanism through which metformin improves the fertility of patients with PCOS, highlighting its role in promoting placental development. Mechanistically, metformin is commonly used to ameliorate metabolic disturbances in patients with PCOS because of its insulin‐sensitizing and androgen‐lowering properties. Previous studies have demonstrated that metformin exerts its antidiabetic effects by targeting AMP‐activated protein kinase (AMPK) signaling and complex I of the mitochondrial respiratory chain.^[^ ^51^, ^52^ ^]^ Notably, metformin exerts its antiandrogenic effect by suppressing androgen biosynthesis through the suppression of the activity of steroidogenic enzymes (HSD3B2 and CYP17A1).^[^ ^53^ ^]^ In this study, we show that the competitive binding between metformin and androgens to the AR ligand‐binding domain decreases its protein levels. Our findings provide novel evidence for the androgen antagonist effect of metformin, supporting the use of metformin in the treatment of patients with PCOS with hyperandrogenism.

Given the complex and multifactorial nature of metformin's actions, we further sought to specifically assess whether direct suppression of AR could improve placental development. Flutamide, a clinically approved AR antagonist, is widely used to treat androgen‐dependent accessory genital organ disorders^[^ ^54^ ^]^ and has been shown to improve metabolic dysfunction in patients with PCOS with hyperandrogenemia.^[^ ^55^, ^56^ ^]^ Because androgen retards the transport of the embryo in the fallopian tubes (at E2.5–5.5), results in aberrant preimplantation and subsequently delays timely implantation in rodents, we constructed an acute DHT‐exposed mouse model during pregnancy to better understand the effects of androgens on placental formation. We subcutaneously injected DHT or flutamide or provided drinking water containing metformin daily from E5.5 (the time of implantation completion) to E11.5 (Figure S9A, Supporting Information). Our results indicated that both flutamide and metformin markedly upregulated placental FTH1 expression and elevated iron ion levels in DHT‐exposed mice (Figure S9B,C, Supporting Information). Notably, metformin exhibited greater efficacy than flutamide in improving placental nutrient exchange, primarily through expansion of the labyrinth layer (Figure S9D,E, Supporting Information), thereby markedly reducing embryonic resorption rates (Figure S9F,G, Supporting Information). This effect may be attributable to the potential reproductive toxicity of flutamide, which has adverse effects on embryonic and placental development. Moreover, in addition to its antiandrogenic activity, metformin improves insulin sensitivity and regulates glucose and lipid metabolism. In addition to competitively binding to the AR with DHT and destabilizing the AR protein, metformin also directly suppresses ovarian androgen synthesis, lowering serum androgen levels. Furthermore, a recent study demonstrated that metformin directly chelates iron ions to form a complex,^[^ ^57^ ^]^ suggesting an additional mechanism by which metformin may regulate ferroptosis‐mediated EPL in PCOS patients with hyperandrogenemia.

Although metformin reduces the early pregnancy loss rate in PCOS mice, its safety profile requires further verification. This concern is underscored by evidence that metformin readily crosses the placenta via active transporters, resulting in substantial fetal exposure, as demonstrated by the high fetal concentrations detected following maternal administration during the third trimester.^[^ ^58^ ^]^ Moreover, emerging data from RCTs have linked in utero metformin exposure to greater adiposity and higher BMI in offspring during childhood.^[^ ^59^, ^60^ ^]^ However, recent research has shown that compared with insulin, metformin use during pregnancy does not have adverse effects on long‐term outcomes in children and their mothers.^[^ ^61^, ^62^ ^]^ Given these findings, the safety of metformin use during pregnancy remains controversial. Further longitudinal studies are warranted to clarify its long‐term effects on offspring health. Moreover, there is an urgent need to develop safer therapeutic alternatives for pregnant women, guided by mechanistic insights into maternal–fetal metabolic crosstalk.

In addition to AR, our findings suggest potential molecular targets for new therapeutic interventions. For instance, inhibition of the CMA pathway may represent an alternative strategy for inhibiting ferroptosis. In fact, targeting LAMP2A, a key molecule in the CMA pathway, is only partially effective as inhibition of LAMP2A suppresses the degradation of ferroptosis‐related proteins with KFERQ‐like motifs. In particular, trophoblasts may also experience an influx of extracellular ferric ion when exposed to iron‐rich environments; under such conditions, increasing Fe^2+^ chelation may represent a more effective therapeutic intervention. Currently, directly increasing ferritin levels in trophoblasts remains unachievable. Paradoxically, women with PCOS often exhibit elevated serum ferritin levels, which correlate with their hyperandrogenic status.^[^ ^63^ ^]^ Beyond its impact on iron metabolism, our study also revealed significant alterations in other DEGs involved in amino acid metabolism and antioxidant processes, including *SLC7A11* and *CYBB*, which warrants further investigation.

During pregnancy, reproductive hormones undergo dynamic interconversion and are tightly regulated to maintain a stable equilibrium, which is essential for pregnancy maintenance and fetal development.^[^ ^64^, ^65^, ^66^ ^]^ Serum androgen levels gradually increase from the first trimester and peak toward the end of pregnancy. However, excessive elevations in circulating androgen levels during gestation have been associated with adverse maternal and fetal outcomes, including EPL, preeclampsia, preterm delivery, and impaired neonatal development.^[^ ^3^, ^67^ ^]^ Since androgens can be aromatized into estrogens via aromatase, PCOS patients with impaired aromatase activity often have relatively low estrogen levels. Our study demonstrated that gestational serum estrogen levels are lower in PCOS patients with hyperandrogenemia than in healthy individuals (Table S13, Supporting Information), which is consistent with the findings of previous reports.^[^ ^68^ ^]^ Recent research has demonstrated that estrogen regulates iron metabolism by inhibiting TFR1 expression while increasing ferroportin and ferritin levels in doxorubicin‐treated ovarian and breast cancer cells.^[^ ^69^ ^]^ Estrogen can also increase ferroptosis resistance by promoting the anti‐ferroptotic hydropersulfide system and suppressing ether lipid plasticity through estrogen receptor alpha (ESR1) in renal tubular epithelial cells,^[^ ^70^ ^]^ suggesting that estrogen protects against ferroptosis. Low levels of estrogens may combine with excessive androgens to induce trophoblast ferroptosis, but little evidence on the regulation of ferroptosis by estrogen in trophoblasts has been reported. Thus, we focused on investigating how estrogen regulates iron metabolism in trophoblasts and elucidating the underlying molecular mechanisms involved.

**Figure 7:**
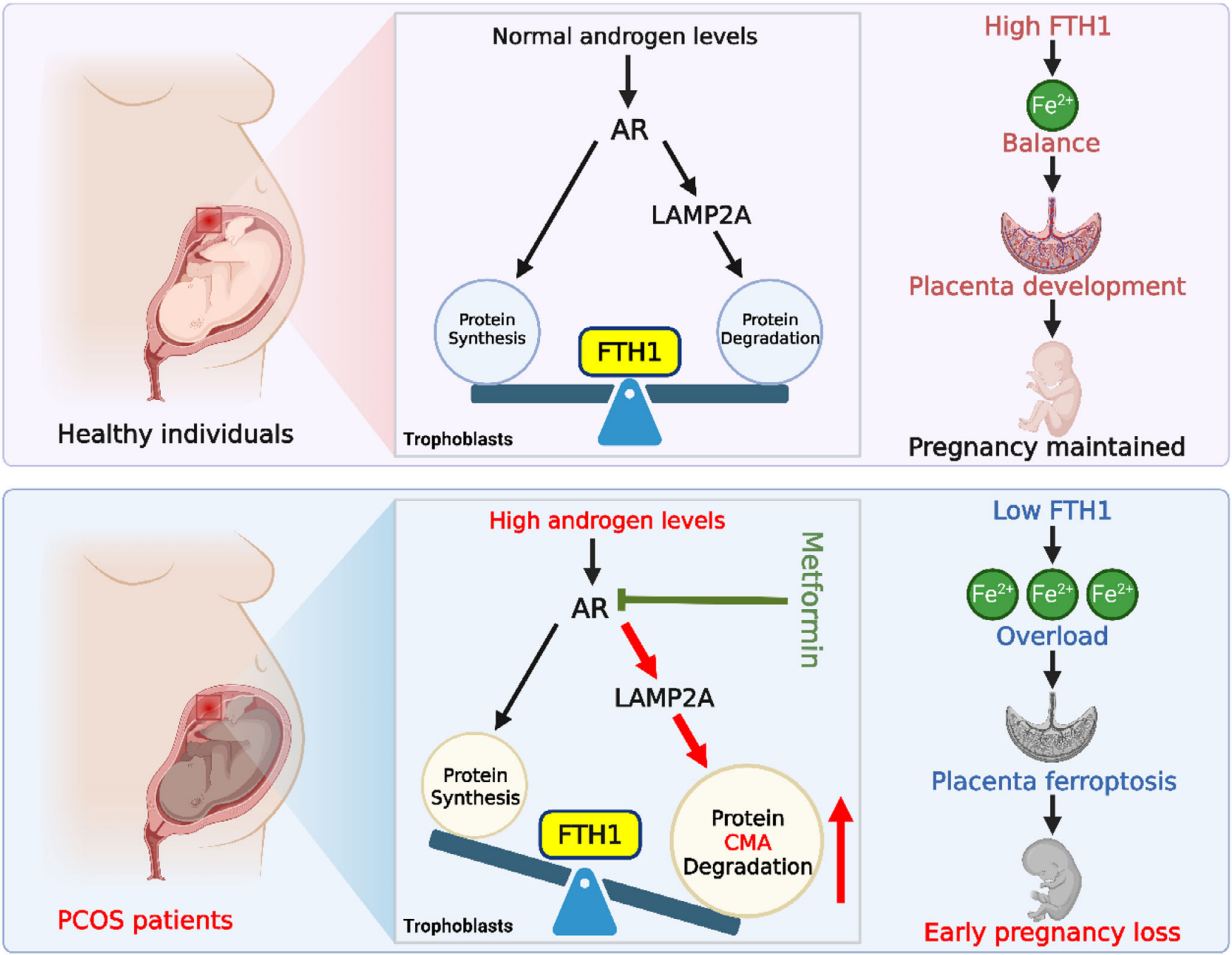
Schematic of the ferroptosis mechanism induced by hyperandrogenemia in trophoblasts in patients with PCOS. Androgens promote FTH1 synthesis to resist ferroptosis via AR while simultaneously activating the LAMP2A‐mediated CMA pathway to subsequently accelerate FTH1 degradation and ferroptosis. In healthy individuals, the synthesis and degradation of FTH1 are in equilibrium in the presence of normal androgen levels, ensuring that intracellular Fe^2+^ concentrations remain within a physiological range, thereby preventing ferroptosis and sustaining pregnancy. In patients with PCOS, the rate of FTH1 degradation surpasses that of its synthesis, leading to a decrease in FTH1 levels and the accumulation of intracellular Fe^2+^, ultimately inducing ferroptosis and pregnancy loss. Metformin improves pregnancy outcomes in individuals with PCOS by increasing FTH1 levels via AR antagonism.

In conclusion, our study demonstrated that ferroptosis contributes to EPL in PCOS patients with hyperandrogenemia, with disrupted iron metabolism playing a central role in this process. We further propose a mechanism whereby excessive androgen signaling promotes Fe^2^⁺ accumulation by disrupting the balance between ferritin synthesis and degradation through AR‐dependent pathways (**Figure** **7**). Moreover, we demonstrated that metformin exerts somewhat optimal antiandrogenic effects by competitively binding to the AR with androgens, thereby destabilizing the AR protein. Furthermore, metformin improved early placental development and reduced EPL in hyperandrogenic PCOS mice, supporting its therapeutic potential for treating EPL in PCOS patients with hyperandrogenemia. Collectively, our findings elucidate a novel mechanism by which hyper‐androgen triggers trophoblast ferroptosis through disrupting iron homeostasis. These insights provide a conceptual framework for developing targeted therapeutic strategies for treating PCOS patients and their associated pregnancy complications.

## 4 Experimental Section

### Villous Explant Culture

Matrigel (8 mg mL^−1^) was added to a 24‐well plate and incubated at 37 °C to form a gel drop. The placental villus tips were dissected into small pieces (2–3 mm). Each piece was carefully placed on the top of the gel, covered with 50 µL of medium and incubated at 37 °C with 5% CO_2_ in air for 4–6 h to allow anchorage. Explants were subsequently cultured in 500 µL of DMEM/F‐12 supplemented with 10% carbon‐adsorbed fetal bovine serum (CA–FBS) and 1% penicillin–streptomycin solution (P/S) and subjected to the indicated treatments. The outgrowth of the explants was recorded and photographed on day 1 and day 4 under a microscope (Carl Zeiss Primovert, Germany). ImageJ software was used to measure the longest expansion length of the explants on day 4 in each image.

### Primary TSC Isolation and Differentiation

The isolation and culture of human TS^CT^ cells were performed following a published protocol.^[^ ^36^ ^]^ Three first‐trimester placental villi from healthy mothers were collected. The sex and developmental stage of each human sample used for the derivation of TS^CT^ cells are summarized in Table S7, Supporting Information. In brief, first‐trimester placental villi were collected and cut into small pieces. All the tissues were enzymatically digested three times in a mixture containing equal amounts of TrypLE and Accumax for 20 min at 37 °C. The cell suspensions were then filtered through a 70 µm mesh filter (BD Falcon, 352 350, USA). ITGA6 is a widely used cell lineage marker for CTBs. CTBs were immunomagnetically purified using an EasySep phycoerythrin (PE)‐positive selection kit and a PE‐conjugated anti‐ITGA6 antibody. The selected cells were seeded in a 6‐well plate (Corning, USA) coated with 5 µg mL^−1^ collagen IV at a density of 0.5—1 × 10^6^ cells per well and cultured in 2 mL of TS medium. CTBs were dissociated with TrypLE for 10–15 min at 37 °C. Cells at passages 10–30 were used for analyses. For ST(2D)‐TS^CT^ cell differentiation, TS^CT^ cells were collected and then seeded into a 6‐well plate precoated with 2.5 mg mL^−1^ collagen IV at a density of 1 × 10^5^ cells per well. The ST(2D) medium was replaced at day 3, and the culture was continued for 3 days. Information regarding the TS medium is presented in Table S8, Supporting Information.

### Cell Lines and Culture

All cell lines cultured in this study were obtained from the American Type Culture Collection (ATCC). In detail, Dulbecco's Modified Eagle Medium (DMEM) supplemented with 10% CA–FBS and 1% P/S was prepared for culturing HEK293T cell lines. HTR8 and JEG3 cell lines were cultured in RPMI 1640 supplemented with 10% CA–FBS and 1% P/S. The cells were cultured at 37 °C in a humidified 95% air/5% CO_2_ incubator. The culture medium was changed every other day. After the cell density reached ≈80–90% confluence, the cells were digested with 0.25% trypsin‐EDTA solution.

### RNA‐seq

TS^CT^ cells were seeded into 6‐cm cell culture dishes and treated with 20 µM testosterone for 24 h. Three replicate samples were prepared for each group. The samples were then processed for reverse transcription, cDNA purification and library construction by LC‐Bio Technologies Co., Ltd. (Hangzhou, China). The results were analyzed on the Illumina Nova Seq 6000/MGISEQ‐T7 platform. Differentially expressed genes were analyzed with significance criteria of log2 (fold change) ≥ 2 and multitest adjusted *p* ≤ 0.05 by Cufflinks and R. GO and KEGG pathway analyses were performed using clusterProfiler. The processed RNA‐seq data with fragments per kilobase per million (FPKM) values of all the genes are presented in Table S9, Supporting Information.

### RNA Isolation and qRT‒PCR Assays

Total RNA was isolated from TS^CT^ cells using an RNA‐Quick Purification Kit in accordance with the manufacturer's protocol. Total RNA (1 µg) was reverse transcribed with HiScript II Reverse Transcriptase; ChamQ Universal SYBR qPCR Master Mix and the CFX96 system (Bio‐Rad, USA) were used for real‐time qPCR. β‐Actin was used as an endogenous control. The amplification conditions were as follows: a. denaturation at 95 °C for 30 s; b. annealing at 95 °C for 10 s; and c. extension at 60 °C for 30 s for 40 cycles. The Ct values were collated, and the 2^−ΔΔCt^ method was used to calculate the relative expression levels of target genes. The sequences of the primers designed for human target transcripts are presented in Table S10, Supporting Information.

### CUT&Tag and qRT‒PCR Assays

The CUT&Tag assays were performed using the Hyperactive universal CUT&Tag assay kit according to the manufacturer's instructions. Briefly, prepared concanavalin A‐coated magnetic beads (ConA beads) were added to resuspended cells and incubated at room temperature to bind cells. The nonionic detergent digitonin was used to permeate the cell membrane. An anti‐AR (CST, 5153, 1:100), anti‐H3K4me3 (positive control, 1:100), and rabbit IgG (negative control; 1:100) antibodies were subsequently added. The secondary antibody (1:100) and hyperactive pA‐Tn5 transposase were then incubated with the cells bound to the ConA beads so that the hyperactive pA‐Tn5 transposase can cleave the exact DNA fragments containing specific AR binding sites that are bound to the AR protein. In addition, the cut DNA fragments were purified using spin columns, quantified, and stored for further qPCR analysis. Primers (Table S11, Supporting Information) that flank the predicted putative AREs were designed, and qPCR was performed to identify ligand‐dependent AR binding to AREs in the *LAMP2* or *FTH1* gene promoters.

### Western Blotting

Protein samples were extracted from tissues and cells using ice‐cold RIPA lysis buffer supplemented with 1% phenylmethanesulfonyl fluoride (PMSF). The concentrations of proteins in the supernatant were determined using a Pierce bicinchoninic acid (BCA) protein assay kit. Equal quantities of protein samples were then mixed with loading buffer, heated for 10 min at 100 °C, separated by sodium dodecyl sulfate–polyacrylamide gel electrophoresis (SDS–PAGE) and subsequently transferred to polyvinylidene difluoride (PVDF) membranes (Millipore, USA). After blocking with 5% skim milk at room temperature for at least 1 h, the membranes were incubated with primary antibodies overnight at 4 °C. After being washed in Tris‐buffered saline with Tween 20 (TBST) buffer, the membranes were incubated with horseradish peroxidase‐conjugated anti‐rabbit or anti‐mouse IgG secondary antibodies (1:5000) and visualized by enhanced chemiluminescence. The images were captured using a ChemiDoc Touch Imaging System (Bio‐Rad, USA). ImageJ software was used to quantify the intensities of the Western blot bands. All the antibodies used in the immunoblot analysis are listed in Table S14, Supporting Information.

### Immunofluorescence Staining

The cells were fixed with 4% paraformaldehyde for at least 30 min and then permeabilized with 0.3% Triton X‐100 for 5 min at room temperature. The frozen sections were permeabilized with 0.3% Triton X‐100 for 10 min at room temperature. After blocking in 2% goat serum for 1 h at room temperature, the cells or sections were incubated with primary antibodies overnight at 4 °C. After being washed with TBST buffer, the cells or sections were incubated with Alexa Fluor 488‐, Alexa Fluor 568‐, or Alexa Fluor 647‐conjugated secondary antibodies (1:500) for 1 h at room temperature. The nuclei were stained with Hoechst 33 342 Stain Solution (1:500) for an additional 10 min, and images were captured with a laser scanning confocal microscope (Zeiss, LSM 800, Germany).

### siRNA and Plasmid Transfection

TS^CT^ cells were cultured in 12‐well plates and transfected with siRNAs targeting *ATG5* (si*ATG5*), *ATG7* (si*ATG7*), *AR* (si*AR*), *FTH1* (si*FTH1*), *Hsc70* (si*Hsc70*), or *LAMP2A* (si*LAMP2A*) or negative control siRNA (siNC) with Lipofectamine 3000 Transfection Reagent (Thermo Fisher Scientific, L3000015, USA) following the manufacturer's instructions. The negative control (NC), *ATG5*, *ATG7*, *FTH1*, *Hsc70*, and *LAMP2A* siRNAs were purchased from Ribo Bio Co., Ltd. (Guangzhou, China), and the *AR* siRNA was obtained from Santa Cruz (sc‐29204, USA). TS^CT^ cells were allowed to recover in fresh growth medium for 48 h to permit gene silencing, with or without chemical stimulation for another 24 h. To overexpress genes in TS^CT^ cells, plasmids containing full‐length gene sequences were designed by PPL Bio Co., Ltd. (Nanjing, China) and obtained from other companies. TS^CT^ cells at 30–40% confluence were seeded in 12‐well plates the day before transfection. The next day, TS^CT^ cells were transfected with plasmids using Lipofectamine 3000 Transfection Reagent following the manufacturer's instructions. After 6 h, the medium was replaced, and the cells were cultured for another 72 h. Successful transfection was confirmed by qPCR and Western blotting. Primers for siRNAs and plasmids are presented in Tables S12 and S14, Supporting Information.

### Site‐Directed Mutagenesis

The FTH1 and AR amino acid sequences were obtained from https://www.uniprot.org/. Mutation of the KFERQ sequence of FTH1 was performed using the QuikChange II site‐directed mutagenesis kit with the following primers:

FTH1: 5′‐CACAGTCTGGTTTCTTGATAGCCGCAAGGAAGATTCGGCCACCTC‐3′;

The presence of the desired mutations and the absence of additional mutations within the coding sequence were confirmed by DNA sequencing.

### Cell Proliferation Assay

TS^CT^ cells were seeded in 96‐well plates at a density of 1 × 10^4^ cells per well and incubated with the indicated drugs for 24 h. Subsequently, 100 µL of fresh medium containing 20 µL of MTS solution was added to the cells and incubated for 1 h in an incubator with 5% CO_2_ at 37 °C. The absorbance at 490 nm was measured using a microplate reader.

### Measurement of MDA Levels

Lipid peroxidation generation in tissues and cells were monitored following the MDA Assay Kit instructions. Individual contents of MDA were measured at 532 nm with a microplate fluorometer.

### Iron Measurements

Villi from HC and PCOS patients were harvested, at 10 mg was used for each assay. The tissues were rapidly homogenized in 4–10 volumes of iron assay buffer from an iron assay kit. The supernatants were collected after centrifugation at 16 000 × g for 10 min at 4 °C to remove insoluble materials. The samples and iron probe were added to the sample wells of a 96‐well plate following the manufacturer's protocols. After the samples were incubated at 37 °C for 30 min, the absorbance at 593 nm was measured using a microplate reader. The concentrations of iron (Fe^2+^), iron (Fe^3+^), and total iron (Fe^2+^ and Fe^3+^) in all groups were calculated according to the manufacturer's protocol.

### Transmission Electron Microscopy

TS^CT^ cells were harvested using TrypLE centrifuged at 400 × g for 5 min and gently washed with ice‐cold PBS with 5% bovine serum albumin (BSA) once. Then, cell pellets were washed with ice‐cold PBS and received an overnight fixation in 2.5% glutaraldehyde in sodium phosphate for 12 h at a pH of 7.2 at 4 °C. After rinsing with 0.1 M PBS three times for 15 min, cell pellets were postfixed with 1% osmium tetroxide in PBS for 1 h and subjected to gradient dehydration in ethanol (50%, 70%, and 90% for 15 min each and 100% for 20 min) and acetone (two times 100% for 20 min each) at room temperature (RT). Subsequently, cell pellets were infiltrated with a mixture of epoxy resin and acetone at 1:1 ratio for 2 h at RT and processed for epoxy resin embedding at 37 °C. Ultrathin sections were cut with a diamond knife using a Reichert ultramicrotome (Leica UC7, Germany) at 50–60 nm thickness, collected on 300 mesh copper grids, and stained with 3% uranyl acetate and counterstained with lead citrate before visualization. Samples were captured using a Tecnai T10100 kV electron microscope (Philips, Netherlands). Lysosome vacuoles were captured per 8300× field.

### Flow Cytometry

After being treated with 20 µM testosterone for 24 h, TS^CT^ cells were harvested gently using TrypLE centrifuged at 400 × g for 5 min and washed with ice‐cold PBS three times. Then, cells were stained with FITC‐conjugated Annexin V and propidium iodide (PI) from Cell Apoptosis Kit. Briefly, 5 µL FITC‐conjugated Annexin V and 1 µL 100 µg mL^−1^ PI solution was added, and the cells were vortexed gently and incubated for 15 min at room temperature in the dark. Finally, 400 µL 1× Annexin V binding buffer was added to each tube to stop the reaction. FITC‐positive cells were considered apoptotic cells, which were assessed by the flow cytometry. Images were analyzed by FlowJo software.

### Colony Forming Assay

TS^CT^ cells (1000 per dish) were cultured in 60 mm petri dishes in three replicates in TS medium and exposed to the drugs for 24 h. The drugs were removed and continuing cultured for 8 days. After 8 days, the cell colonies were stained with 0.1% crystal violet in 20% methanol post fixation with 100% ethanol. The number of colonies (cell counts > 50) were counted with the ImageJ software.

### Coimmunoprecipitation

TS^CT^ cells were transfected with FLAG‐tagged FTH1 expression plasmids for 48 h, followed by treatment with testosterone for another 24 h. After treatment, the cells were removed by scraping, and cell extraction buffer was added. The lysates were subsequently centrifuged at 16 000 × g at 4 °C for 10 min. Before IP, the samples were precleared with protein A+G agarose beads at 4 °C for 2 h and subsequently incubated with various unrelated IgG or anti‐FLAG M2 magnetic beads for 1 h at room temperature with gentle rotation. Following incubation, anti‐FLAG M2 magnetic beads were extensively washed with Tris‐buffered saline (TBS), and proteins were eluted by boiling in 4× sodium dodecyl sulfate sample buffer before SDS–PAGE. The samples were resuspended in 40 µL of SDS sample buffer and then boiled for 5 min. Finally, the samples were separated by SDS–PAGE and analyzed by Western blotting.

### Tandem Mass Spectrometry

TS^CT^ cells were treated with testosterone for 24 h, and the cells were harvested and processed for IP with anti‐FTH1 protein A+G agarose beads, followed by SDS–PAGE. The gel was stained with Coomassie blue and cut into sections for trypsin digestion. The digested gel fractions were processed for LC–MS/MS analysis on a Thermo Scientific Q Exactive Plus mass spectrometer (Thermo Fisher Scientific, USA). Mass spectrometry was performed at Lumingbio Co., Ltd. (Shanghai, China), and the resulting MS/MS data were analyzed with Proteome Discoverer.

### In Silico Analysis of the LAMP2 and FTH1 Promoters

Putative AR binding sites on the human *LAMP2* gene (NCBI ID: 3920) and human *FTH1* gene (NCBI ID: 2495) promoters were identified with the JASPAR database.^[^ ^71^ ^]^ Briefly, the entire upstream (−2 kb) sequence to downstream (+100 b) sequences from the TSSs were analyzed using human reference core matrices (MA0007.2) from the JASPAR transcription factor database, with a relative profile score threshold of 80%. The identified AR binding sites were utilized for the downstream CUT&Tag assay to assess the binding efficiency of the AR to the respective motifs, followed by qPCR analysis to determine whether AR binding to these motifs activates the *LAMP2* and *FTH1* transcriptional machinery.

### Structural Prediction of the Metformin–AR Complex

The structure of the complex of human AR and metformin was predicted using AlphaFold3 with the default parameters (ModelSeeds = 2).^[^ ^72^ ^]^ The pairwise ipTM score of the metformin–AR complex was 0.9 (>0.7 indicates a confident complex), and the pairwise min PAE value of the metformin–AR complex was 2.22 (<10 indicates high precision). The overall AF3 ranking score was 1.11. Molecular docking between AR and metformin was performed using AutoDock Vina.^[^ ^73^, ^74^ ^]^ The human AR structure (PDB ID: 1T63) and the metformin coordinates (CCD code: MT8) were sourced from the Protein Data Bank. The AR structure was prepared by removing ligands and water molecules and adding polar hydrogen atoms. Docking with an exhaustiveness of 32 yielded nine poses, with binding affinities ranging from −4.0 to −4.7 kcal mol^−1^. Seven of the nine metformin poses were located in the AR ligand‐binding pocket. All the structures were visualized using UCSF ChimeraX.^[^ ^75^ ^]^

### Thermal Shift Assays

HEK293T cells were transfected with pcDNA3.1(+)‐AR plasmids. After 48 h of transfection, the cells were collected, suspended in PBS and flash frozen with liquid nitrogen. The cells were lysed through three freeze–thaw cycles. The resulting cell lysates were incubated with metformin for 30 min on ice and then heated at various temperatures for 10 min. Next, the lysates were boiled for 10 min in loading buffer and subjected to Western blot analysis using an anti‐AR antibody.

### Purification of the Recombinant AR Protein

Vectors carrying the full‐length AR sequence fused with His and SUMO tags were transfected into HEK293T cells using Lipofectamine 3000 Transfection Reagent. After being cultured at 37 °C for ≈72 h post‐transfection, the cells were harvested and lysed in lysis buffer provided with the His‐tag protein purification kit. After centrifugation, the supernatant was incubated with His‐tag purification resins overnight at 4 °C to enrich the tagged recombinant proteins. The resins were subsequently washed with wash buffer consisting of 20 mM (HEPES pH 8.0), 150 mM NaCl, 0.1% CHAPS, 10% glycerol, and 2 mM DTT. After on‐column cleavage using SUMO protease at 4 °C for 16 h, the immobilized proteins were eluted from the resins and concentrated for further biochemical analysis.

### Bio‐Metformin Capture Assay

Metformin was labeled with biotin, and the product bio‐metformin (bio‐Met) was purified through sequential washing, extraction, and column chromatography and was subsequently validated by H‐NMR. The purified AR protein was preincubated with 20 mM free metformin for 1 h at 4 °C in binding buffer (20 mM Tris‐HCl, 100 mM KCl, 0.2% Triton X‐100, and protease inhibitors). The protein was subsequently incubated with 2 µM biotin‐labeled metformin for 1 h at 4 °C. Bio‐Met was then captured using high‐capacity streptavidin‐coated agarose over 16 h at 4 °C, followed by three washes with binding buffer. The agarose was then boiled in SDS–PAGE loading buffer and subjected to Western blot analysis using anti‐AR or anti‐FTH1 antibodies. To assess competitive binding between DHT and metformin for AR, the AR protein was preincubated with 1 µM DHT for 1 h at 4 °C, after which the capture assay was performed.

### Human Sample Procurement

The first‐trimester human villi tissues from HC individuals (n = 20), PCOS patients without hyperandrogenemia (n = 10), and PCOS patients with hyperandrogenemia (n = 20) were collected from women undergoing dilatation and curettage at the 6 to 8 weeks of gestation in the Department of Obstetrics and Gynecology of the Sir Run Run Shaw Hospital affiliated to the School of Medicine, Zhejiang University.

The study included healthy women aged 20 to 35 with normal ovarian morphology and ovulatory cycles, confirmed by ultrasound. They had a healthy full‐term pregnancy before and chose to have an elective abortion in a subsequent apparently healthy pregnancy at 6 to 8 weeks. The inclusion criteria for PCOS patients were: (a) Women aged 20 to 35; (b) Patients had at least two of the following signs: oligo‐ or anovulation, clinical and/or biochemical hyperandrogenemia, and polycystic ovaries on ultrasound, based on the Rotterdam PCOS Diagnostic Criteria after exclusion of related disorders. The exclusion criteria were: (a) Age > 35 years old or < 20 years old; (b) Patients had organic lesions of the uterus or malformation of reproductive tract; (c) Chromosomal abnormalities in both spouses or embryos; (d) In addition to PCOS, other causes of hyperandrogenemia, such as Cushing's syndrome, congenital adrenal hyperplasia, adrenal or ovarian tumors, or those taking androgen medication; (e) Patients with other endocrinal or metabolic diseases; (f) Autoimmune diseases, such as antiphospholipid antibody syndrome, Sjogren's syndrome, systemic lupus erythematosus; (g) Infection; (h) Patients had improper drug treatment, chemical hazards or radiation exposure; (i) Incomplete clinical data; and (j) Vulnerable groups, including patients with mental illness, cognitive impairment, or minors. The clinical characteristics of the recruited patients are provided in Table S13, Supporting Information.

### Animal Study—1 PCOS Mouse Model

PCOS was induced in female wild‐type C57BL/6 mice. For each experiment, age‐matched mice were used and randomly allocated to each experimental group. Animals were housed in groups of 4–6 mice per individually ventilated cage in a 12 h light‐dark cycle (6:30–18:30 light; 18:30–6:30 dark) with constant room temperature (21 ± 1 °C) and relative humidity (40–60%). Animals had access to food and water ad libitum.

On postnatal day 21, mice with similar body weights were randomly divided into DHT‐treated and control groups (n = 60 per group). In the PCOS model group, the mice were implanted subcutaneously with DHT‐releasing implants from the 3 weeks of age—a peripubertal stage in mice that corresponds developmentally to the onset of puberty in humans. These implants were produced by loading 1 cm silastic implants (internal diameter 1.47 mm, external diameter 1.96 mm, 508‐006; Dow Corning, USA) with 10 mg of DHT in accordance with the methods in previous studies,^[^ ^47^ ^]^ and were remained in mice for the entire duration of the experiment without replacement. Blank implants were implanted into the neck subcutaneous tissues of the mice in the control group. Subsequently, each group of mice was divided into two subgroups (n = 30, per group). Two groups were implanted with blank or DHT implants drinking water daily. The other two groups were embedded with blank or DHT implants and were given daily water containing metformin at a concentration designed to deliver a dose of 500 mg kg^−1^ body weight day^−1^. The body weights of the mice were recorded every week. Vaginal smears were examined daily for 2 weeks beginning at 13 weeks of age. Insulin tolerance tests (ITTs) and oral glucose tolerance tests (oGTTs) were performed at the age of 15 and 16 weeks, individually, in line with the methods in previous studies.^[^ ^47^ ^]^ At 16 weeks of age, ten mice from each group were sacrificed at the indicated time by CO_2_ asphyxia. The blood samples and tissues were collected. Serum was further collected immediately after centrifugation at 16 000 × g for 10 min at 4 °C. Serum total testosterone (TT), 17β‐estradiol (E2), progesterone (P4), triglycerides (TG), cholesterol (CHOL), high‐density lipoprotein (HDL), and low‐density lipoprotein (LDL) levels were analyzed with a UniCel DxI 800 Access Immunoassay System (Beckman Coulter, USA) in a clinical laboratory. Tissue samples were collected, snap‐frozen in liquid nitrogen, and stored at −80 °C. Formalin‐fixed ovary samples were processed, and 5‐µm‐thick paraffin sections were stained with hematoxylin and eosin for histological analysis. Histological images were captured with an Axio Scope A1 (Zeiss, Germany). A portion of mice (n = 14, per group) were mating at 1:1 ratio (male: female) overnight after injection with pregnant mare serum gonadotrophin (PMSG; 5 IU per 10 g of body weight) for 44 h, followed by injecting with human chorionic gonadotropin (hCG; 5 IU per 10 g of body weight) for 16 h to induce production of metaphase II (MII) oocytes. The PMSG and hCG were purchased from Ningbo Sansheng Pharmaceutical Co. (Ningbo, China). In the morning of the next day, couple were separated and copulation was verified by the presence of a vaginal plug, and this was considered embryonic day (E) 0.5. The mice were sacrificed between 8:00 and 9:00 h on E11.5. Evaluation of the fetal outcome included the numbers of implantation sites, embryo numbers and resorptions, the placental weights. The numbers of viable fetuses and resorbed fetuses were recorded visually. Viable fetuses were a healthy pink color, whereas non‐viable (dead and resorbed) fetuses were usually small, haemorrhagic, and/or emaciated and pale. Only viable placentas and fetuses were analyzed further. The rest of mice (n = 6, per group) were continuously mating at 1:2 ratio (male: female) for long‐term fertility experiments. The offspring appearance, numbers, and the offspring sex ratio were recorded visually.

### 2 Gestational Acute DHT Exposure Model

Because treatment with testosterone propionate or DHT retards the transport of the embryo in the fallopian tubes (E2.5−E5.5) and results in an aberrant preimplantation process and subsequently delays on‐time implantation in rodents, we injected pregnant mice with DHT from E5.5 (the time of completion of implantation) to establish an acute DHT exposure mouse model. Pregnant mice at 6 weeks of age were randomly divided into two groups at E5.5 and treated with sesame oil as controls (n = 18) or DHT (1.66 mg kg^−1^ day^−1^, suspended in sesame oil) subcutaneous injection for six consecutive days (n = 18). In each group, six dams were given daily water, six dams were given flutamide subcutaneous injection with 50 mg kg^−1^ daily from E5.5, and six dams were given daily water containing metformin with 500 mg kg^−1^ daily from E5.5. All mice were sacrificed between 08.00 and 09.00 h on E11.5. Uterus and placentas were dissected as rapidly as possible on a single animal. Harvested fresh tissues were weighed and fixed in 4% formaldehyde and neutral buffered solution or immediately frozen in liquid nitrogen and stored at −80 °C. The numbers of viable and resorbed fetuses were recorded visually.

### Follicle Count

Ovaries were collected and fixed in 10% buffered formalin, embedded in paraffin and cut into serial sections (5 µm). The sections were then stained with hematoxylin and eosin. To evaluate follicular development in mice, all follicles with a visible nucleus were counted every fifth section as previously described.^[^ ^76^ ^]^ In brief, primordial follicles were surrounded by a flat layer of granulosa cells, while primary follicles contained a layer of cuboidal granulosa cells. Multiple layers of granulosa cells surrounding follicles were determined to be in the secondary stage, and follicles with a visible cavity between oocytes and granulosa cells were considered to be in the antral stage. Atretic follicles contained degenerating oocytes, disorganized granulosa cells or apoptotic bodies.^[^ ^77^ ^]^ All sections were counted by two independent individuals for comparison.

### Study Approval

This study was initiated on 1 February 2023 and terminated on 31 January 2025. Studies utilizing human villus tissues were approved by the Ethics Committee of Sir Run Run Shaw Hospital affiliated to the School of Medicine, Zhejiang University (Approval No. 20 230 202). All participants provided written informed consent to participate in this study. All animal studies were performed in accordance with ethical regulations and protocols approved by the Sir Run Run Shaw Hospital Committee for Animal Resources and the Institutional Animal Care and Use Committee of Zhejiang Center of Laboratory Animals (Approval No. ZJU20240063). All mouse experimental procedures were performed following the Regulations for the Administration of Affairs Concerning Experimental Animals approved by the State Council of People's Republic of China.

### Quantification and Statistical Analysis

GraphPad Prism software was used for statistical analysis. Statistical comparisons between two groups were carried out using unpaired Student's *t*‐test after confirming the normal distribution of the data by one‐sample Kolmogorov‐Smirnov test for Gaussian distribution or using Mann‐Whitney *U*‐test for comparisons of data that did not show Gaussian distribution. The comparisons of more than two groups were carried out by one‐way analysis of variance (ANOVA) with Tukey's multiple comparison test or Sidak's multiple comparison test. All the data are shown as the mean values ± standard error of the means (SEMs) and *p*‐values of < 0.05 were considered significant. Statistical significance was determined as indicated in the figure legends: ns, not significant, *p < 0.05, **p < 0.01, ***p < 0.001, ****p < 0.0001.

## Conflict of Interest

The authors declare no conflict of interest.

## Author Contributions

H.Z. and W.Y. contributed equally to this work and shared first authorship. H.Z. designed and performed the experiments. N.L., Q.L., and X.J. collected the clinical samples. H.Z., W.Y., J.J., and C.Z. conducted the PCOS model mice. H.Z. and W.Y. analyzed the data. M.G. analyzed protein structure of AR and performed molecular docking. H.Z. made the figures and drafted the article. Y.Z., S.Z., and H.Z. critically reviewed the article. Y.Z and S.Z. conceived and supervised the project.

## Supporting information



Supporting Information

Supporting Information

Supporting Information

Supporting Information

Supporting Information

Supporting Information

Supporting Information

## Data Availability

The data that support the findings of this study are available in the supplementary material of this article.
