# Supplementary material for: Hyperandrogenemia Induces Trophoblast Ferroptosis and Early Pregnancy Loss in Patients With PCOS via CMA‐Dependent FTH1 Degradation
Source: Adv Sci (Weinh). 2025 Dec 16;13(12):e06091. doi: 10.1002/advs.202506091 (PMC12948281; doi:10.1002/advs.202506091)
Supplement: Supplementary file 1 — Supporting Information [file ADVS-13-e06091-s006.docx]

**Supplemental information**

1. **Supplemental figures**


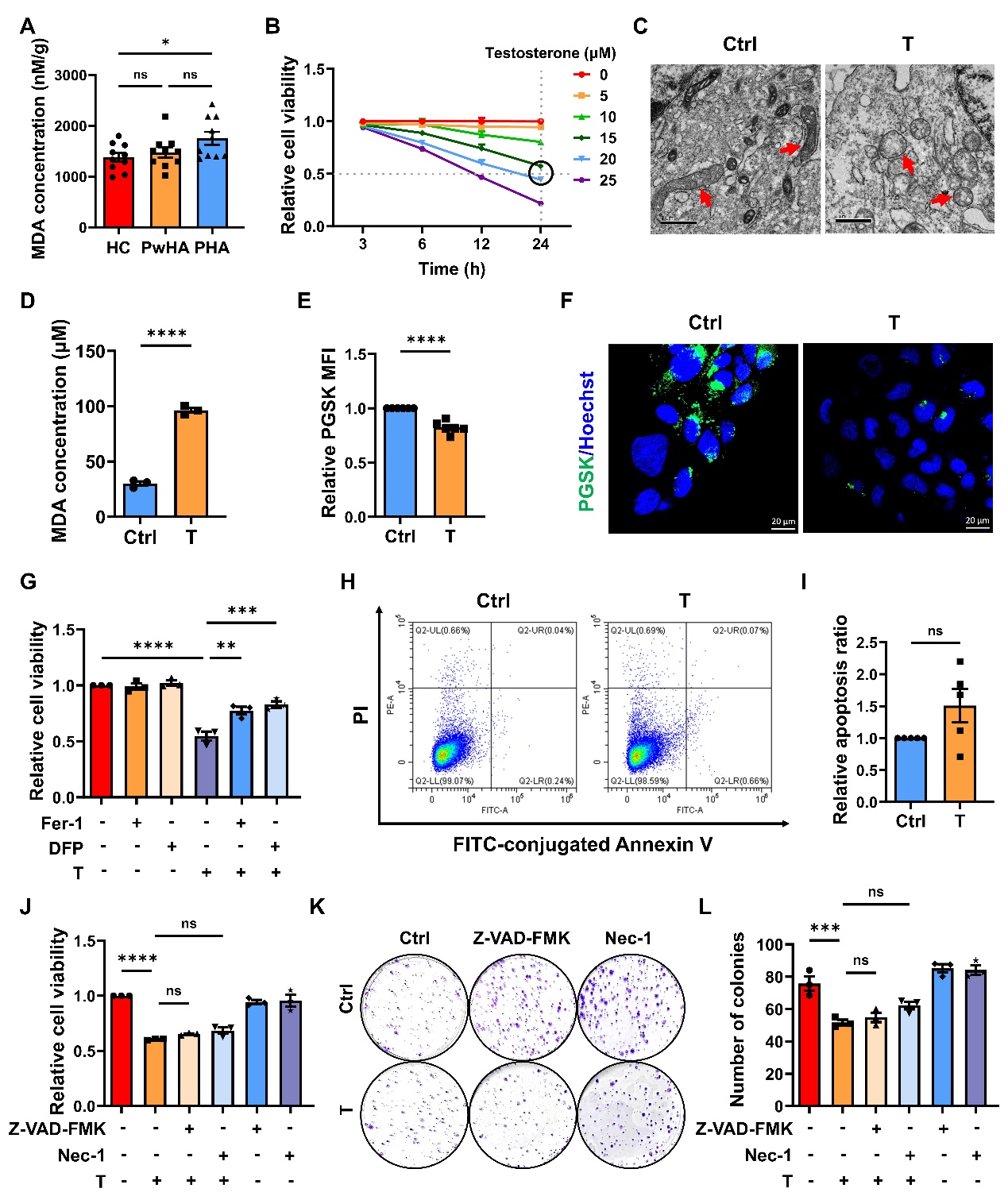


**Figure S1. Androgen treatment induces ferroptosis in villi and TS^CT^ cells.** (**A**) MDA levels of villi collected between the 6^th^ and 8^th^ weeks from the HC, PwHA, and PHA groups. Ten villus explants from ten individuals in each group were analyzed. (**B**) Analysis of the viability of TS^CT^ cells treated with 5 μM, 10 μM, 15 μM, 20 μM, or 25 μM T for different durations. The results of six independent experiments were analyzed. (**C**) Representative TEM images of TS^CT^ cells with or without T treatment from three independent experiments. Red arrows indicated mitochondria. Scale bars, 1 µm. (**D**) Intracellular MDA levels in TS^CT^ cells with or without T treatment for 24 h. Three independent experiment results were analyzed. (**E**) Flow cytometry analysis showing the relative mean fluorescence intensity of PGSK in TS^CT^ cells in the T-treated group compared with that in the control group. The results of six independent experiments were analyzed. (**F**) Representative immunofluorescence images of PGSK staining in control and T-treated groups. Hoechst 33342 indicated the nucleus. Scale bars, 20 µm. (**G**) Analysis of the viability of TS^CT^ cells pretreated with Fer-1 or DFP for 24 h, followed by T treatment for another 24 h. The results of three independent experiments were analyzed. (**H**) Flow cytometry results showing the apoptotic ratio in control and T-treated TS^CT^ cells. LL quadrant: Annexin V (–), PI (–) represents living cells; LR quadrant: Annexin V (+), PI (–) represents early apoptotic cells; UR quadrant: Annexin V (+), PI (+) represents late apoptotic cells; UL quadrant: Annexin V (–), PI (+) represents mechanically necrotic cells. Apoptotic cells include early apoptotic cells and late apoptotic cells. (**I**) The relative apoptosis ratio in the T-treated group compared with that in the control group. The results of five independent experiments were analyzed. (**J**) Viability of TS^CT^ cells pretreated with Z-VAD-FMK or Nec-1 for 24 h, followed by T treatment for another 24 h. The results of three independent experiments were analyzed. (**K**) Representative images of cell colony formation in each group. (**L**) The number of colonies in each group was determined. The results of three independent experiments were analyzed.

The data are presented as the mean ± SEM. Statistical analysis was performed using one-way ANOVA in (**A**), (**G**), (**J**) and (**L**) and unpaired *t* tests in (**D**), (**E**) and (**I**). ns, not significant; **P* < 0.05, ***P* < 0.01, ****P* < 0.001, *****P* < 0.0001. HC, healthy control; PwHA, PCOS without hyperandrogenemia; PHA, PCOS with hyperandrogenemia; TEM, transmission electron microscopy; MDA, malonaldehyde; PGSK, Phen Green™ SK; MFI, mean fluorescence intensity; Fer-1, ferrostatin-1; DFP, deferiprone; T, testosterone; PI, propidium iodide; Nec-1, necrostatin-1. T: 20 μM; Fer-1: 1 μM; DFP: 100 μM; Z-VAD-FMK: 5 μM; Nec-1: 10 mM.

^
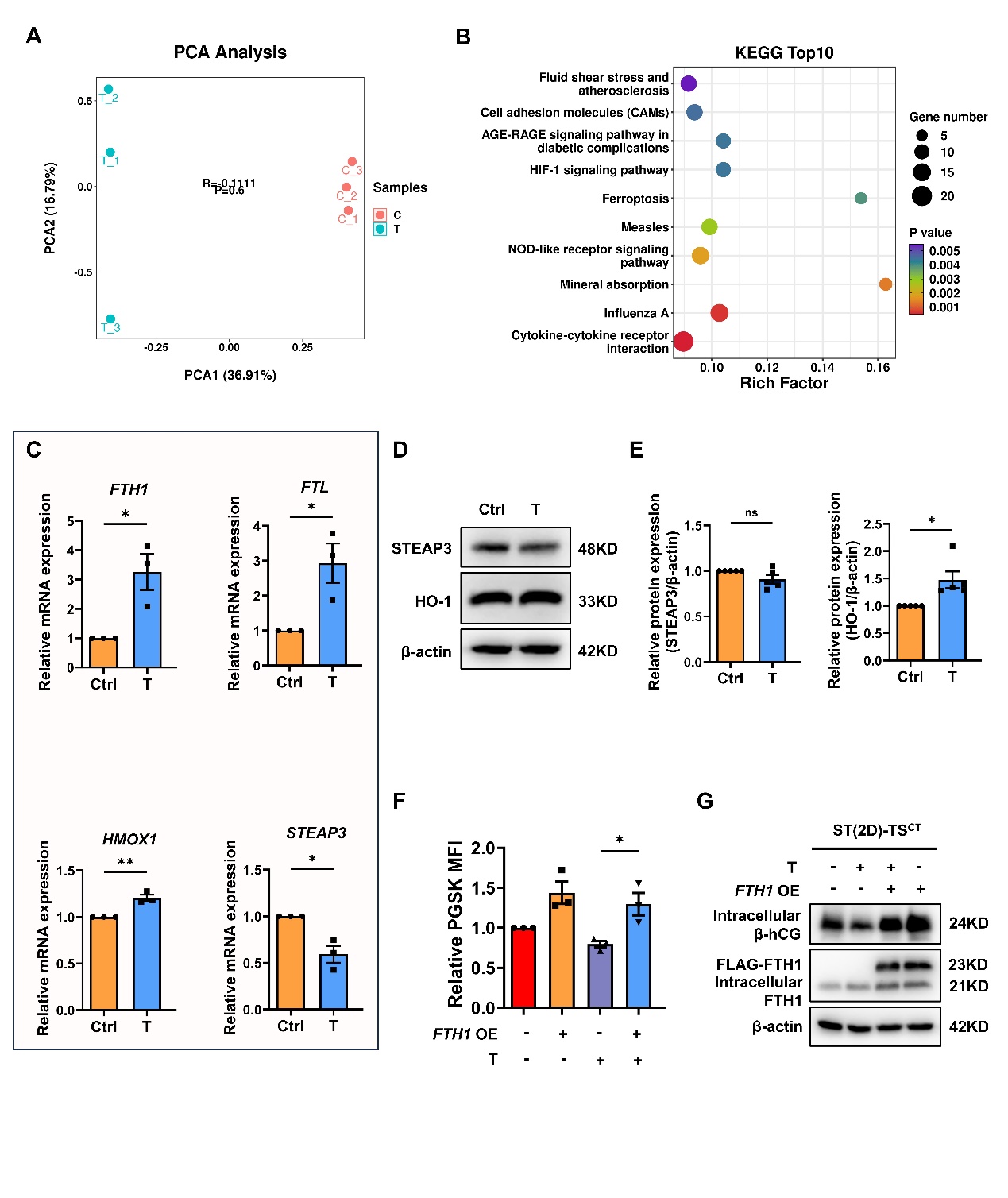
^

**Figure S2. Effects of androgen treatment on the transcriptome and ferroptosis-related gene expression.** (**A**) PCA plot showing the distinct clusters of TS^CT^ cells in the control and T-treated groups (*R* = -0.1111 and *P =* 0.6). (**B**) The top 10 enriched KEGG pathways in TS^CT^ cells under T conditions are highlighted. (**C**) RT–qPCR analysis of *FTH1*, *FTL*, *HMOX1*, and *STEAP3* gene expression. n = 3 biological replicates were included in each group. (**D**) Western blot analysis showing the STEAP3 and HO-1 protein levels in TS^CT^ cells treated with T for 24 h. (**E**) Statistical quantification of STEAP3 and HO-1 protein levels in each group, normalized to that of β-actin. The results of five independent experiments were analyzed. (**F**) Flow cytometry analysis shows the relative mean fluorescence intensity of PGSK in TS^CT^ cells transfected with FLAG-FTH1 plasmids for 24 h, followed by T treatment for another 24 h. The results of three independent experiments were analyzed. (**G**) Western blot analysis showing secreted and intracellular β-hCG and FTH1 protein levels in ST(2D)-TS^CT^ cells transfected with FLAG-FTH1 plasmids for 24 h, followed by T treatment for another 24 h. n = 3 independent experiments were performed.

The data are presented as the mean ± SEM. Statistical analysis was performed using unpaired *t*-test in (**C**) and (**E**) and one-way ANOVA in (**F**). ns, not significant; **P* < 0.05, ***P* < 0.01. PCA, principal component analysis; KEGG, Kyoto Encyclopedia of Genes and Genomes; T, testosterone; MFI, mean fluorescence intensity; OE, overexpression; ST, syncytiotrophoblast. T: 20 μM.

^
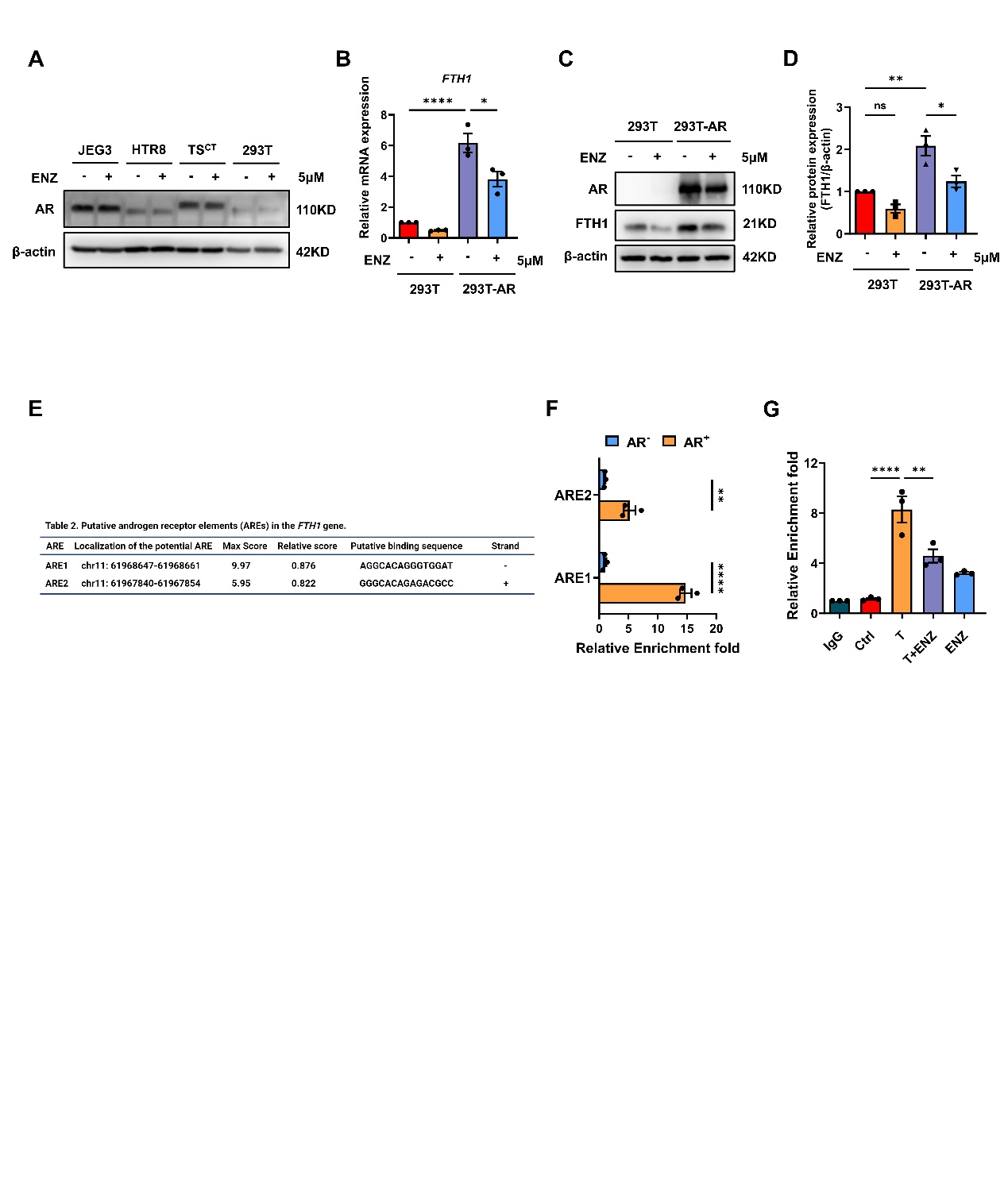
^

**Figure S3. Androgen receptor (AR) directly binds to the promoter of FTH1 to promote its mRNA expression.** (**A**) Western blot analysis showing endogenous AR expression in a panel of AR-high-expressing trophoblasts (JEG3 and TS^CT^ cells) and AR-low-expressing cells (HTR8 and HEK293T cells). Cells were treated with or without ENZ for 24 h. n = 3 independent experiments were performed. (**B**) RT–qPCR analysis showing *FTH1* expression in control and *AR*-overexpressing HEK293T cells with or without ENZ treatment for 24 h. n = 3 biological replicates were included in each group. (**C**) Western blot analysis showing AR and FTH1 expression in control and *AR*-overexpressing HEK293T cells with or without ENZ treatment for 24 h. (**D**) Statistical quantification of the FTH1 protein levels in each group normalized to those of β-actin. The results of three independent experiments were analyzed. (**E**) Prediction of two AR binding sites in the human *FTH1* promoter region. (**F**) RT–qPCR analysis followed by a CUT&Tag assay revealed that both ARE1 and ARE2 were AR binding sites on the *FTH1* promoter in *AR*-overexpressing HEK293T cells. n = 3 biological replicates were included in each group. (**G**) CUT&Tag‒qPCR results showing the occupancy of AR on the human *FTH1* ARE1 region in TS^CT^ cells treated with T and/or ENZ for 24 h. n = 3 biological replicates were included in each group.

The data are presented as the mean ± SEM. Statistical analysis was performed using one-way ANOVA in (**B**), (**D**), (**F**) and (**G**). ns, not significant; **P* < 0.05, ***P* < 0.01, *****P* < 0.0001. AR, androgen receptor; ARE, androgen response element; ENZ, enzalutamide; T, testosterone. T: 20 μM; ENZ: 5 μM.

^
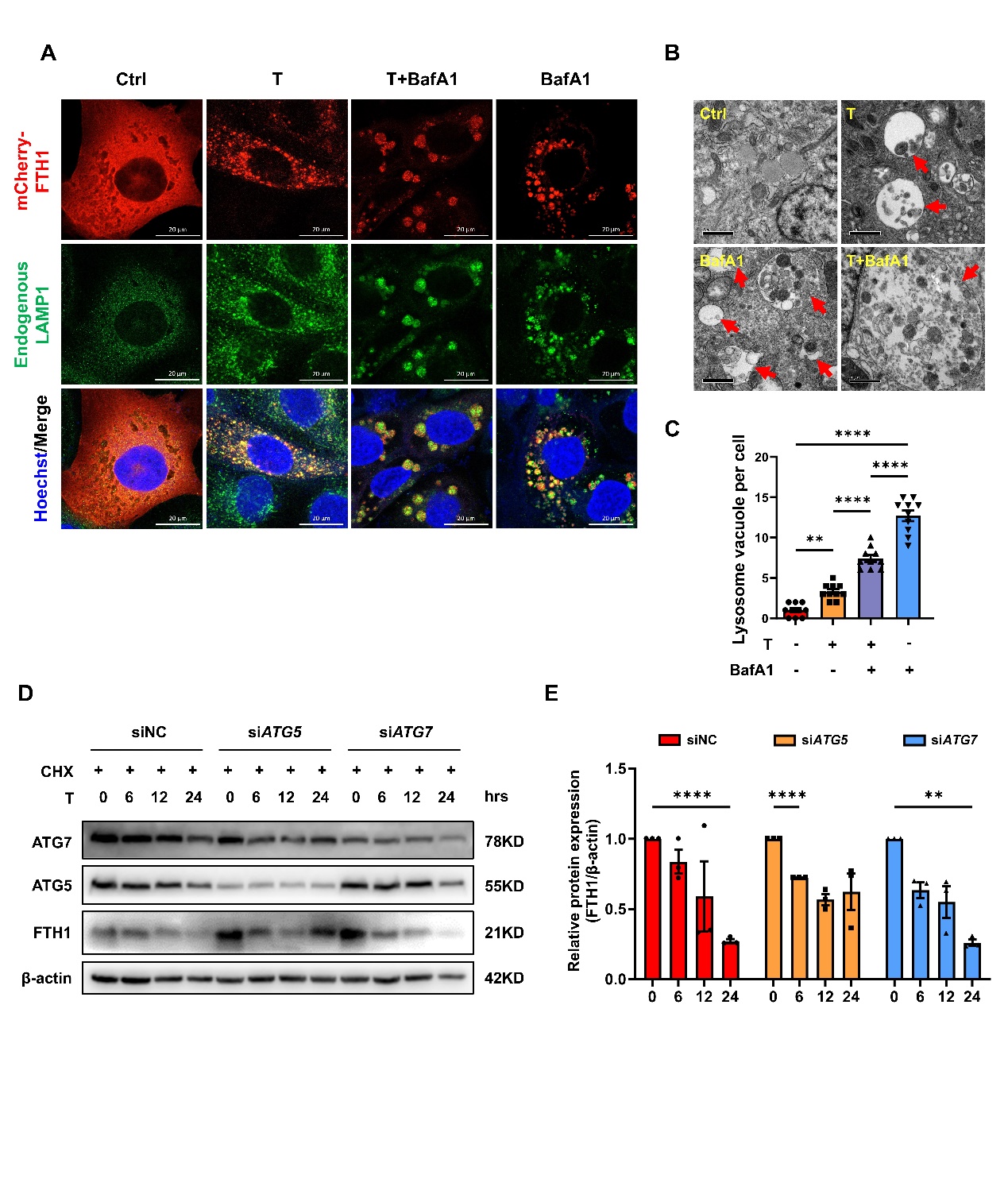
^

**Figure S4. The degradation of FTH1 is independent of the macroautophagy-lysosome pathway.** (**A**) Representative immunofluorescence images showing colocalization of endogenous LAMP1 (green) and Hoechst 33342 (blue) in mCherry-FTH1 (red)-overexpressed TS^CT^ cells with or without T and/or BafA1 treatment for 24 h. Scale bars, 20 µm. n = 3 independent experiments were performed. (**B**) Representative TEM images of TS^CT^ cells with or without T and/or BafA1 treatment for 24 h. Red arrows indicated lysosomes. Scale bars, 1 µm. n = 3 independent experiments were performed. (**C**) Statistical quantification of the number of lysosomal vacuoles per cell. Ten cells were included in each group. (**D**) Western blot analysis showed ATG5, ATG7 and FTH1 protein levels in CHX-pretreated TS^CT^ cells with si*ATG5* or si*ATG7* interference for 48 h, followed by T treatment for 6 h, 12 h or 24 h. (**E**) Statistical quantification of ATG5, ATG7 and FTH1 protein levels in each group normalized to that of β-actin. The results of three independent experiments were analyzed.

The data are presented as the mean ± SEM. Statistical analysis was performed using one-way ANOVA in (**C**) and (**E**). ***P* < 0.01, *****P* < 0.0001. TEM, transmission electron microscopy; T, testosterone; BafA1, bafilomycin; CHX, cycloheximide. T: 20 μM; BafA1: 100 nM; CHX: 50 μg/mL.

^
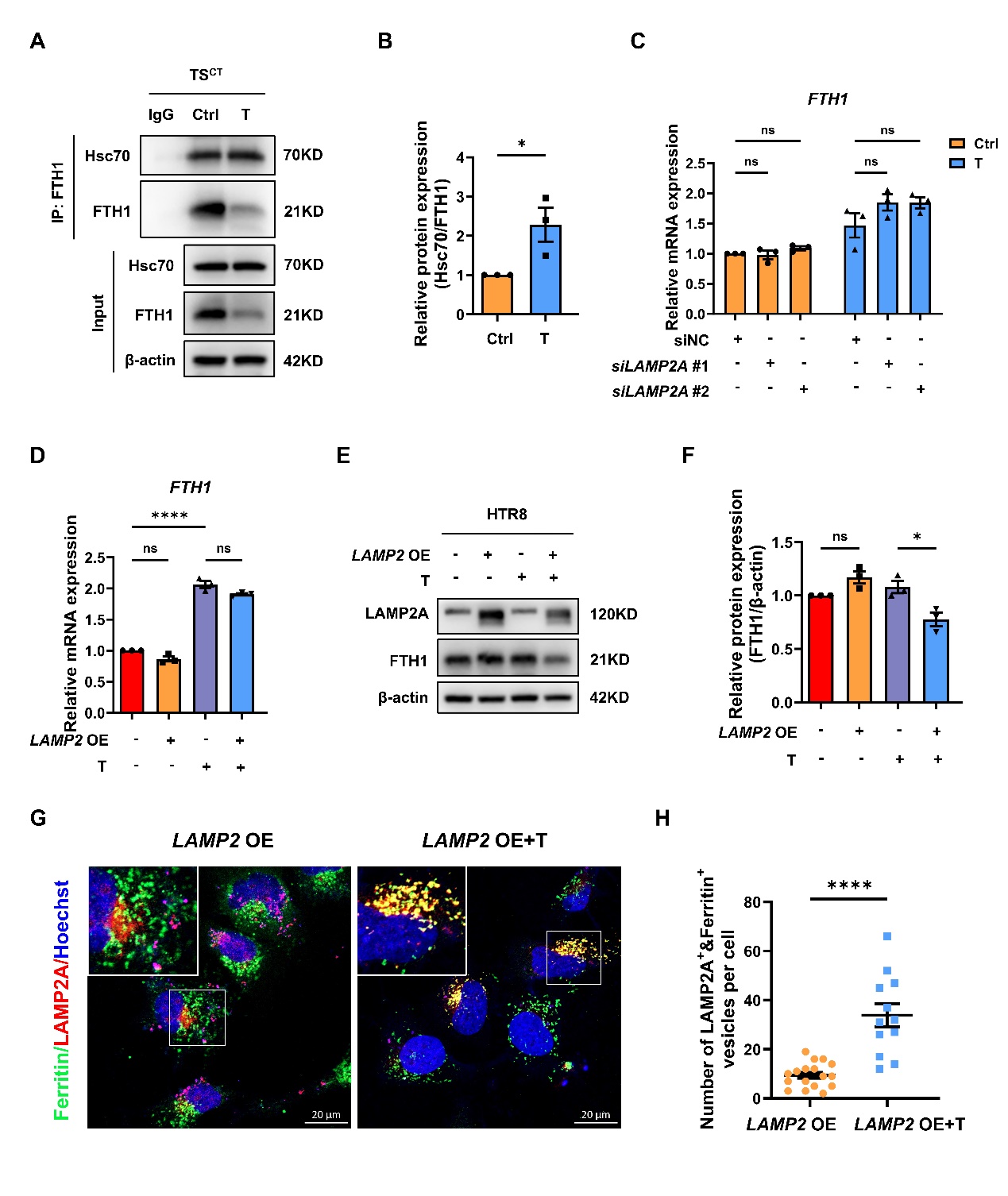
^

**Figure S5. Modulation of CMA influences the FTH1 protein level.** (**A**) TS^CT^ cells were treated with or without T for 24 h. The binding of endogenous FTH1 and endogenous Hsc70 was confirmed by co-IP. (**B**) Statistical quantification of Hsc70 protein levels in control and T-treated groups normalized to that of FTH1. The results of three independent experiments were analyzed. (**C**) RT–qPCR analysis of *FTH1* mRNA expression in control and *LAMP2A* knockdown (two different sequences #1 and #2) TS^CT^ cells treated with or without T for 24 h. n = 3 biological replicates were included in each group. (**D**) RT–qPCR analysis of *FTH1* mRNA expression in HTR8 cells transfected with *LAMP2* plasmids for 48 h, followed by treatment with or without T for another 24 h. n = 3 biological replicates were included in each group. (**E**) Western blotting showing LAMP2A and FTH1 protein levels in HTR8 cells transfected with *LAMP2* plasmids for 48 h, followed by treatment with or without T for another 24 h. (**F**) Statistical quantification of the FTH1 protein levels in each group normalized to those of β-actin. The results of three independent experiments were analyzed. (**G**) Representative immunofluorescence images showing colocalization of endogenous ferritin (green) and mCherry-LAMP2A (red) in *LAMP2*-overexpressing TS^CT^ cells with or without T treatment for 24 h. Hoechst 33342 indicated the nucleus. Scale bars, 20 µm. n = 3 independent experiments were performed. (**H**) Statistical quantification of both LAMP2A- and ferritin-positive vesicles per cell was performed. Seventeen cells were counted in the *LAMP2*-overexpression group, and twelve cells were included in the *LAMP2*-overexpression with T treatment group.

The data are presented as the mean ± SEM. Statistical analysis was performed using an unpaired *t* test in (**B**) and (**H**) and one-way ANOVA in (**C**), (**D**) and (**F**). ns, not significant; **P* < 0.05, *****P* < 0.0001. T, testosterone; IP, immunoprecipitation; OE, overexpression. T: 20 μM.

^
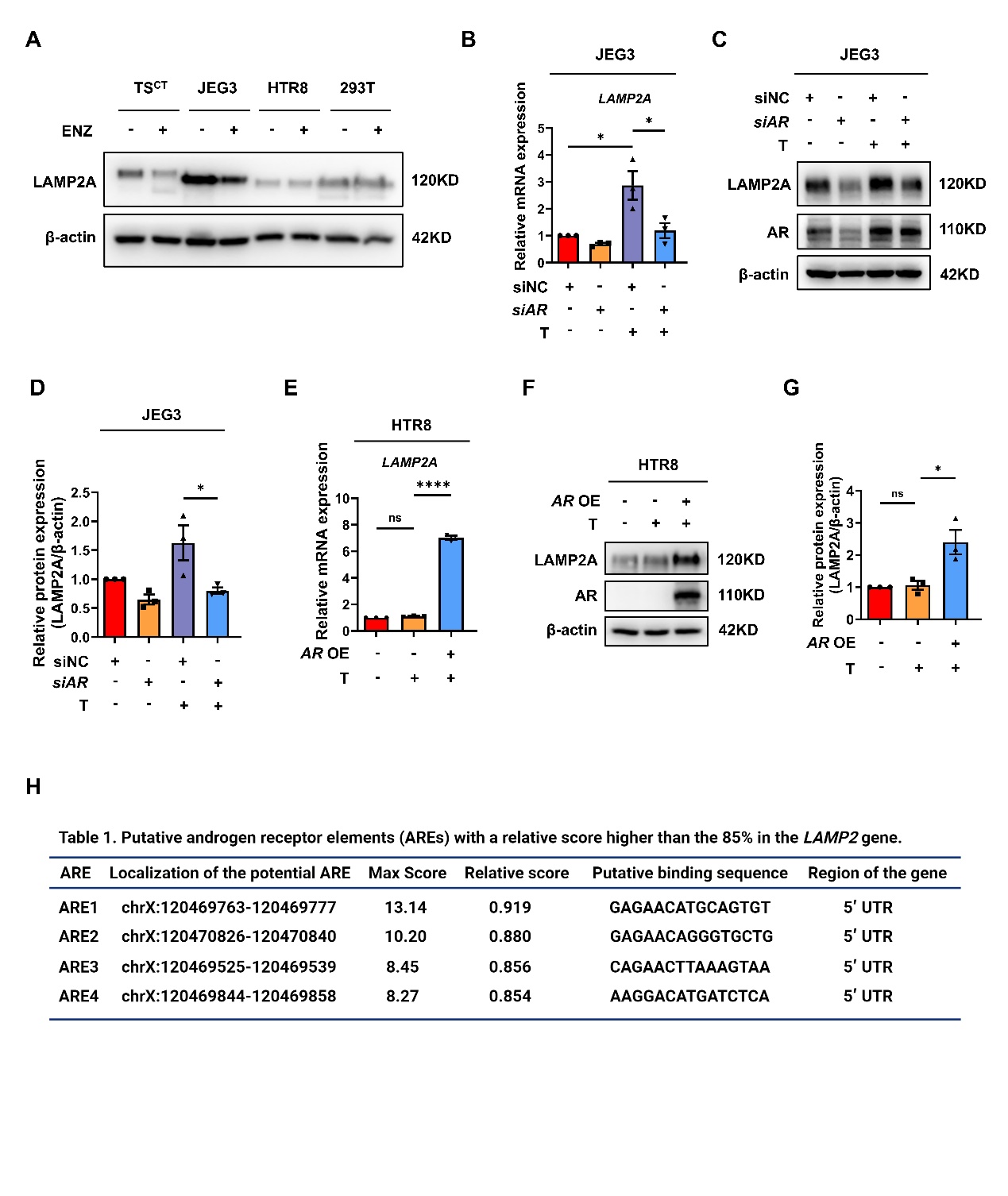
^

**Figure S6. Androgen-AR signaling upregulates LAMP2A expression in trophoblasts.** (**A**) Western blot analysis showing endogenous LAMP2A expression in a panel of AR high-expressing trophoblasts (JEG3 and TS^CT^ cells) and AR low-expressing cell lines (HTR8 and HEK293T). Cells were treated with DMSO or ENZ for 24 h. n = 4 independent experiments were performed. (**B**) RT–qPCR analysis of *LAMP2A* expression in JEG3-siNC and JEG3-si*AR* cells with or without T treatment for 24 h. n = 3 biological replicates were included in each group. (**C**) Western blot analysis showing LAMP2A and AR expression in JEG3-siNC and JEG3-siAR cells with or without T treatment for 24 h. (**D**) Statistical quantification of LAMP2A protein levels in each group normalized to that of β-actin. The results of three independent experiments were analyzed. (**E**) RT–qPCR analysis of *LAMP2A* expression in control and T-treated HTR8 cells with or without *AR* overexpression. n = 3 biological replicates were included in each group. (**F**) Western blot analysis showing AR and LAMP2A expression in control and T-treated HTR8 cells with or without *AR* overexpression. (**G**) Statistical quantification of LAMP2A protein levels in each group normalized to that of β-actin. The results of three independent experiments were analyzed. (**H**) Prediction of four AREs in the human *LAMP2* promoter regions.

The data are presented as the mean ± SEM. Statistical analysis was performed using one-way ANOVA in (**B**), (**D**), (**E**) and (**G**). not significant; **P* < 0.05, *****P* < 0.0001. AR, androgen receptor; ARE, androgen response element; ENZ, enzalutamide; T, testosterone; siNC, negative control siRNA; OE, overexpression. T: 20 μM; ENZ: 5 μM.

^
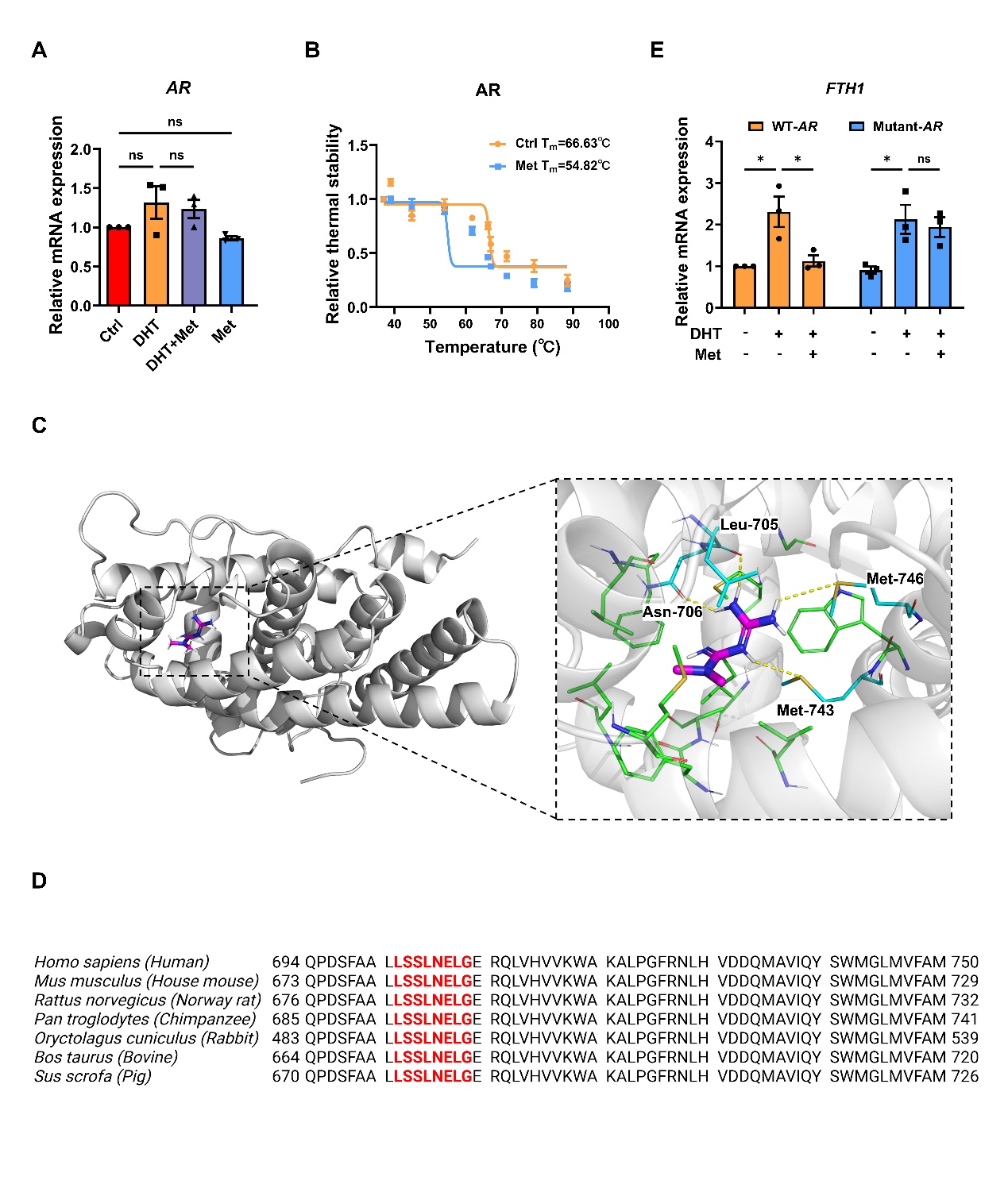
^

**Figure S7.** **Metformin binds to and stabilizes the AR protein rather than downregulating *AR* mRNA expression.** (**A**) RT–qPCR analysis showing *AR* mRNA expression in TS^CT^ cells pretreated with or without Met for 12 h, followed by DHT incubation for another 12 h. n = 3 biological replicates were included in each group. (**B**) The thermal stability of AR was evaluated after treatment with 5 mM Met for 30 min. The results of three independent experiments were analyzed. (**C**) Predicted amino acid sites of the AR protein that interact with Met through H-bonds (Leu705, Asn706, Met743, and Met746). (**D**) Sequence alignment of the core binding site of AR to which metformin binds across different species. (**E**) RT–qPCR analysis of *FTH1* mRNA expression in HEK293T cells transfected with WT or mutant AR (deletion of 702–709 aa) plasmids and then incubated with or without Met for 12 h, followed by DHT treatment for another 12 h. n = 3 biological replicates were included in each group.

The data are presented as the mean ± SEM. Statistical analysis was performed using one-way ANOVA in (**A**) and (**E**), and a standard curve was interpolated with sigmoidal, 4PL, where X is the concentration model in (**B**). ns, not significant; **P* < 0.05. DHT, dihydrotestosterone; Met, metformin; Del, deletion. DHT: 100 nM; Met: 5 mM.

^
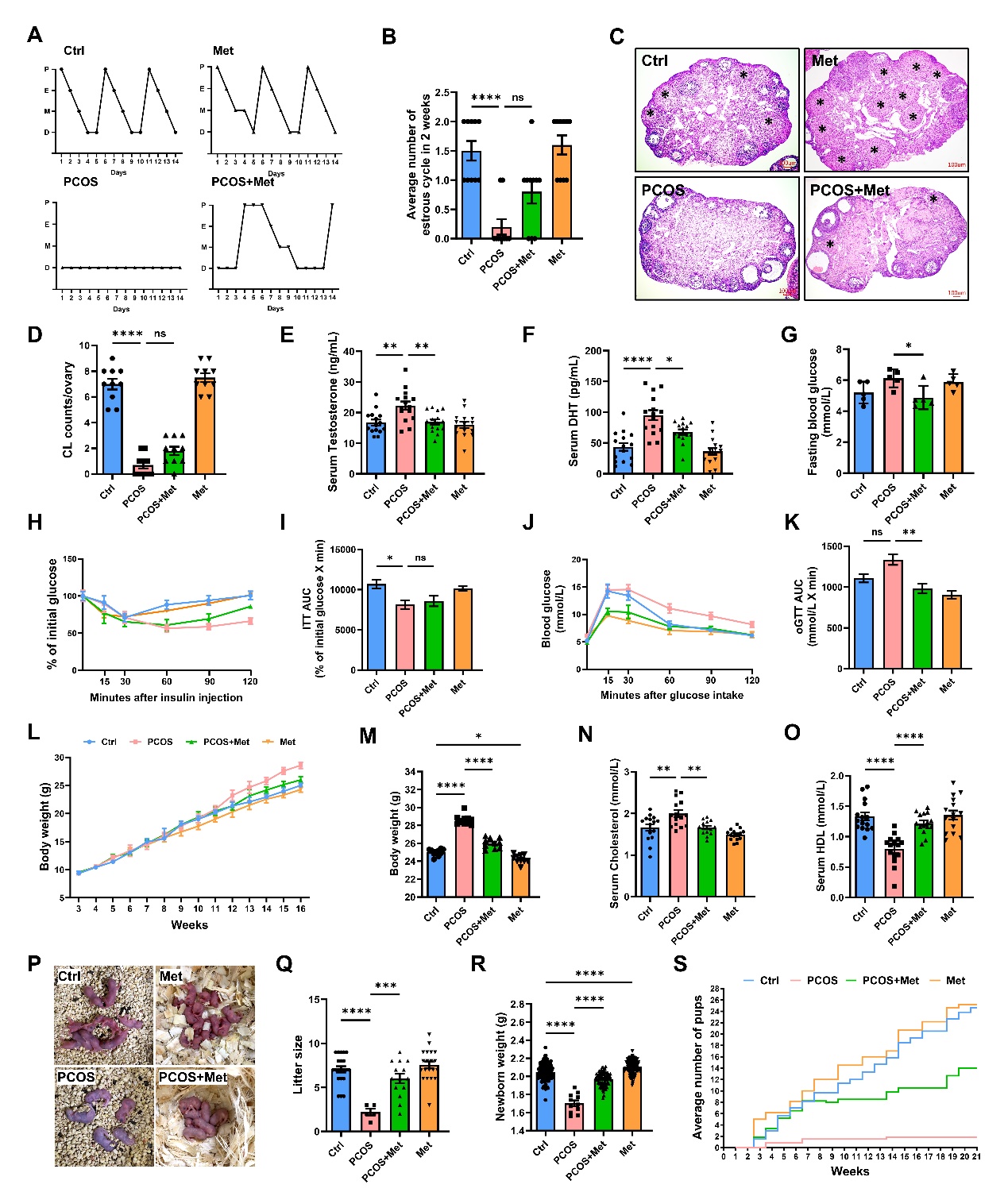
^

**Figure S8. Reproductive and metabolic indicators of mice in the control, metformin, DHT, and DHT and metformin groups.** (**A**) Estrous cycle patterns of representative mice from the control, Met, PCOS, and PCOS + Met groups. (**B**) Average number of estrous cycles at 2 weeks in each group. n = 10 per group. (**C**) Representative images of hematoxylin–eosin-stained ovaries from each group. The red stars indicate CLs. Scale bar, 100 μm. n = 10 per group. (**D**) Numbers of CLs per ovary at 13 weeks of age in each group. n = 10 per group. (**E-F**) Measurement of serum T (**E**) and DHT (**F**) levels in different groups at 16 weeks of age. n = 15 per group. (**G**) Fasting blood glucose levels in different groups at 15 weeks of age. n = 5 per group. (**H-K**) ITT (**H**) and oGTT (**J**) results at 15 weeks of age. AUC analysis of the ITT (**I**) and oGTT (**K**) results. n = 5 per group. (**L**) The body weights of the mice in each group were recorded every week from 3 to 16 weeks of age. n = 10 per group. (**M**) Body weights of the mice in each group at 13 weeks of age. n = 10 per group. (**N-O**) Measurement of serum CHOL (**N**) and HDL levels (**O**) in different groups at 16 weeks of age. n = 15 per group. (**P**) Representative morphological images of offspring from different groups. 6 labors per group. (**Q**) The number of offspring per litter in each group. n = 21 litters in Ctrl group, n = 5 litters in PCOS group, n = 14 litters in PCOS + Met group, n = 20 litters in Met group. (**R**) Newborn weights in each group. n = 148 newborns in Ctrl group, n = 11 newborns in PCOS group, n = 84 newborns in PCOS + Met group, n = 151 newborns in Met group. (**S**) Fertility curves showing the average number of offspring (total offspring/number of labors) every week in each group.

The data are presented as the mean ± SEM. Statistical analysis was performed using one‐way ANOVA in (**B**), (**D**) to (**G**), (**I**), (**K**), (**M**) to (**O**), (**Q**) and (**R**). ns, not significant; **P* < 0.05, ***P* < 0.01, ****P* < 0.001, *****P* < 0.0001. CL, corpora lutea; P, pro-estrus; E, estrus; M, metestrus; D, diestrus; T, testosterone; DHT, dihydrotestosterone; Met, metformin; CHOL, cholesterol; HDL, high-density lipoprotein; ITT, insulin tolerance test; oGTT, oral glucose tolerance test; AUC, area under the curve.

^
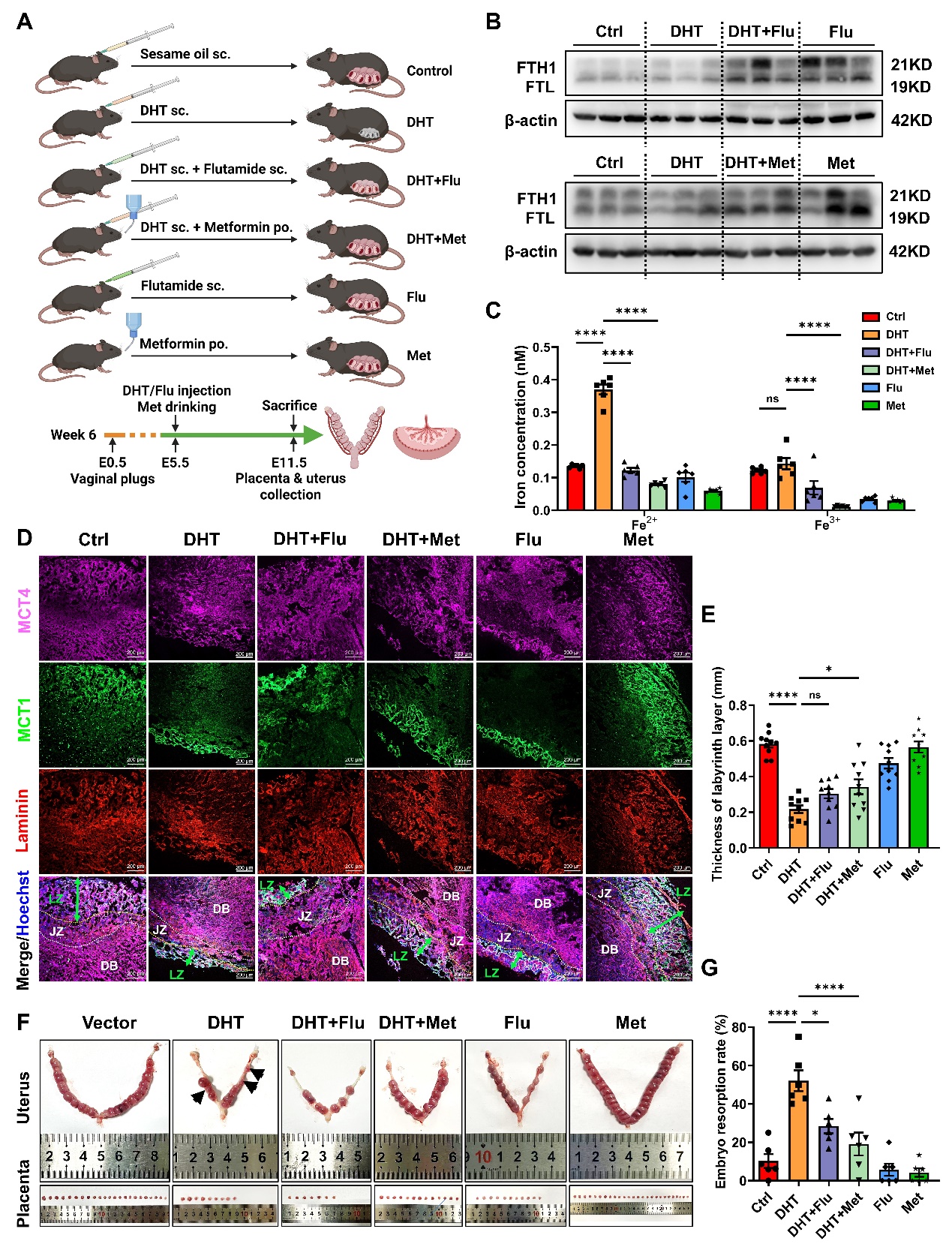
^

**Figure S9. AR antagonism ameliorates placental development and fetal loss in acute DHT-exposed mice during early gestation.** (**A**) Schematic depiction of the acute DHT exposure experiment. (**B**) Representative Western blot analysis of FTH1 and FTL expression in E11.5 placentas from the control, DHT, Flu, Met, DHT + Flu, and DHT + Met groups. n = 3 placentas were included in each group. (**C**) Measurement of iron concentrations in viable placentas at E11.5 in different groups. n = 6 placentas were included in each group. (**D**) Representative immunofluorescence images of placentas from different groups stained for SynT I and SynT II with MCT1, MCT4, and laminin. Hoechst 33342 indicated the nucleus. The white and yellow dotted lines indicated the boundaries of the DB/JZ and JZ/LZ layers, respectively. The green double-sided arrows indicated the thickness of the labyrinth layer. Scale bars, 200 μm. (**E**) The thickness of the labyrinth layer of the placenta at E11.5 was measured. n = 10 placentas were included in each group. (**F**) Representative morphological images of the uteri and placentas in mice from different groups at E11.5. The black arrows indicated the absorbed embryos. (**G**) Embryo resorption rate in each group at E11.5. n = 6 per group.

The data are presented as the mean ± SEM. Statistical analysis was performed using one‐way ANOVA in (**C**), (**E**) and (**G**). ns, not significant; **P* < 0.05, *****P* < 0.0001. LZ, labyrinth zone; JZ, junctional zone; DB, decidua basal zone; SynT I, syncytiotrophoblast I; SynT II, syncytiotrophoblast II; DHT, dihydrotestosterone; Flu, flutamide; Met, metformin.

1. **Supplemental tables**

**Table S1. Treat vs. Control gene differential expression.**

**Table S2. Treat vs. Control KEGG enrichment gene.**

**Table S3. Tandem mass spectrometry.**

**Table S4. Energy decomposition analysis.**

**Table S9. Genes FPKM expression.**

**Table S1-S4 and Table S9 were presented in .xlsx format in Data File.**

**Table S5.** **Effects of continuous treatment with DHT and/or metformin on the endocrine and metabolic alterations in C57BL/6 mice.**

|  | Control | PCOS | PCOS + Met | Met |
| --- | --- | --- | --- | --- |
| BW (g) | 25.77 ± 0.12 | 28.59 ± 0.18^a^ | 25.99 ± 0.18 | 24.28 ± 0.15 |
| E2 (pmol/L) | 17.61 ± 4.79 | 20.16 ± 4.67^a^ | 18.99 ± 5.59 | 18.47 ± 6.69 |
| P4 (ng/mL) | 4.45 ± 1.57 | 2.18 ±1.10^a^ | 3.89 ± 1.65^b^ | 4.89 ± 1.80 |
| TT (ng/mL) | 10.28 ± 2.57 | 8.07 ± 2.13^a^ | 9.95 ± 4.56^b^ | 9.86 ± 2.81 |
| DHT (pg/mL) | 43.55 ± 6.35 | 95.18 ± 8.10^a^ | 67.66 ± 4.41^b^ | 36.50 ± 5.55 |
| TG (mmol/L) | 0.85 ± 0.21 | 0.82 ± 0.20 | 0.83 ± 0.23 | 0.81 ± 0.16 |
| CHOL (mmol/L) | 1.66 ± 0.08 | 2.00 ± 0.08^a^ | 1.66 ± 0.04^b^ | 1.49 ± 0.03 |
| HDL (mmol/L) | 1.33 ± 0.06 | 0.80 ± 0.06^a^ | 1.22 ± 0.04^b^ | 1.36 ± 0.07 |
| LDL (mmol/L) | 0.36 ± 0.07 | 0.36 ± 0.05 | 0.30 ± 0.14 | 0.36 ± 0.15 |
| OGTT (mmol/L) | | | | |
| Glucose 0 min | 5.20 ± 0.63 | 6.12 ± 0.52 | 4.88 ± 0.68^b^ | 5.88 ± 0.46 |
| Glucose 15 min | 14.24 ± 2.52 | 14.48 ± 1.58 | 10.56 ± 1.11 | 9.76 ± 0.73 |
| Glucose 30 min | 13.42 ± 1.60 | 14.52 ± 1.76 | 10.40 ± 2.48 | 8.88 ± 1.12 |
| Glucose 60 min | 8.22 ± 1.08 | 11.08 ± 1.69 | 7.80 ± 1.08 | 7.06 ± 1.33 |
| Glucose 90 min | 7.16 ± 0.46 | 9.7 ± 1.30 | 7.44 ± 0.80 | 6.88 ± 0.99 |
| Glucose 120 min | 6.16 ± 0.50 | 8.18 ± 1.09 | 6.26 ± 0.38 | 6.30 ± 1.08 |
| Glucose AUC | 1108.00 ± 51.63 | 1336.00 ± 65.84 | 980.10 ± 58.50 | 903.00 ± 49.00 |

Values are mean ± SEMs (n = 15 per group). The multiple comparisons between data were performed using one-way ANOVA followed by Tukey’s multiple comparisons test for normally distributed data or the Kruskal–Walli’s test followed by the Mann–Whitney *U*-test for skewed data. *P* < 0.05 were considered statistically significant.

^a^PCOS versus Control group; ^b^PCOS + Met versus PCOS group. AUC, area under the curve; BW, body weight; CHOL, cholesterol; DHT, 5α-dihydrotestosterone; E2, 17β-estradiol; HDL, high-density lipoprotein; LDL, low-density lipoprotein; OGTT, oral glucose tolerance test; P4, progesterone; TG, triglycerides; TT, total testosterone.

**Table S6. Impacts of continuous treatment with DHT and metformin treatment on pregnancy outcomes of mice.**

|  | Ctrl | PCOS | PCOS + Met | Met |
| --- | --- | --- | --- | --- |
| Maternal outcome | | | | |
| Total dam (n) | 6 | 6 | 6 | 6 |
| Absence of labor (n) | 0 | 1^a^ | 0 | 0 |
| Litter size (n) | 7.05 ± 1.59 | 2.20 ± 0.75^a^ | 6.00 ± 1.96 | 7.55 ± 1.72 |
| Offspring outcome (postnatal day 1-2) | | | | |
| Male: Female | 1.06 ± 0.34 | 1.40 ± 0.49^a^ | 1.38 ± 0.52 | 0.99 ± 0.37 |
| Male (body weight, g) | 2.05 ± 0.11 | 1.72 ± 0.11^a^ | 1.96 ± 0.08^b^ | 2.11 ± 0.08 |
| Female (body weight, g) | 2.05 ± 0.06 | 1.67 ± 0.06^a^ | 1.99 ± 0.05^b^ | 2.09 ± 0.06 |

Maternal treatment with DHT decreased the number of labors and litter size. ^a^*P* < 0.05 PCOS versus Ctrl group; ^b^*P* < 0.05 PCOS + Met versus PCOS group.

**Table S7. Summary of TS^CT^ Cell Derivation.**

| TS line | Maternal age (years) | Gestational age at D&C | Karyotype | Doubling time  (Passage number) |
| --- | --- | --- | --- | --- |
| TS^CT^ #1 | 31 | 8 weeks | 46, XY | 25 ± 2.2 hours (P13) |
| TS^CT^ #2 | 24 | 6 weeks | 46, XX | 24 ± 4.7 hours (P13) |
| TS^CT^ #3 | 32 | 7 weeks | 46, XX | 27 ± 1.8 hours (P13) |

D&C, dilatation and curettage.

**Table S8. Formula of trophoblast stem cell (TS) medium and ST(2D) medium supplements added to DMEM/F12.**

|  | **Component** | **Concentration** | **Identifier and Company** |
| --- | --- | --- | --- |
| TS medium | 2-Mercaptoethanol | 0.1 mM | Cat#21985023  Thermo Fisher Scientific, USA |
|  | FBS | 0.2% (v/v) | Cat#16141-079  Thermo Fisher Scientific, USA |
|  | Penicillin-Streptomycin | 1% (v/v) | Cat#15140122  Thermo Fisher Scientific, USA |
|  | BSA | 0.3% (w/v) | Cat#017-22231  Wako, Japan |
|  | ITS-X supplement | 1% (v/v) | Cat#094-06761  Wako, Japan |
|  | L-ascorbic acid | 1.5 μg/ml | Cat#013-12061  Wako, Japan |
|  | EGF | 50 ng/ml | Cat#053-07871  Wako, Japan |
|  | CHIR99021 | 2 μM | Cat#038-23101  Wako, Japan |
|  | A83-01 | 0.5 μM | Cat#035-24113  Wako, Japan |
|  | SB431542 | 1 μM | Cat#031-24291  Wako, Japan |
|  | VPA | 0.8 mM | Cat#227-01071  Wako, Japan |
|  | Y27632 | 5 μM | Cat#257-00511  Wako, Japan |
| ST(2D) medium | 2-Mercaptoethanol | 0.1 mM | Cat#21985023  Thermo Fisher Scientific, USA |
|  | Penicillin-Streptomycin | 0.5% (v/v) | Cat#15140122  Thermo Fisher Scientific, USA |
|  | BSA | 0.3% (w/v) | Cat#017-22231  Wako, Japan |
|  | ITS-X supplement | 1% (v/v) | Cat#094-06761  Wako, Japan |
|  | Y27632 | 2.5 μM | Cat#257-00511  Wako, Japan |
|  | Forskolin | 2 μM | Cat#067-02191  Wako, Japan |
|  | KnockOut Serum Replacement | 4% (v/v) | Cat#10828028  Thermo Fisher Scientific, USA |

**Table S10. Primers for qRT-PCR.**

| Gene | Forward (5’-3’) | Reverse (5’-3’) |
| --- | --- | --- |
| *ACTB* | CGGAACCGCTCATTGCC | ACCCACACTGTGCCCATCTA |
| *AR* | GACAGAGGGAAAAAGGGCCGA | GCTTCCTCCGAGTCTTTAGCAG |
| *FTH1* | GCCAGAACTACCACCAGGACT | CAGCATGTTCCCTCTCCTCAT |
| *FTL* | GCTCCTTCTTGCCAACCAAC | GCCCAGAGAGAGGTAGGTGT |
| *HMOX1* | AAGACTGCGTTCCTGCTCAAC | AAAGCCCTACAGCAACTGTCG |
| *LAMP2A* | GTGCAACAAAGAGCAGACTGT | GGCACAAGGAAGTTGTCGTC |
| *STEAP3* | CCTCTGCAGAACCAGGTTGA | GAGCTCATGAAACCTCCCTGG |

**Table S11. Primers for CUT&Tag-qPCR.**

| Genes | Sites | Forward (5’-3’) | Reverse (5’-3’) |
| --- | --- | --- | --- |
| *LAMP2* | ARE1 | AAGCTGGAAGCCATCATCCTT | CCTCCCTGTGTCCATGTGTT |
|  | ARE2 | AACAGGGTGCTGTAACTAGGTG | GGTAGTATGGAGTCACGGACC |
|  | ARE3 | GACGGGTTGATAGGTGCAGT | CTGAGAGATATGCGCGGACG |
|  | ARE4 | ACCAACCCAAATGCCCATCA | TGCTAAGGATGATGGCTTCCA |
| *FTH1* | ARE1 | CGCGACAGAACAGTGGACCT | GGCGGGTGACAGGTGAAAGA |
|  | ARE2 | GGAGCCGAATCAGGATCACC | GCGGTGGCGTCTCTGT |
| DNA Spike-in |  | GCCTTCTTCCCATTTCTGATCC | CACGAATCAGCGGTAAAGGT |
| Positive Control |  | CGAAGACCCACTGCCCTTTTG | AAGAATGTCAACTACCACGAATGC |

**Table S12. Primers for siRNAs.**

| Genes | 001 site-targeted sequence (5’ to 3’) | 002 site-targeted sequence (5’ to 3’) |
| --- | --- | --- |
| *ATG5* | TGACGTTGGTAACTGACAA | GTGAGATATGGTTTGAATA |
| *ATG7* | ACTCGAGTCTTTCAAGACT | GAACGAGTATCGGCTGGAT |
| *FTH1* | GTCCATGTCTTACTACTTT | CGTTTACCTGTCCATGTCT |
| *HSPA8* | GAATTCACTTGAGTCCTAT | GATCGATTCTCTCTATGAA |
| *LAMP2A* | GCAGTGCAGATGACGACAA | TAGTGTTGCTGGCTTATTT |
| Negative Control | GATCATACGTGCGATCAGA |  |

**Table S13. Clinical characteristics of the recruited patients.**

| Parameters | HC (n = 20) | PNA (n = 10) | PHA (n = 20) |
| --- | --- | --- | --- |
| Age (years) | 26.10 ± 0.78 | 30.20 ± 1.65 | 28.10 ± 1.12 |
| BMI (kg/m^2^) | 20.12 ± 0.34 | 22.23 ± 1.15 | 21.50 ± 0.86 |
| Cycle length (days) | 30.95 ± 1.39 | 50.50 ± 7.43^a^ | 38.95 ± 2.63 |
| AMH (ng/mL) | 2.97 ± 0.26 | 9.43 ± 0.82^a^ | 6.94 ± 0.69^b,c^ |
| Baseline LH (IU/L) | 4.00 ± 0.57 | 10.44 ± 2.17^a^ | 7.41 ± 1.03 |
| Baseline FSH (IU/L) | 5.88 ± 0.38 | 4.99 ± 0.54 | 5.42 ± 0.43 |
| LH/FSH | 0.72 ± 0.11 | 2.08 ± 0.33^a^ | 1.44 ± 0.23^b^ |
| Baseline TT (ng/mL) | 0.36 ± 0.03 | 0.55 ± 0.03 | 1.02 ± 0.10^b,c^ |
| Baseline E2 (pg/mL) | 69.22 ± 7.48 | 46.51 ± 4.99 | 49.23 ± 6.56 |
| Gestational TT (ng/mL) | 0.95 ± 0.04 | 0.96 ± 0.04 | 1.58 ± 0.14^b,c^ |
| Gestational E2 (pg/mL) | 1033.32 ± 74.27 | 416.93 ± 82.56^a^ | 511.15 ± 97.36^b^ |
| Gestational Duration (days) | 51.00 ± 0.73 | 52.80 ± 1.55 | 52.20 ± 1.16 |

All the data are shown as the mean value ± SEM (HC and PHA group: n = 20 per group, PwHA group: n = 10 per group). The multiple comparisons between these groups were carried out using one-way ANOVA followed by Tukey’s multiple comparison test. *P* < 0.05 were considered statistically significant. ^a^PwHA versus HC group; ^b^PHA versus HC group; ^c^PHA versus PwHA group. BMI, body mass index; AMH, anti-Müllerian hormone; LH, luteinizing hormone; E2, 17β-estradiol; TT, total testosterone; HC, healthy control; PwHA, PCOS patients without hyperandrogenemia; PHA, PCOS patients with hyperandrogenemia.

**Table S14. Key antibodies and reagents.**

| **Reagent or resource** | **Source** | **Identifier** |
| --- | --- | --- |
| **Antibodies** | | |
| Atg5 (D5F5U) Rabbit mAb | Cell Signaling Technology | Cat#12994 |
| Atg7 (D12B11) Rabbit mAb | Cell Signaling Technology | Cat#8558 |
| Androgen Receptor (D6F11) XP Rabbit mAb | Cell Signaling Technology | Cat#5153 |
| Androgen receptor Polyclonal antibody | Proteintech | Cat#22089-1-AP |
| DYKDDDDK (flag) Tag mAb | Medical & Biological Laboratories | Cat#MBL-385 |
| Anti-Ferritin antibody [EPR3004Y] | Abcam | Cat#ab75973 |
| Ferritin heavy chain antibody (B-12) | Santa Cruz Biotechnology | Cat#sc-376594 |
| Anti-Heme Oxygenase 1 antibody [EPR1390Y] | Abcam | Cat#ab68477 |
| HSPA8/HSC70 antibody (B-6) | Santa Cruz Biotechnology | Cat#sc-7298 |
| Tri-Methyl-Histone H3 (Lys4) (C42D8) Rabbit mAb | Cell Signaling Technology | Cat#9751 |
| Anti-LAMP1 antibody [H4A3] | Abcam | Cat#ab25630 |
| Anti-LAMP2A antibody [EPR4207(2)] | Abcam | Cat#ab125068 |
| Anti-MAP1LC3B antibody | Sigma | Cat#L7543 |
| Anti-mCherry antibody | Abcam | Cat#ab183628 |
| MCT1 antibody | Merck Millipore | Cat#AB1286-I |
| MCT4 antibody (D-1) | Santa Cruz Biotechnology | Cat#sc-376140 |
| Anti-STEAP3 antibody [EPR9812] | Abcam | Cat#ab151566 |
| Anti-hCG beta antibody [5H4-E2] | Abcam | Cat#ab9582 |
| Choriogonadotropin β antibody (B-4) | Santa Cruz Biotechnology | Cat#sc-271062 |
| Beta Actin Monoclonal antibody | Proteintech | Cat#66009-1-Ig |
| Normal Rabbit IgG | Cell Signaling Technology | Cat#2729 |
| PE-conjugated anti-ITGA6 | Miltenyi Biotec | Cat#130-097-246 |
| Peroxidase AffiniPure™ Goat Anti-Rabbit IgG (H+L) | Jackson | Cat#111-035-003 |
| Peroxidase AffiniPure™ Goat Anti-Mouse IgG (H+L) | Jackson | Cat#115-035-003 |
| Goat anti-Rabbit IgG (H+L) Highly Cross-Adsorbed Secondary Antibody, Alexa Fluor 488 | Invitrogen | Cat#A-11034 |
| Goat anti-Mouse IgG (H+L) Cross-Adsorbed Secondary Antibody, Alexa Fluor 488 | Invitrogen | Cat#A-11001 |
| Goat Anti-Chicken IgY H&L (Alexa Fluor 488) | Abcam | Cat#ab150169 |
| Goat anti-Rabbit IgG (H+L) Highly Cross-Adsorbed Secondary Antibody, Alexa Fluor 568 | Invitrogen | Cat#A-11036 |
| Donkey anti-Mouse IgG (H+L) Highly Cross-Adsorbed Secondary Antibody, Alexa Fluor 568 | Invitrogen | Cat#A10037 |
| Alexa Fluor 647 AffiniPure Goat Anti-Mouse IgG (H+L) | Yeasen | Cat#33213ES60 |
| Goat anti-Rabbit IgG H&L Unconjugated Secondary Antibody for CUT&Tag | Vazyme | Cat#Ab206-01 |
| Normal mouse IgG | Santa Cruz Biotechnology | Cat#sc-2025 |
| **Chemicals** | | |
| DMEM/F-12 | Gibco | Cat#11320-033 |
| DMEM | Gibco | Cat#11995065 |
| RPMI 1640 | Gibco | Cat#C11875500BT |
| TrypLE | Gibco | Cat#12563029 |
| Accumax | Innovative Cell Tech | Cat#AM105-500 |
| Collagen IV | Corning | Cat#354233 |
| Matrigel | Corning | Cat#354234 |
| 0.25% Trypsin-EDTA Solution | Solarbio | Cat#T1300 |
| FBS | Thermo Fisher Scientific | Cat#16141-079 |
| CA–FBS | Nuoyangbio | Cat#CMS003.02 |
| BSA | Sigma | Cat#V900933 |
| Penicillin-Streptomycin | Thermo Fisher Scientific | Cat#15140122 |
| Cell Banker 1 | Nippon Zenyaku Kogyo | Cat#CB011 |
| ITS-X supplement | Wako | Cat#094-06761 |
| 2-Mercaptoethanol | Thermo Fisher Scientific | Cat#21985023 |
| Y27632 | Wako | Cat#253-00513 |
| CHIR99021 | Wako | Cat#034-23103 |
| A83-01 | Wako | Cat#035-24113 |
| SB431542 | Wako | Cat#031-24291 |
| rEGF | Wako | Cat#053-07871 |
| VPA | Wako | Cat#227-01071 |
| L-Ascorbic acid | Wako | Cat#013-12061 |
| Erastin | MCE | Cat#HY-15763 |
| Ferrostatin-1 | MCE | Cat#HY-100579 |
| Deferiprone | MCE | Cat#HY-B0568 |
| Z-VAD-FMK | Beyotime | Cat#C1202 |
| Necrostatin-1 | Beyotime | Cat#SC4359 |
| AR7 | MCE | Cat#HY-101106 |
| 3-Methyladenine | Selleck | Cat#S2767 |
| Bafilomycin A1 | MCE | Cat#HY-100558 |
| Chloroquine | Selleck | Cat#S6999 |
| Ammonium chloride (NH_4_Cl) | MCE | Cat#HY-Y1269 |
| Leupeptin hemisulfate | MCE | Cat#HY-18234A |
| Cycloheximide | MCE | Cat#HY-12320 |
| MG-132 | MCE | Cat#HY-13259 |
| D-(+)-Glucose | Sigma | Cat#G7021 |
| Metformin hydrochloride | MCE | Cat#HY-17471A |
| Dihydrotestosterone | Selleck | Cat#S4757 |
| Testosterone | MCE | Cat#HY-17434 |
| Flutamide | MCE | Cat#HY-B0022 |
| Enzalutamide | MCE | Cat#HY-70002 |
| ARCC-4 | MCE | Cat#HY-130492 |
| DMSO | Solarbio | Cat#D8371 |
| 4% Paraformaldehyde | Meilunbio | Cat#MA0192 |
| 25% Glutaraldehyde | Macklin | Cat#G849973 |
| RIPA buffer (high) | Solarbio | Cat#R0010 |
| 4×Laemmli Sample buffer | Bio-Rad | Cat#1610747 |
| Cell lysis buffer for Western and IP | Beyotime | Cat#P0013 |
| PMSF | Beyotime | Cat#ST506 |
| Cocktail Protease Inhibitor | Roche | Cat#04693116001 |
| Immobilon Western HRP Substrate Kit | Millipore | Cat#WBKLS0500 |
| Trizol Reagent | Vazyme | Cat#R401-01 |
| HiScript II qRT SuperMix | Vazyme | Cat#R201-1 |
| 2×ChamQ Universal SYBR qPCR Master Mix | Vazyme | Cat#Q711 |
| Phen Green SK | Invitrogen | Cat#P14312 |
| Hoechst 33342 | Thermo Fisher Scientific | Cat#H3570 |
| Triton X-100 | Sigma | Cat#T8787 |
| MTS solution | Promega | Cat#G3580 |
| Coomassie blue | Beyotime | Cat#POO17FFT |
| OPTI-MEM | Thermo Fisher Scientific | Cat#31985062 |
| Lipofectamine 3000 Transfection Reagent | Invitrogen | Cat#L3000015 |
| SUMO Protease | Beyotime | Cat#P2312 |
| High-capacity Streptavidin-coated Agarose | Beyotime | Cat# P2159 |
| Anti-FLAG M2 Magnetic Beads | Sigma | Cat#M8823 |
| **Critical commercial assays** | | |
| Hyperactive Universal CUT&Tag Assay Kit | Vazyme | Cat#TD904 |
| QuikChange II Site-directed Mutagenesis Kit | Stratagene | Cat#200522 |
| Lipid Peroxidation (MDA) Assay Kit (Colorimetric/Fluorometric) | Abcam | Cat#ab118970 |
| Iron Assay Kit (Colorimetric) | Abcam | Cat#ab83366 |
| Dead Cell Apoptosis Kit with Annexin V Alexa Fluor 488 & Propidium Iodide | Thermo Fisher Scientific | Cat#V13245 |
| Mouse DHT ELISA Kit | Elabscience | Cat#E-EL-0031c |
| EasySep PE selection kit | Stem Cell Technologies | Cat#18551 |
| Pierce BCA Protein Assay Kit | Thermo Fisher Scientific | Cat#23225 |
| SDS-PAGE Kit | Epizyme Biotech | Cat#PG113, Cat#PG114 |
| Immunoprecipitation Kit with Protein A+G Magnetic Beads | Beyotime | Cat#P2179 |
| His-tag Protein Purification Kit | Beyotime | Cat#P2229 |
| RNA Rapid extraction kit | ESscience | Cat#RN001 |
| **Recombinant DNA** | | |
| pcDNA3.1(+)-AR | Qingke | N/A |
| pcDNA3.4-His-Sumo-AR | Public Protein/Plasmid Library | Cat#PPL00559-2c |
| pcDNA3.4-AR (delAA702-709) | Public Protein/Plasmid Library | Cat#PPL00559-2d |
| pcDNA3.1(+)-FLAG-FTH1 | Public Protein/Plasmid Library | Cat#PPL01716-2a |
| pmCherry-N1-FTH1 | Public Protein/Plasmid Library | Cat#PPL01716-2b |
| pcDNA3.1(+)-FLAG-FTH1 (Q84A/D85A) | Public Protein/Plasmid Library | Cat#PPL01716-2f |
| pmCherry-N1-FTH1 (Q84A/D85A) | Public Protein/Plasmid Library | Cat#PPL01716-2e |
| pmCherry-N1-LAMP2 | Public Protein/Plasmid Library | Cat#PPL01111-2b |
| pcDNA3.1(+)-FLAG | This paper | N/A |
| **Software, Algorithms and Database** | | |
| FlowJo software (v10.8.1) | https://www.flowjo.com/ | N/A |
| GSEA (v3.0) | https://www.broadinstitute.org/gsea | N/A |
| Open Babel software (v8.1) | https://github.com/openbabel/openbabel/releases | N/A |
| AutoDock Vina software (v1.2.0) | https://vina.scripps.edu/ | N/A |
| PyMOL (v2.5.2) | https://www.pymol.org/ | N/A |
| GROMACS (v2022.3) | https://www.gromacs.org/ | N/A |
| Proteome Discoverer (v3.0) | https://www.thermofisher.cn/order/catalog/product/OPTON-31099 | N/A |
| Cufflinks (v2.1.1) | http://cole-trapnell-lab.github.io/cufflinks/ | N/A |
| Cluster Profiler (v3.18.1) | https://rdrr.io/bioc/clusterProfiler/man/ | N/A |
| R (v3.5.1) | http://www.R-project.org/ | N/A |
| JASPAR database | https://jaspar.genereg.net/ | N/A |
| Uniprot database | https://www.uniprot.org/ | N/A |
| UCSC Genome Browser | https://genome.ucsc.edu/ | N/A |
| PrimerBank | https://pga.mgh.harvard.edu/primerbank/ | N/A |
| GraphPad Prism software (v10.1.2) | https://www.graphpad.com/ | N/A |
