# Supplementary material for: Hyperandrogenemia Induces Trophoblast Ferroptosis and Early Pregnancy Loss in Patients With PCOS via CMA‐Dependent FTH1 Degradation
Source: Adv Sci (Weinh). 2025 Dec 16;13(12):e06091. doi: 10.1002/advs.202506091 (PMC12948281; doi:10.1002/advs.202506091)

# All western blots original data

## Figure 1F：

The first repeat: The second repeat: The third repeat:

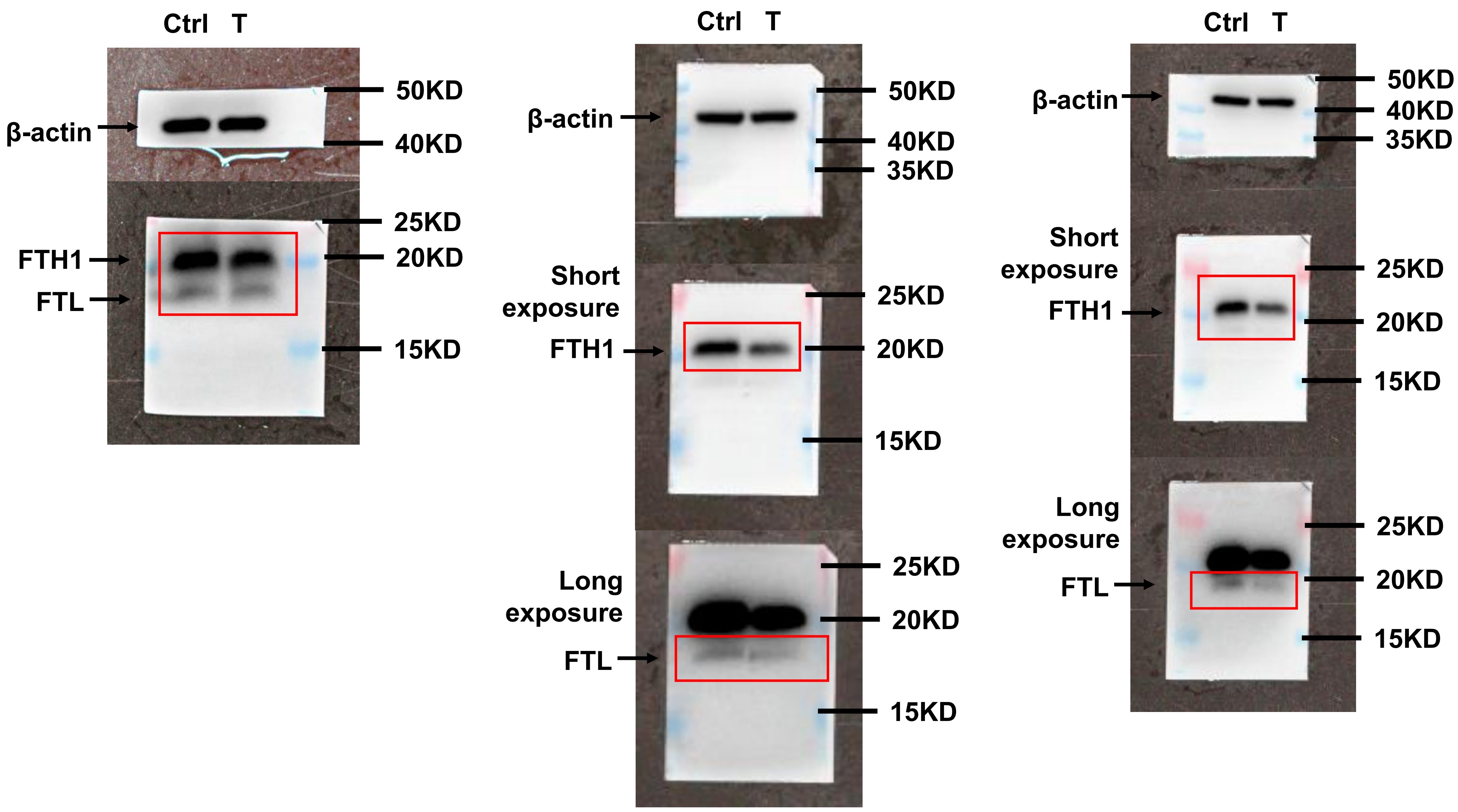


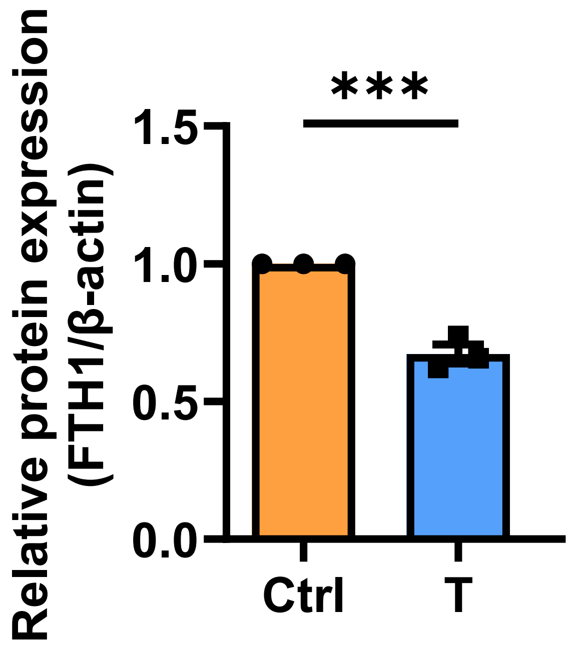

**Figure 1I:**

**The first repeat:**

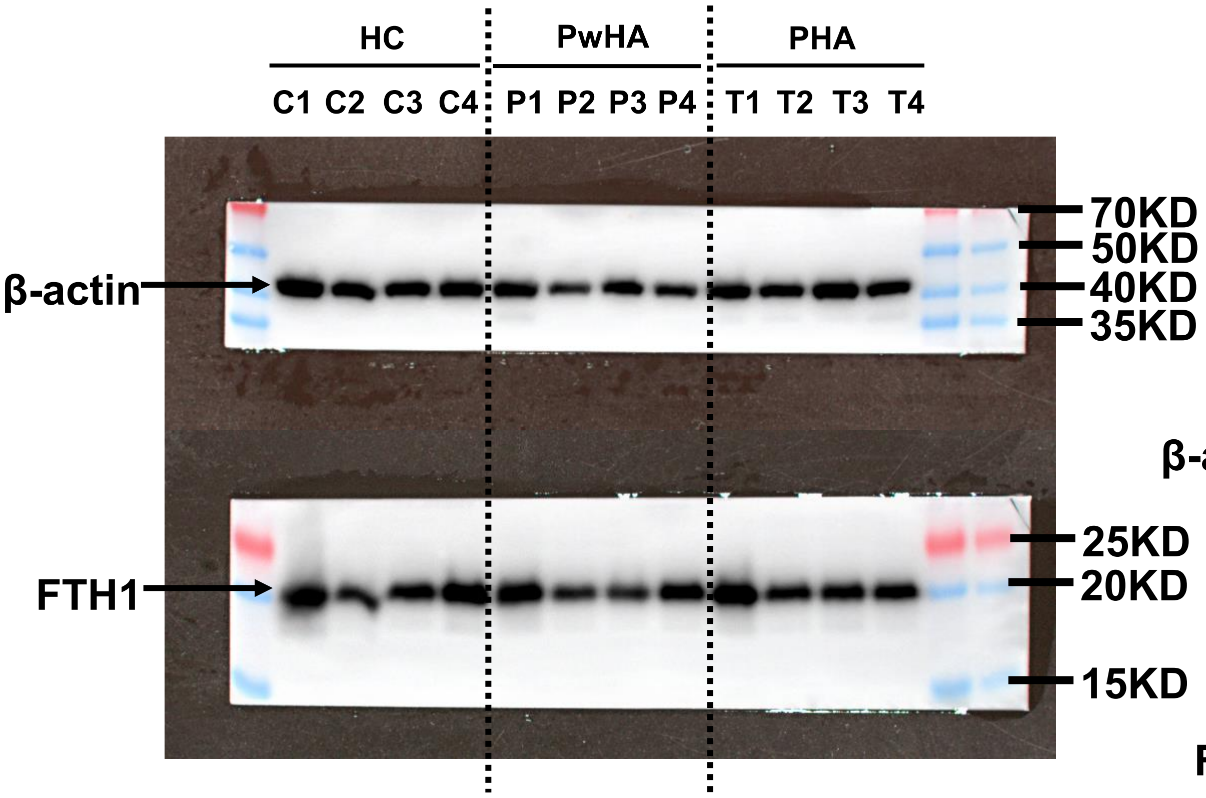


**The second repeat:**

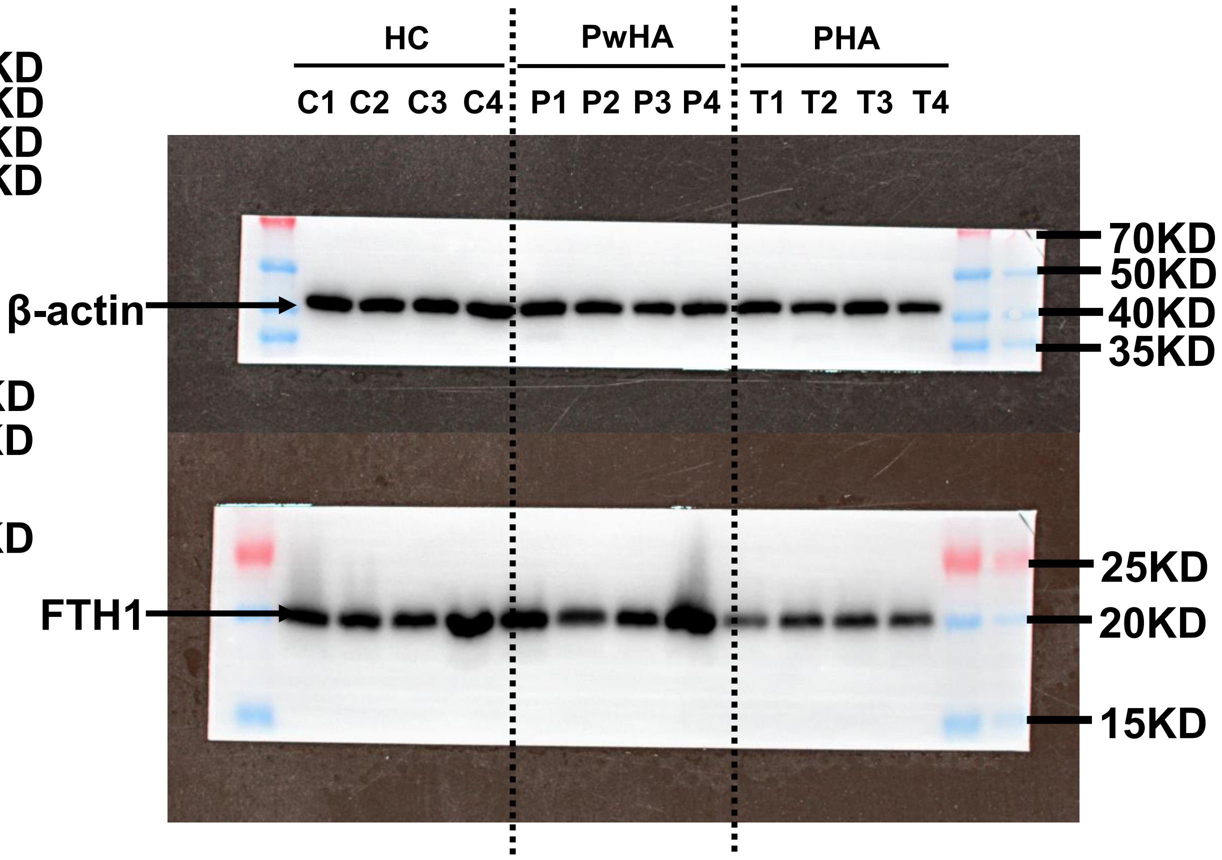


**The third repeat:**

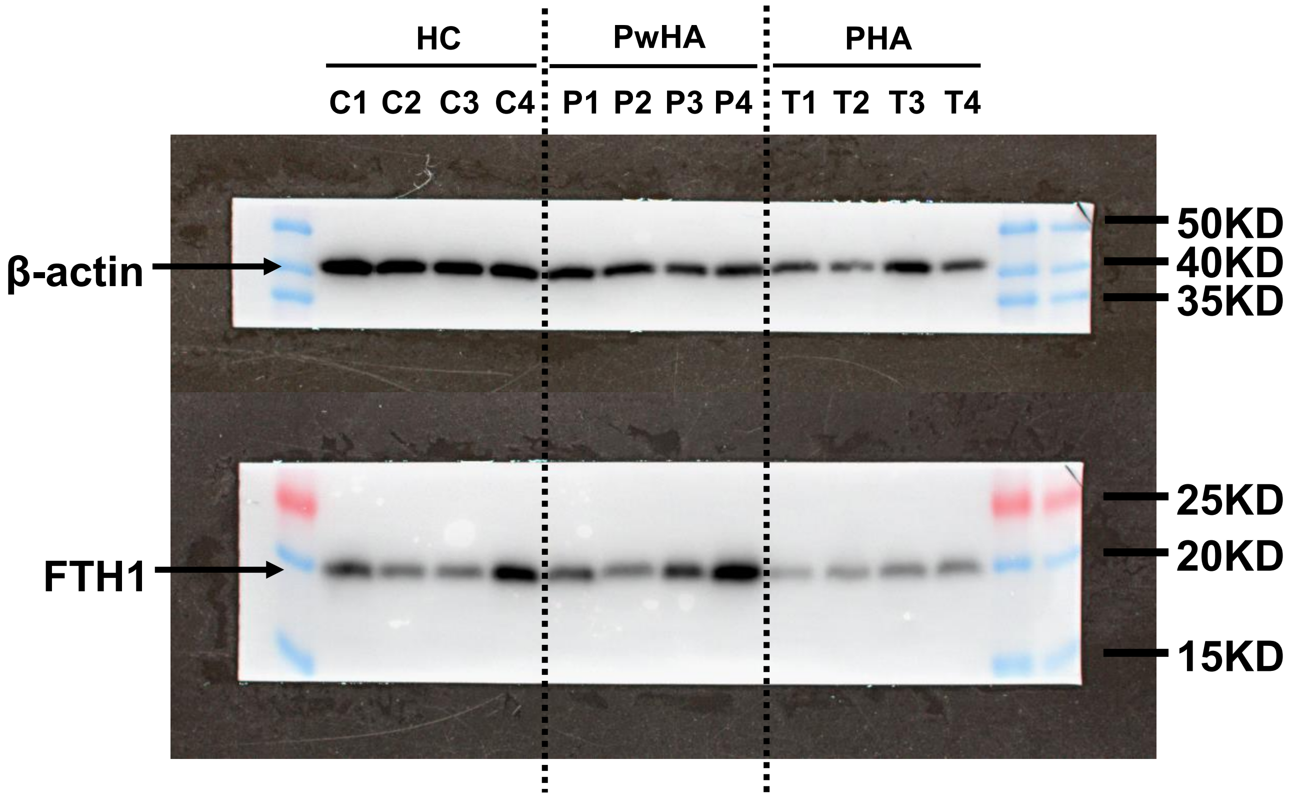


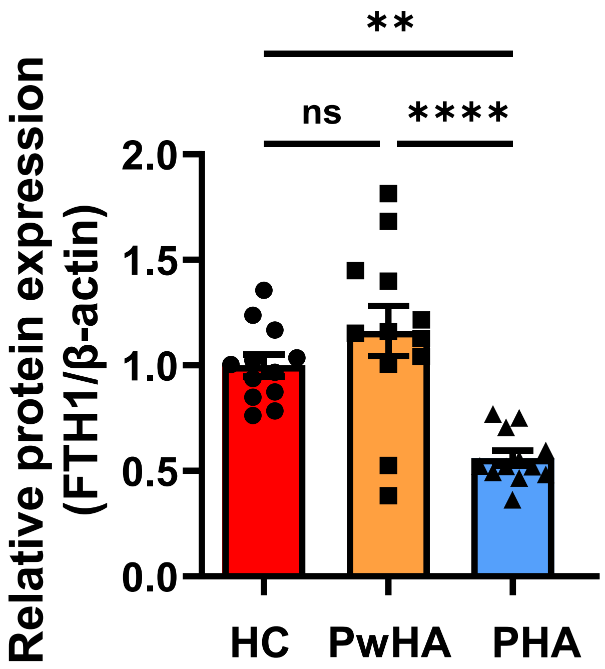

Figure 1K：

The first repeat:

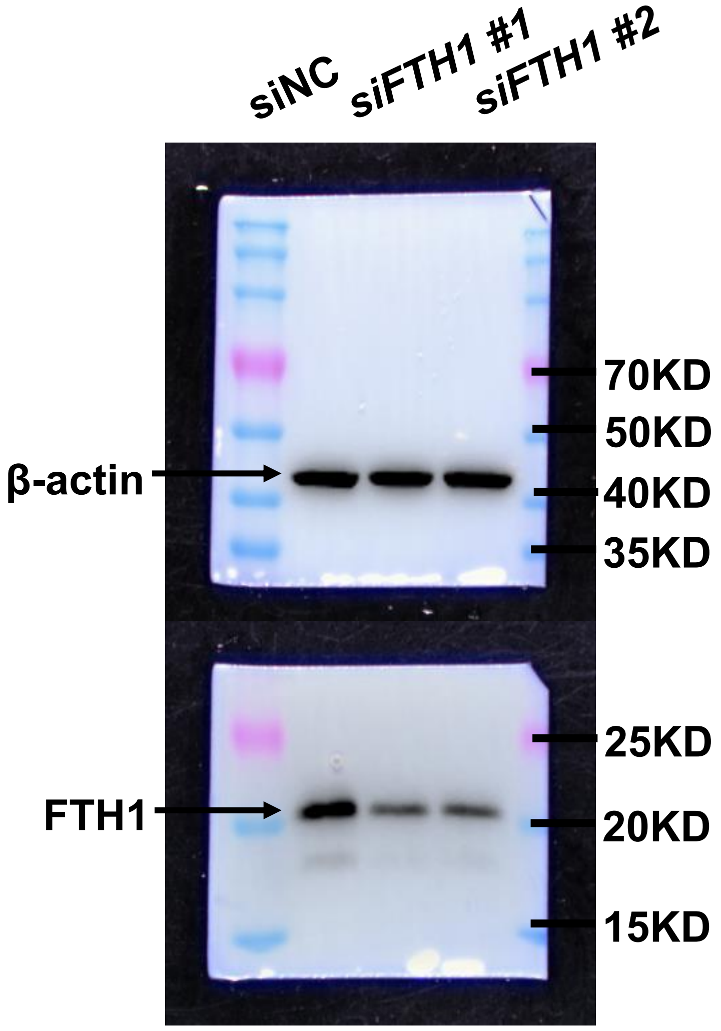


The second repeat:

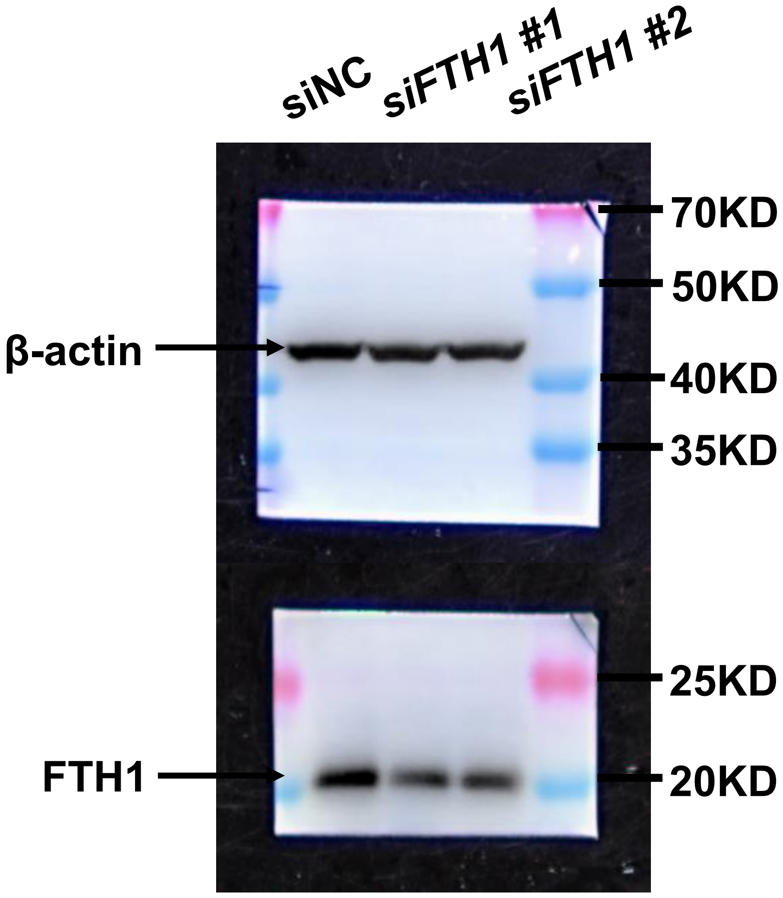


The third repeat:

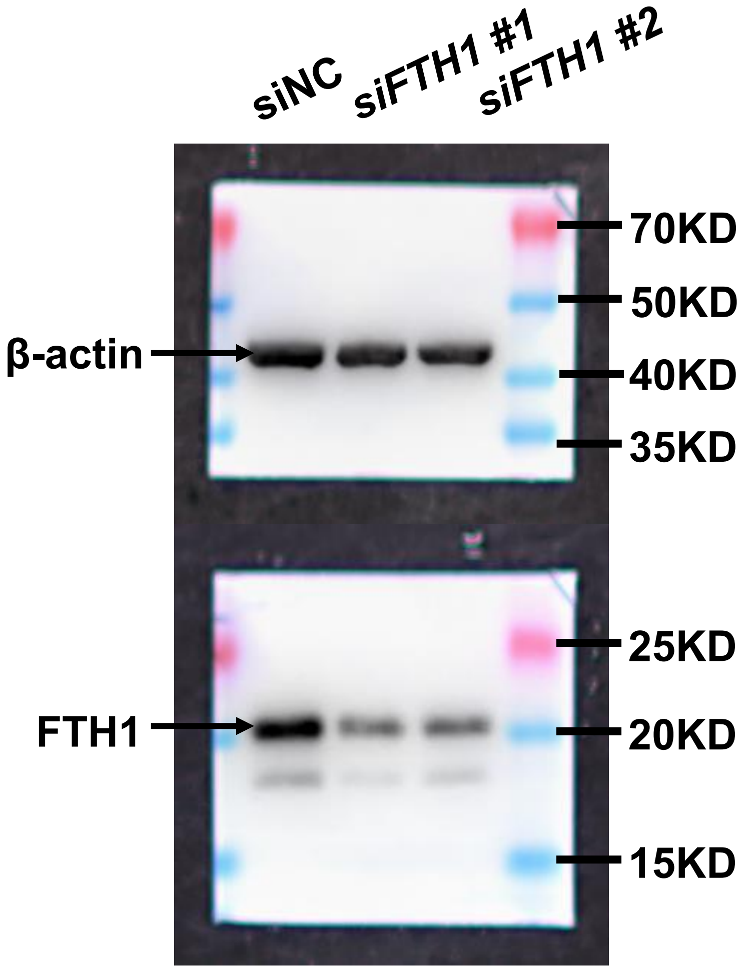


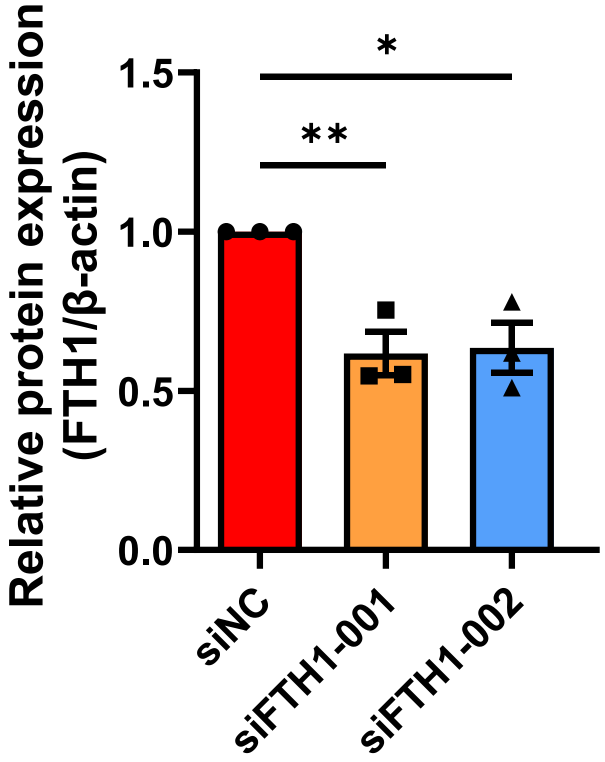

**Figure 2A:**

**The first repeat:**

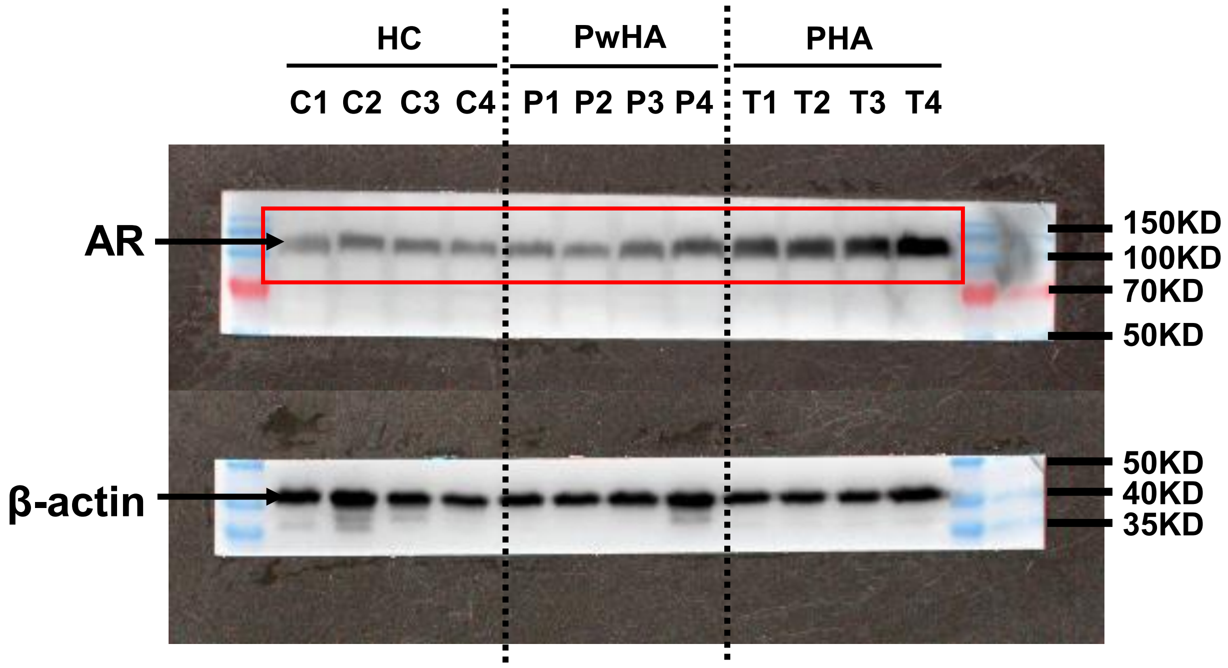


**The second repeat:**

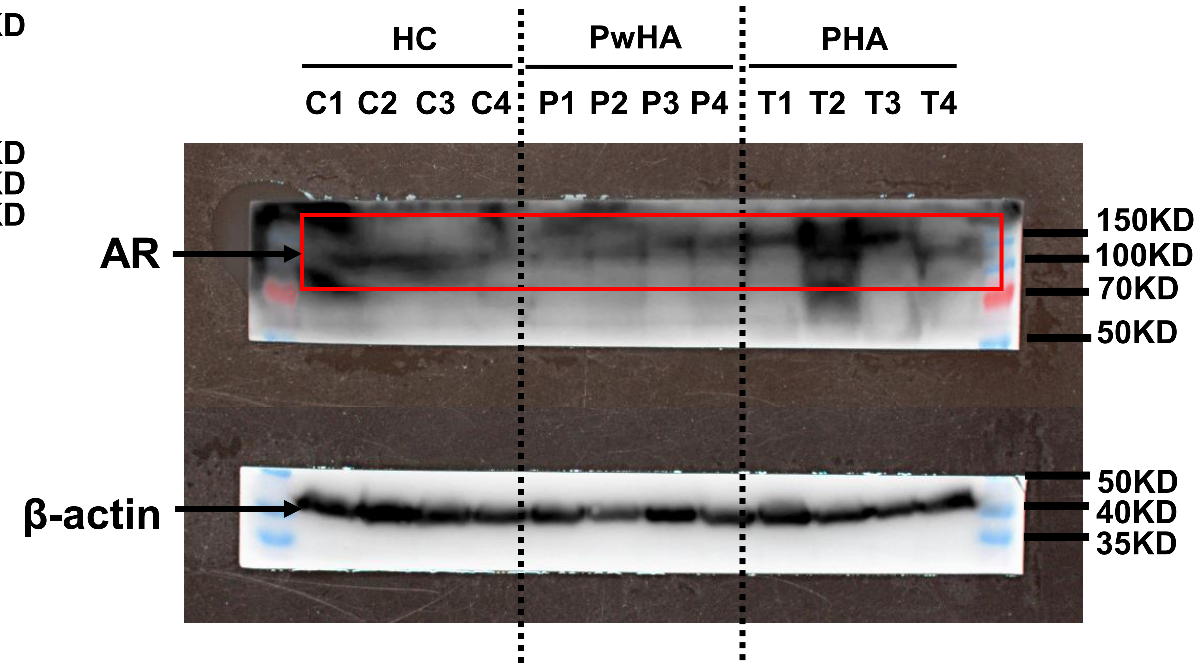


**The third repeat:**

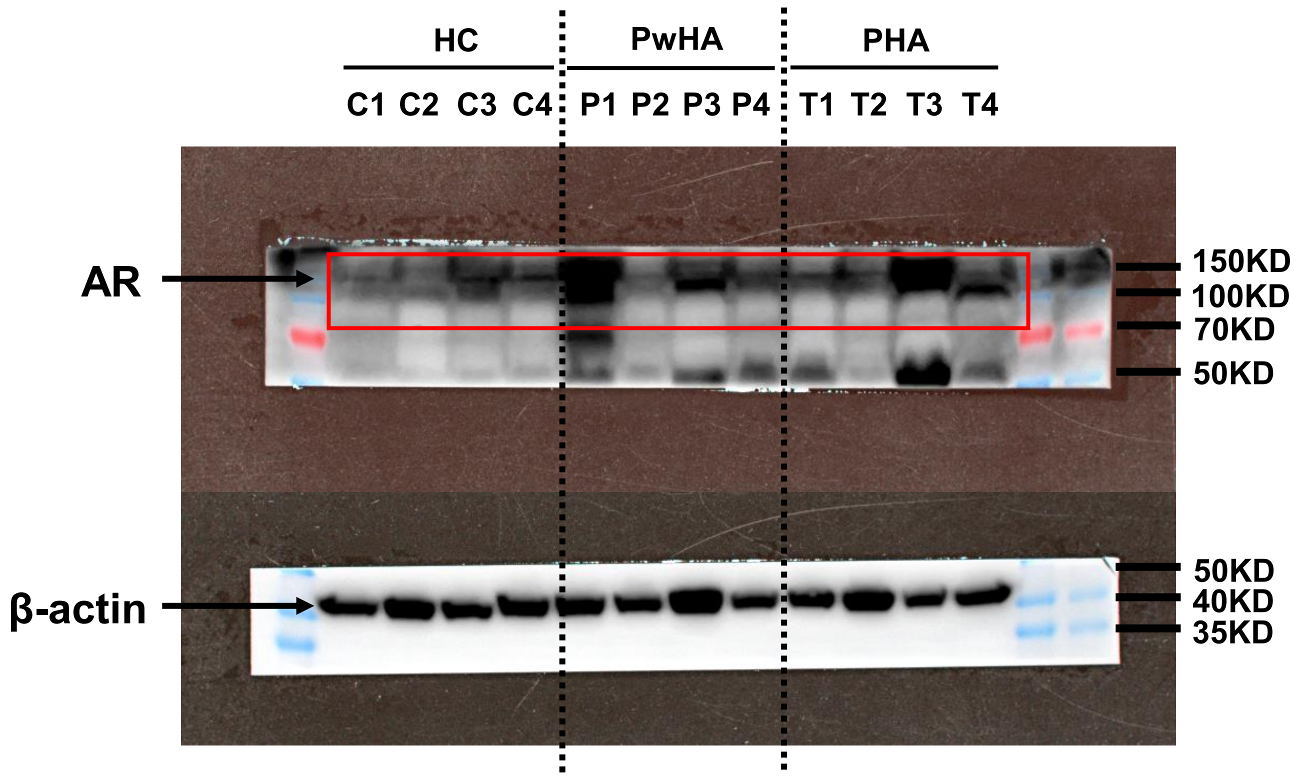


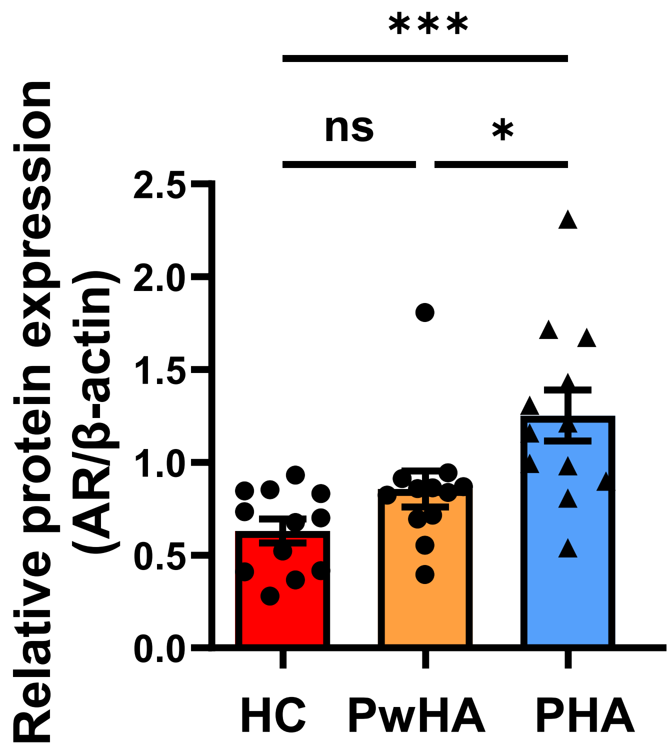


PS: The first repeat in Figure 2A and the second repeat in Figure 4B were derived from the same experiment, the WB membrane was washed with the stripping buffer.

**Figure 2E:**

**The first repeat:**

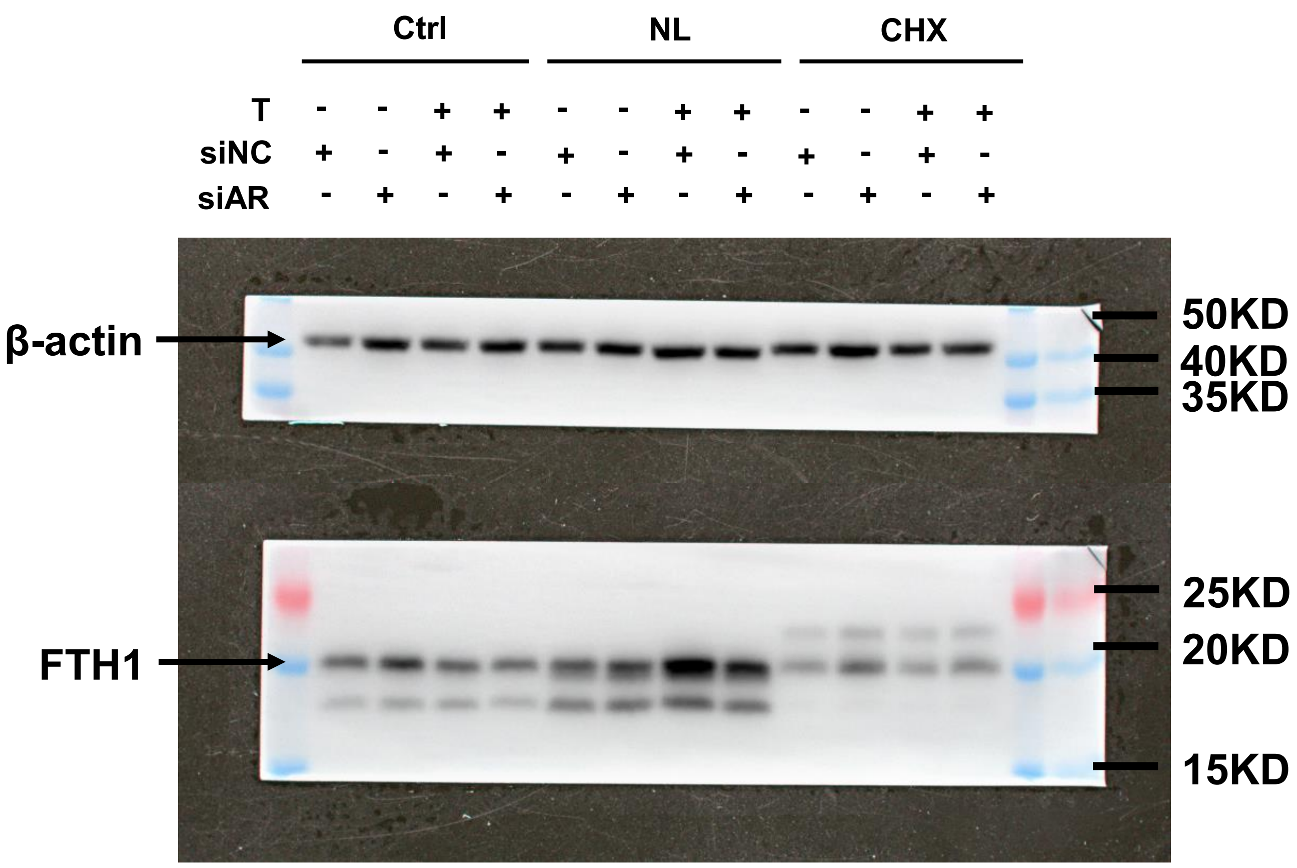


**The third repeat:**

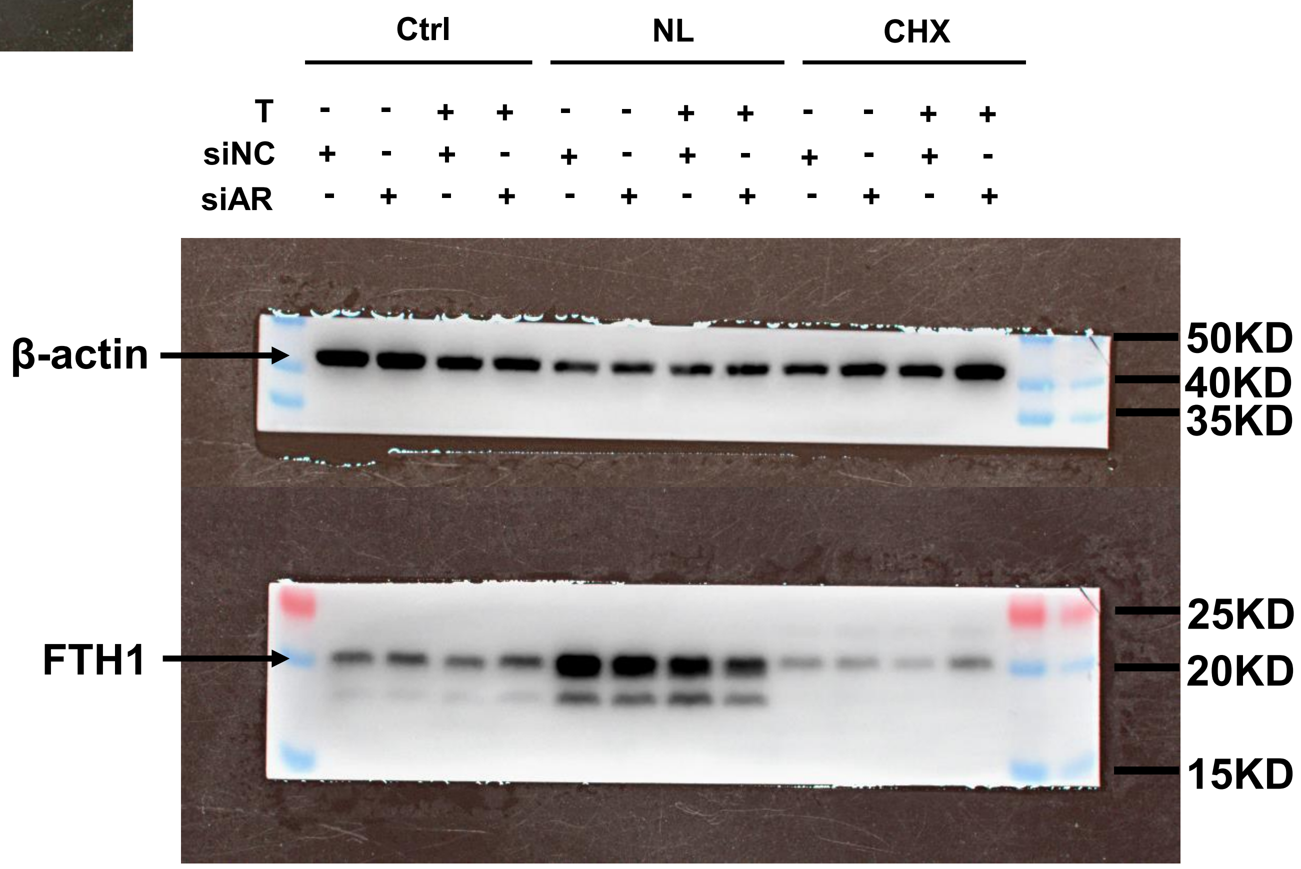


**The second repeat:**

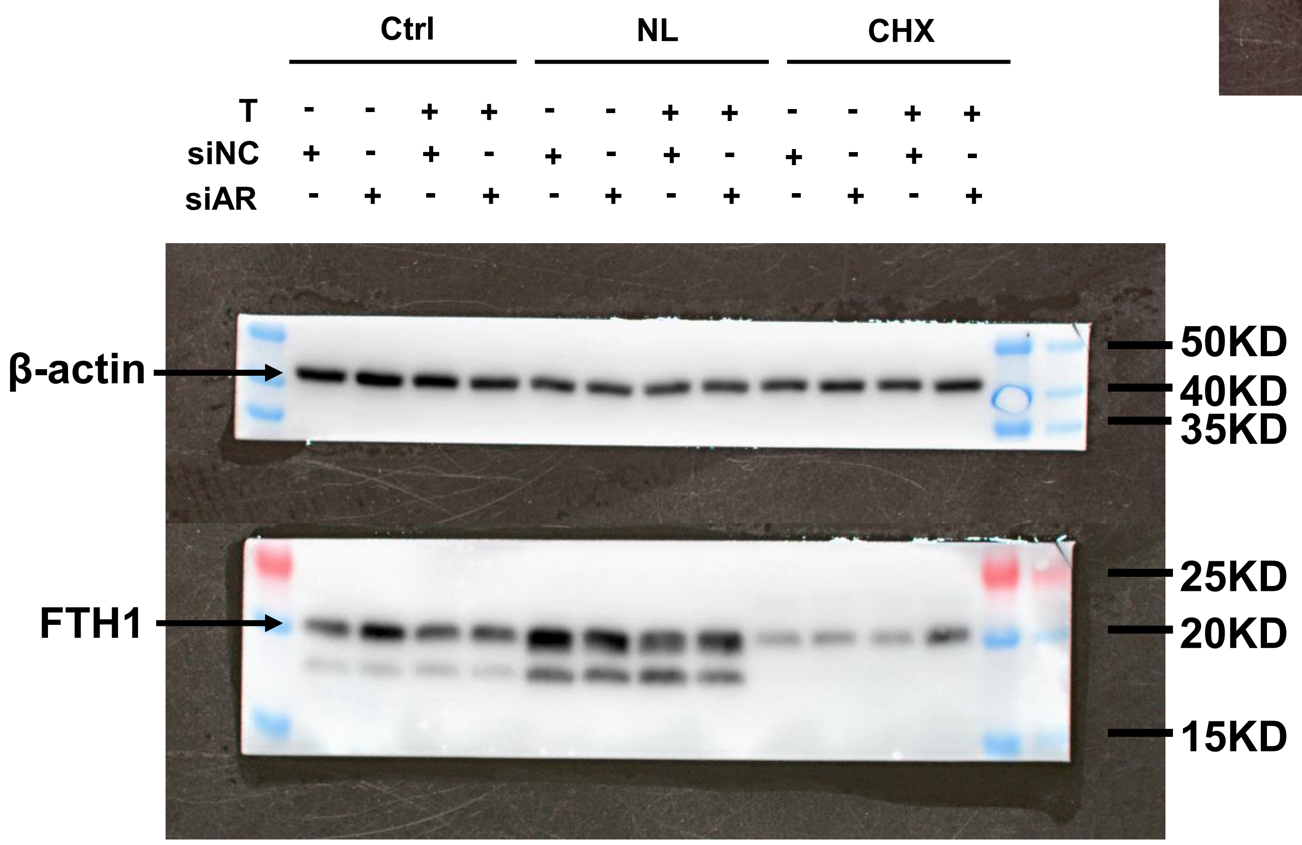


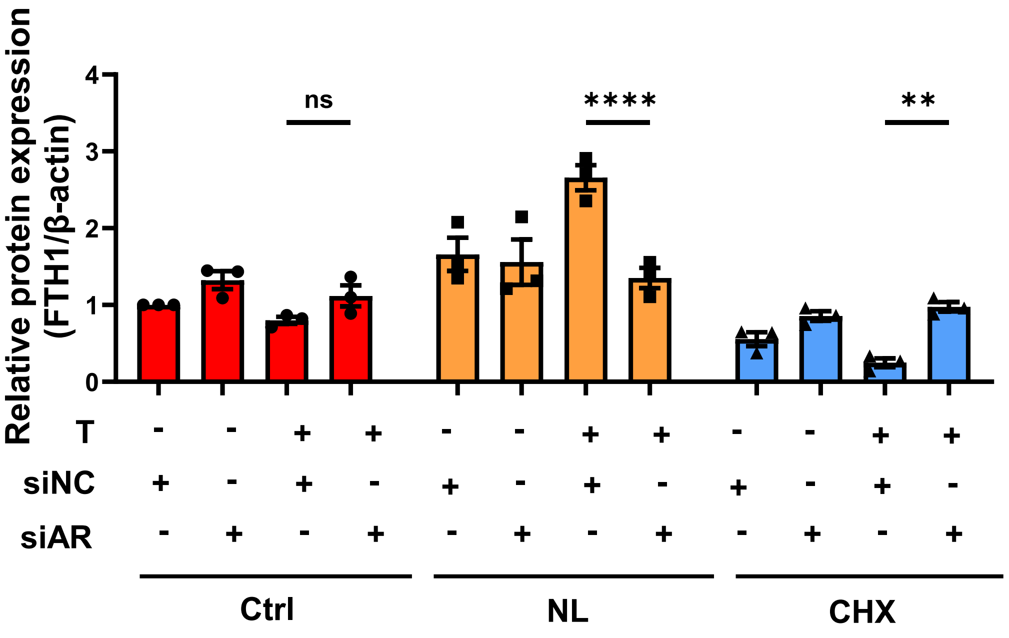

**Figure 2H：**

**The first repeat:**

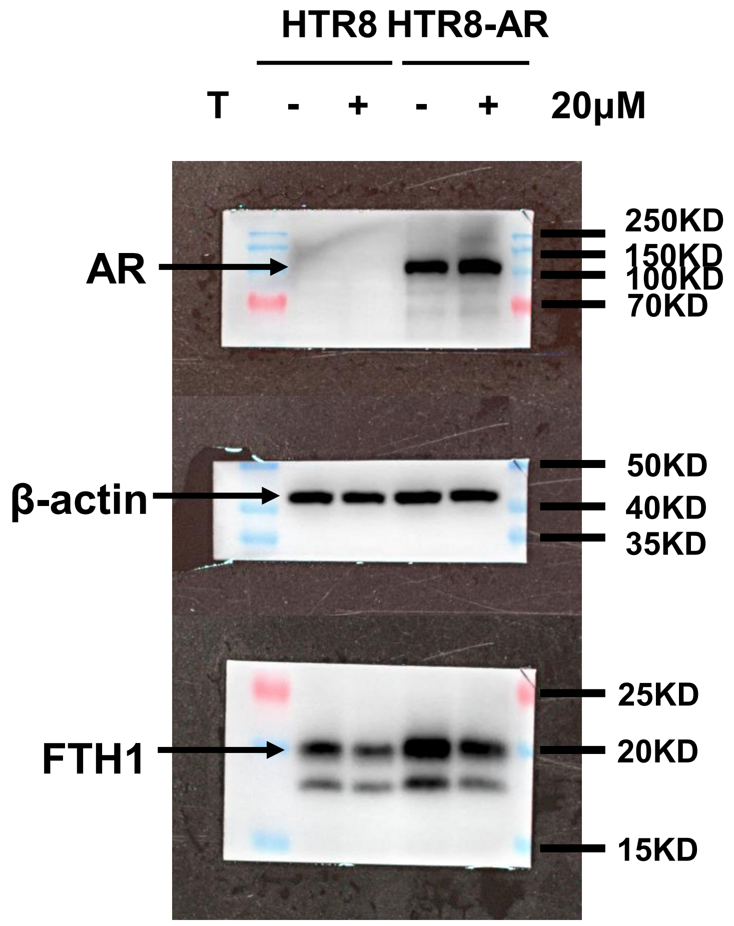


**The third repeat:**

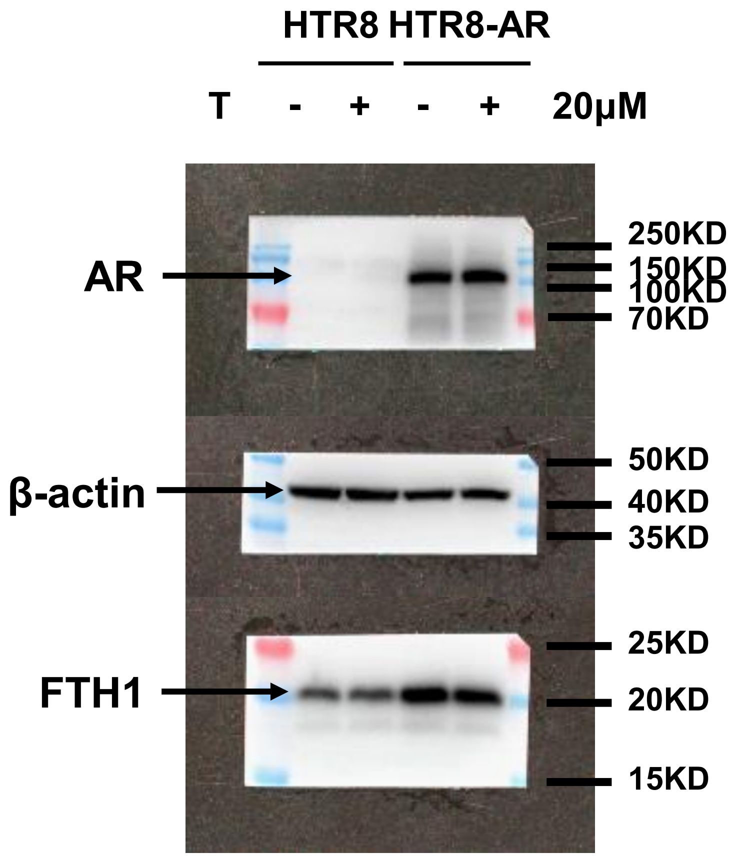


**The second repeat:**

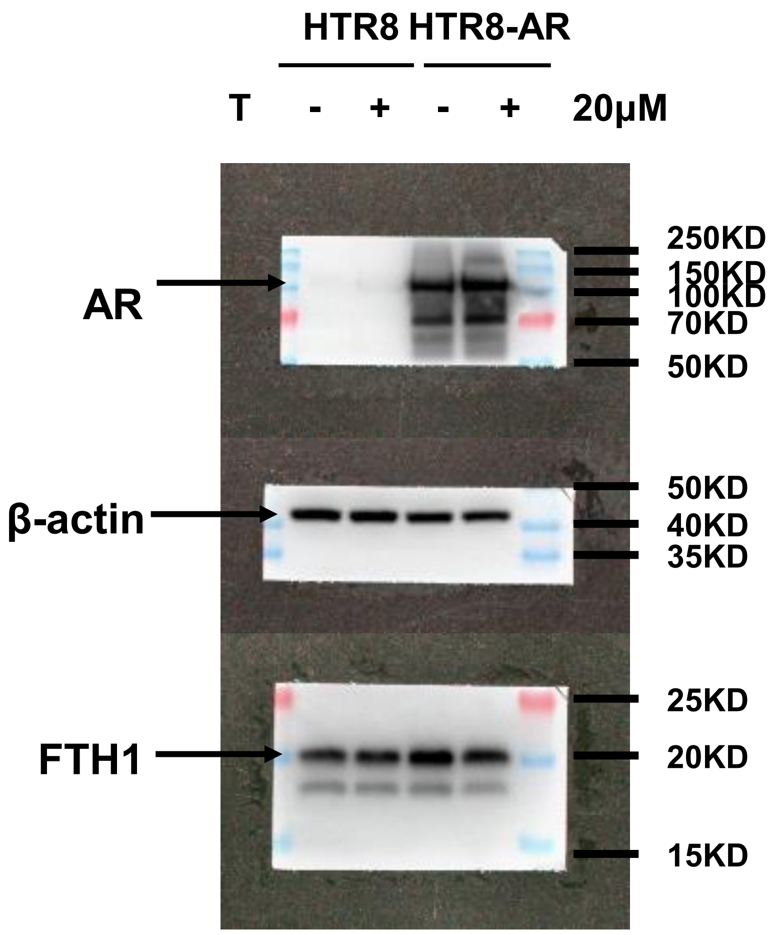


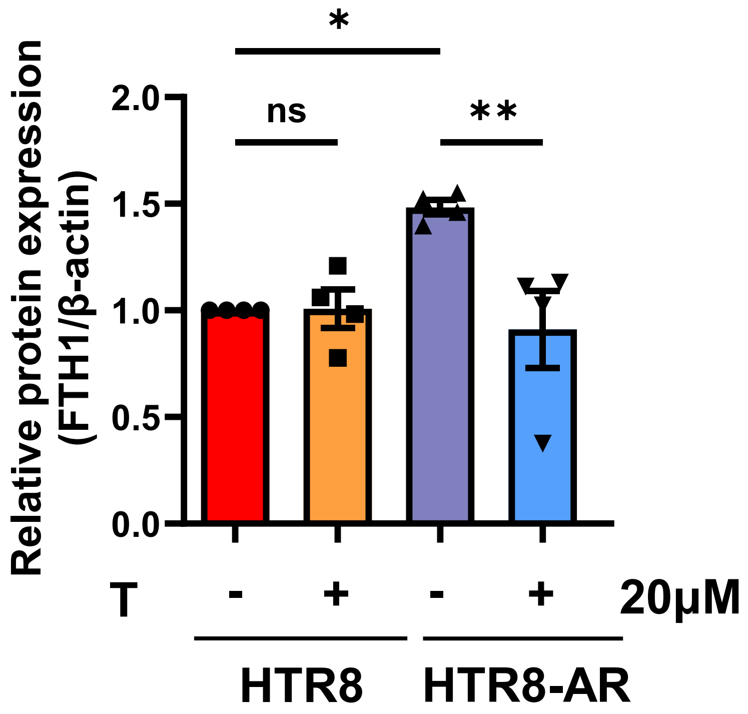

**Figure 2K：**

**The first repeat:**

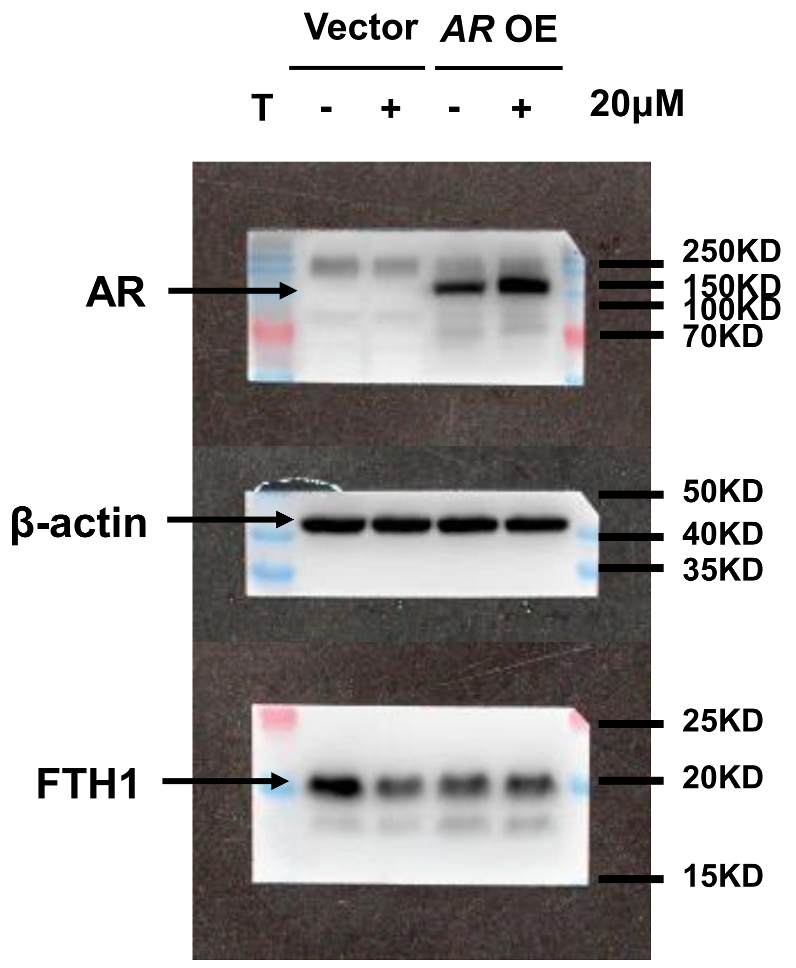


**The third repeat:**

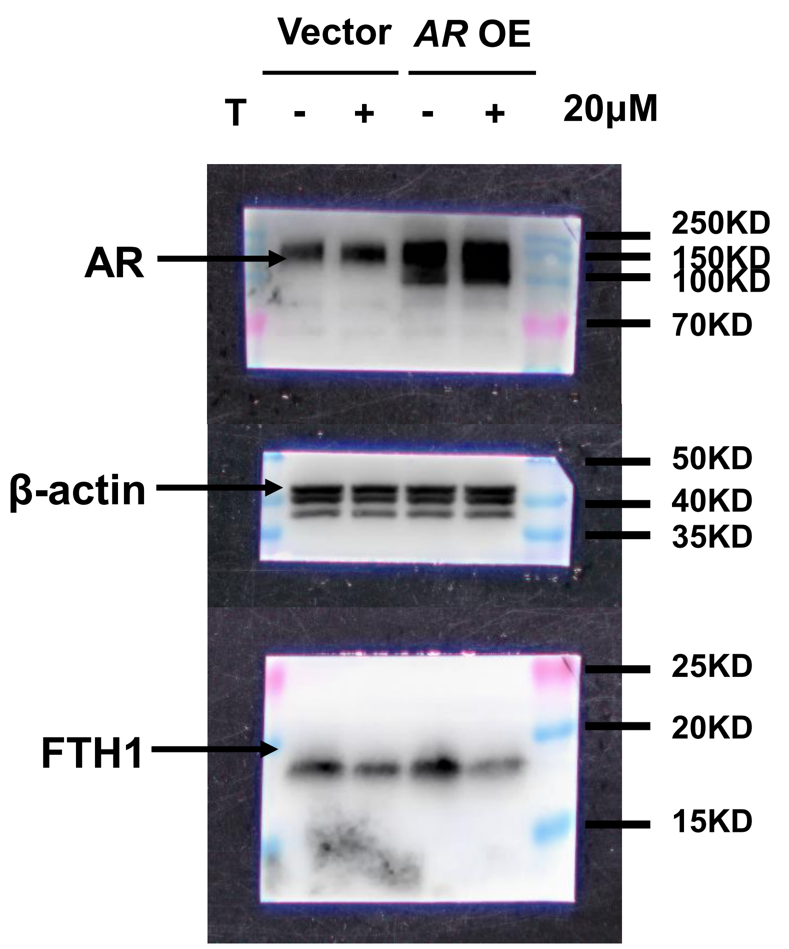


**The second repeat:**

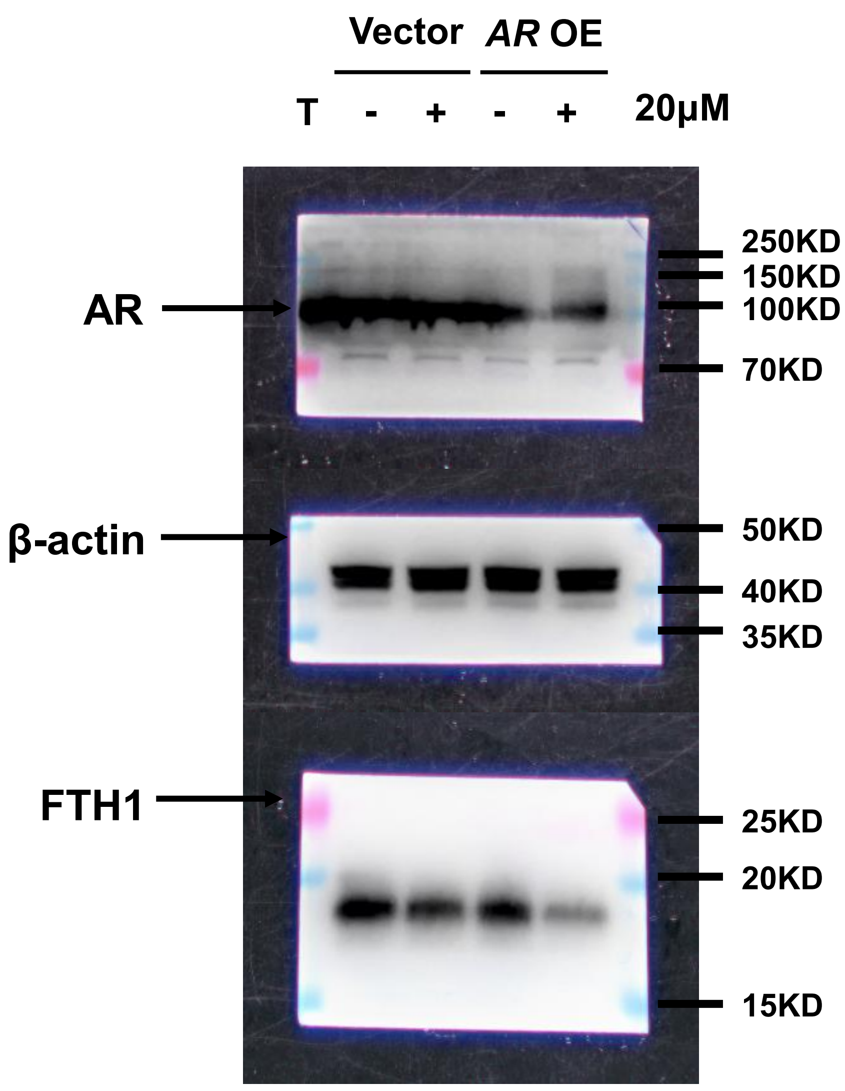


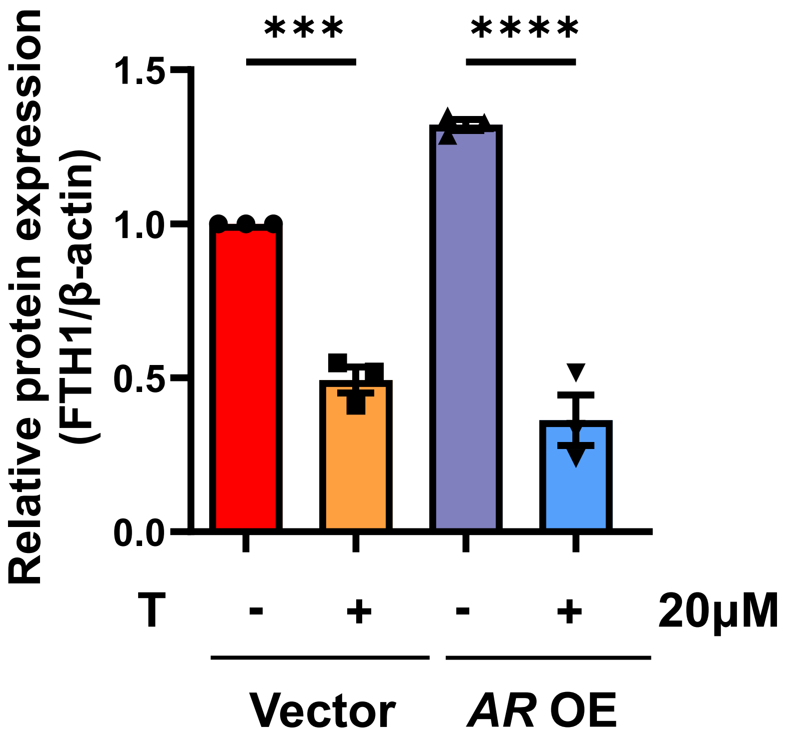

## Figure 2M：

### The first repeat:

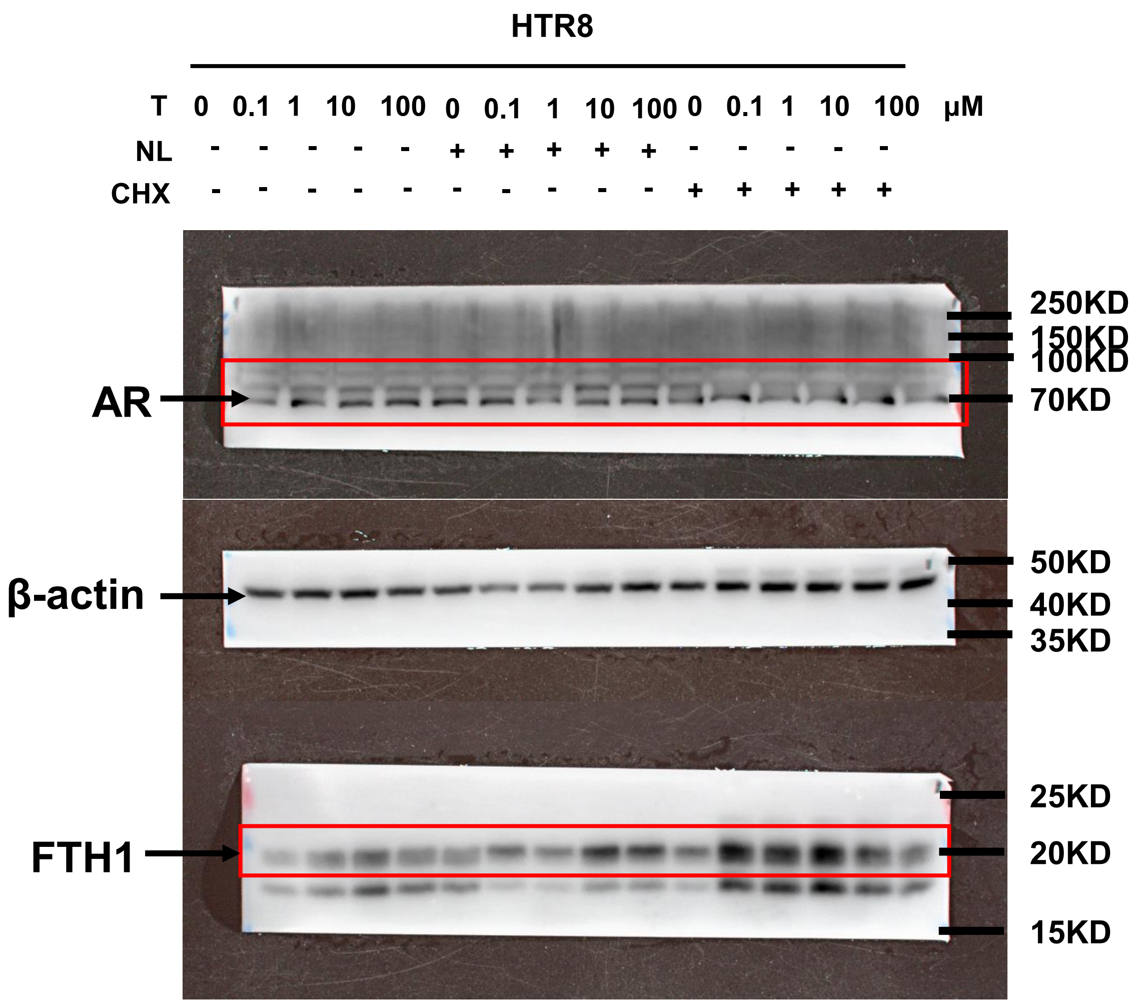


### The second repeat:

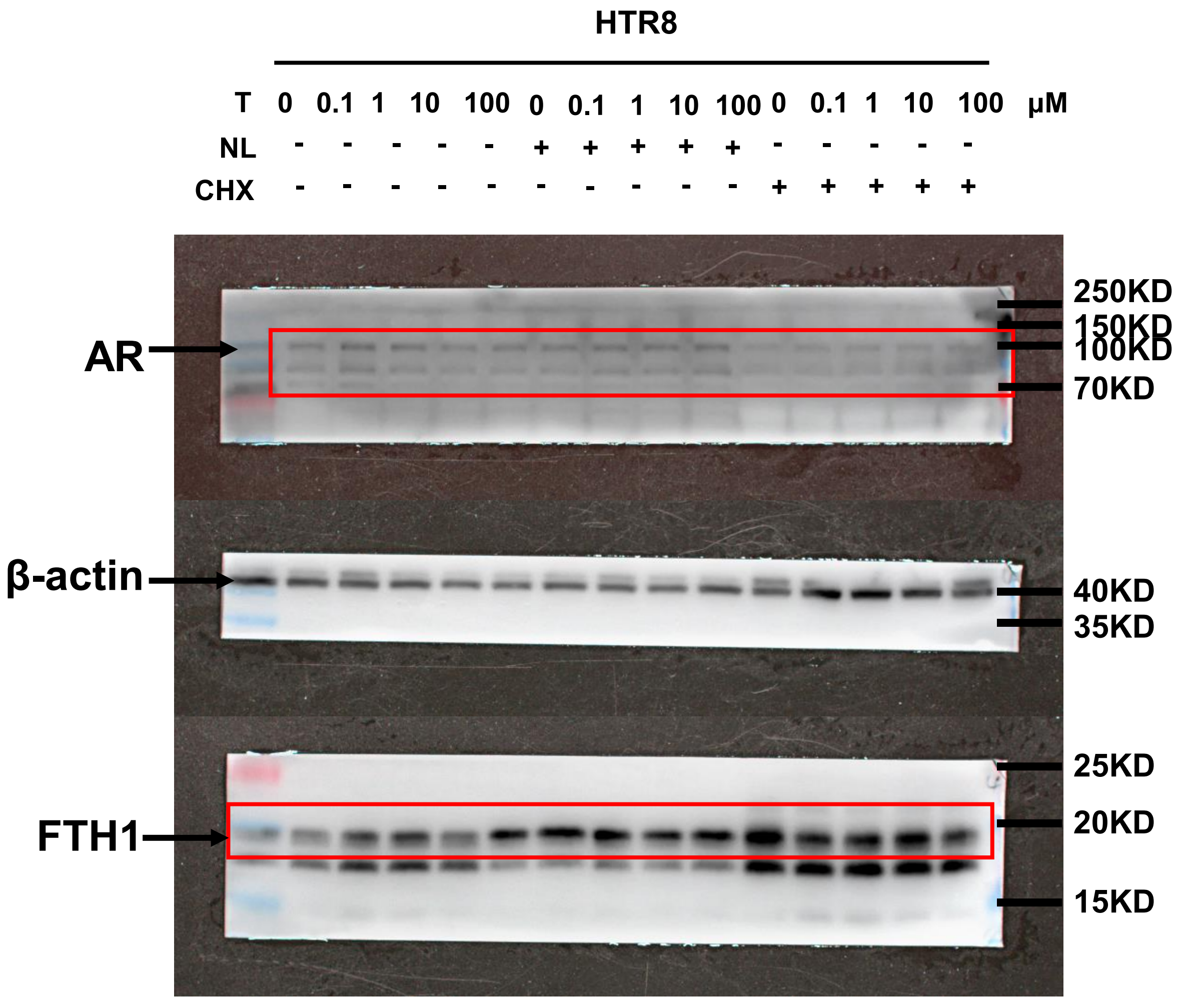


### The third repeat:

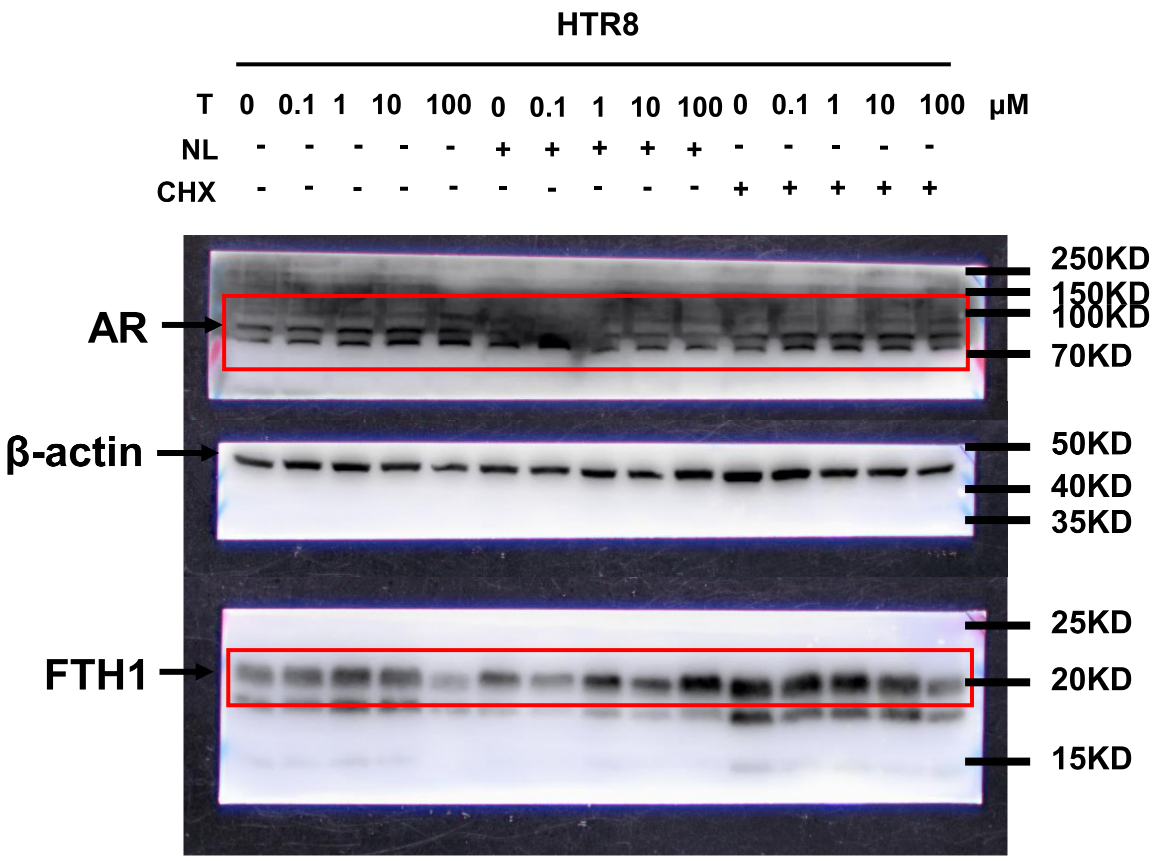


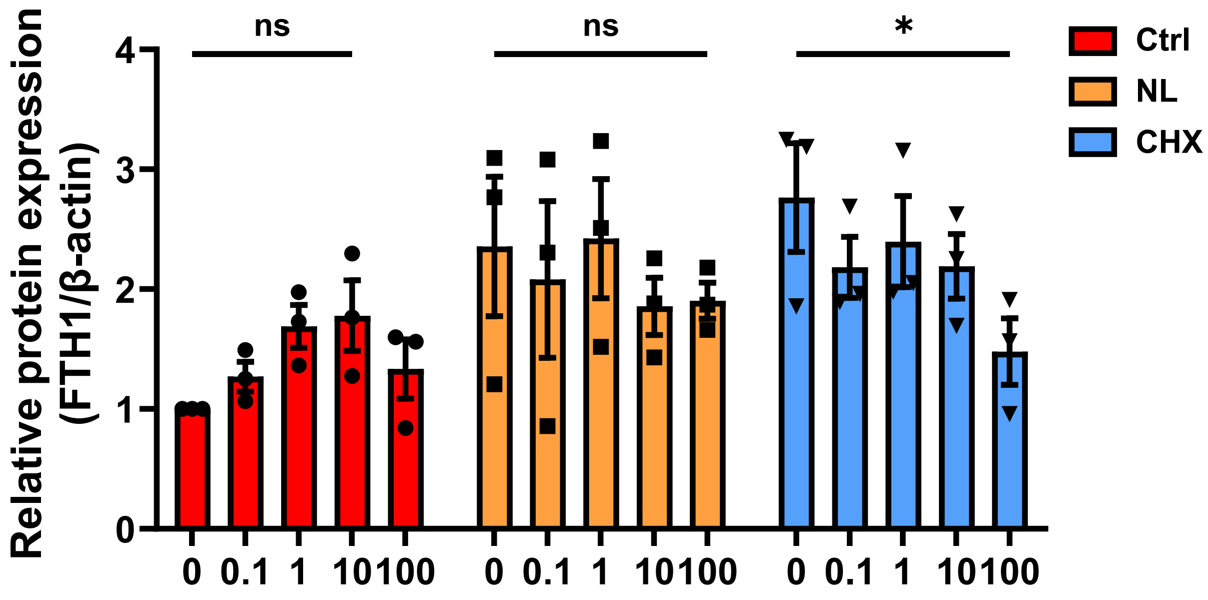

## Figure 2N：

### The first repeat:

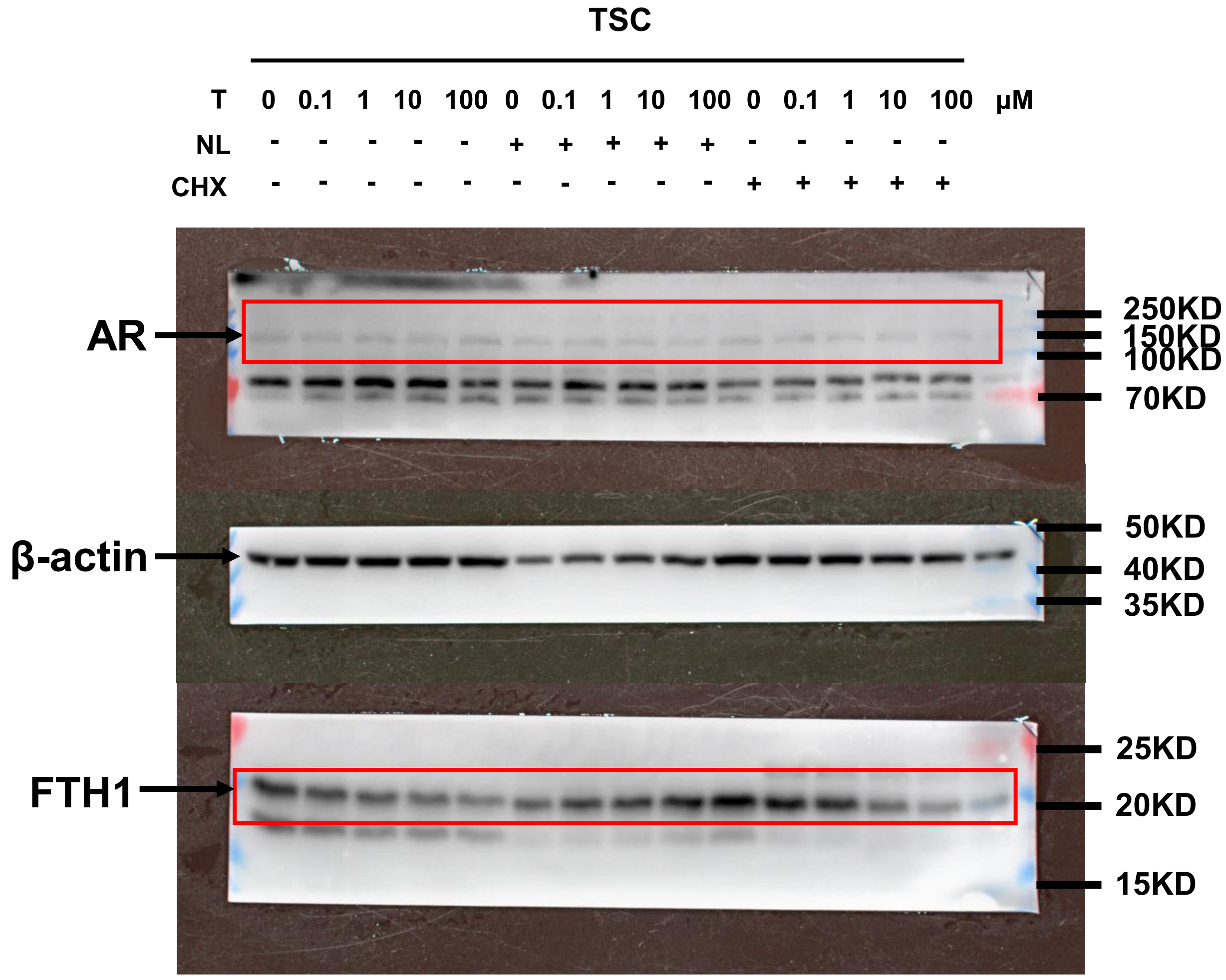


### The second repeat:

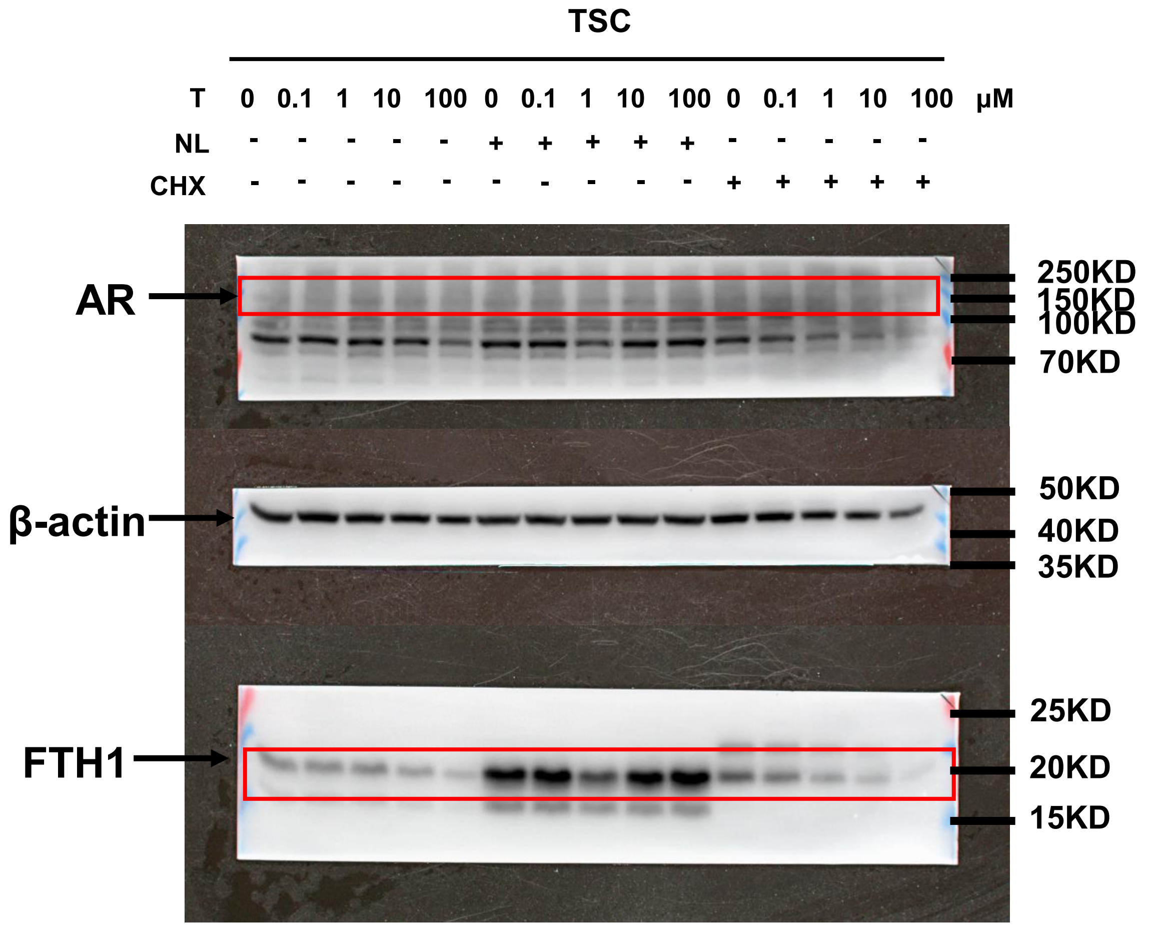


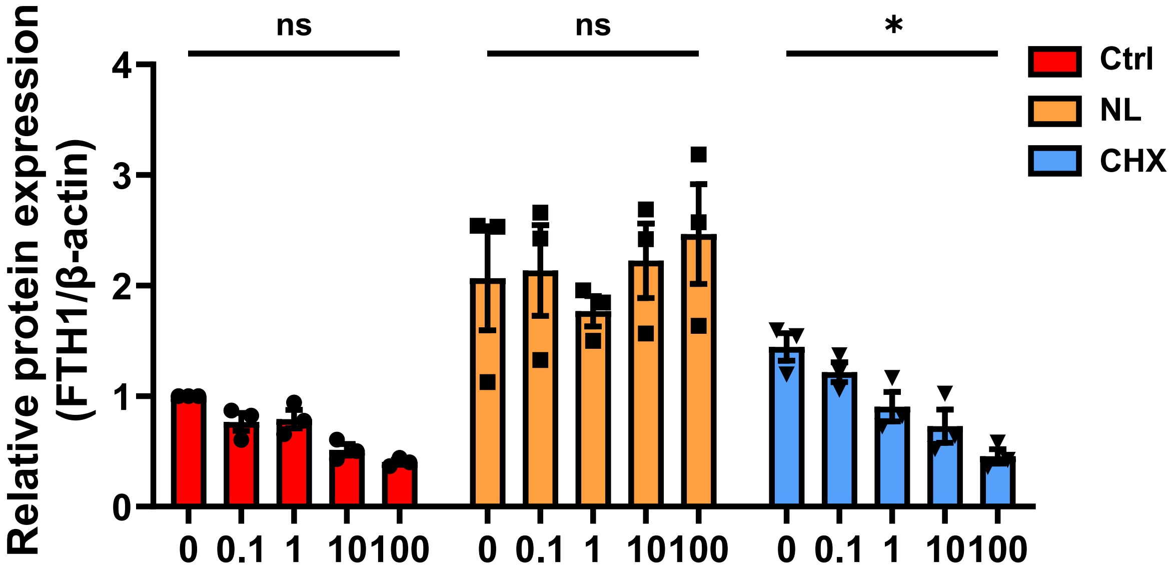


### The third repeat:

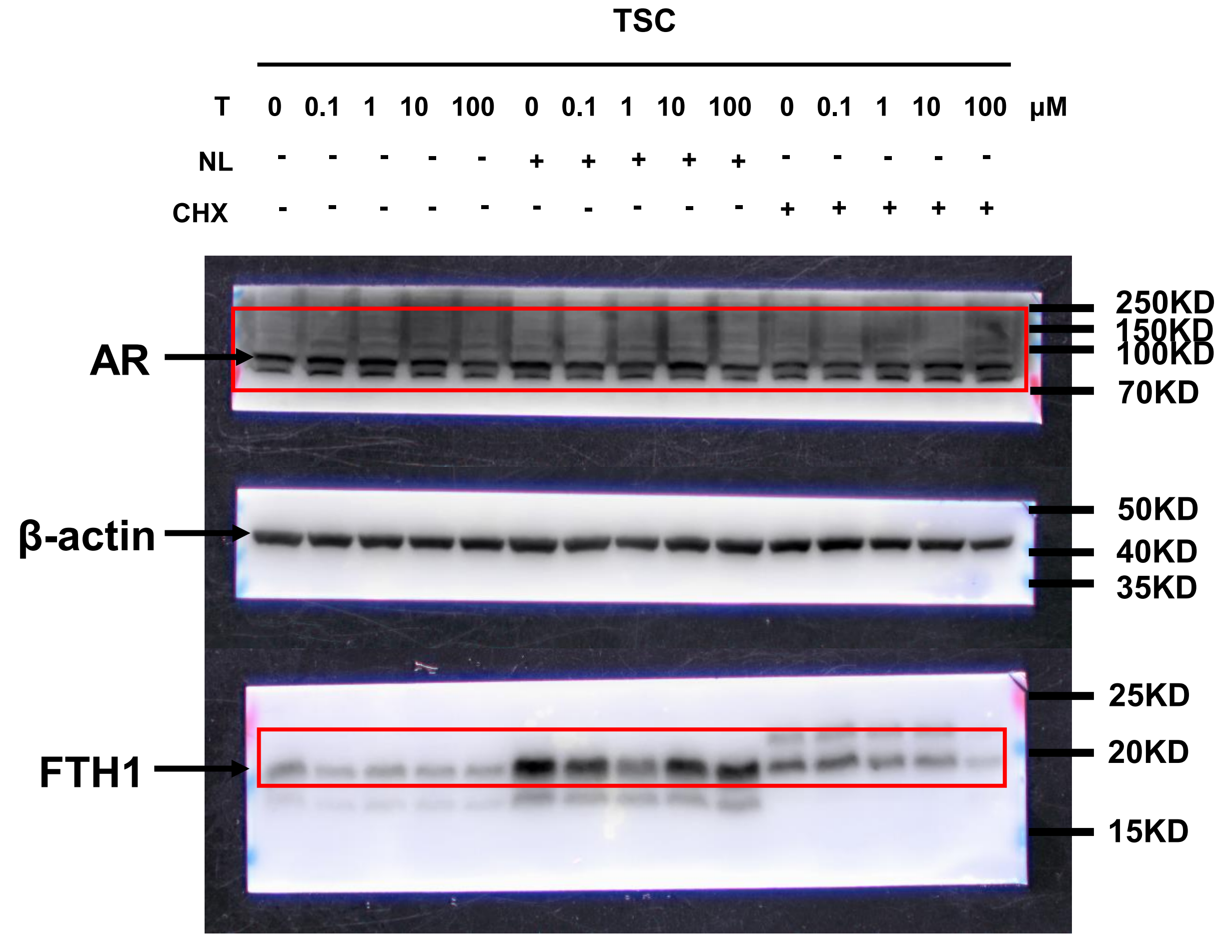

**Figure 3A:**

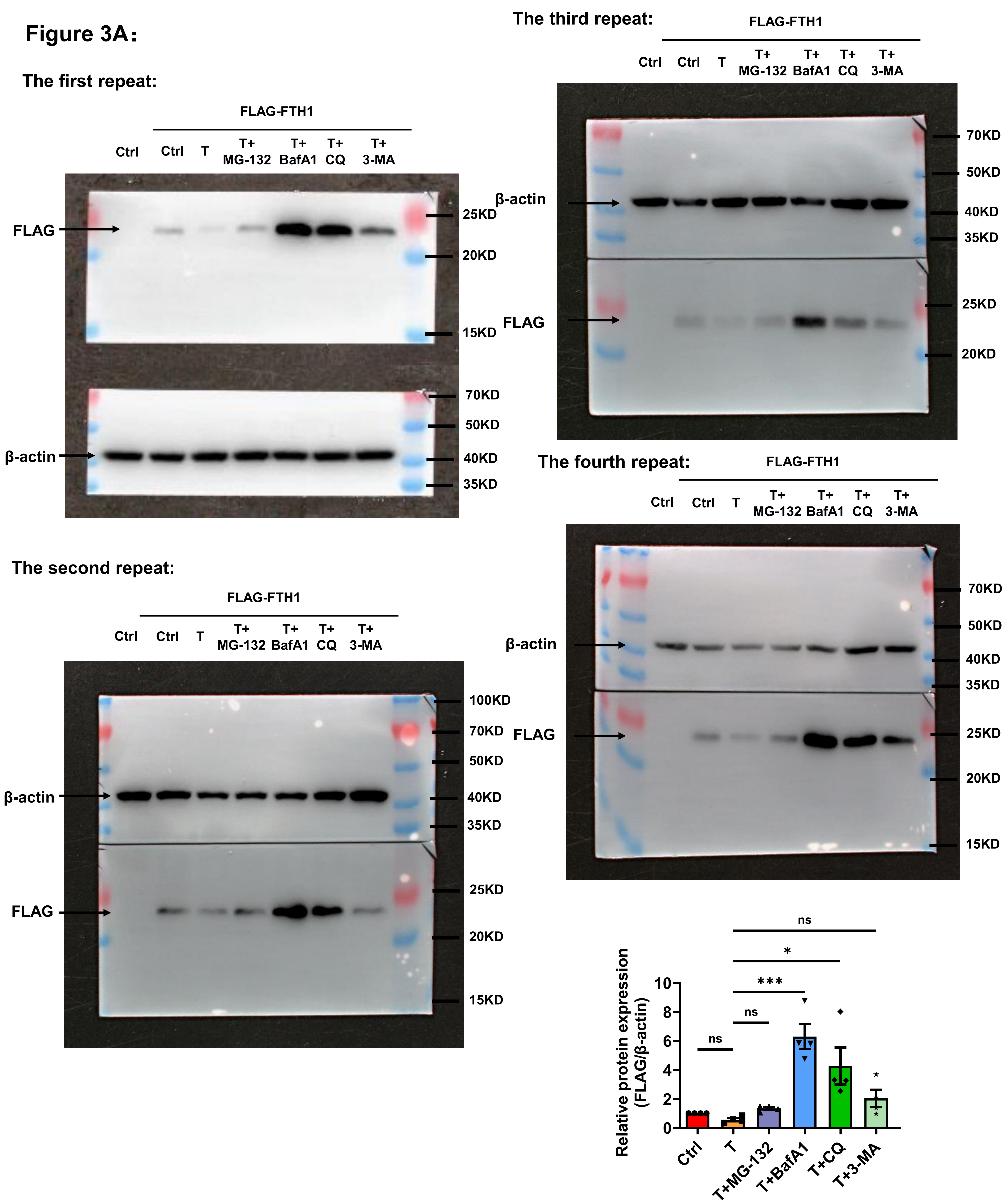
The first repeat:
FLAG-FTH1
Ctrl
Ctrl
T
T+ MG-132
T+ BafA1
T+ CQ
T+ 3-MA
FLAG
β-actin
25KD
20KD
15KD
70KD
50KD
40KD
35KD
The second repeat:
FLAG-FTH1
Ctrl
Ctrl
T
T+ MG-132
T+ BafA1
T+ CQ
T+ 3-MA
β-actin
FLAG
100KD
70KD
50KD
40KD
35KD
25KD
20KD
15KD
The third repeat:
FLAG-FTH1
Ctrl
Ctrl
T
T+ MG-132
T+ BafA1
T+ CQ
T+ 3-MA
β-actin
FLAG
70KD
50KD
40KD
35KD
25KD
20KD
The fourth repeat:
FLAG-FTH1
Ctrl
Ctrl
T
T+ MG-132
T+ BafA1
T+ CQ
T+ 3-MA
β-actin
FLAG
70KD
50KD
40KD
35KD
25KD
20KD
15KD
Relative protein expression (FLAG/β-actin)
10
8
6
4
2
0
ns
ns
***
*
ns
Ctrl
T
T+MG-132
T+BafA1
T+CQ
T+3-MA

**Figure 3B：**

The first repeat:

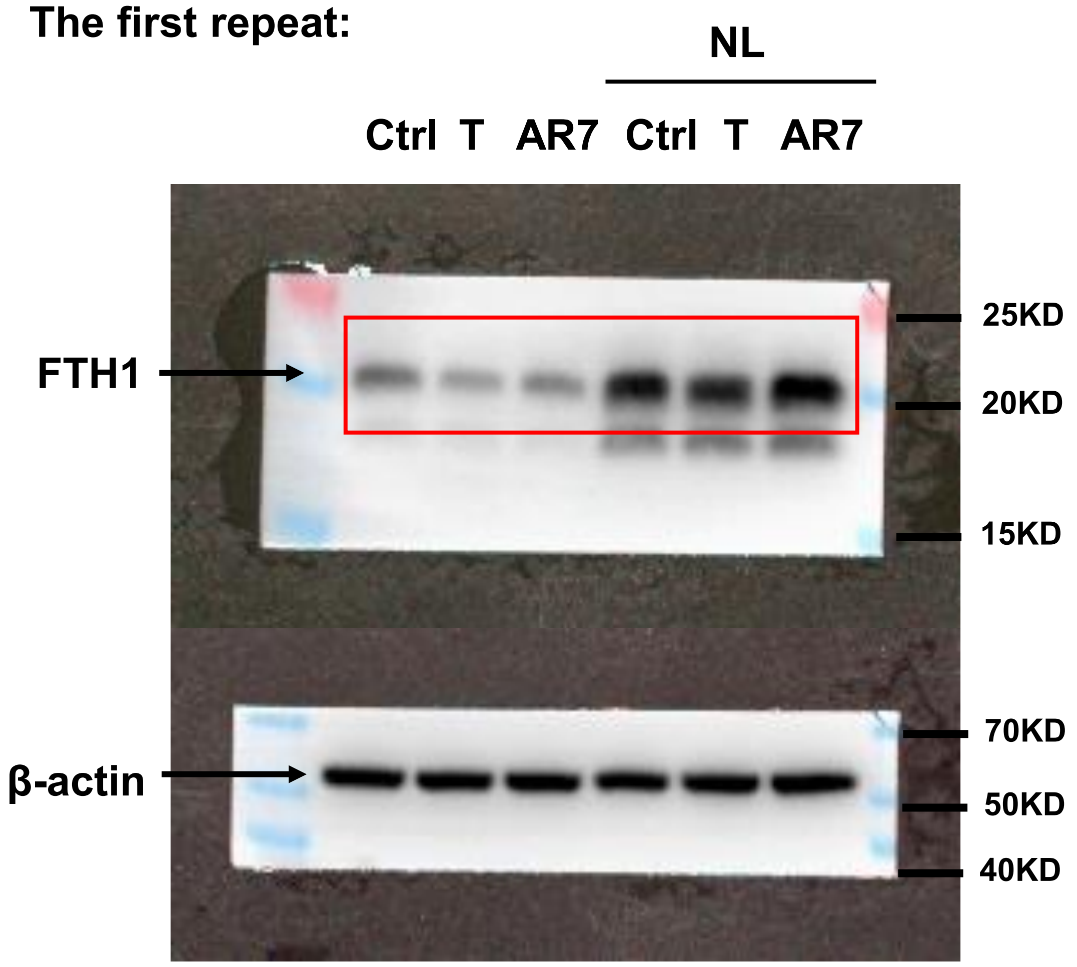


The third repeat:

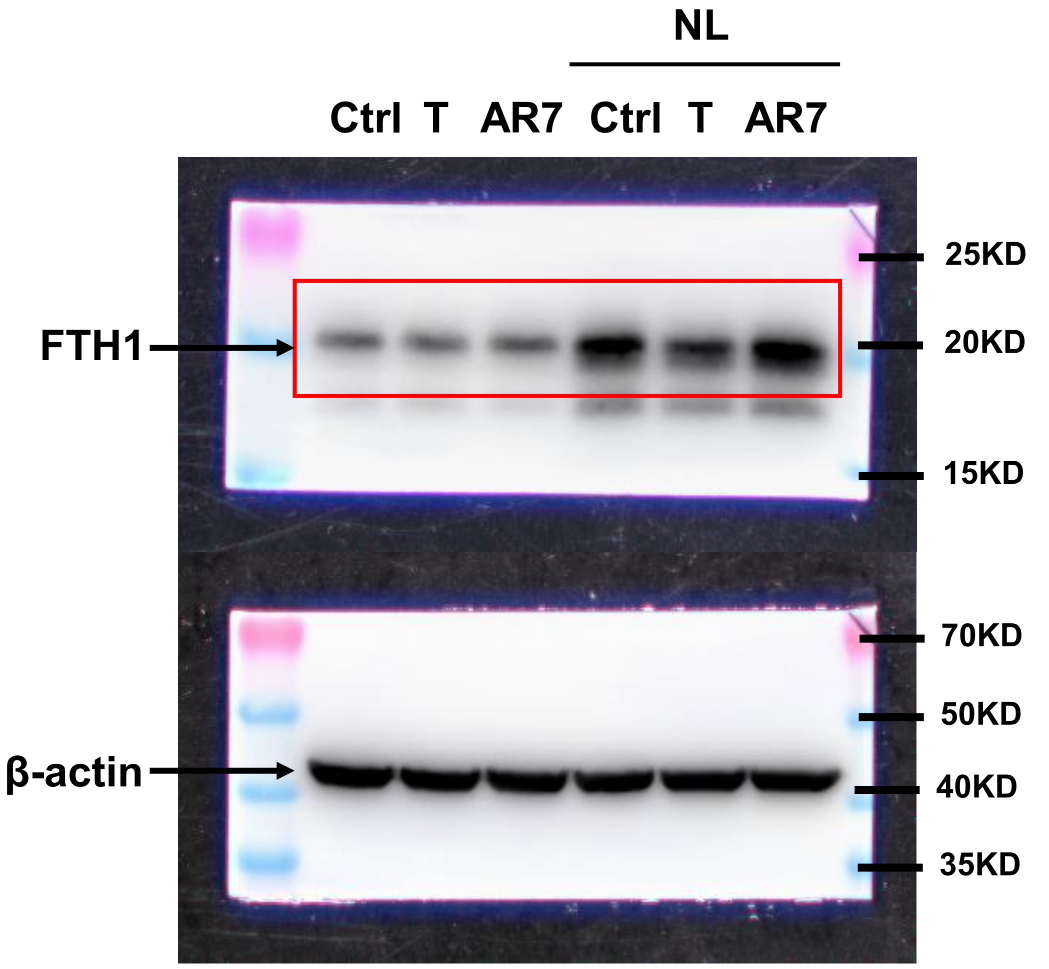


The second repeat:

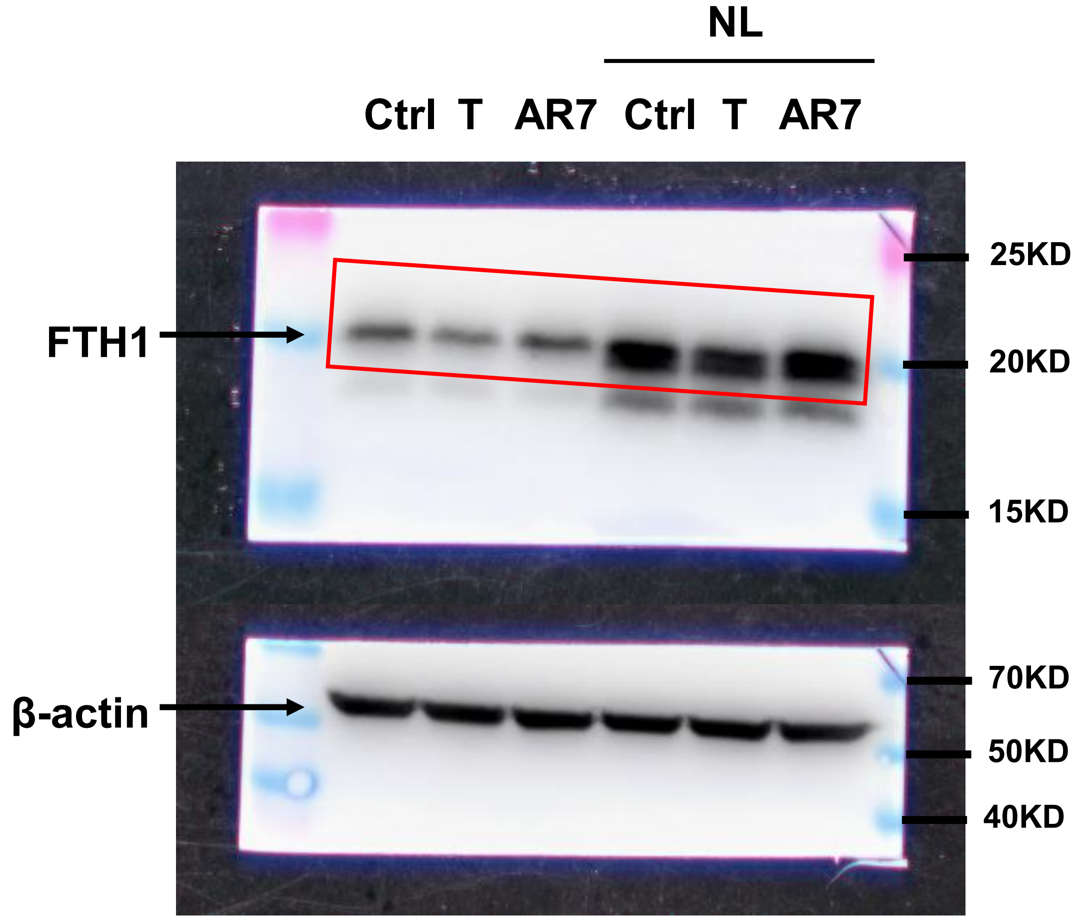


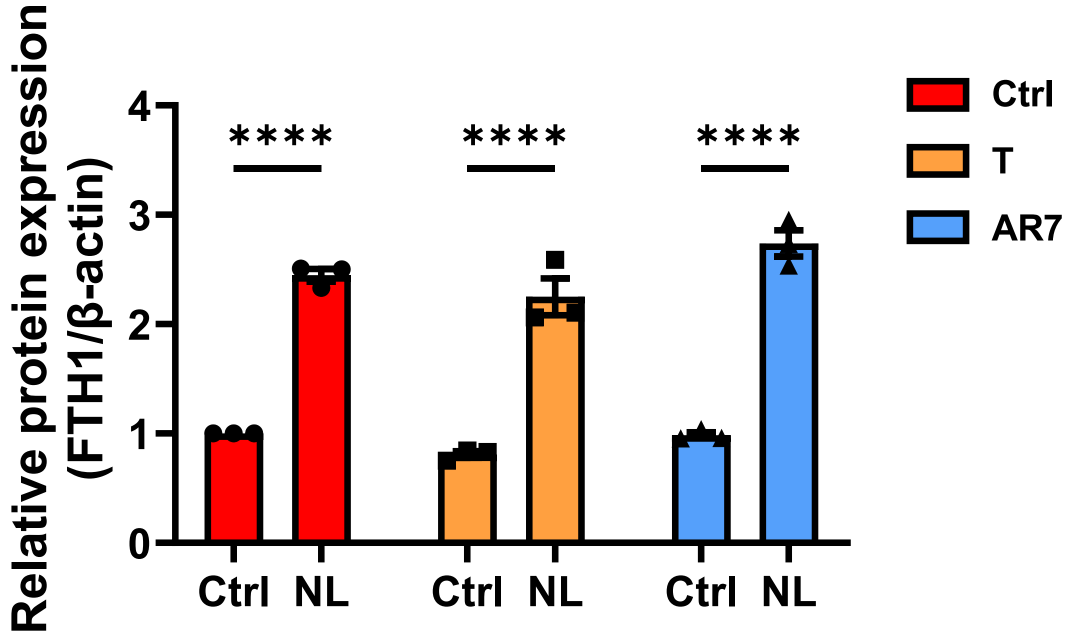

**Figure 3E：**

The first repeat:

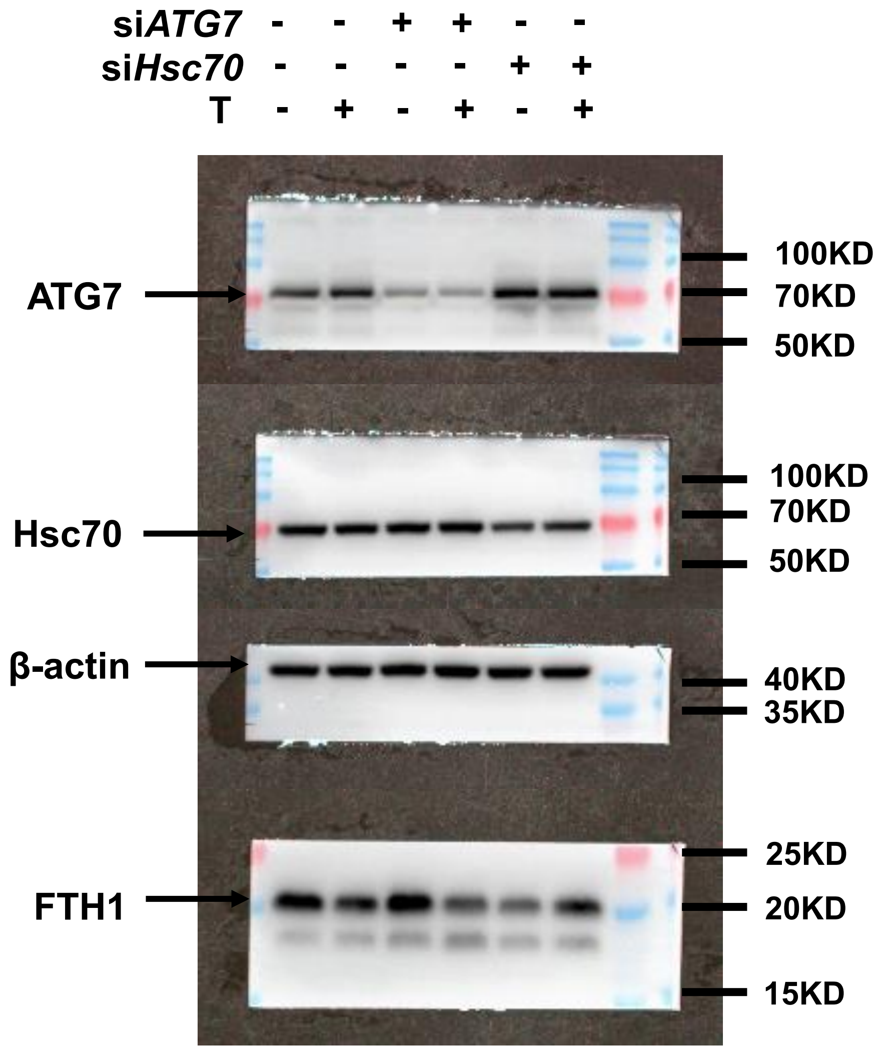


The third repeat:

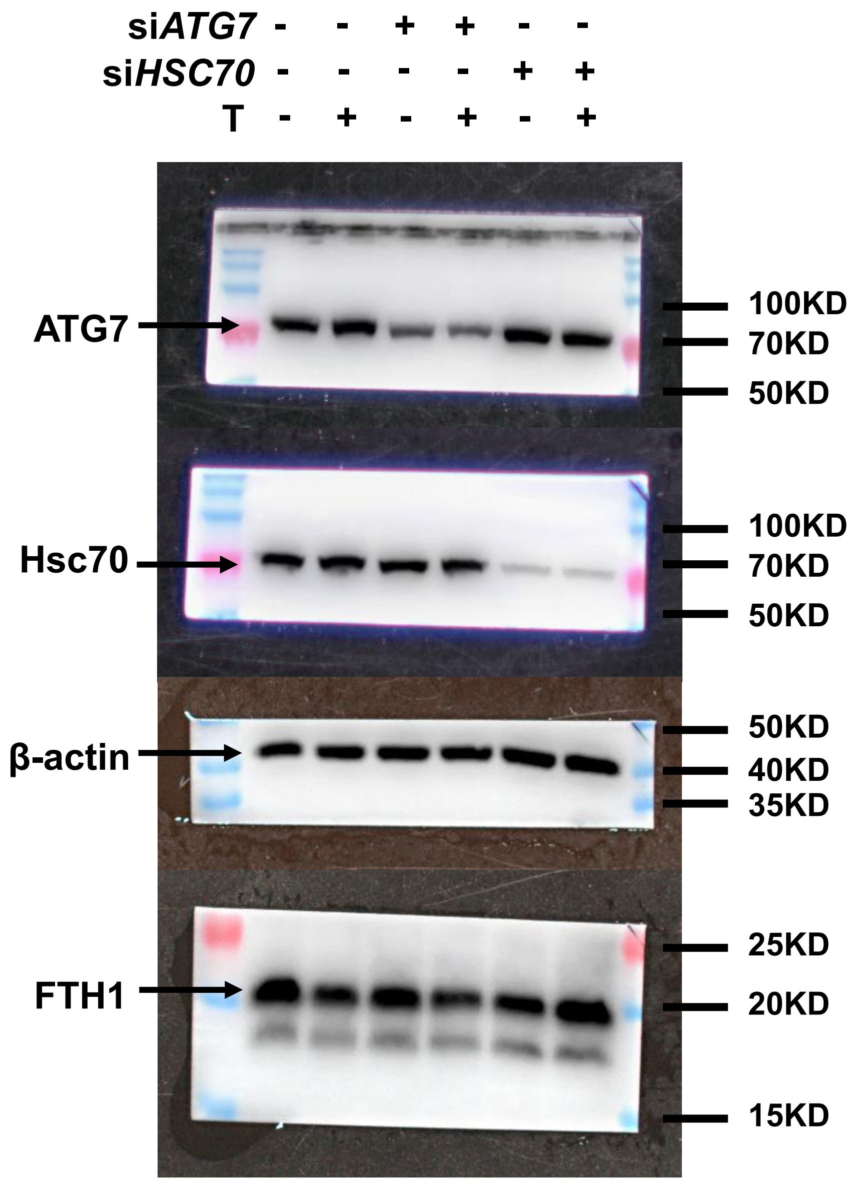


The second repeat:

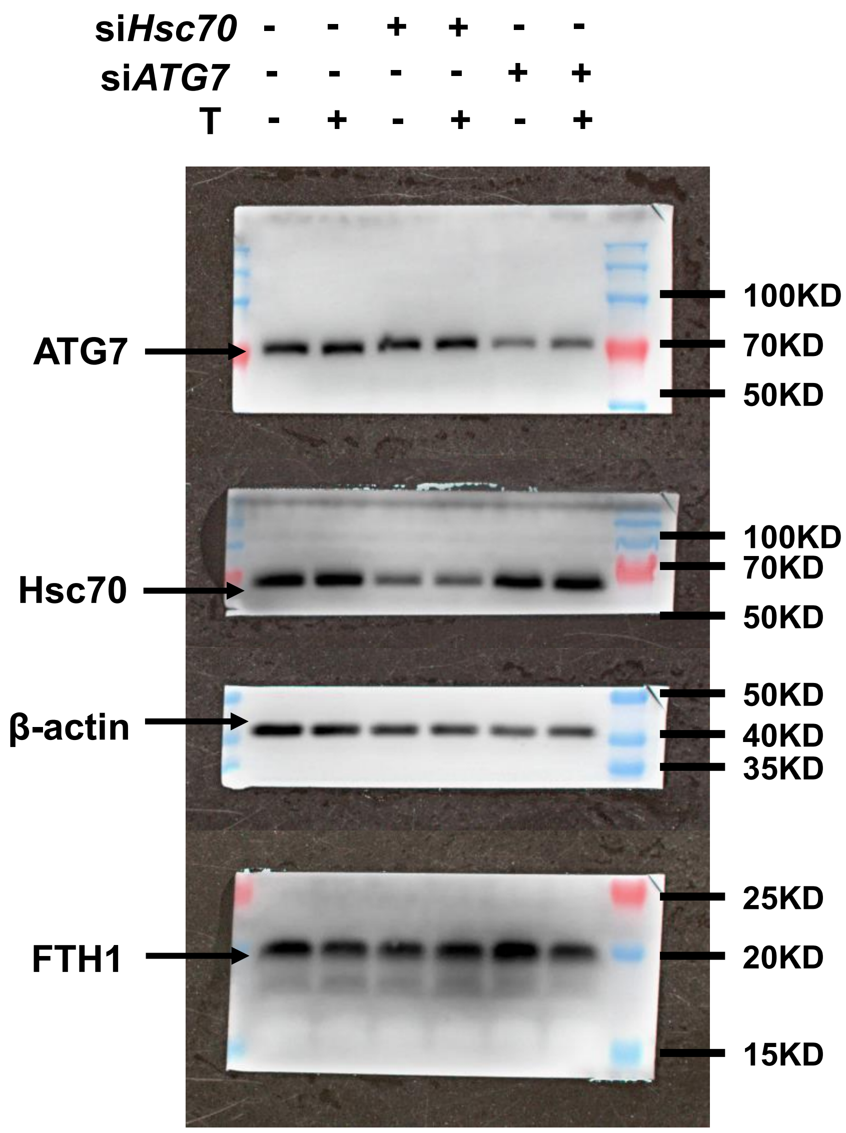


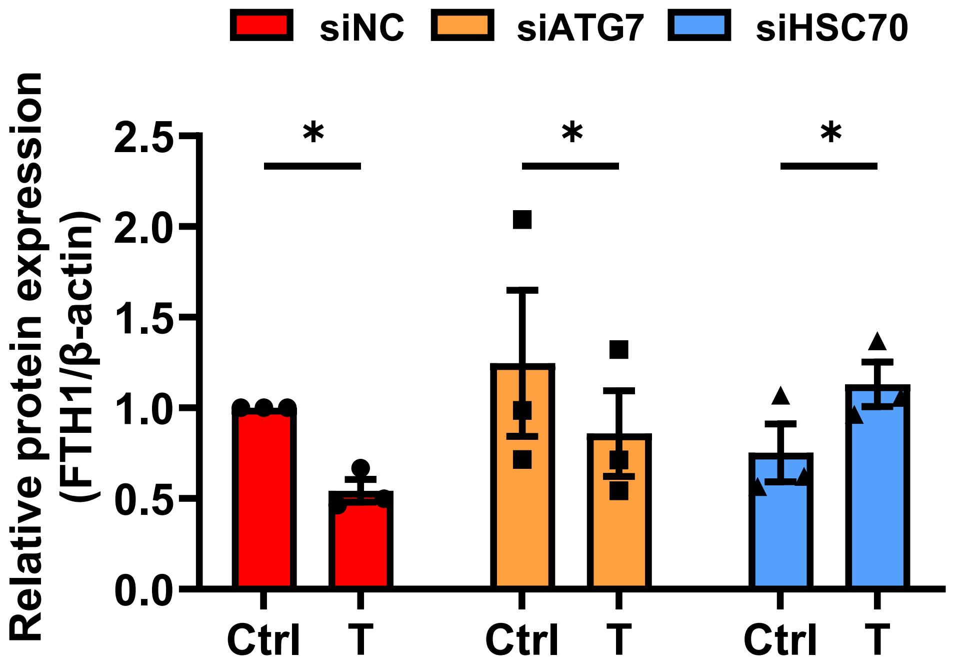

**Figure 3H：**

**The first repeat:**

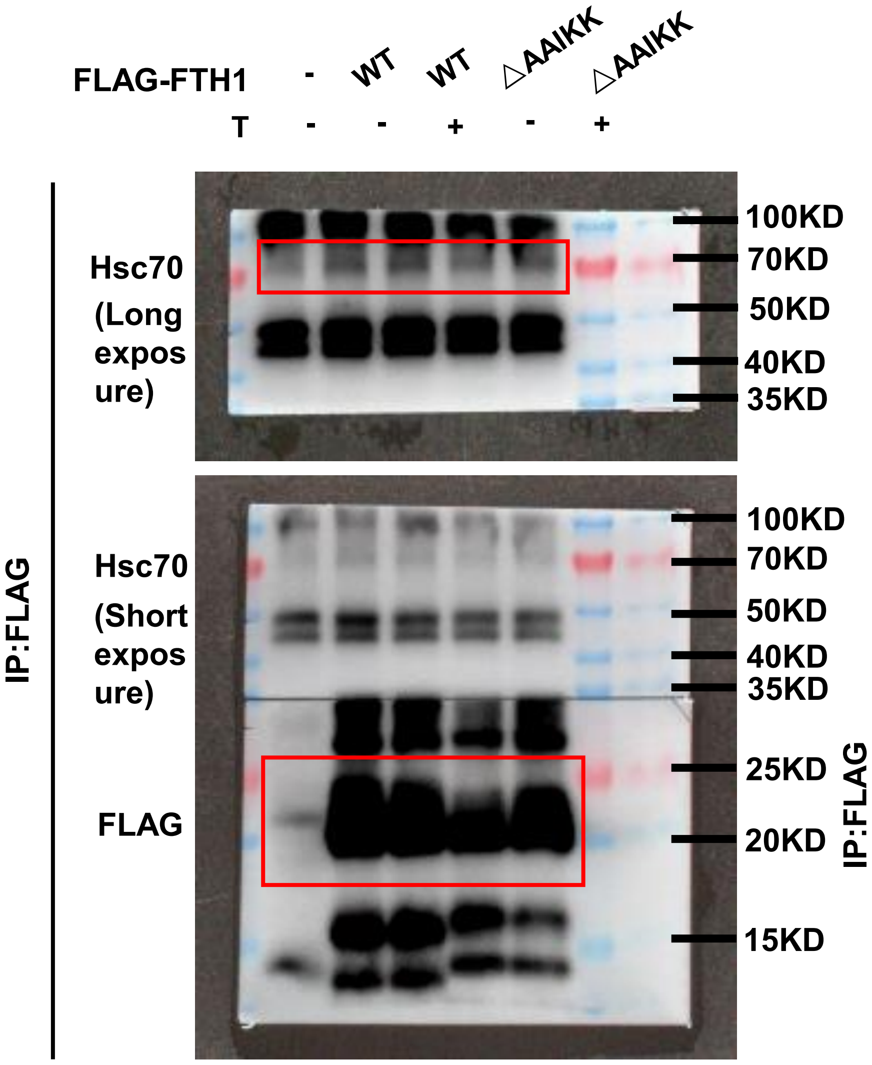


**The second repeat:**

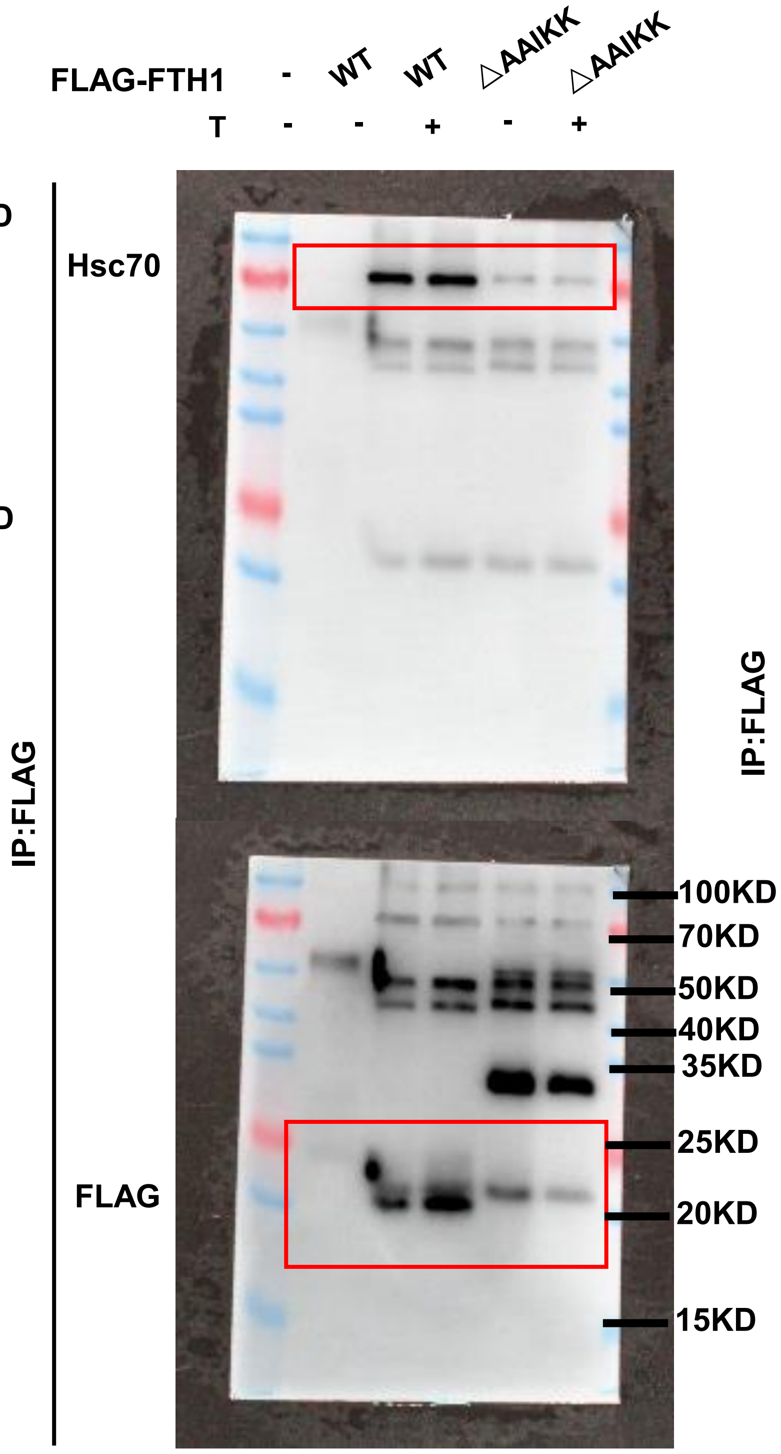


**The third repeat:**

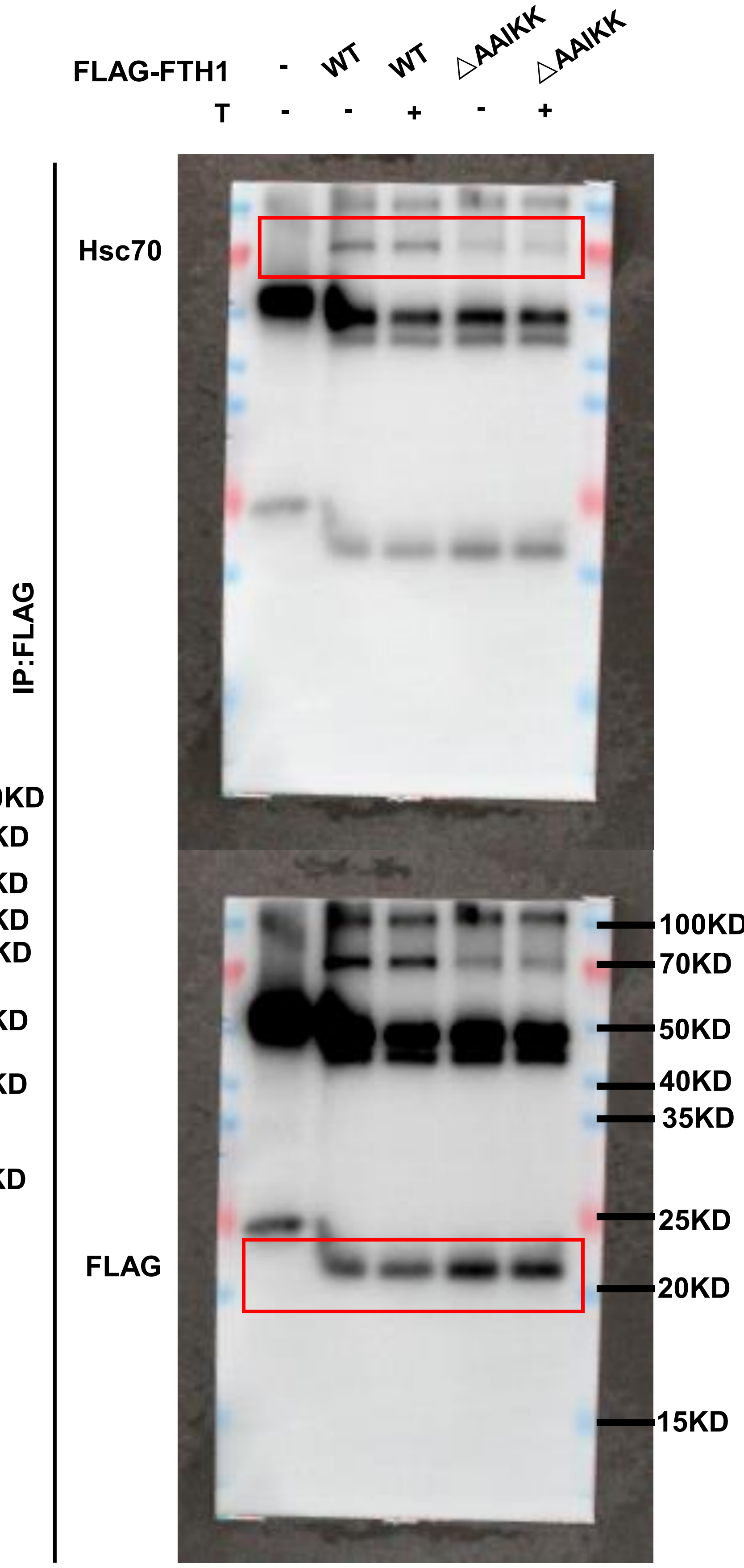


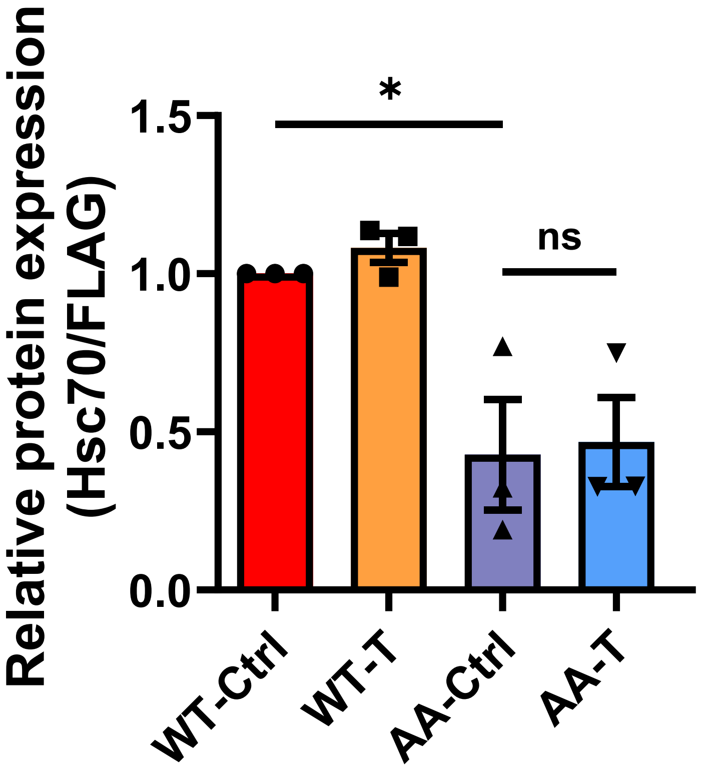

## Figure 3J:

The first repeat:

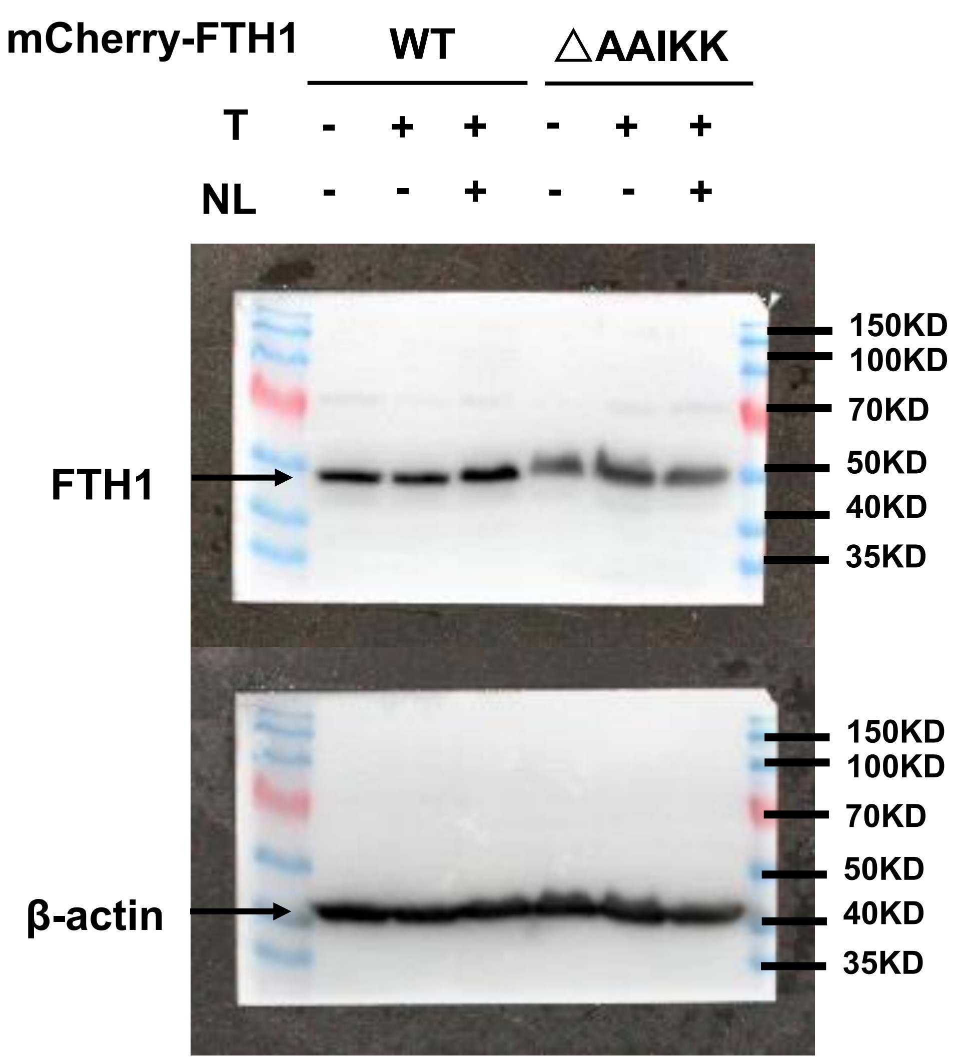


The second repeat:

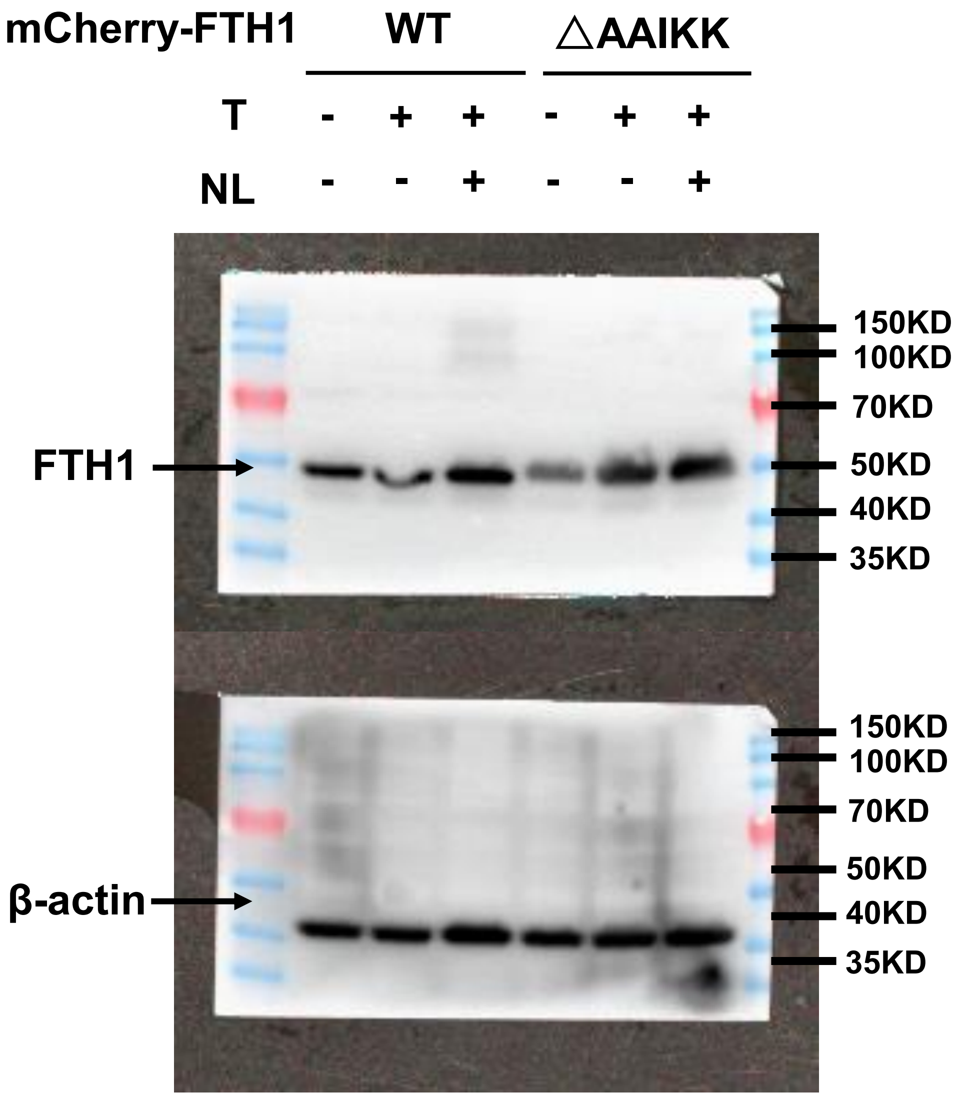


The third repeat:

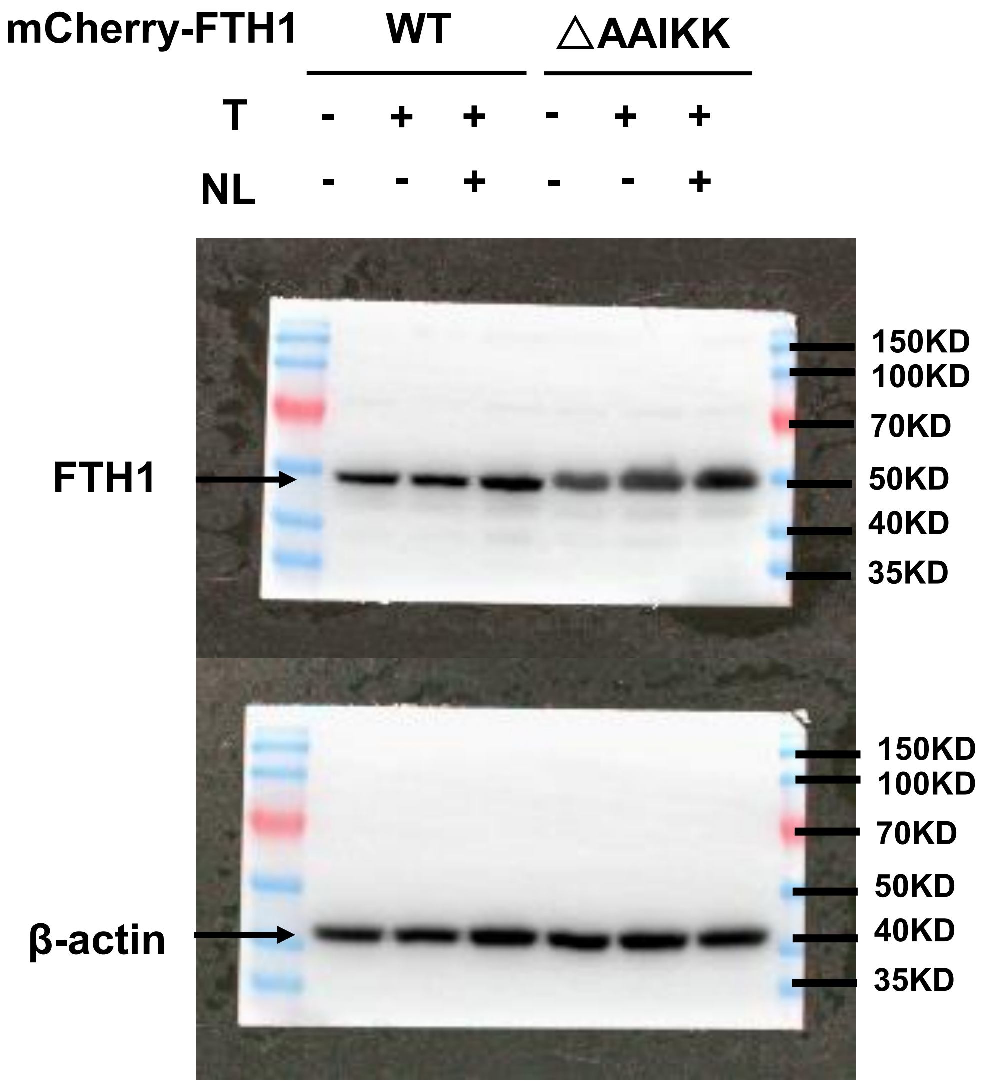


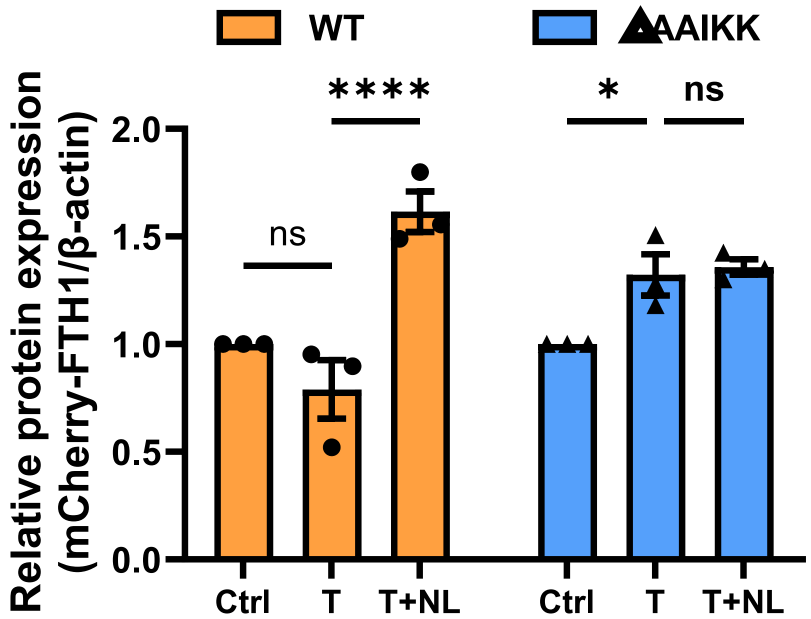

**Figure 3M：**

**The first repeat:**

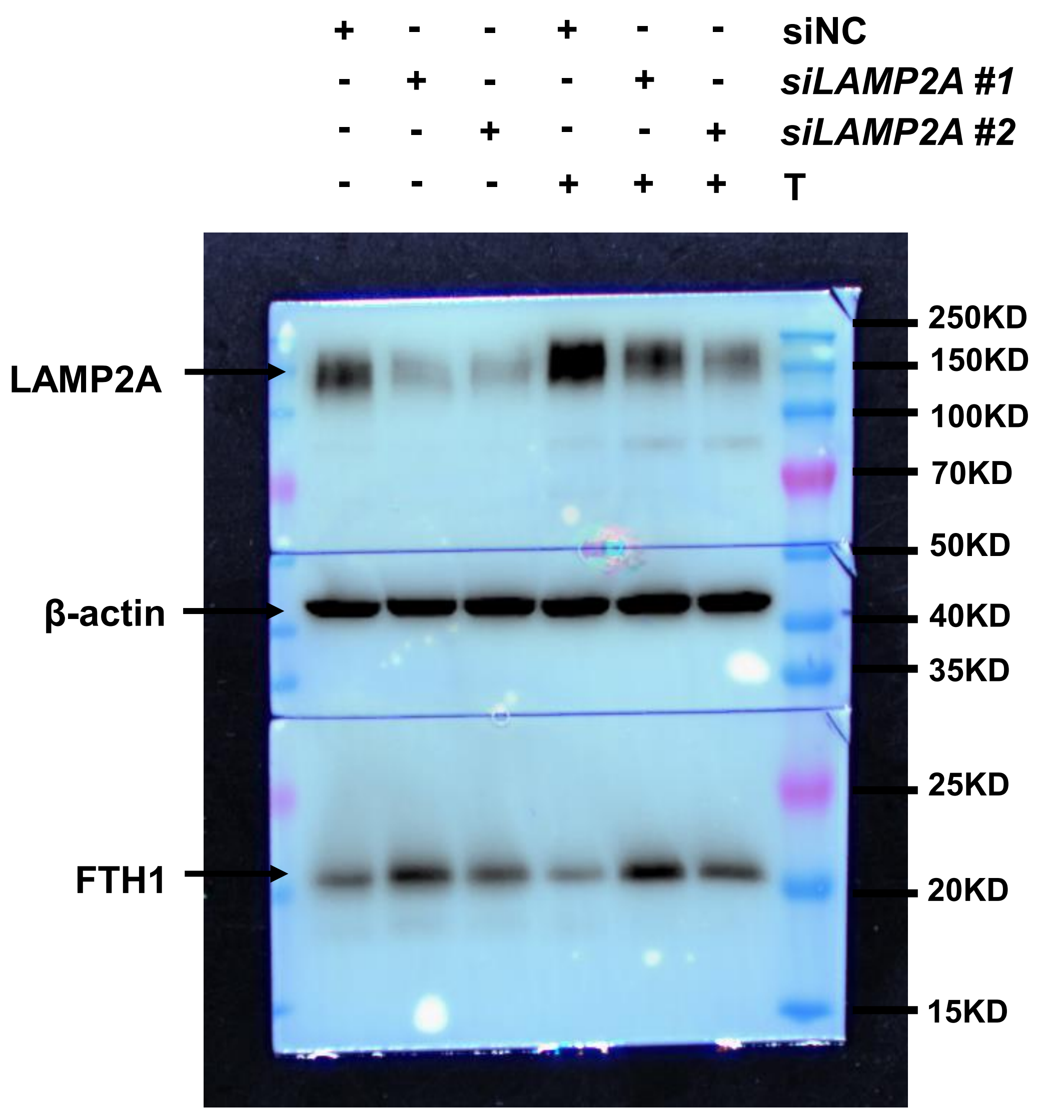


**The second repeat:**

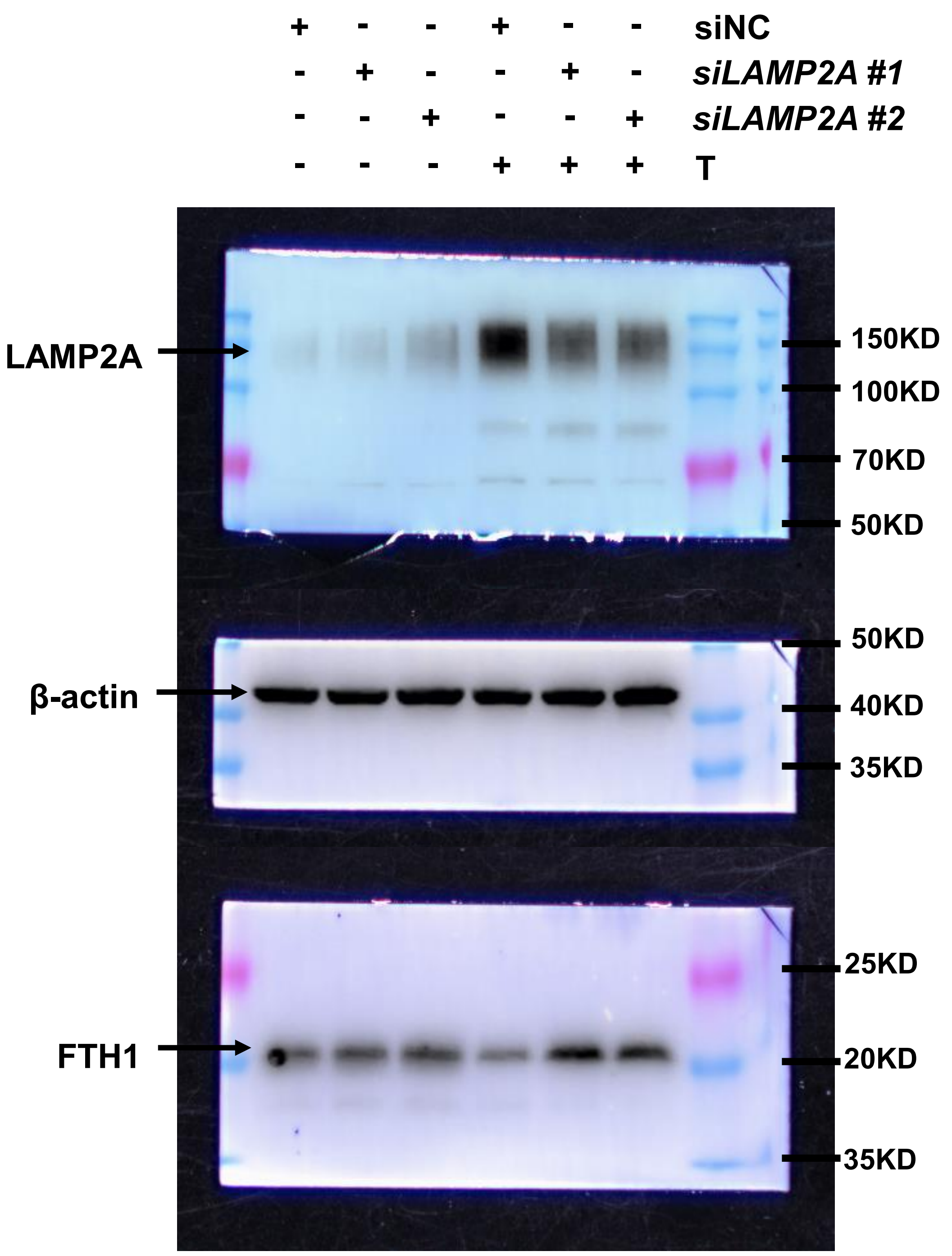


**The third repeat:**

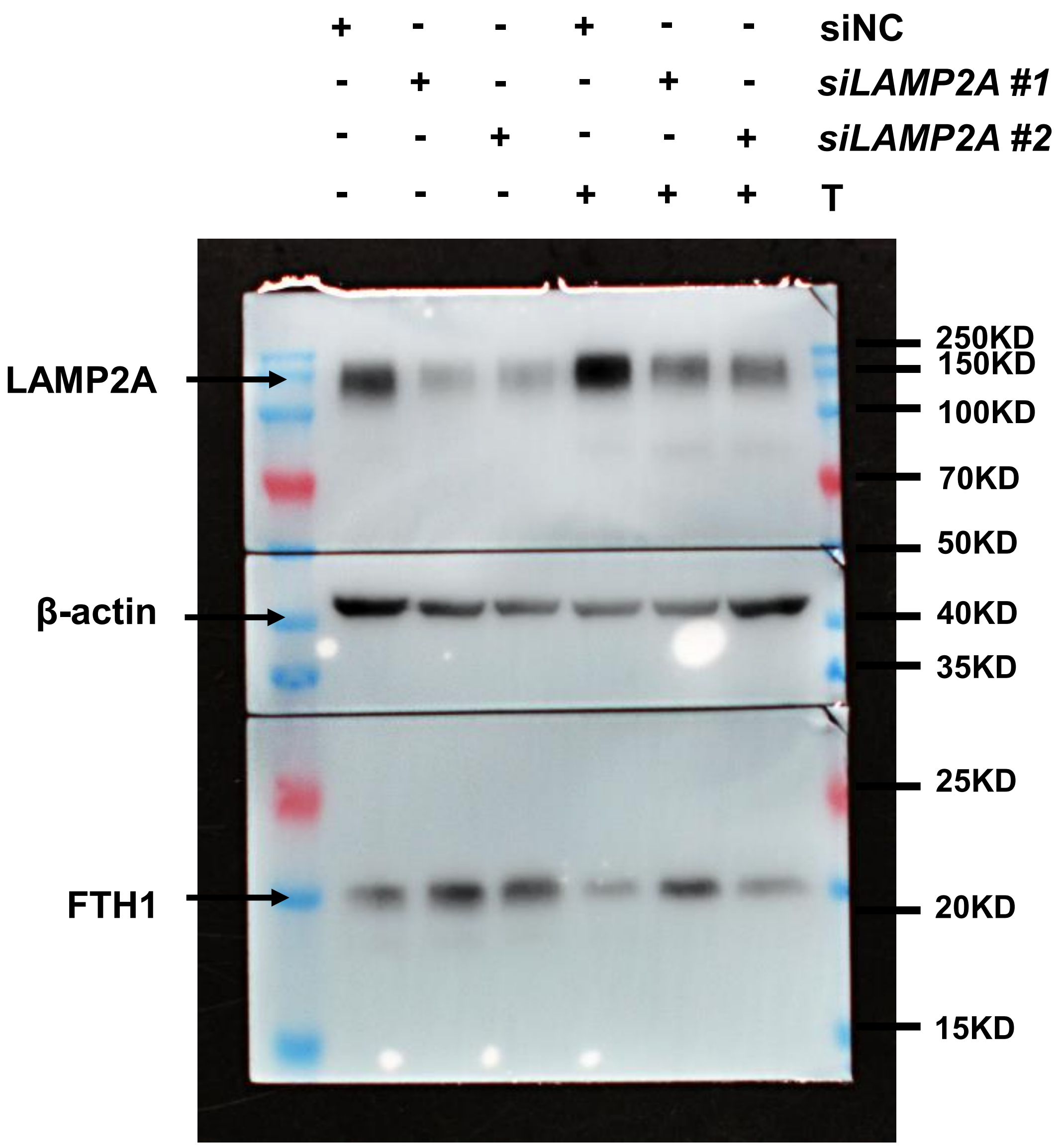


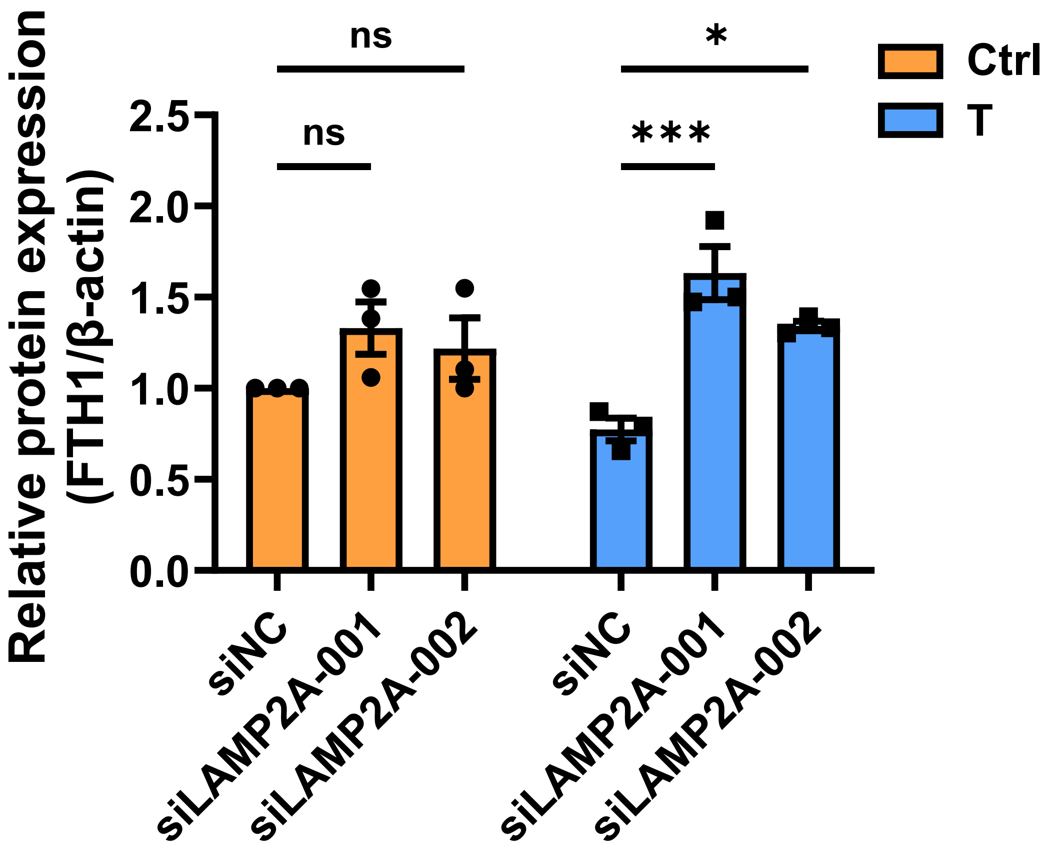

## Figure 4B：

### The first repeat:

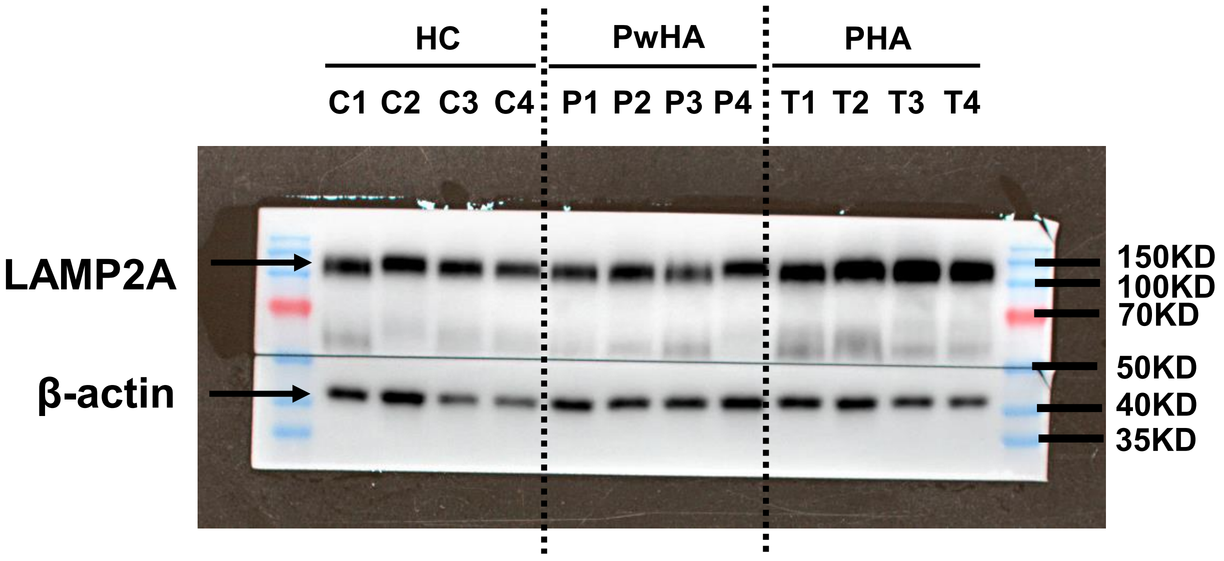


### The second repeat:

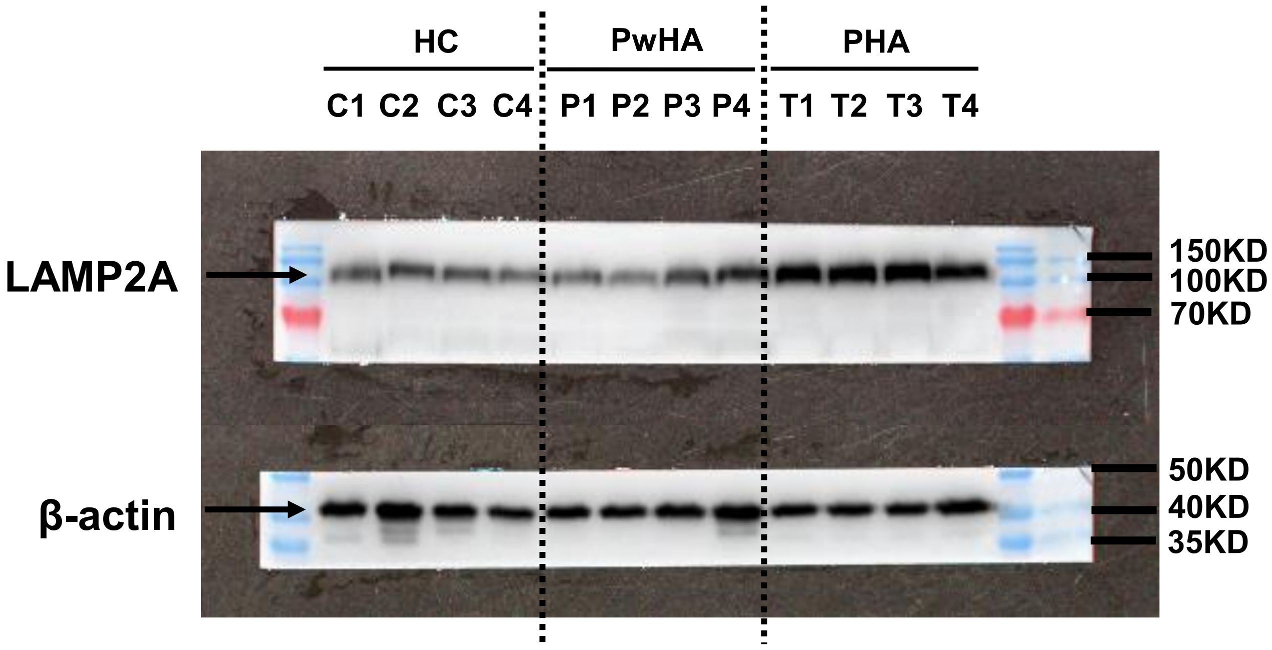


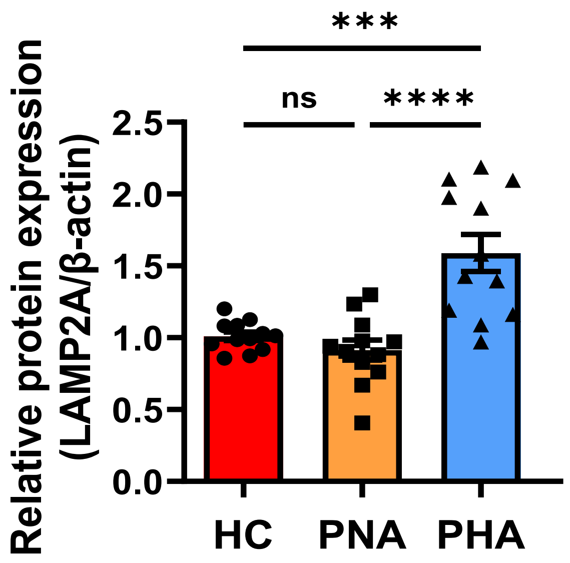


### The third repeat:

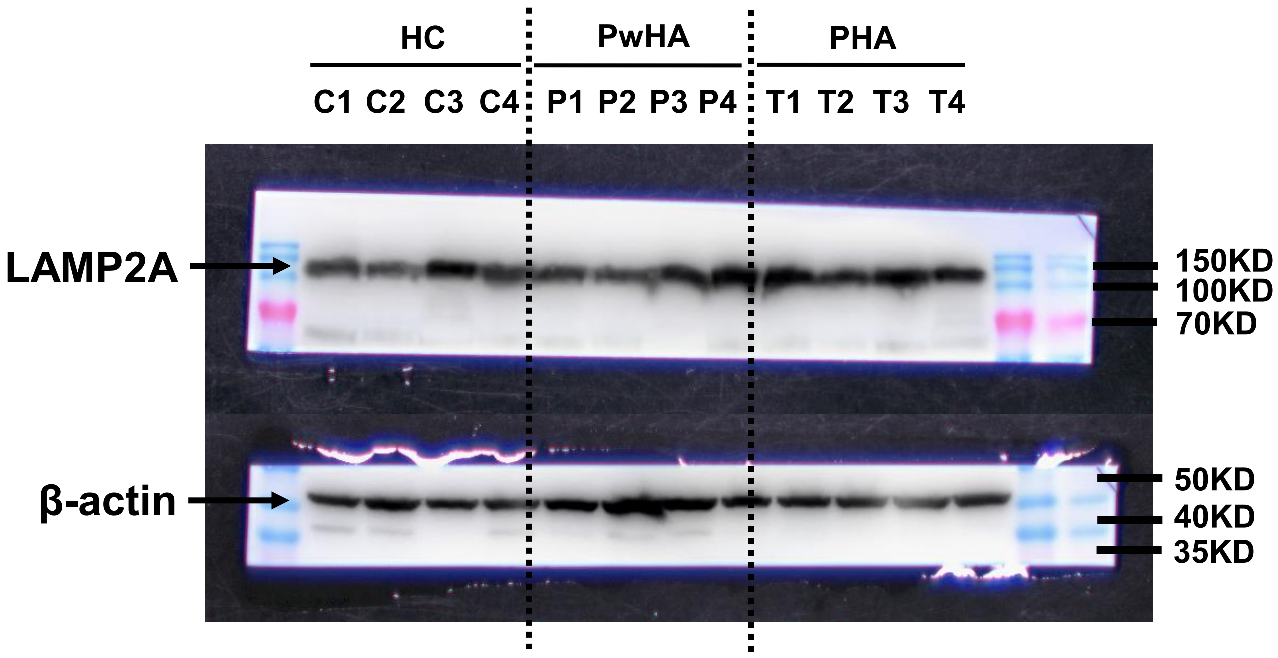


PS: The second repeat in Figure 4B and the first repeat in Figure 2A were derived from the same experiment, the WB membrane was washed with the stripping buffer.

## Figure 4E:

The first repeat:

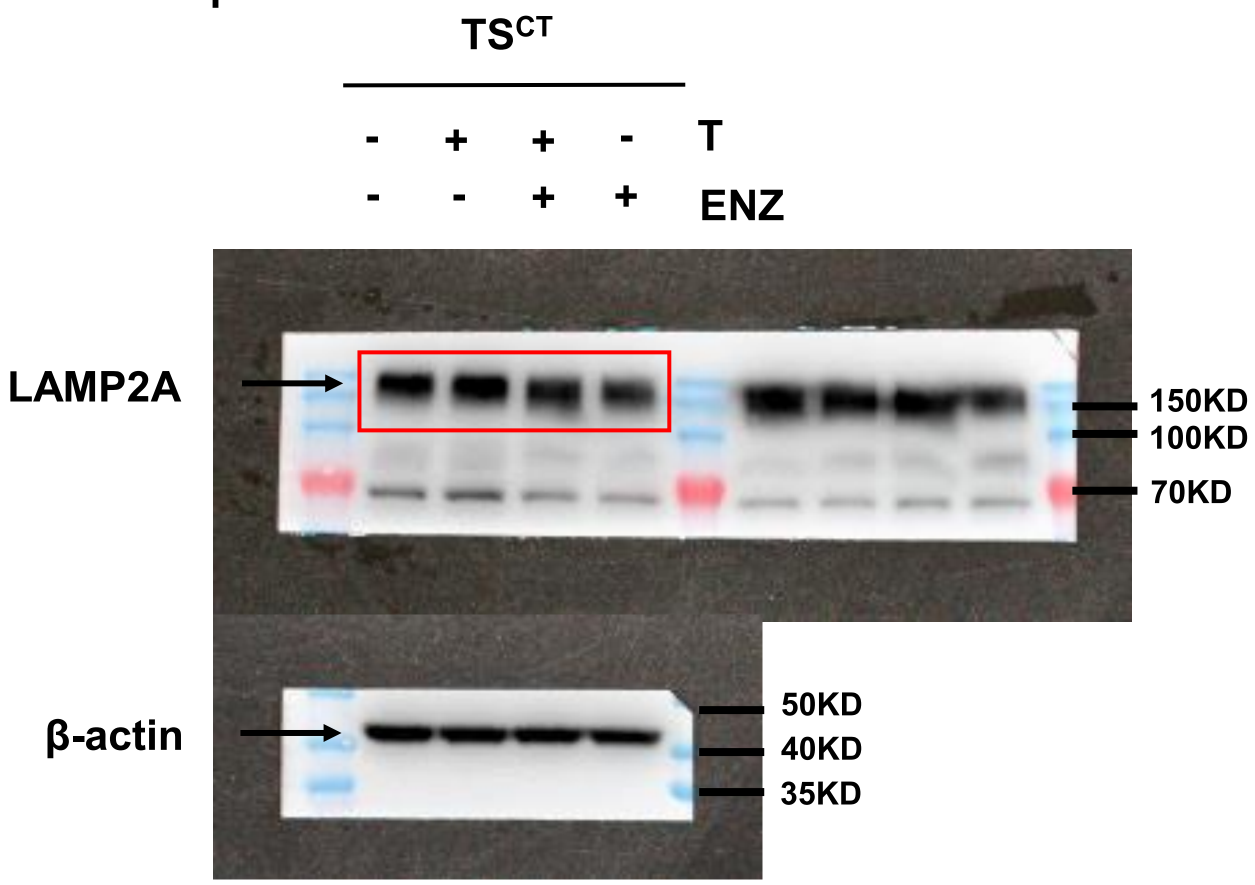


The second repeat:

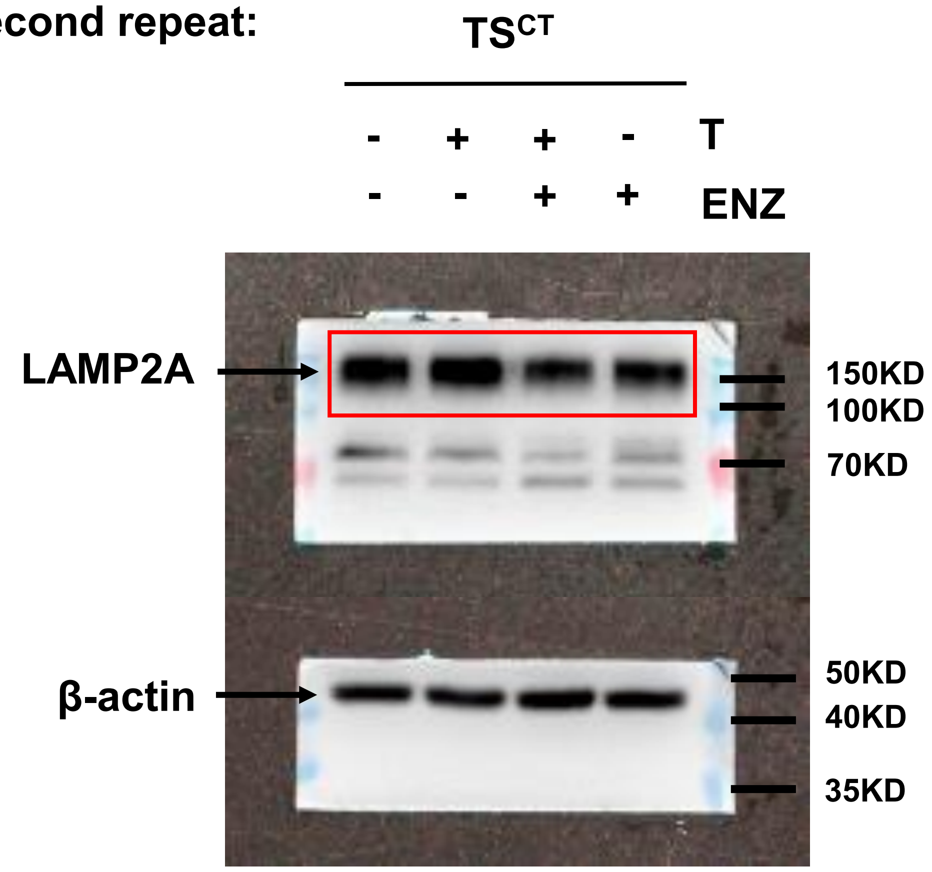


The third repeat:

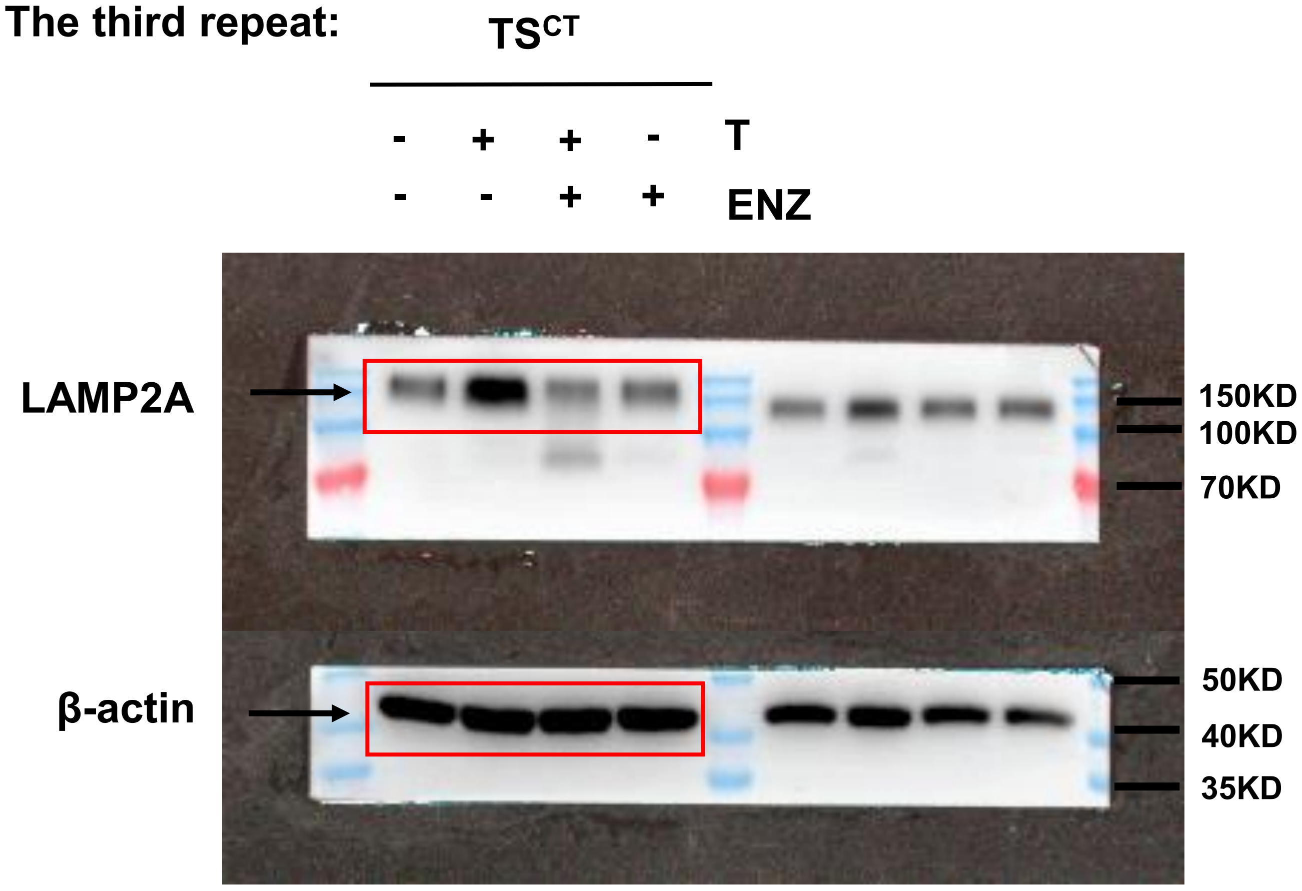


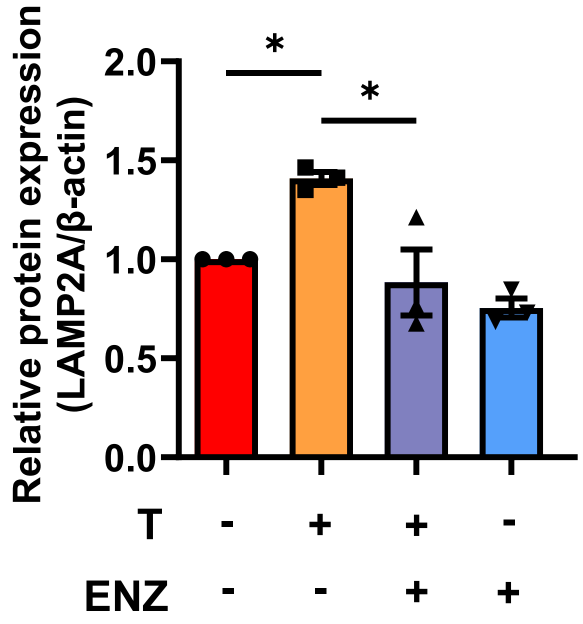

**Figure 4K:**

**The first repeat:**

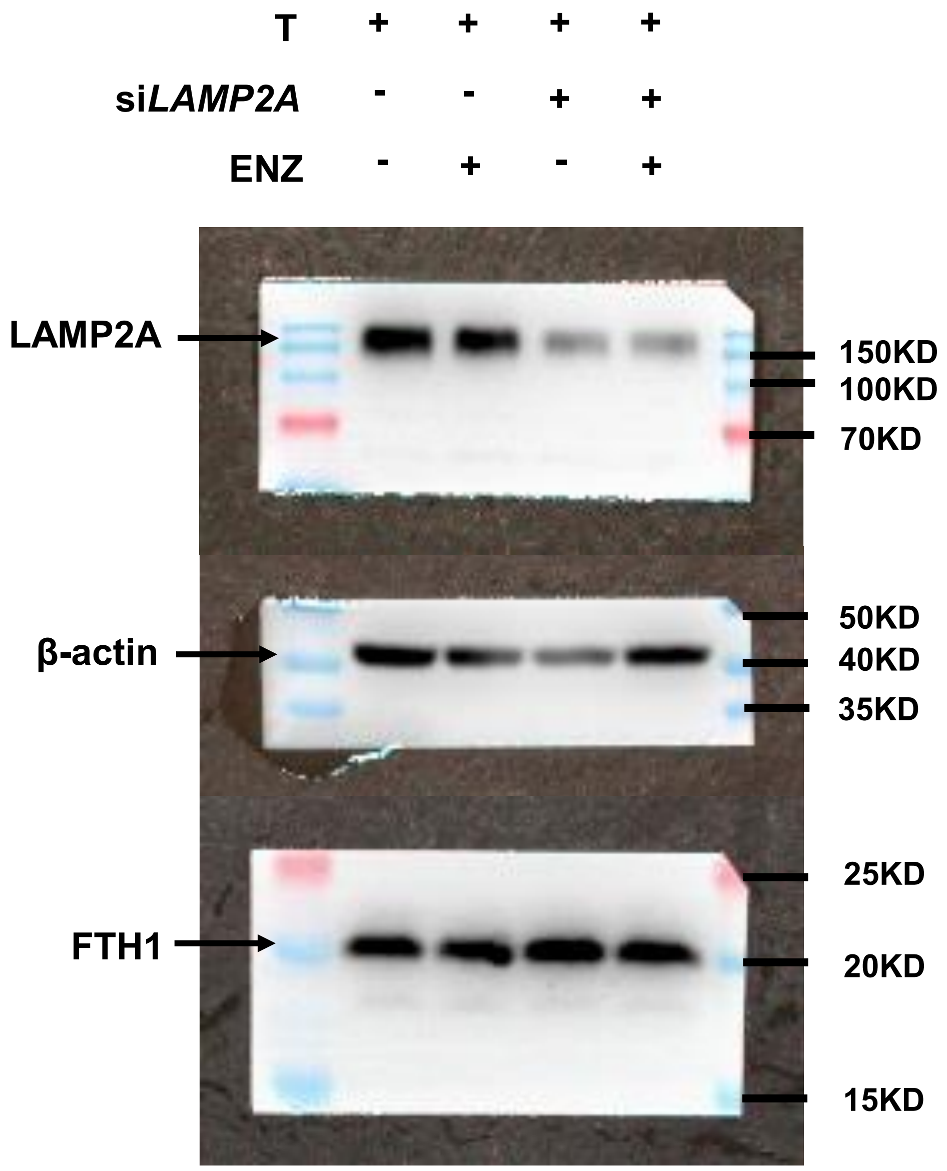


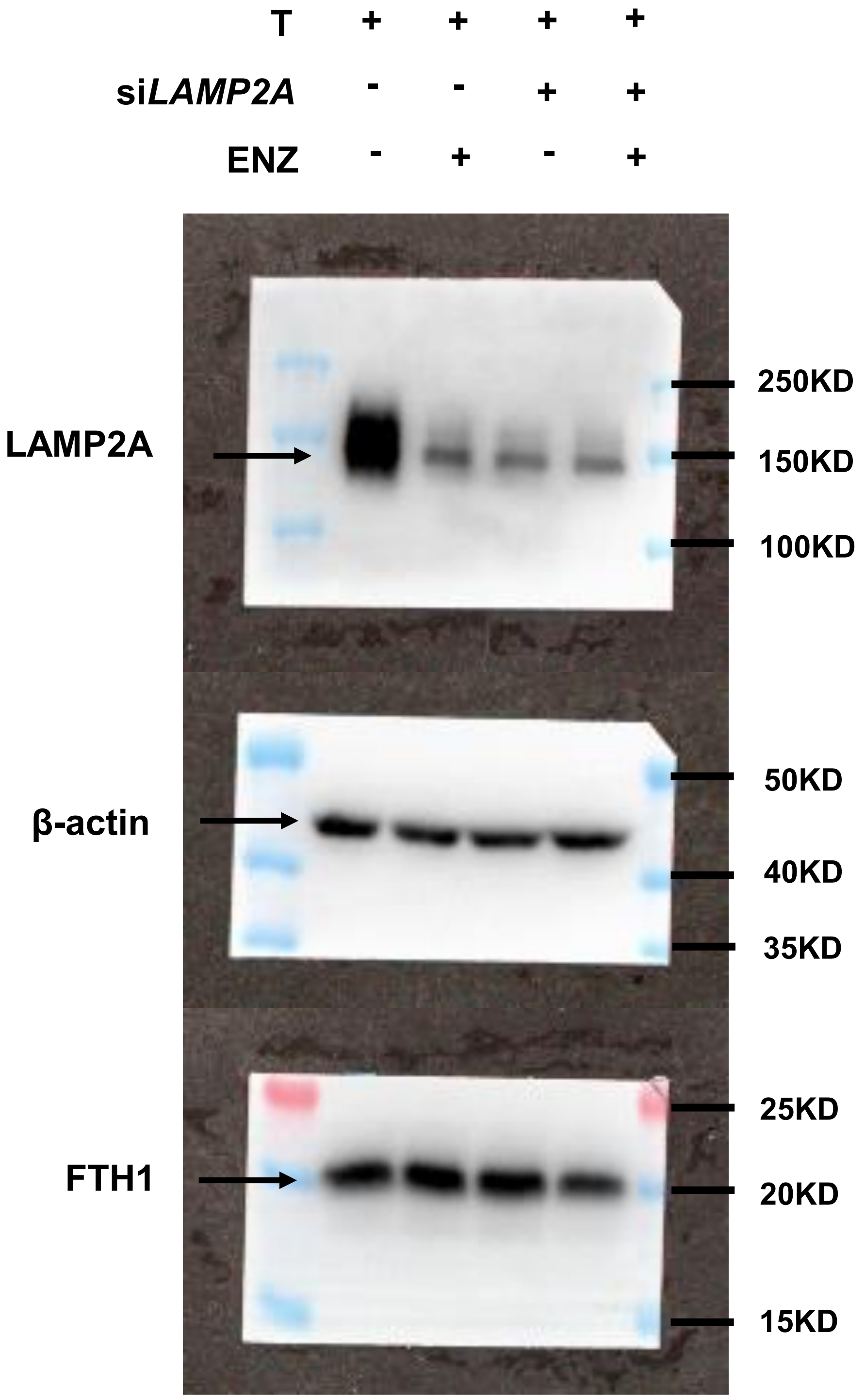


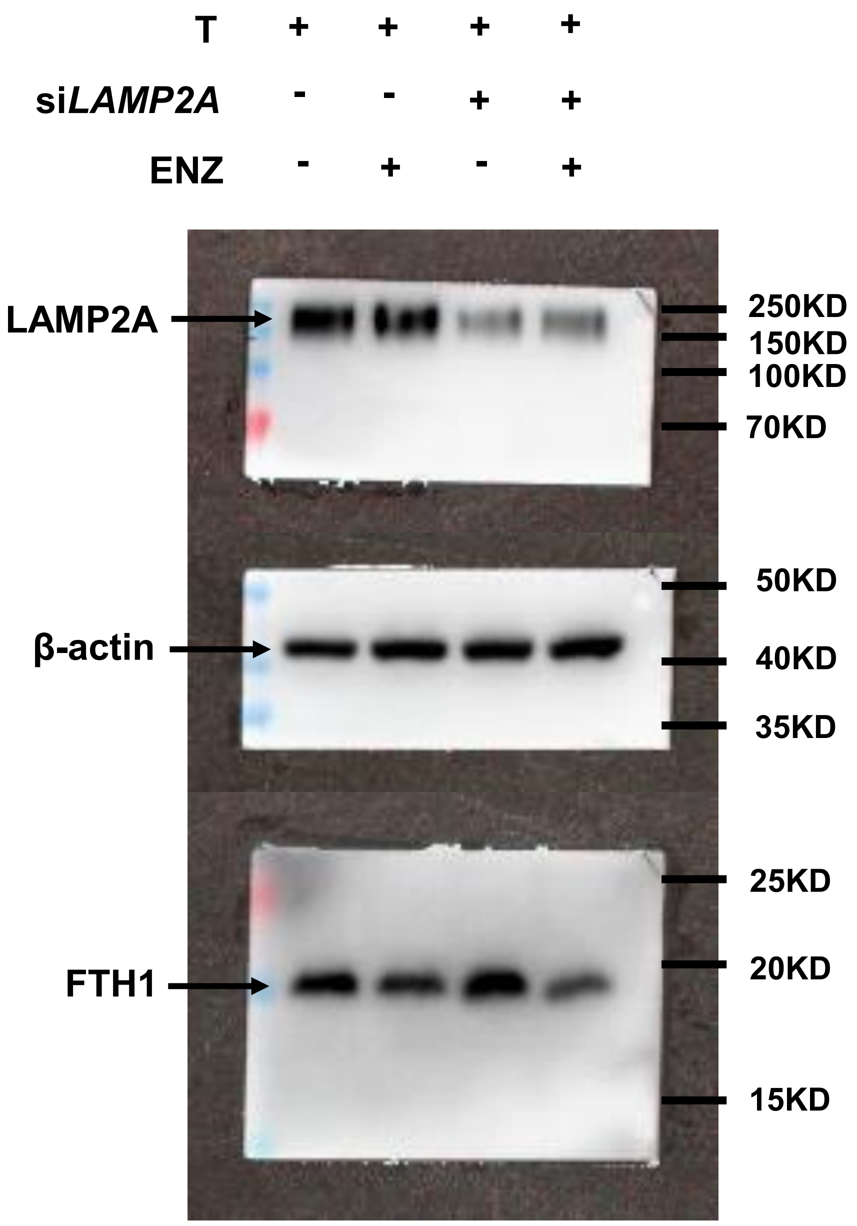


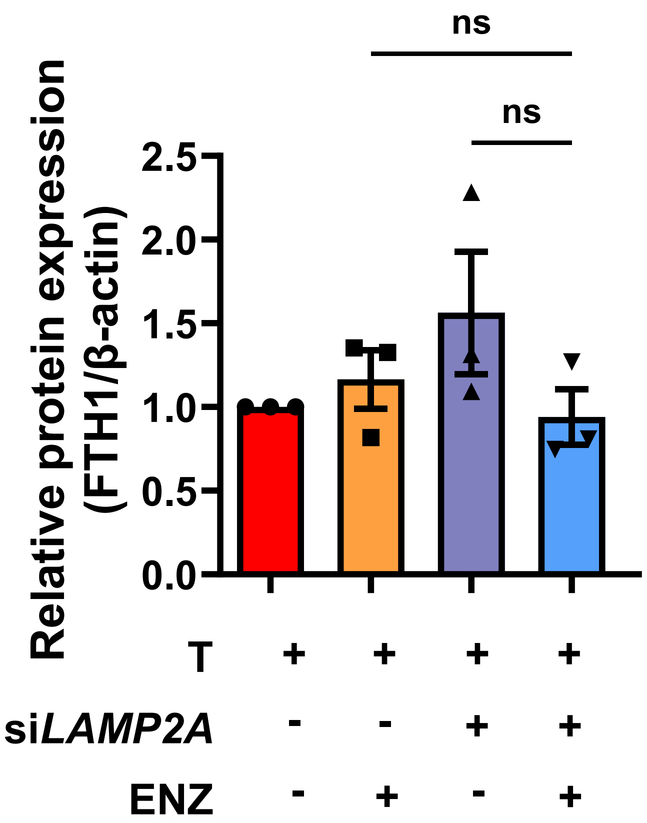

**Figure 5B：**

**The first repeat:**

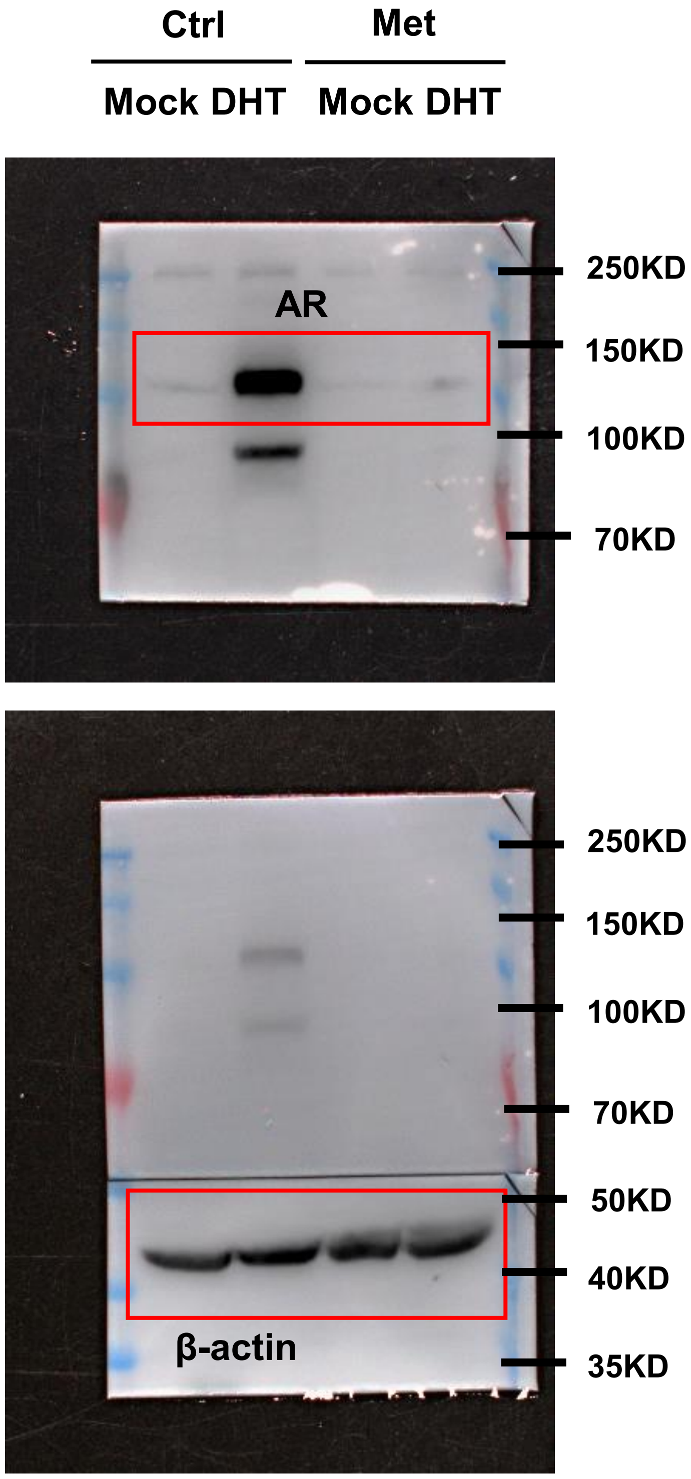


**The second repeat:**

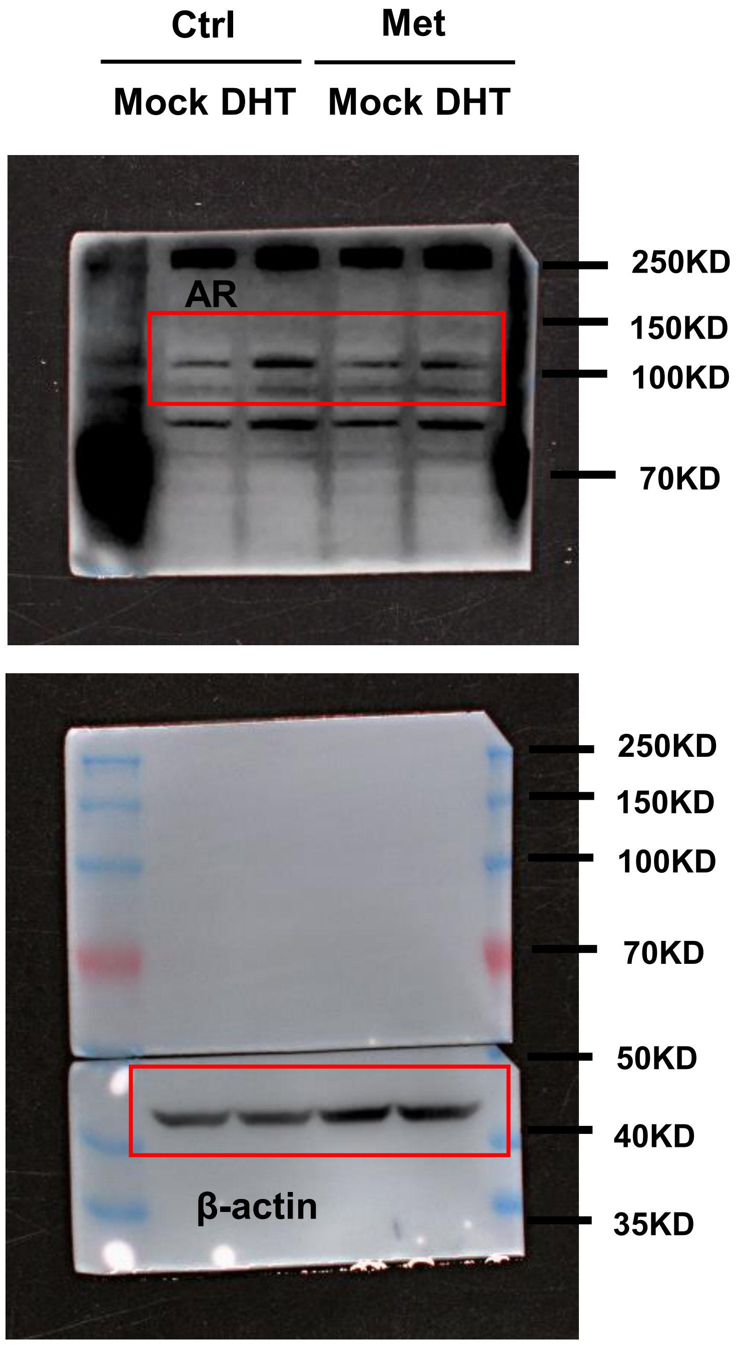


**The third repeat:**

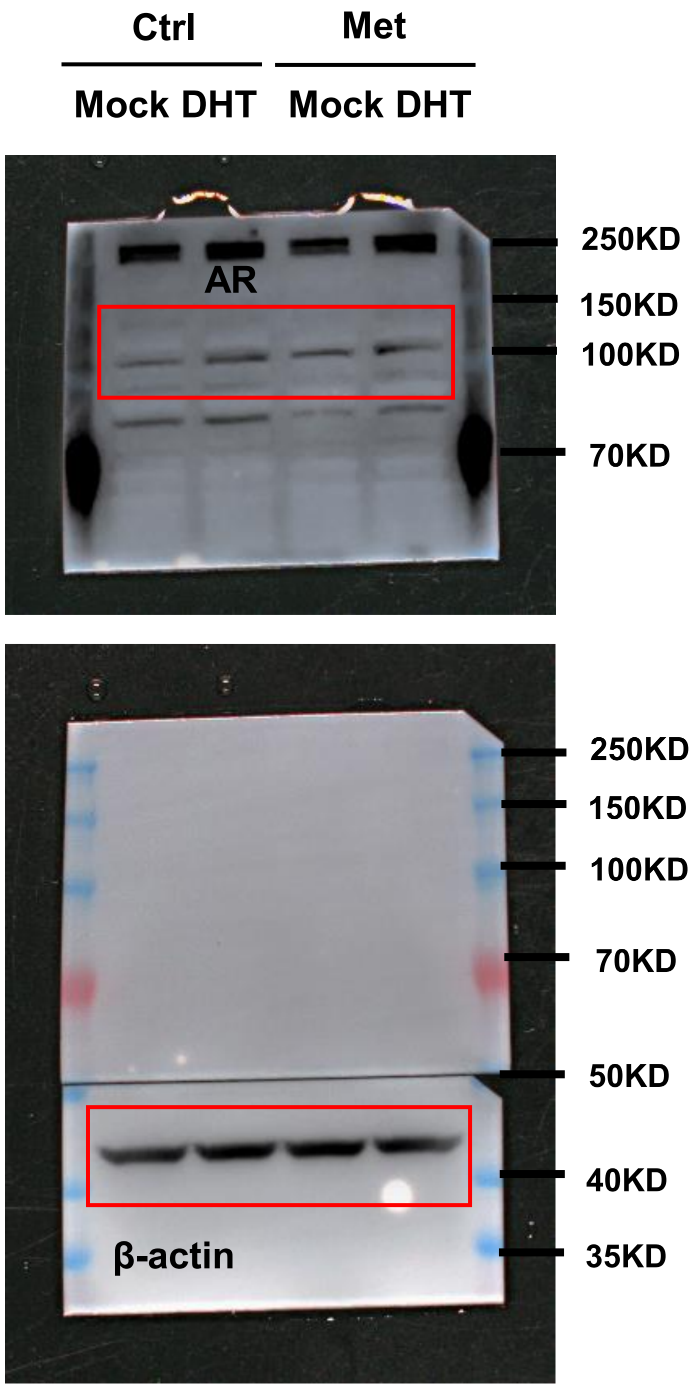


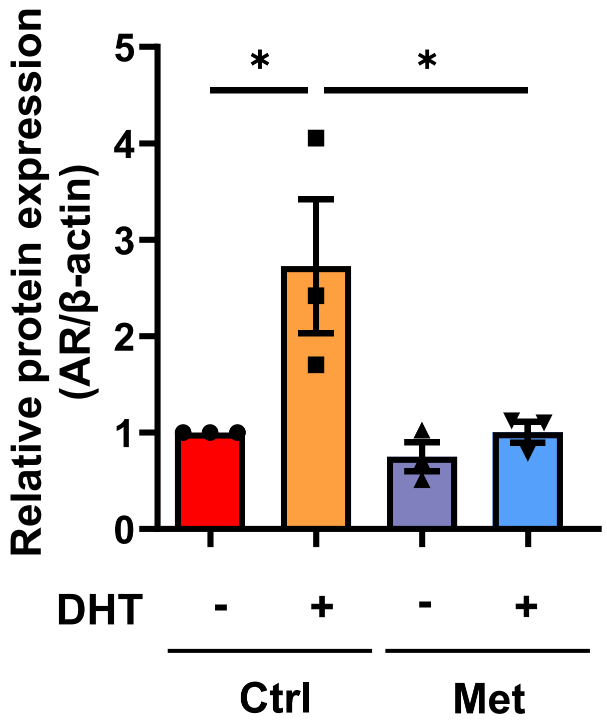

## Figure 5F：

### The first repeat:

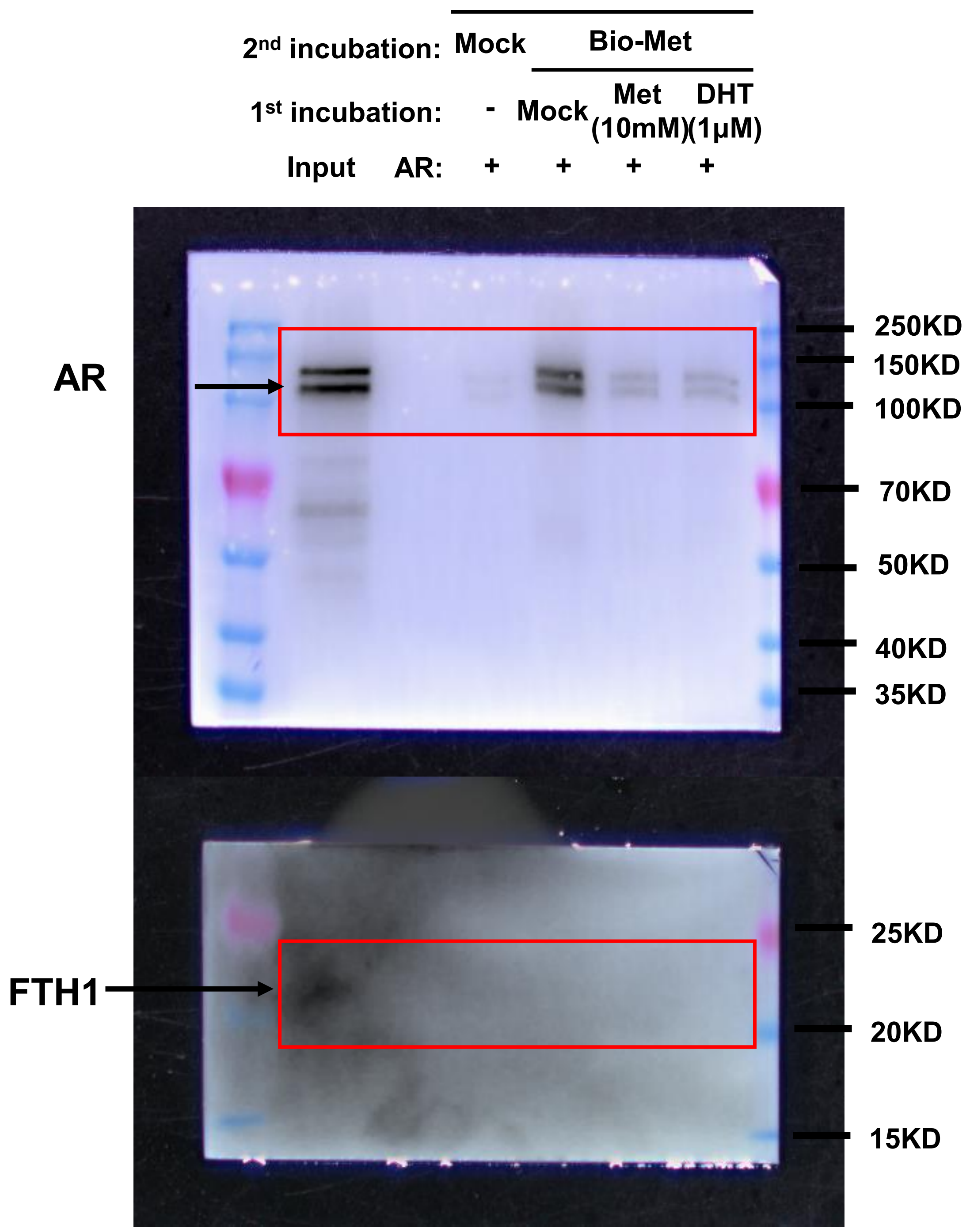


### The second repeat:

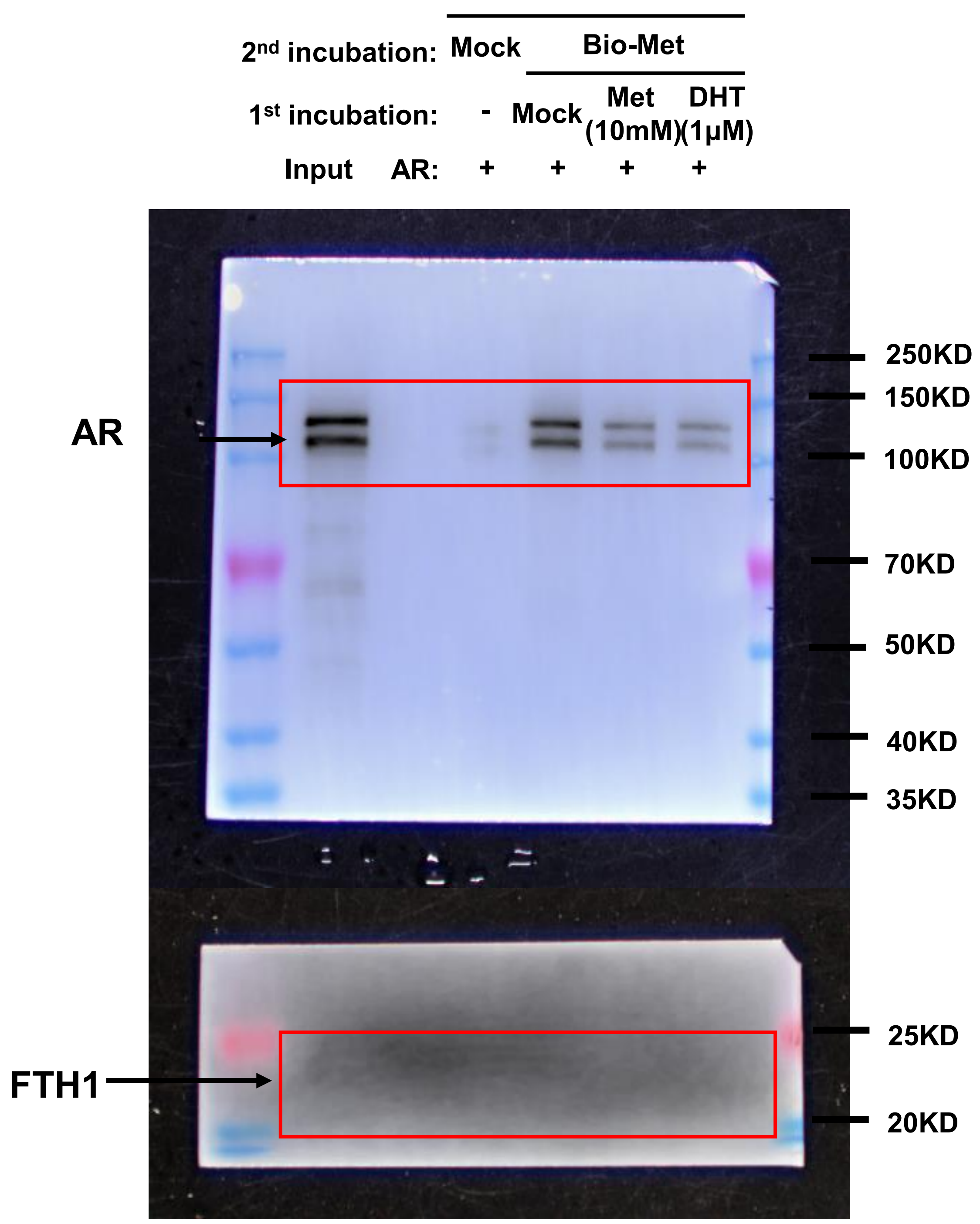


### The third repeat:

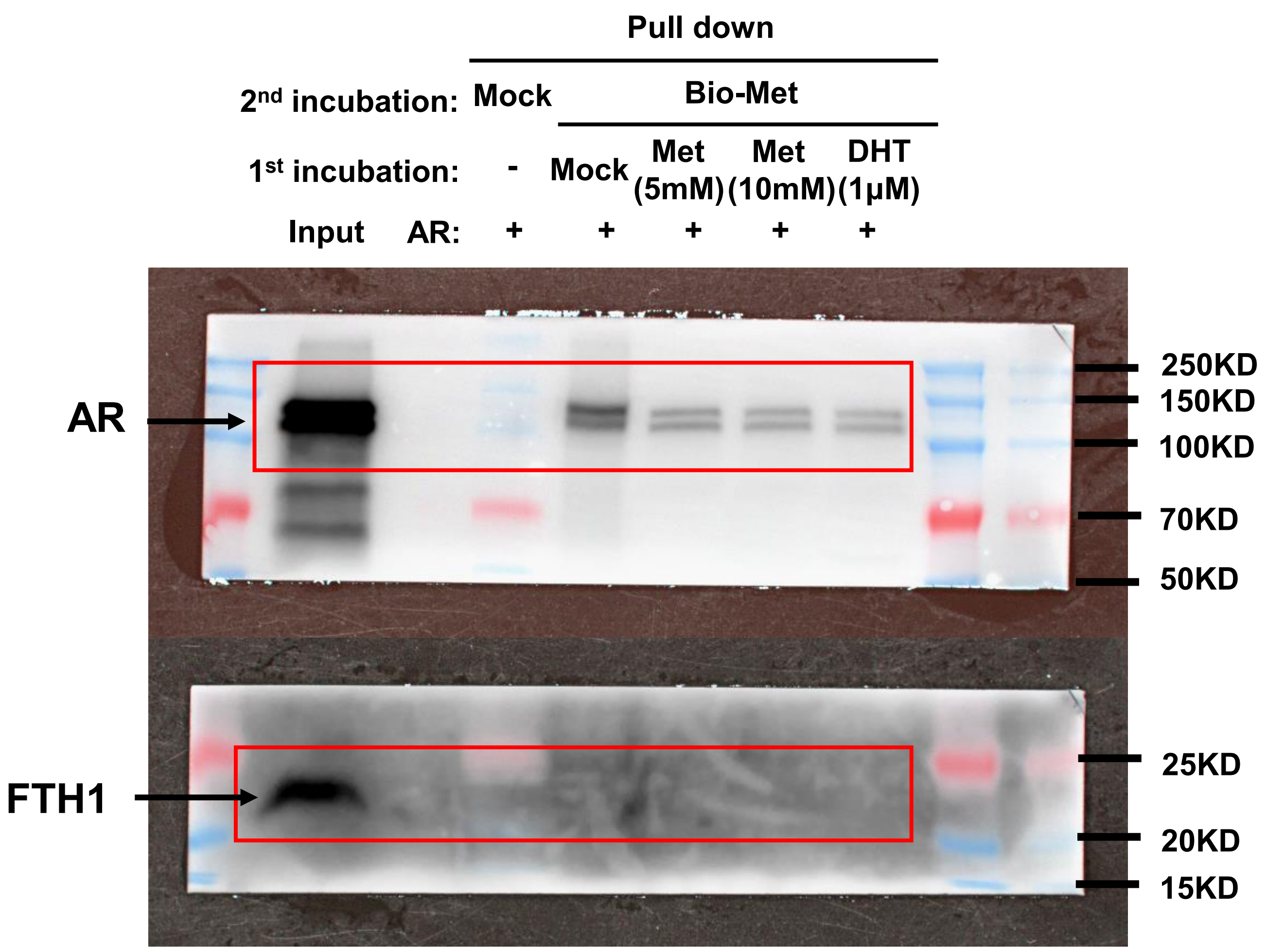

# Figure 5G：

## The first repeat:

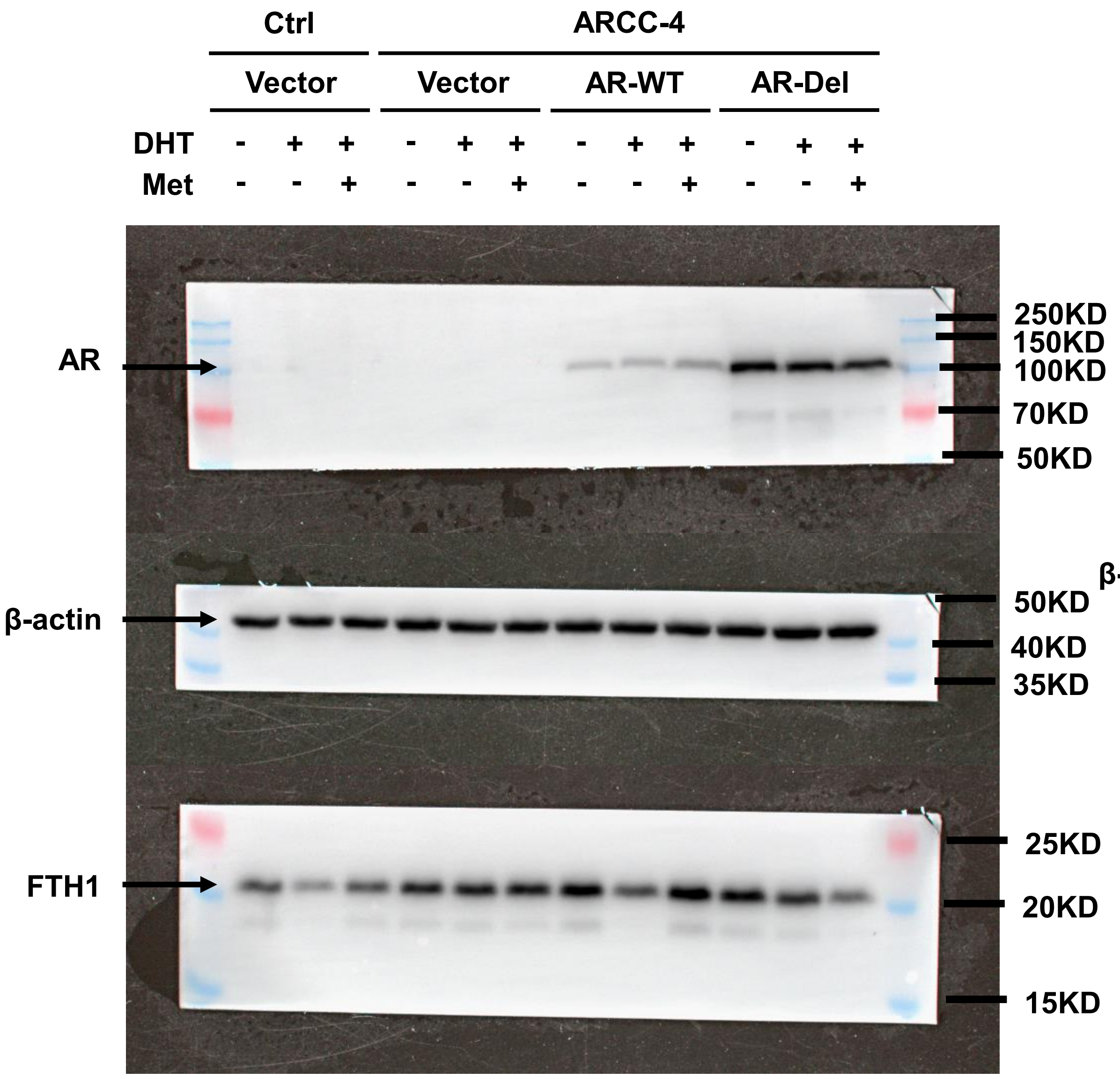


## The second repeat:

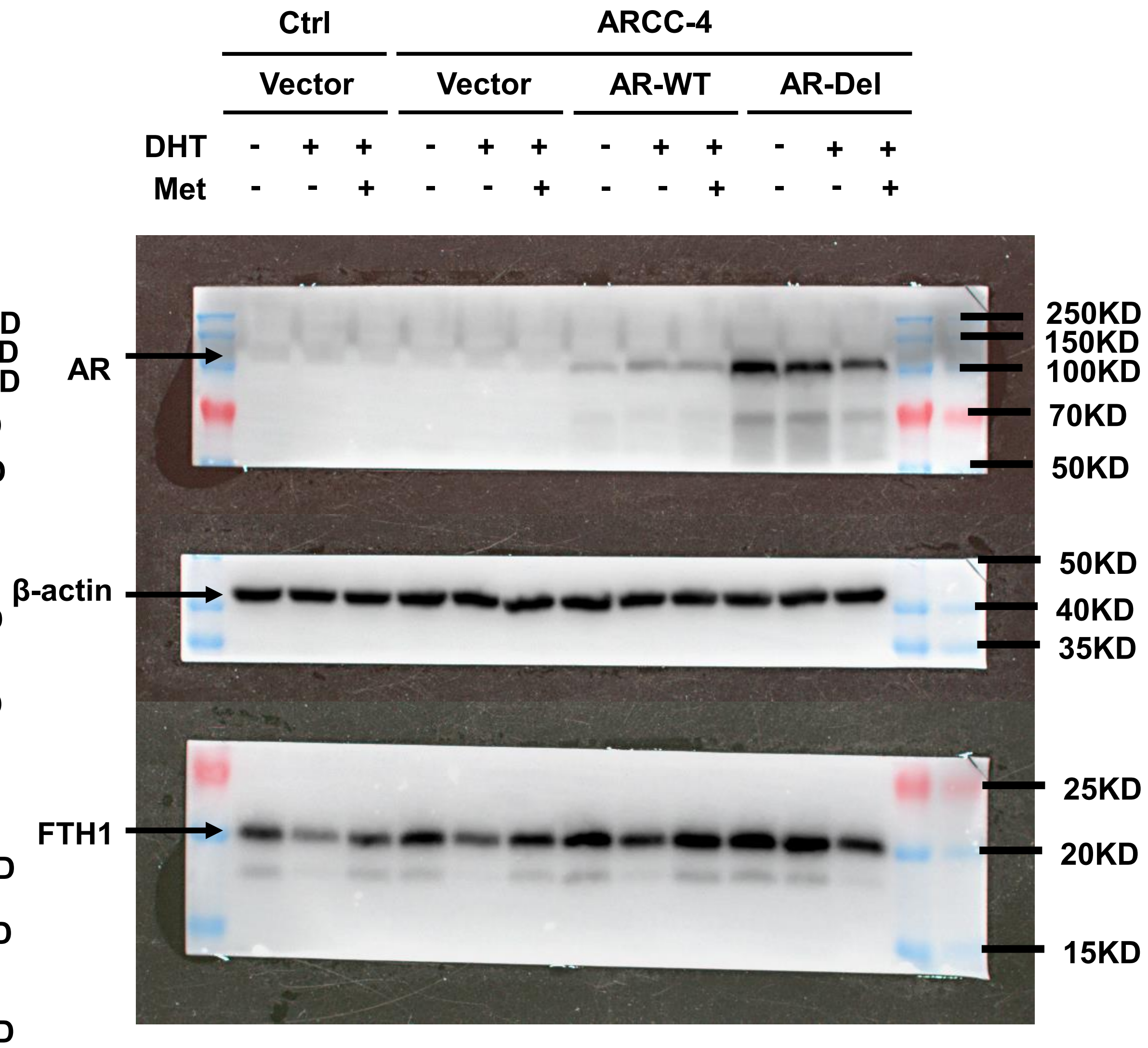


## The third repeat:

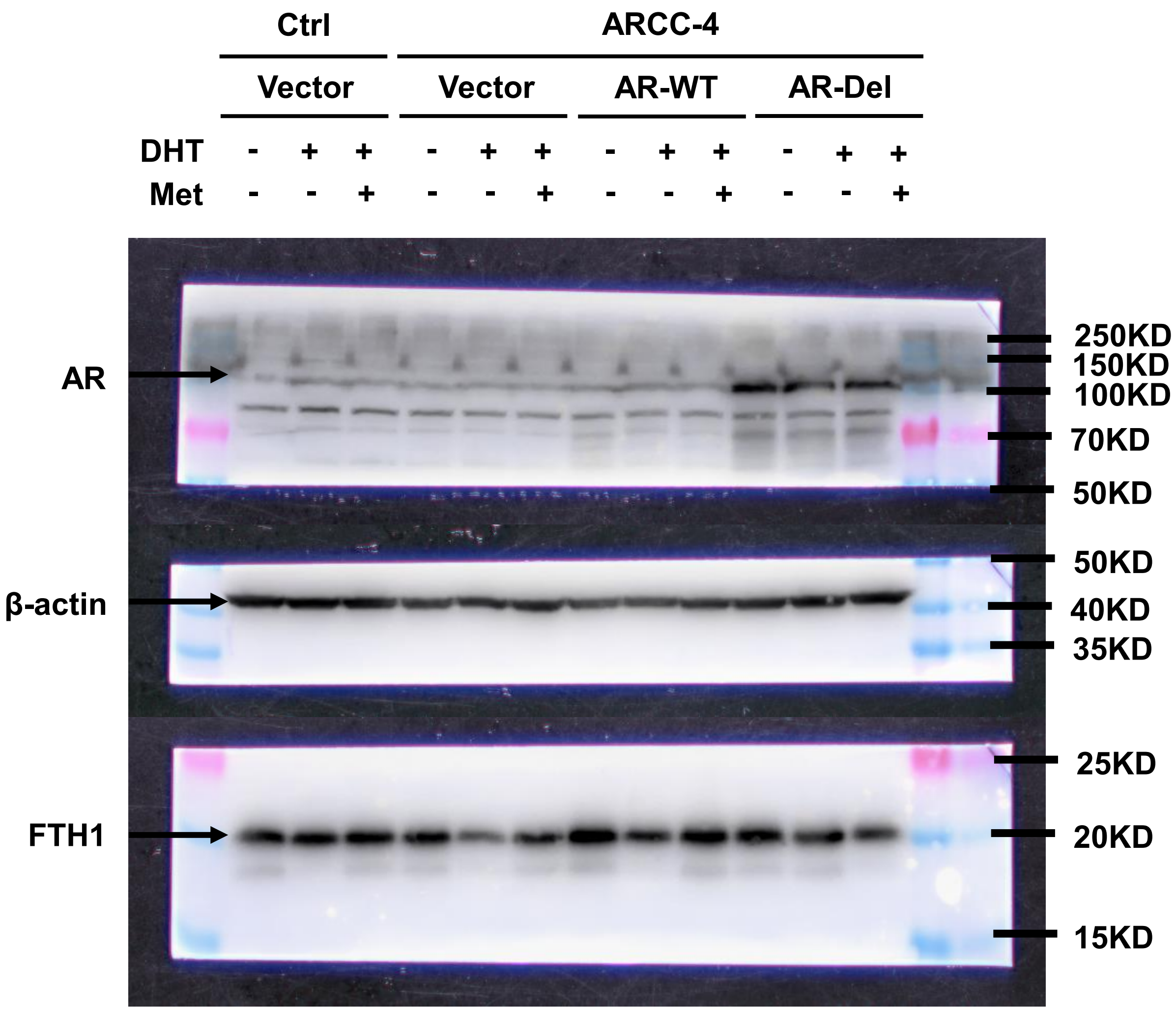


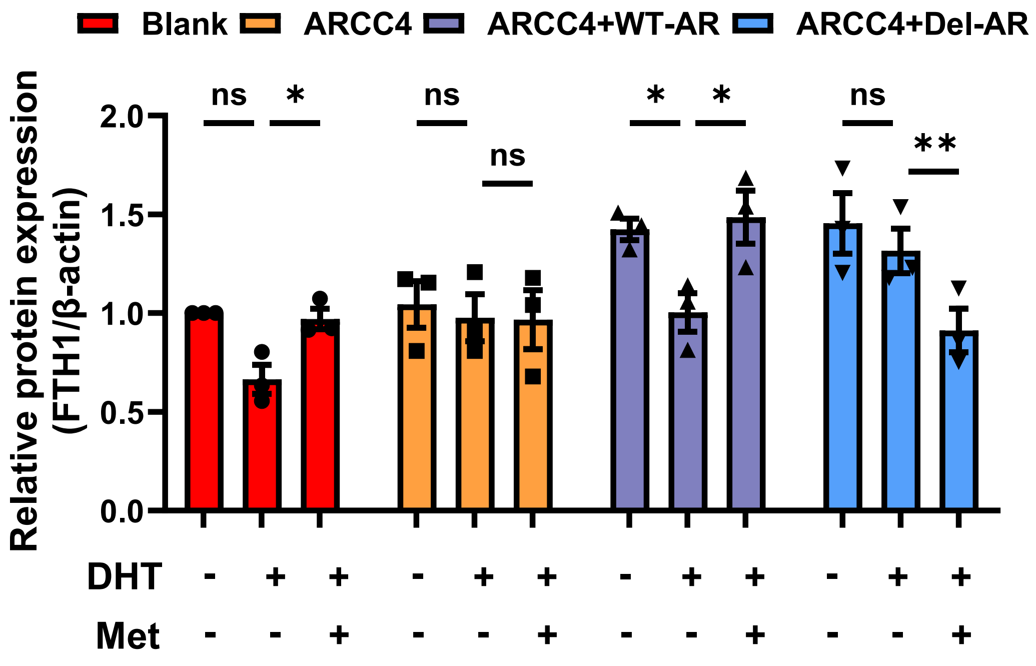

**Figure 6K:**

**The first repeat:**

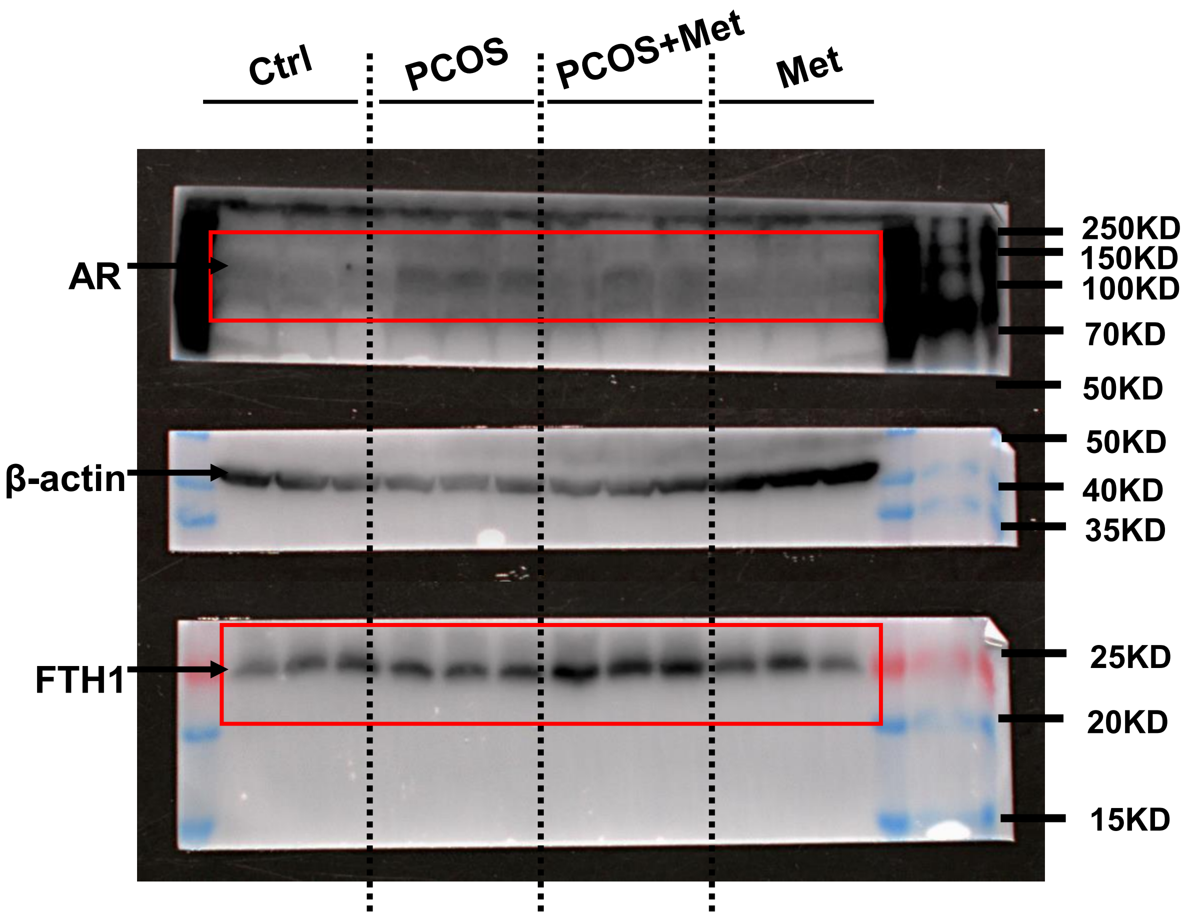


**The third repeat:**

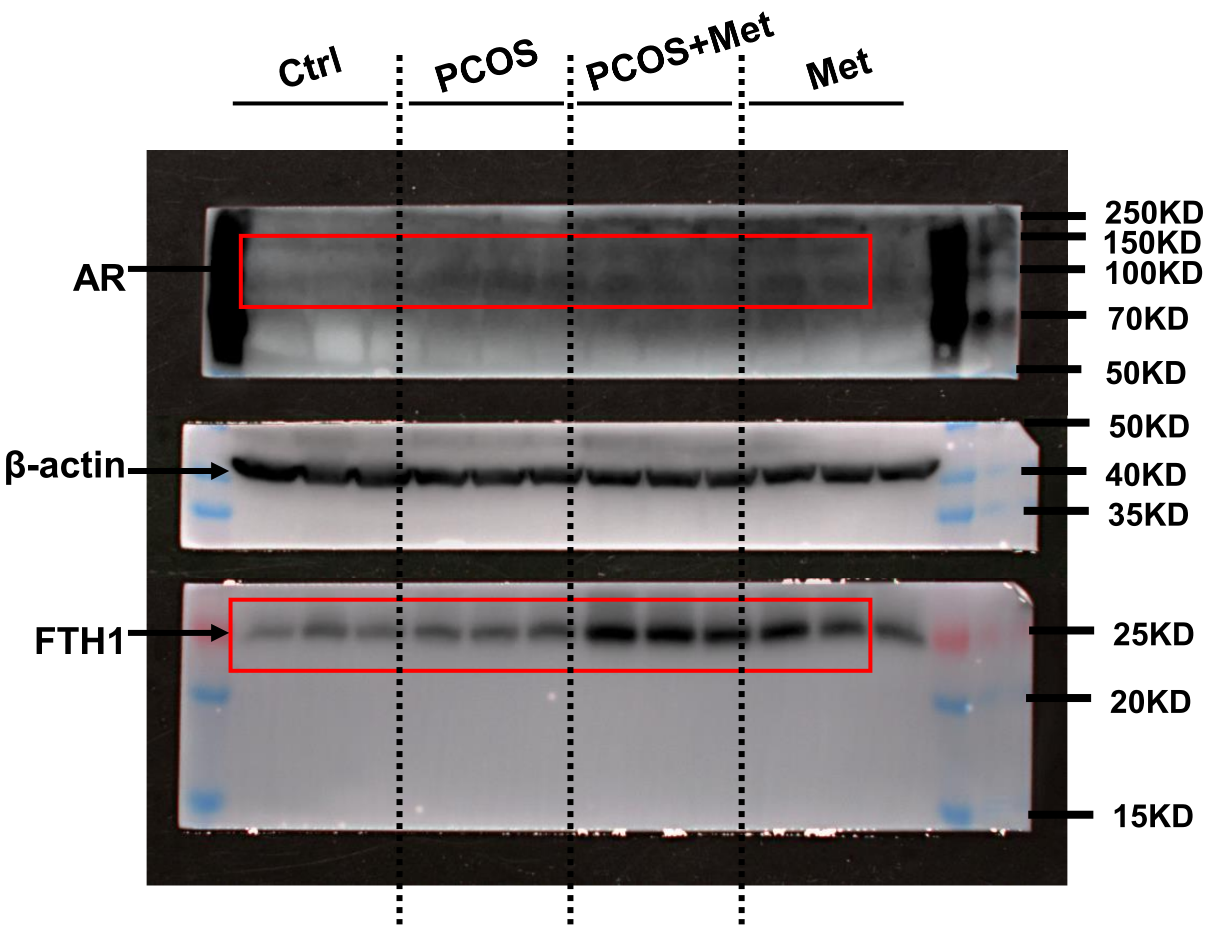


**The second repeat:**

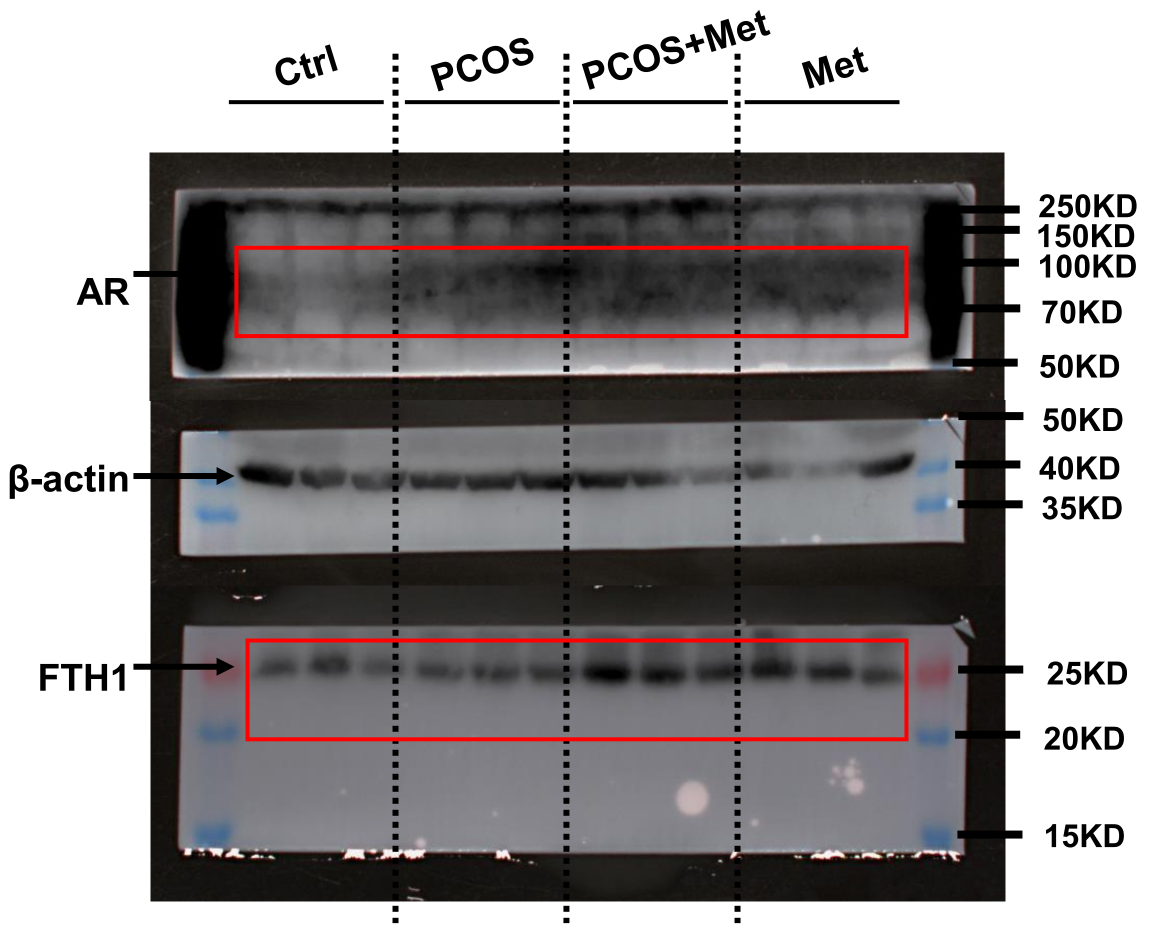


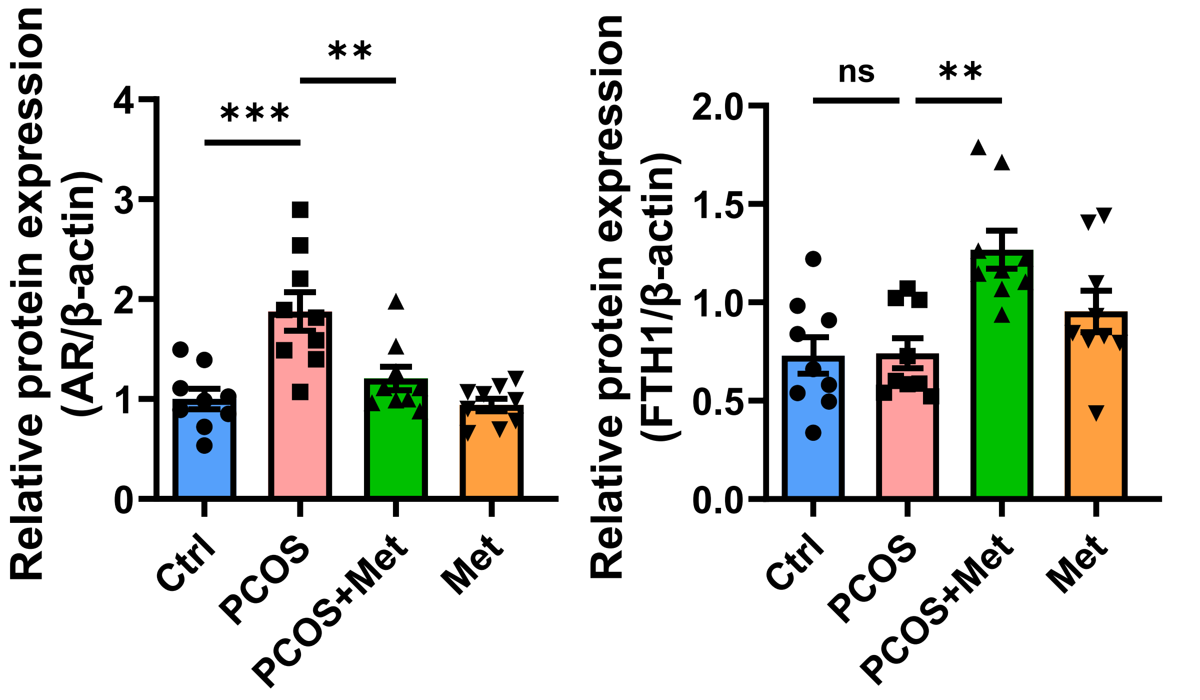

Figure S2D:

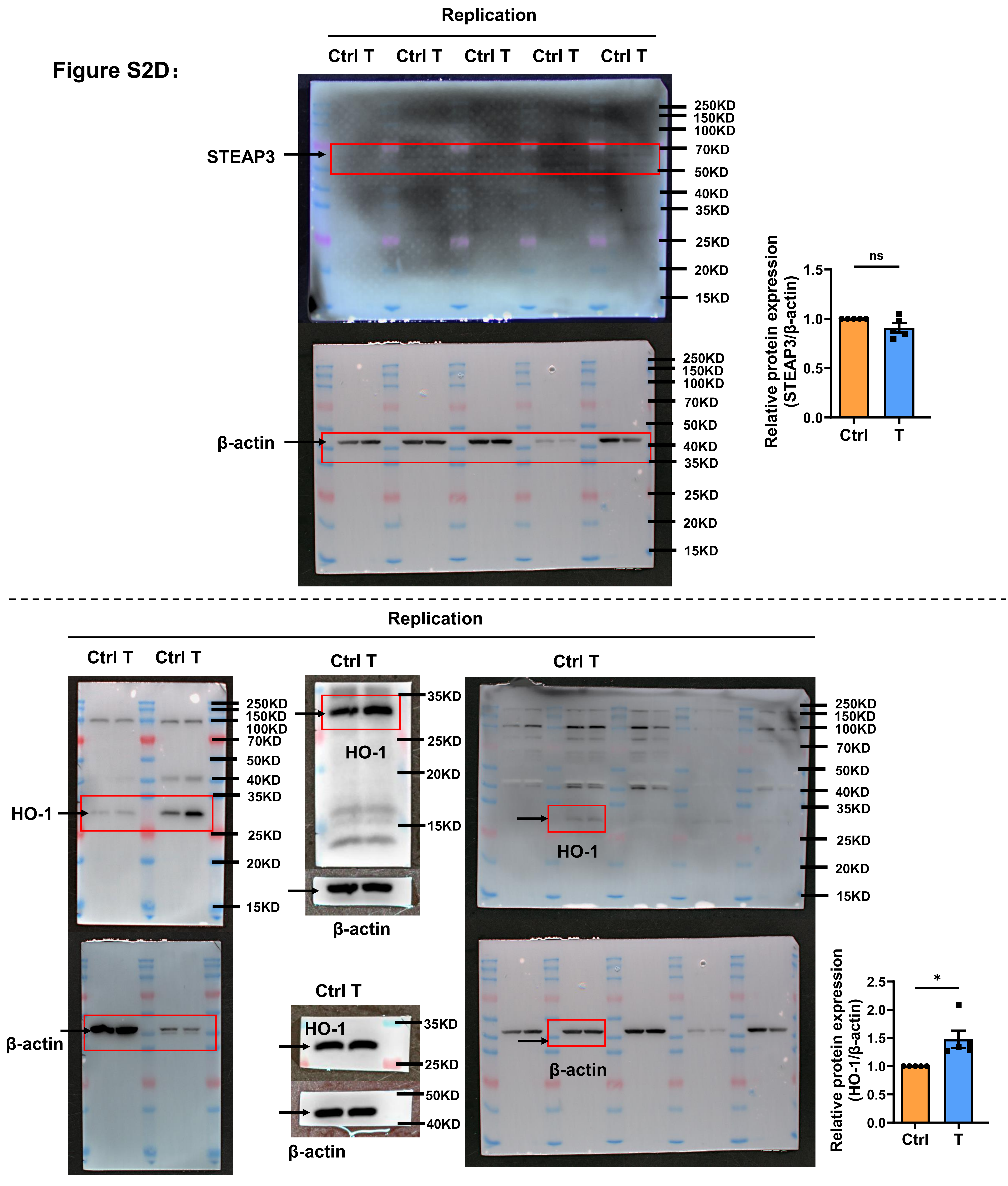

Replication
Ctrl T Ctrl T Ctrl T Ctrl T Ctrl T
STEAP3
β-actin
250KD
150KD
100KD
70KD
50KD
40KD
35KD
25KD
20KD
15KD
Relative protein expression (STEAP3/β-actin)
ns
1.5
1.0
0.5
0.0
Ctrl
T
Replication
Ctrl T Ctrl T
HO-1
β-actin
Ctrl T
HO-1
β-actin
Ctrl T
HO-1
β-actin
Ctrl T
HO-1
β-actin
Relative protein expression (HO-1/β-actin)
*
2.5
2.0
1.5
1.0
0.5
0.0
Ctrl
T

**Figure S2G:**

**The first repeat:**

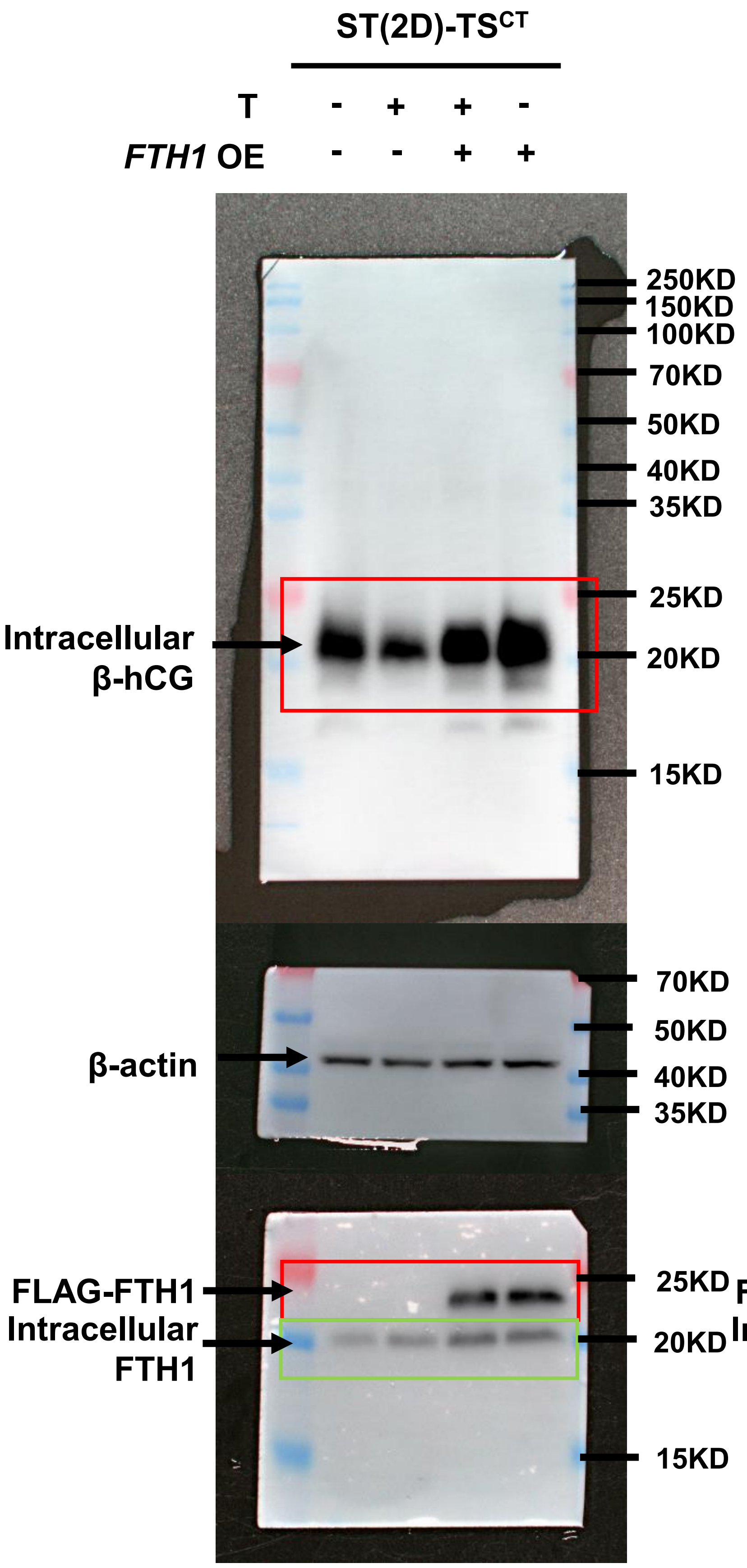


**The second repeat:**

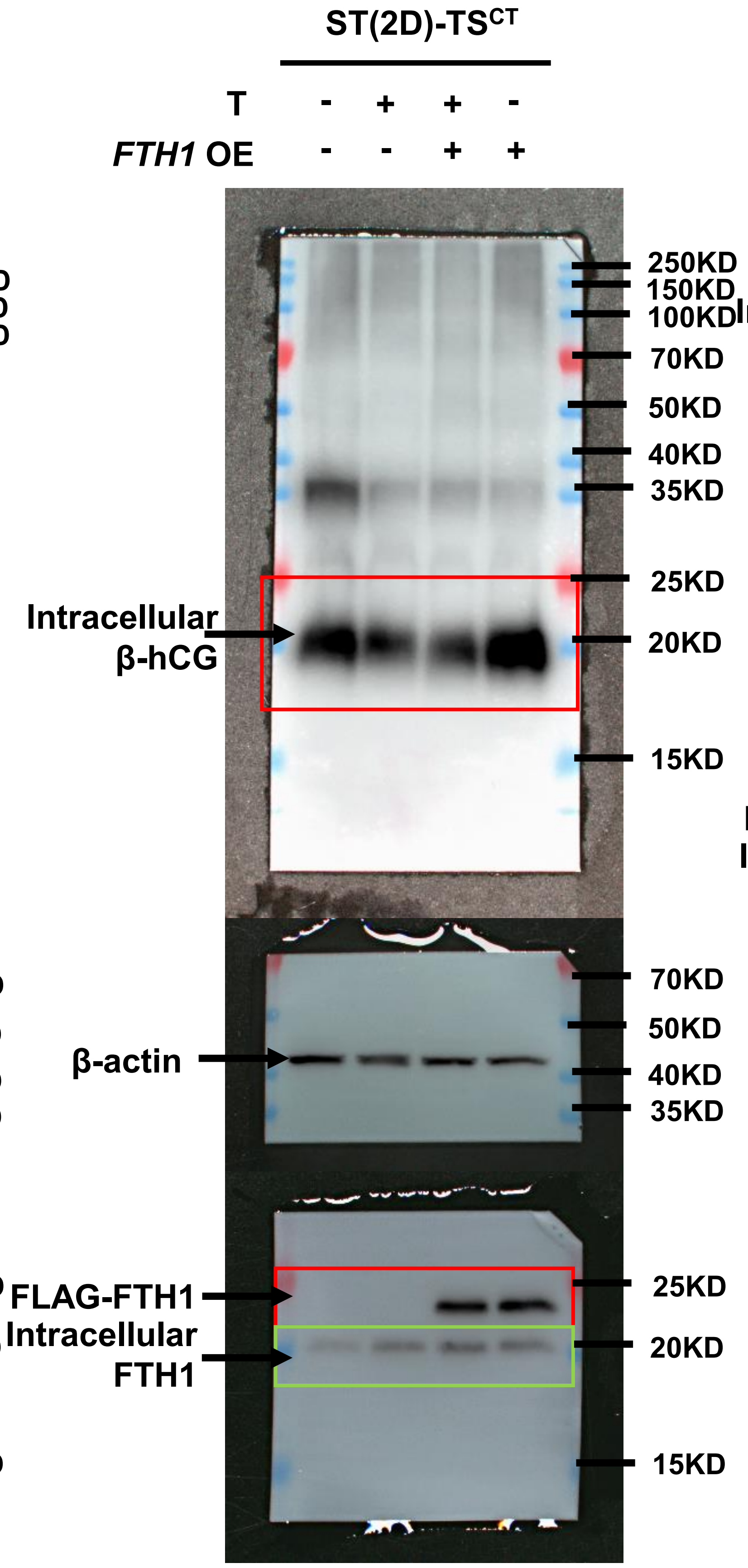


**The third repeat:**

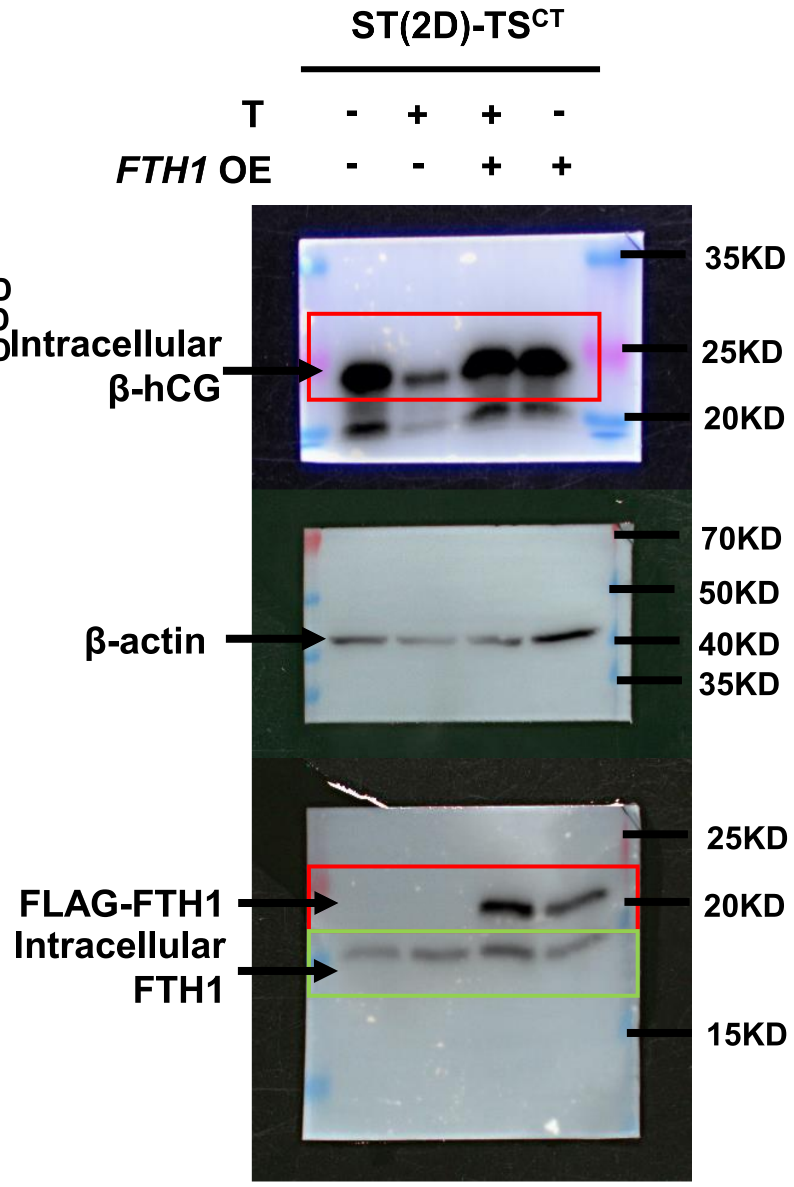


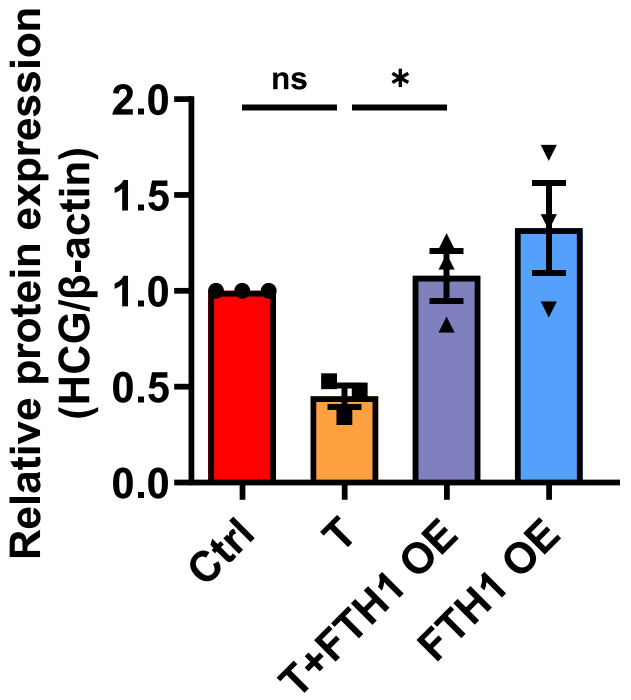

**Figure S3A:**

**The first repeat:**

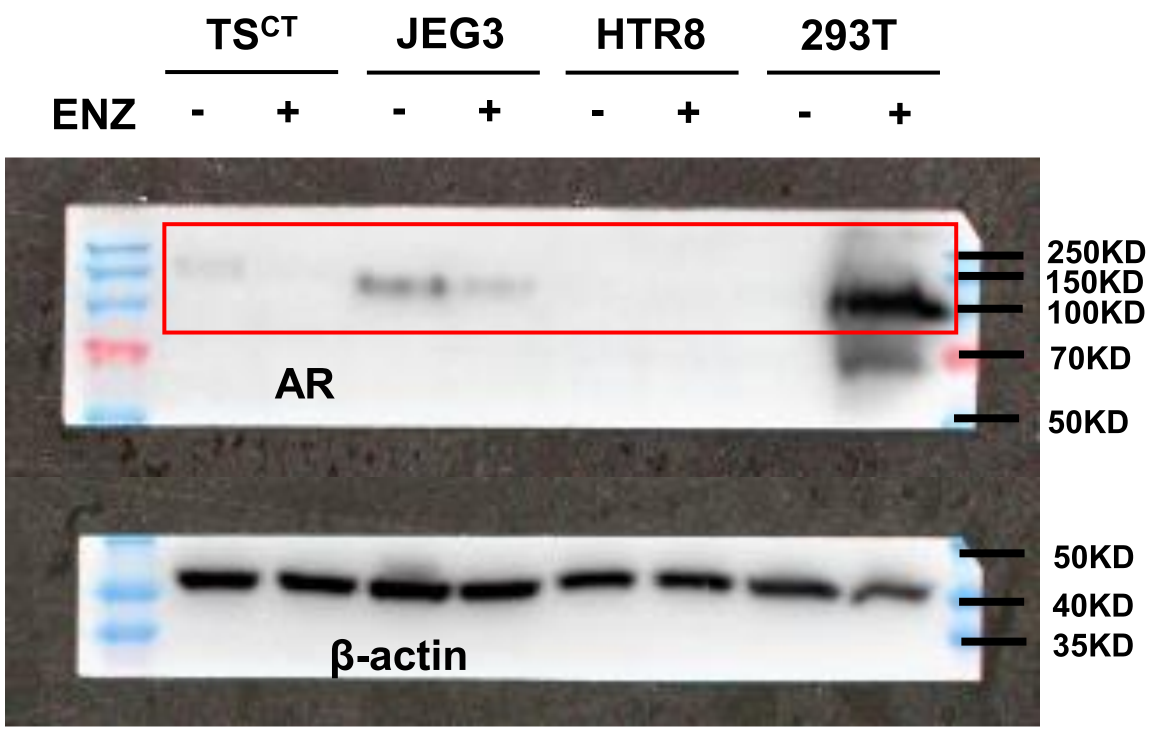


**The second repeat:**

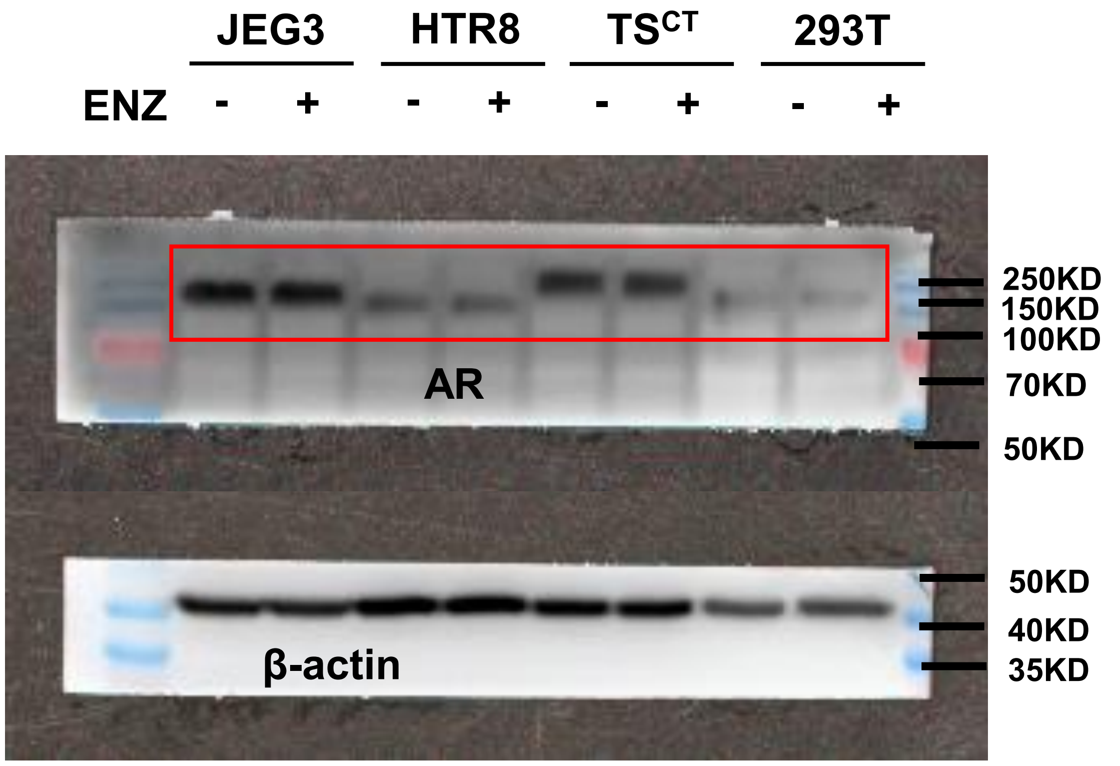


**The third repeat:**

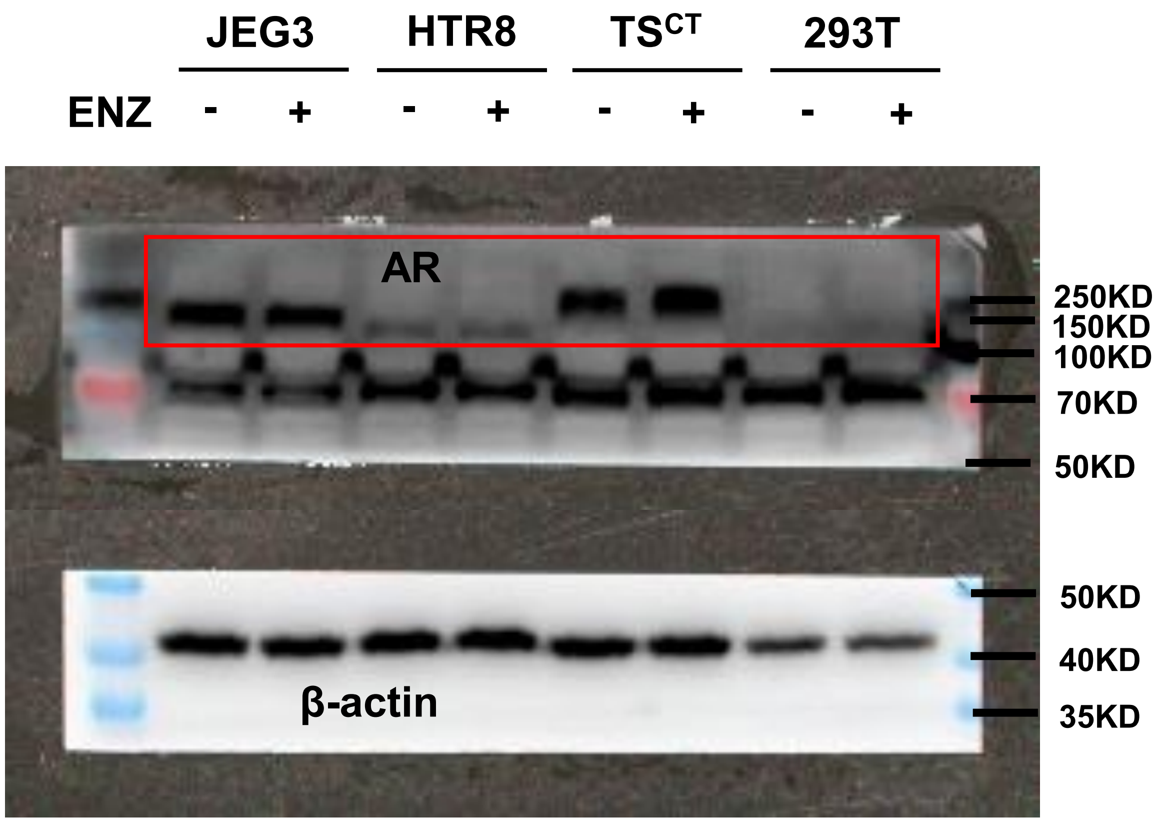


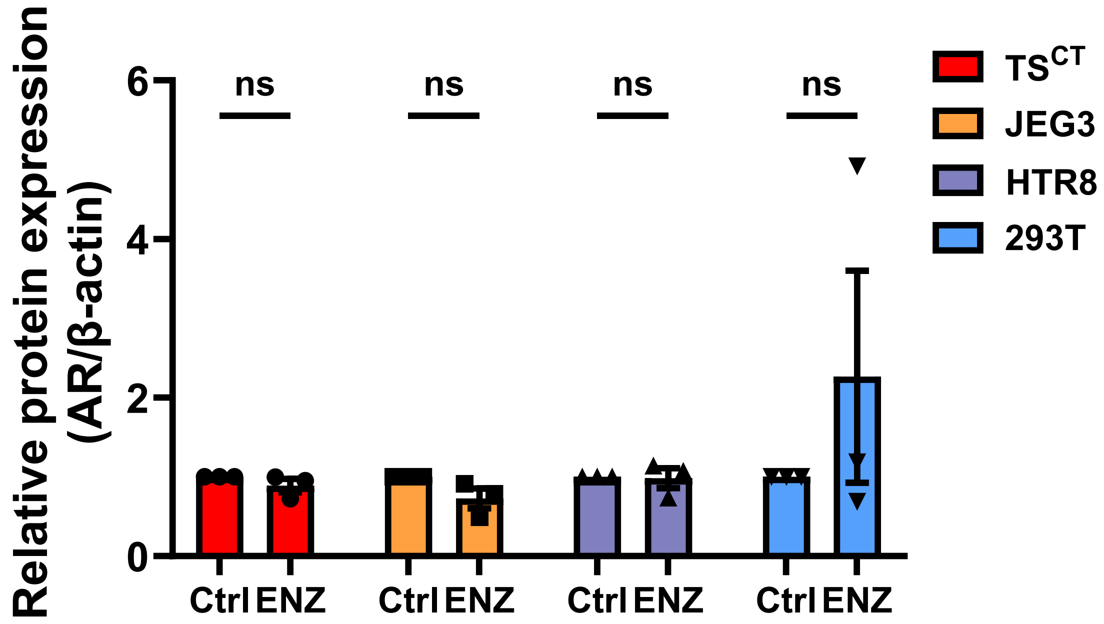


PS: The first/second repeat in Figure S3A and the first/second repeat in Figure S6A were derived from the same experiment, the WB membrane was washed with the stripping buffer.

## Figure S3C：

The first repeat:

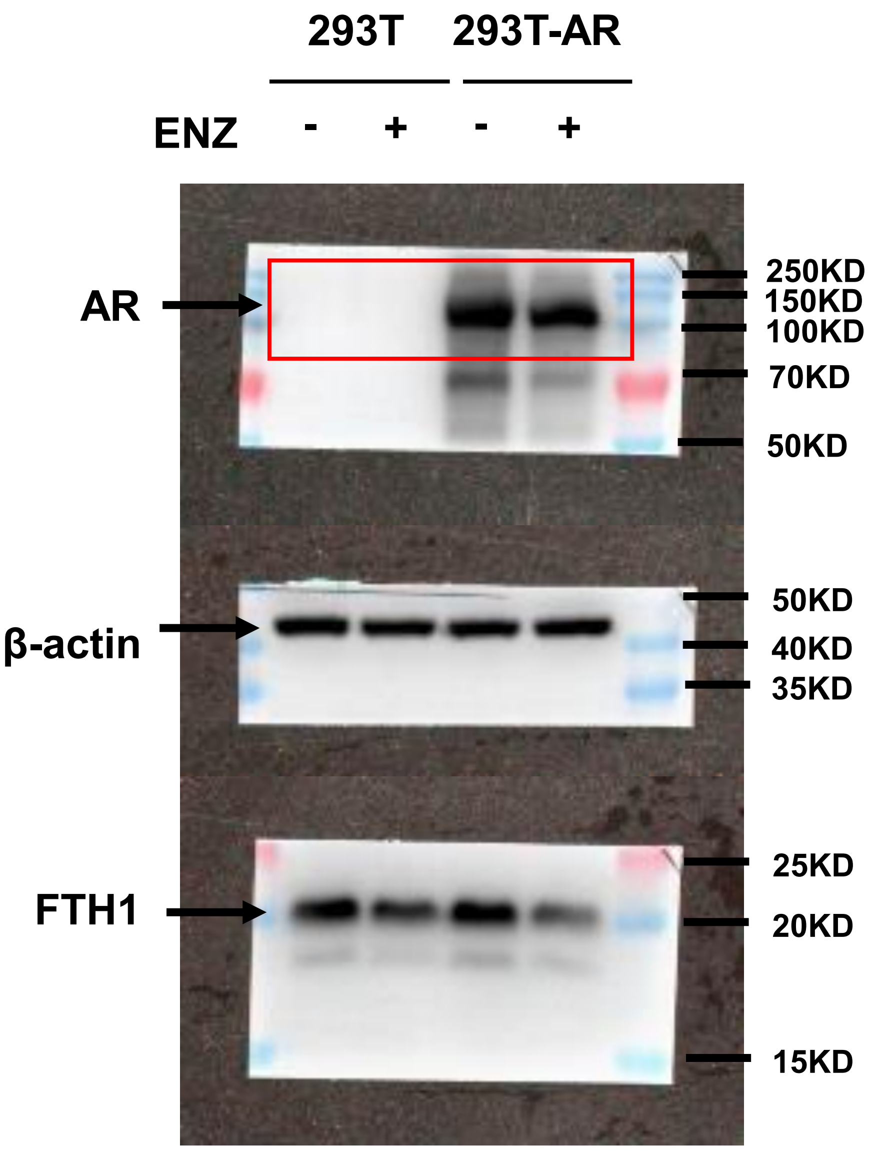


The second repeat:

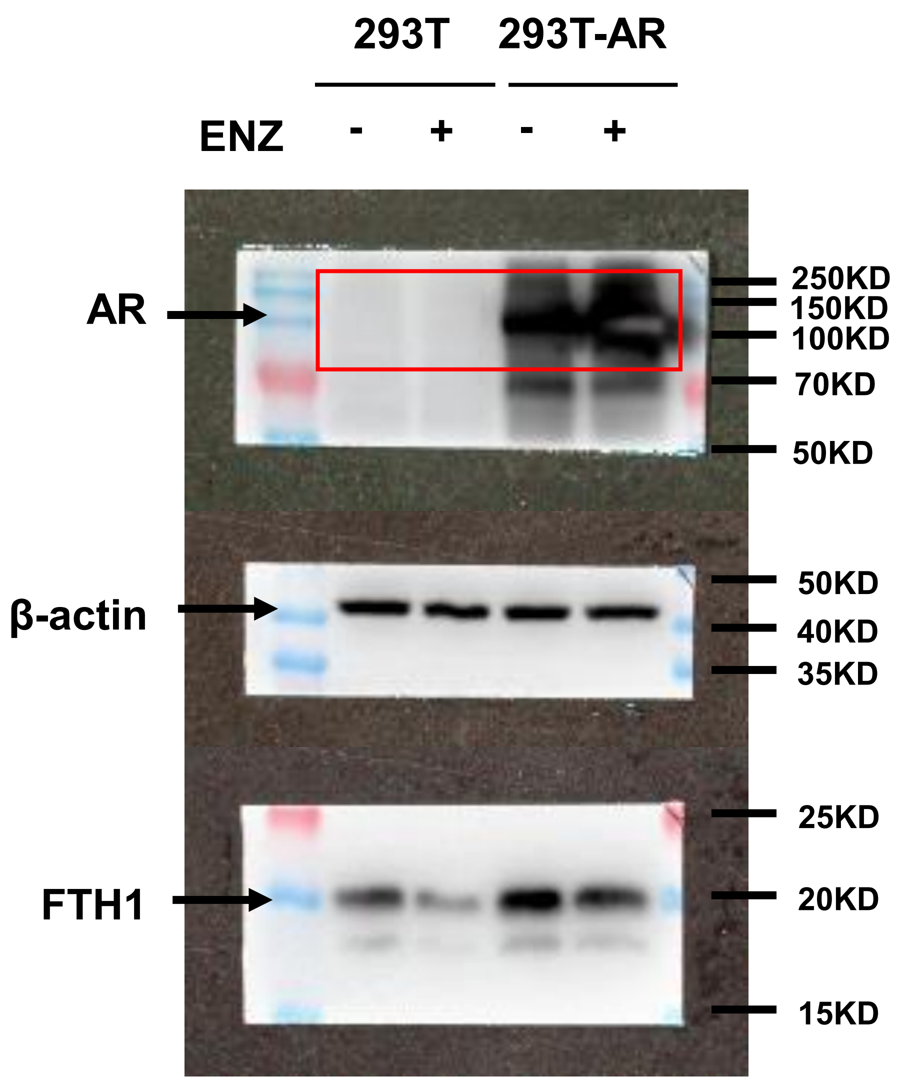


The third repeat:

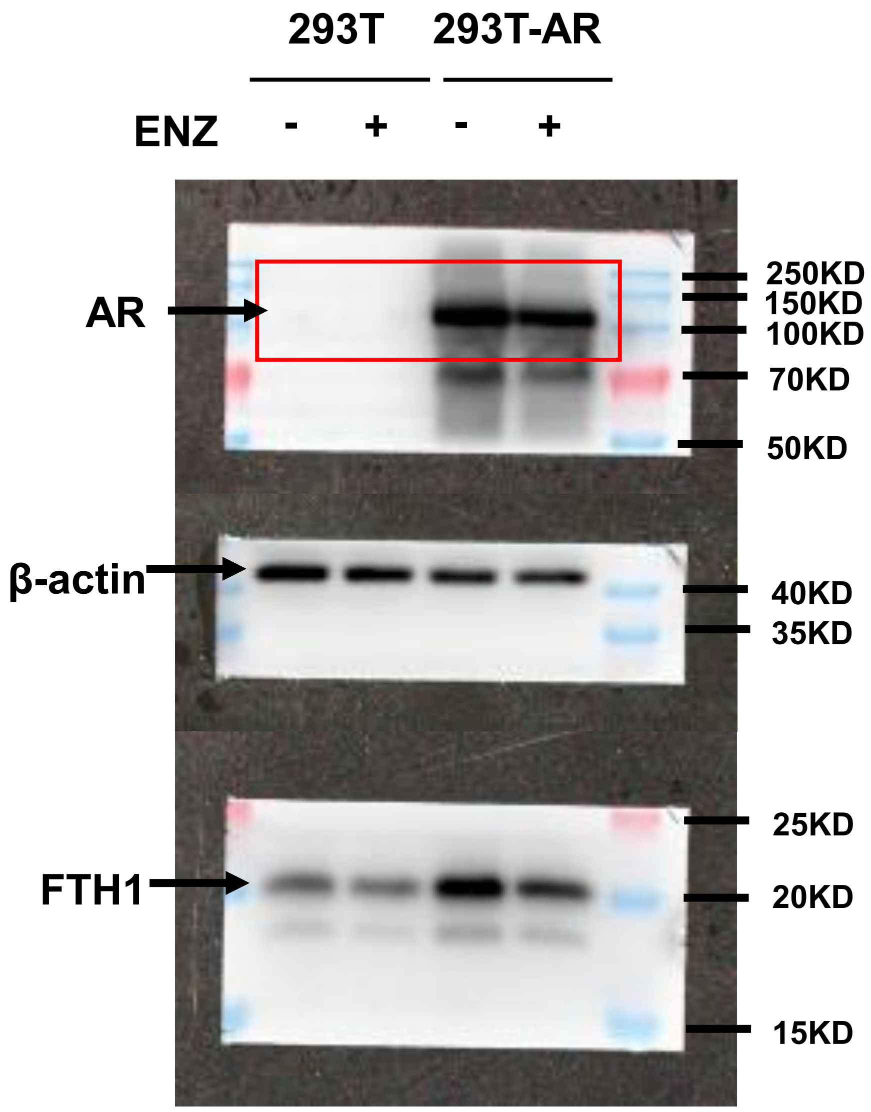


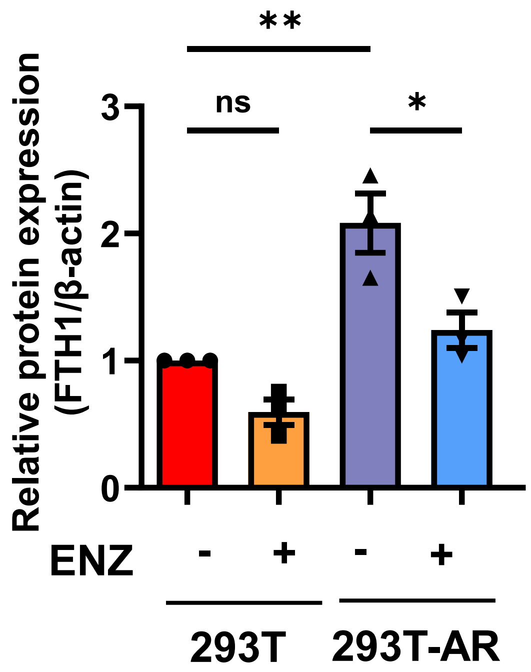

Figure S4D:

The first repeat:

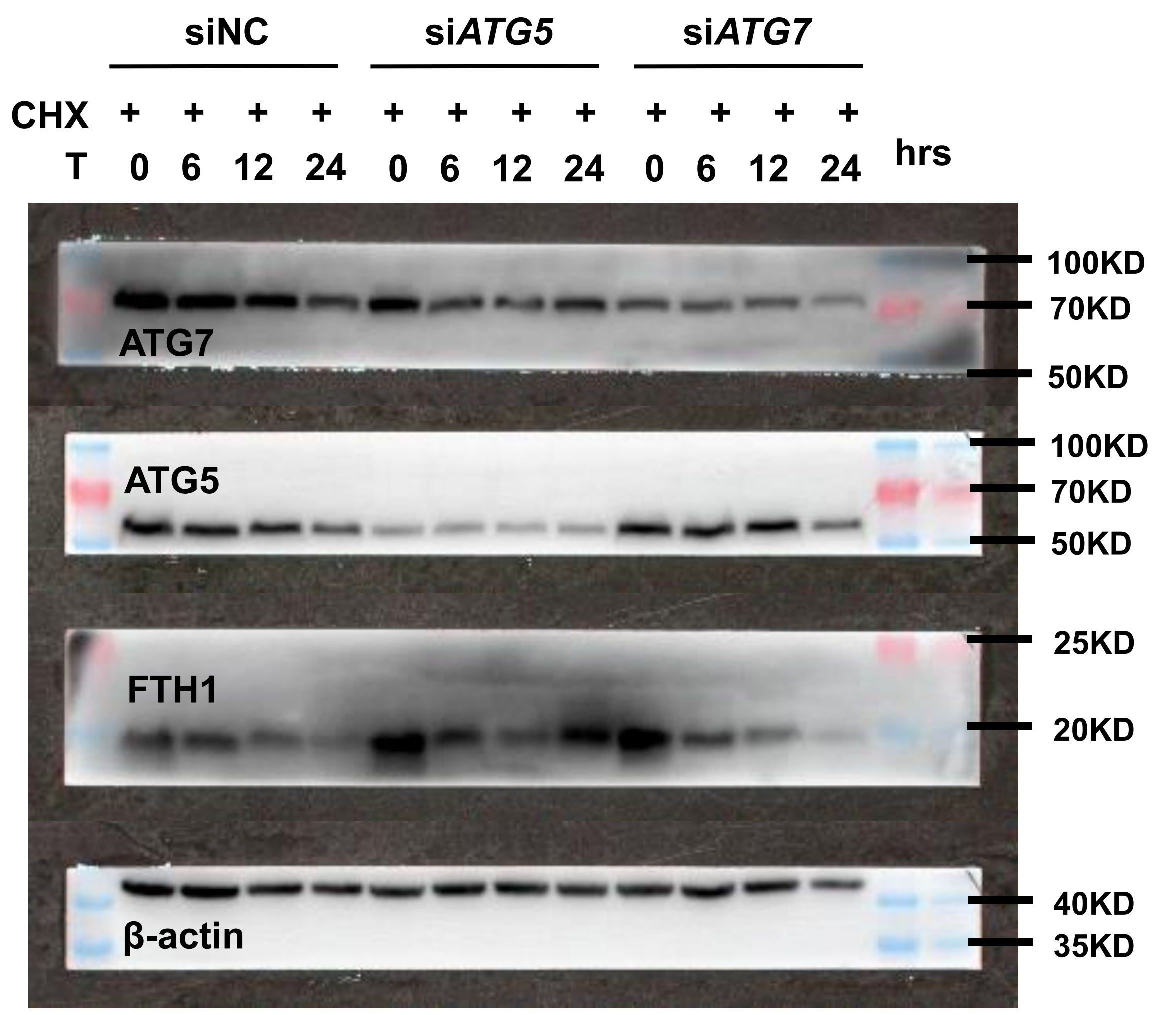


The second repeat:

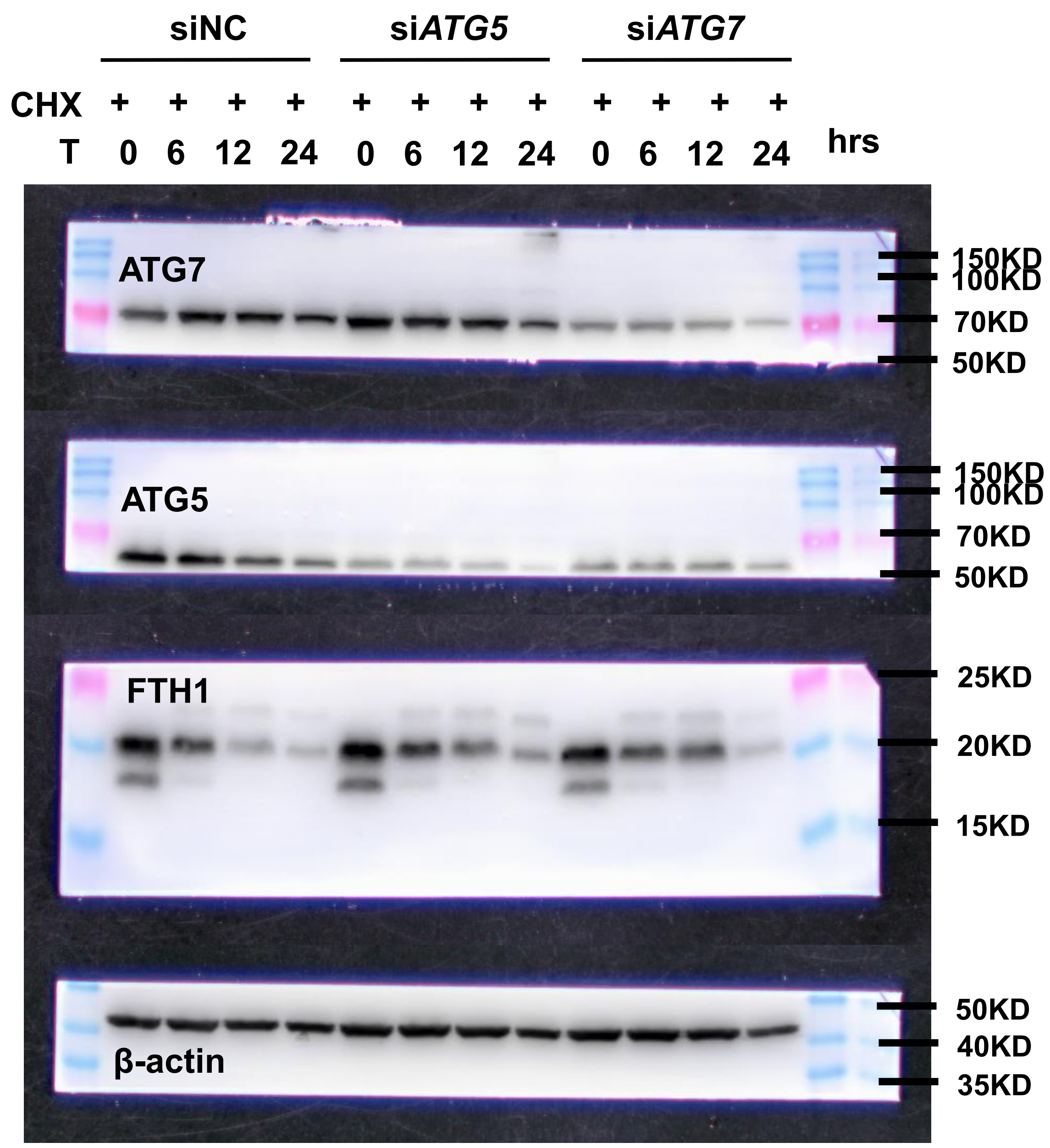


The third repeat:

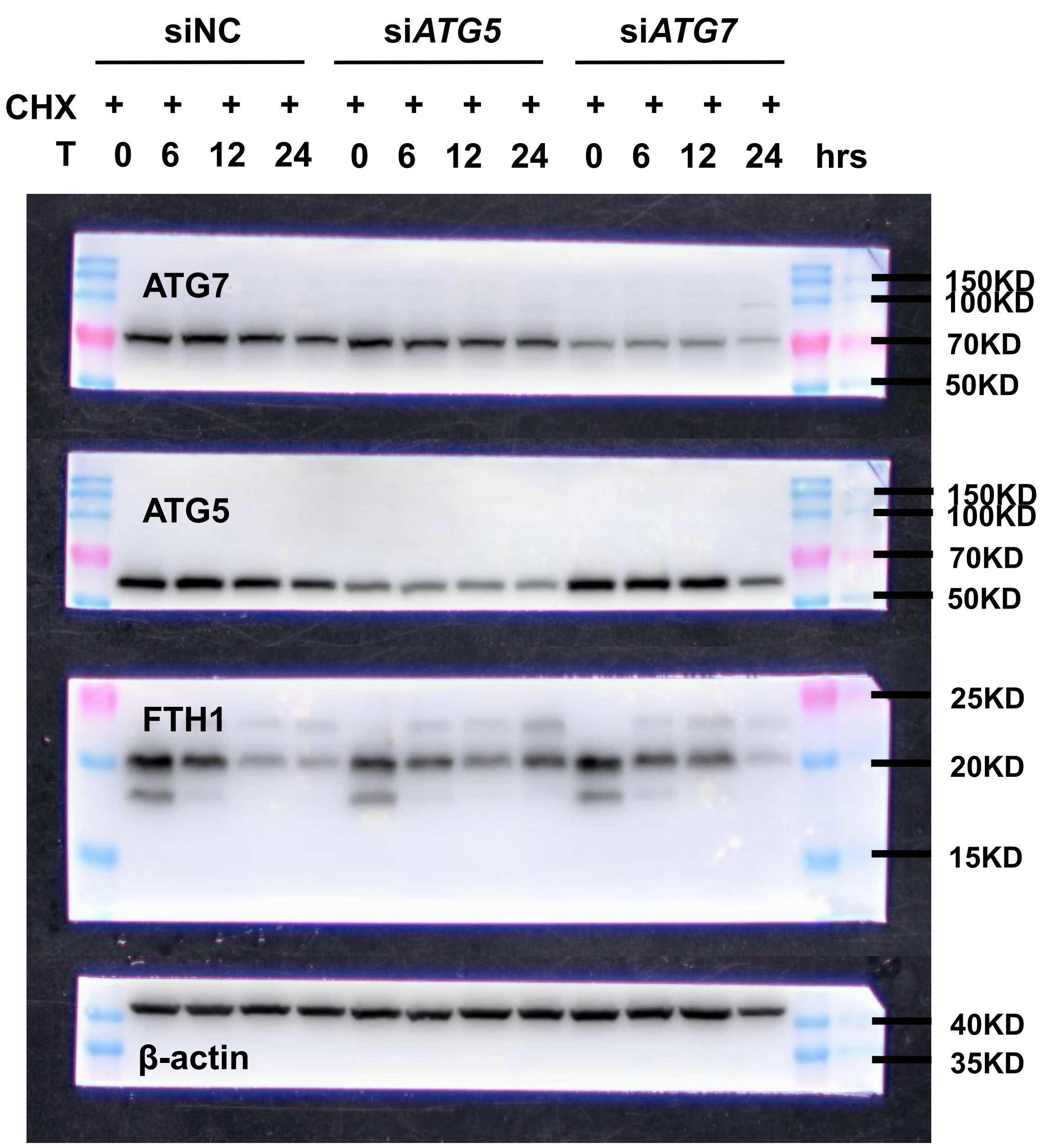


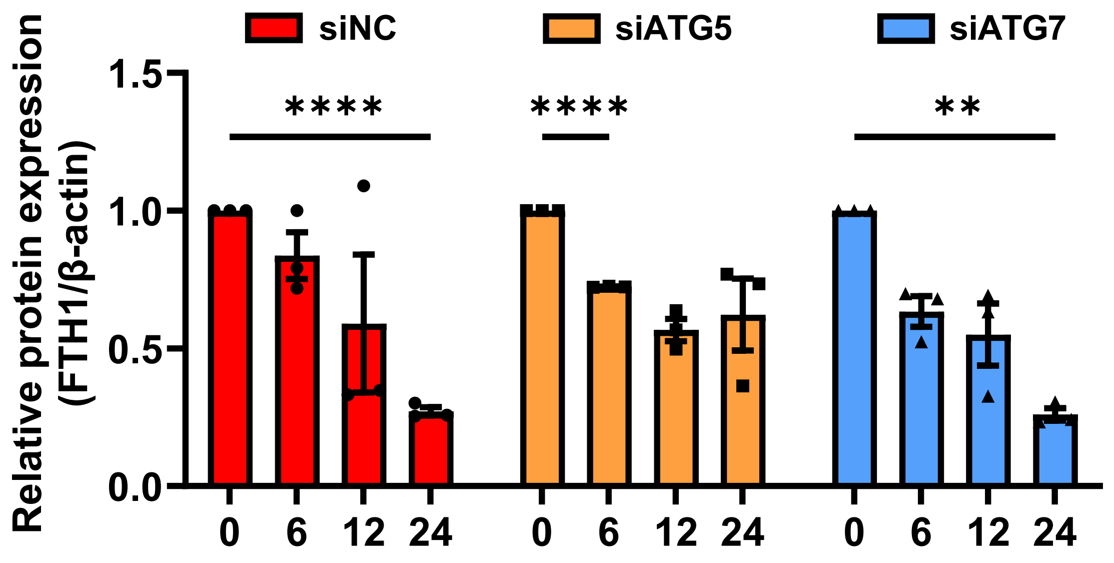

## Figure S5A：

### The first repeat:

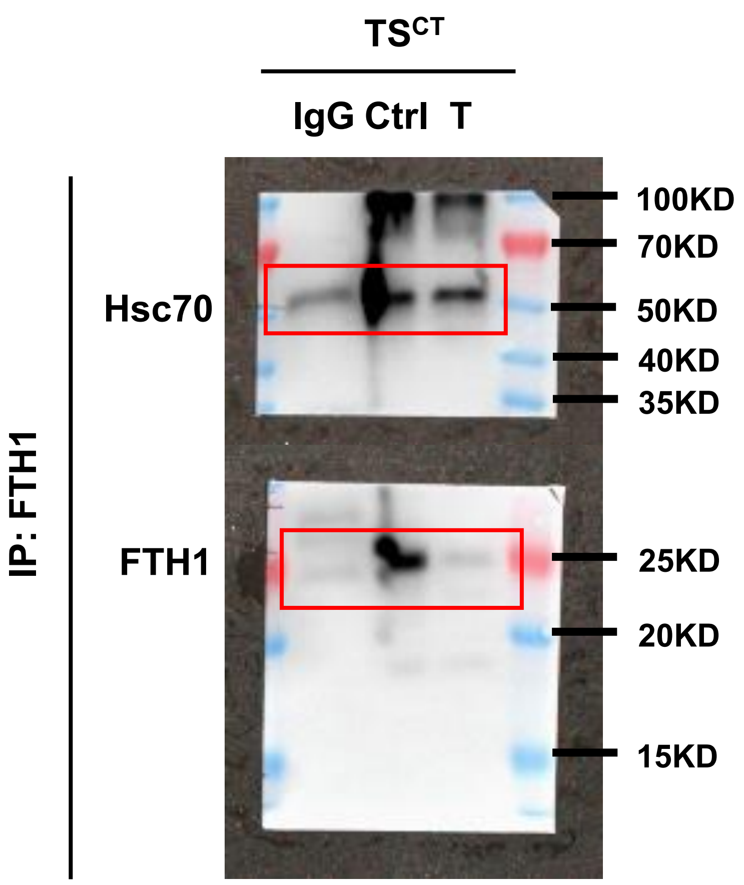


### The second repeat:

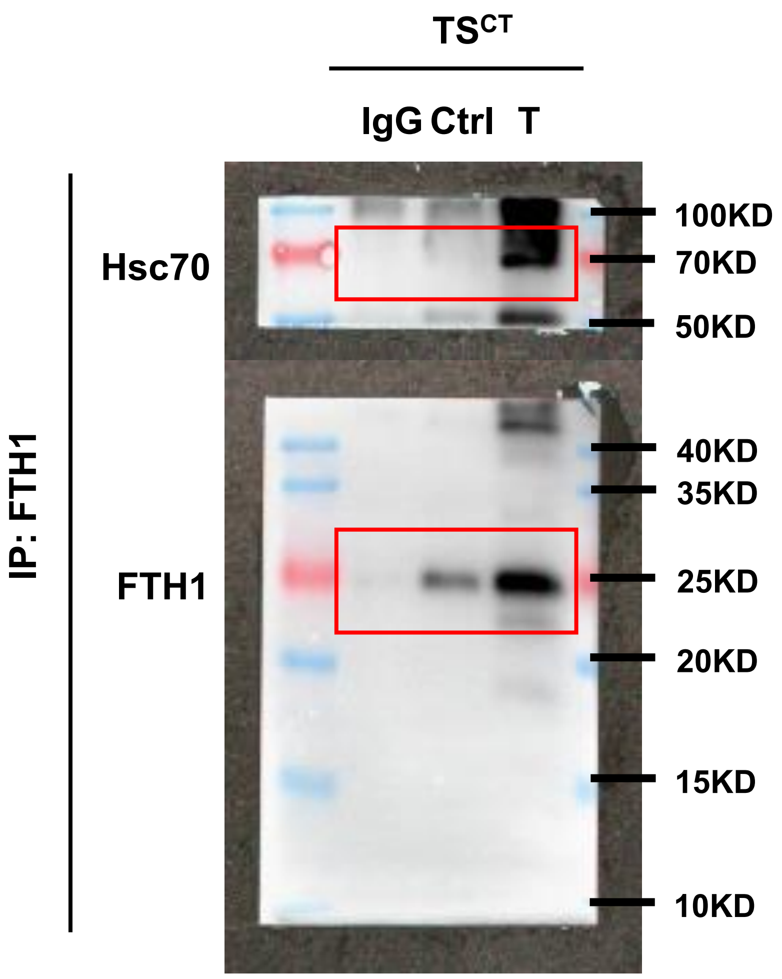


### The third repeat:

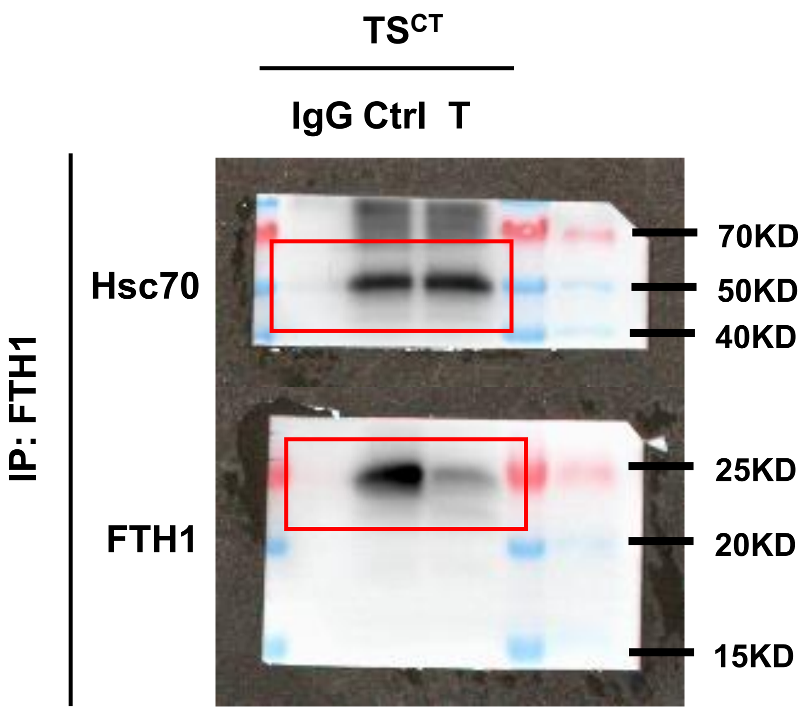


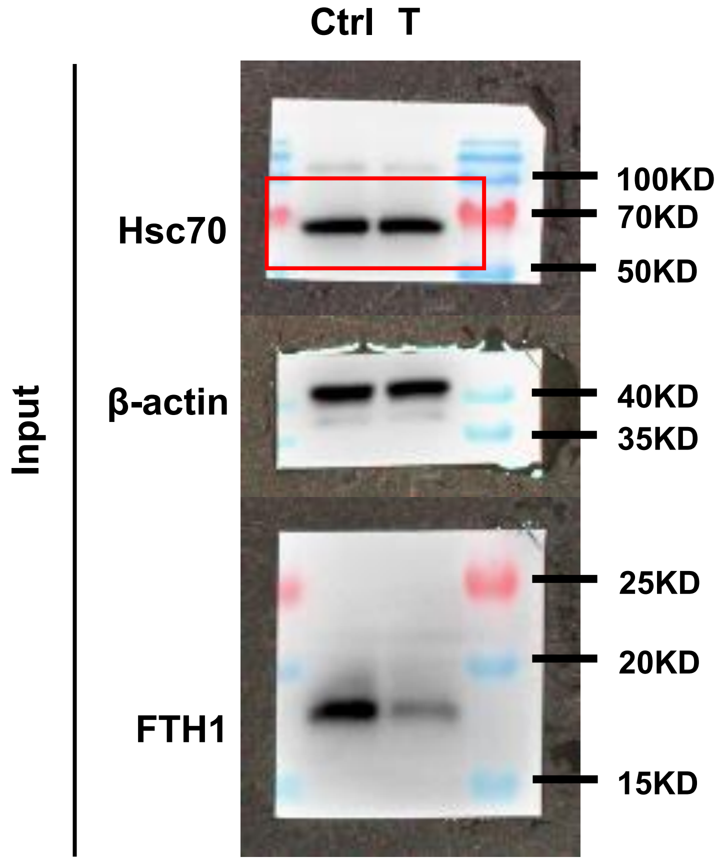


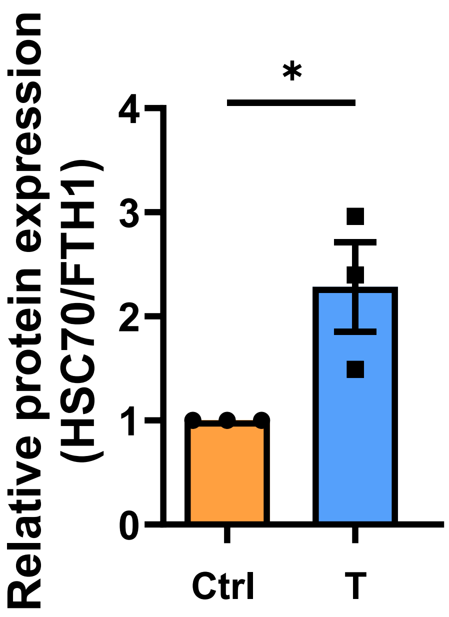

Figure S5E：

The first repeat:

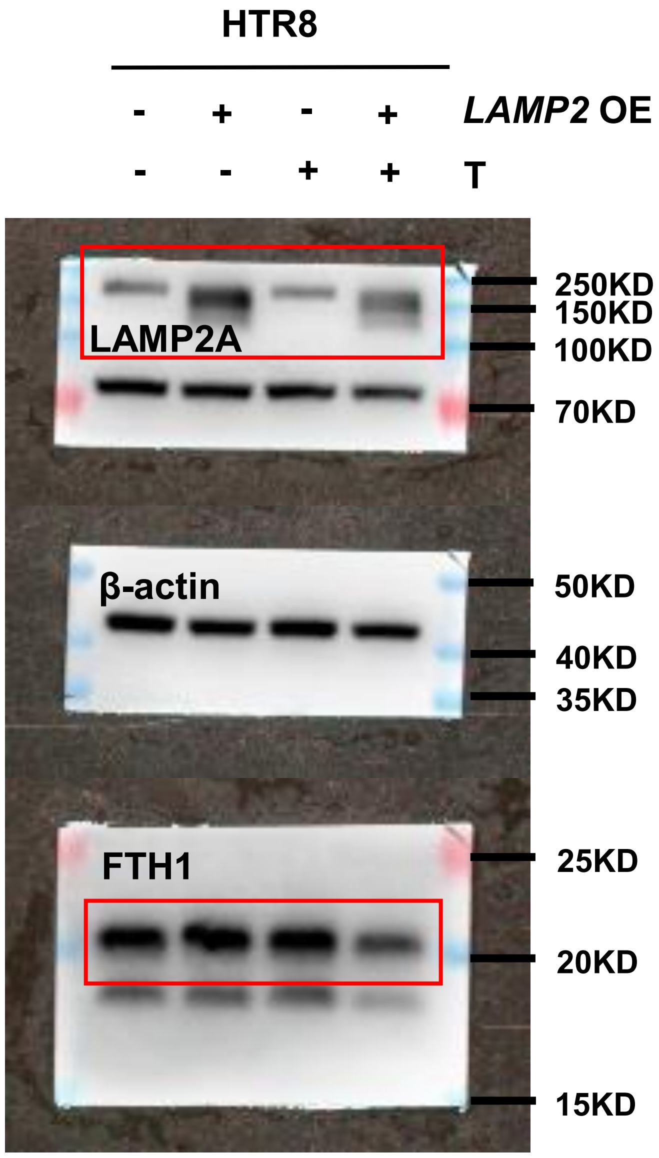


The second repeat:

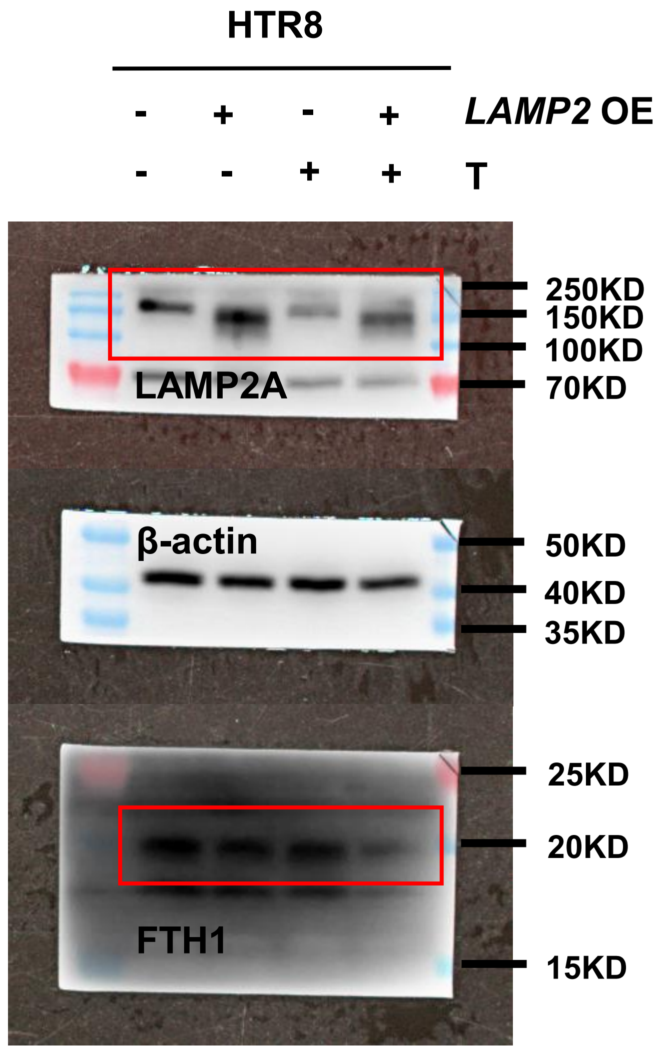


The third repeat:

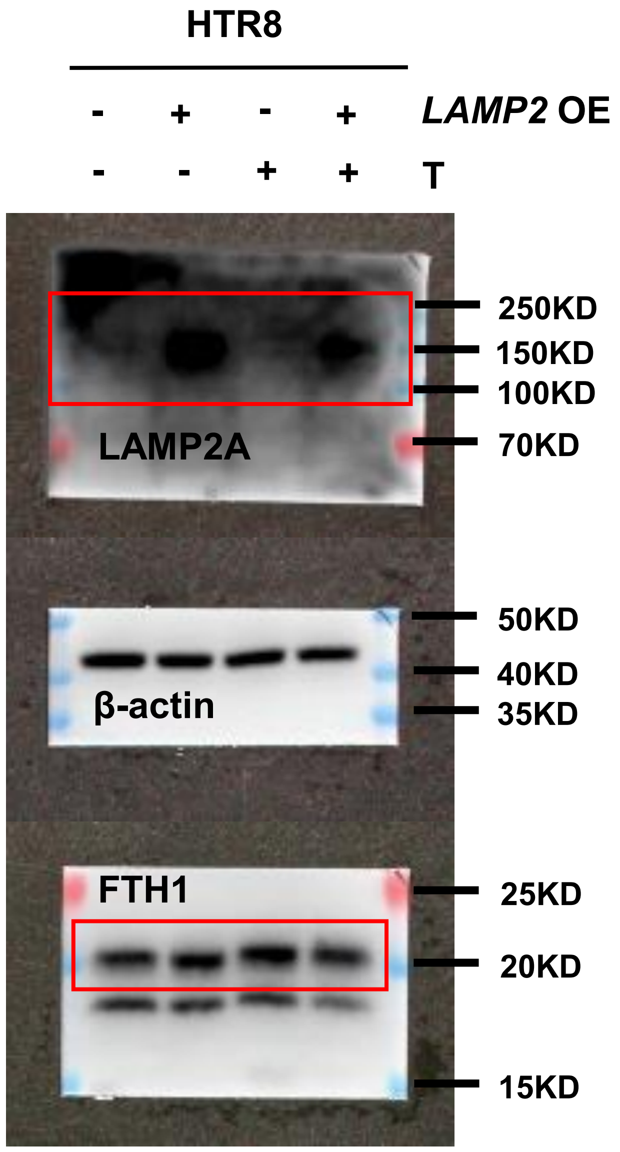


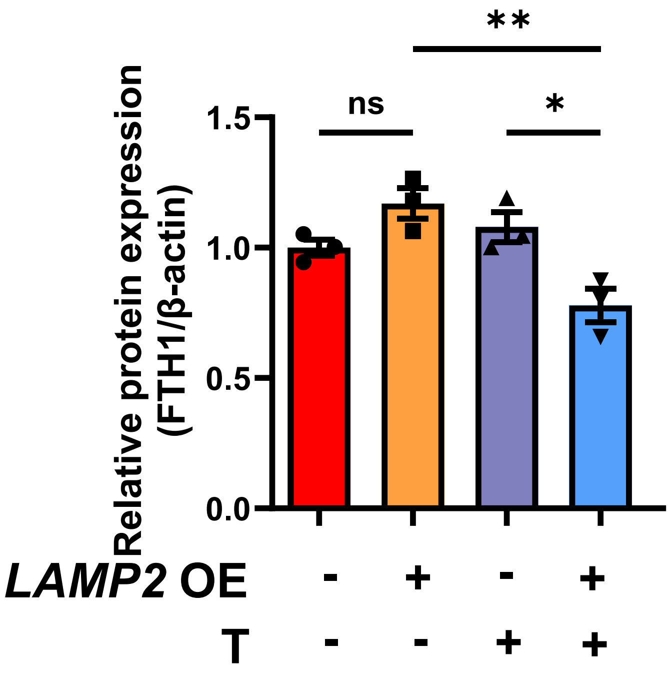

## Figure S6A：

### The first repeat:

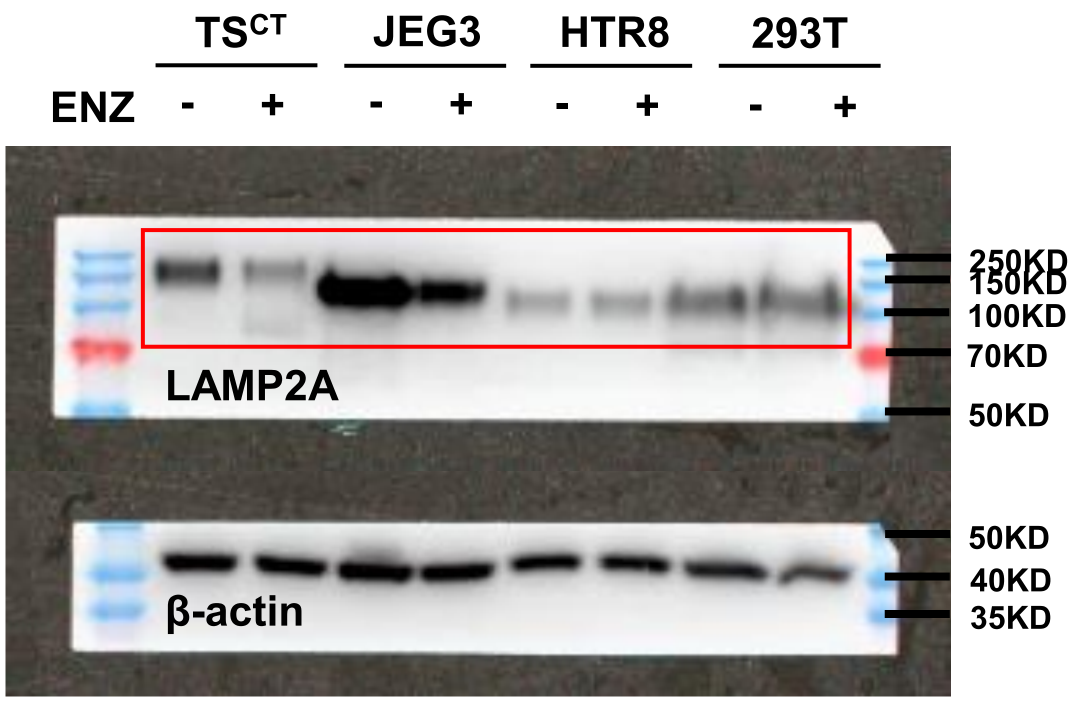


### The second repeat:

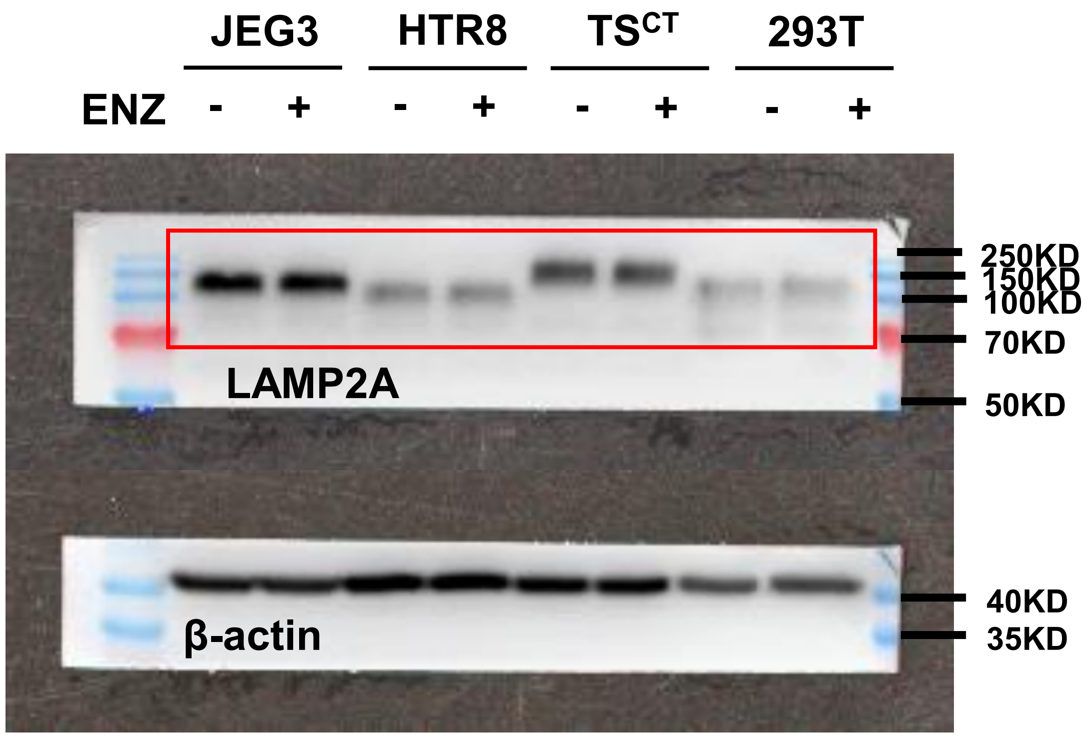


### The third repeat:

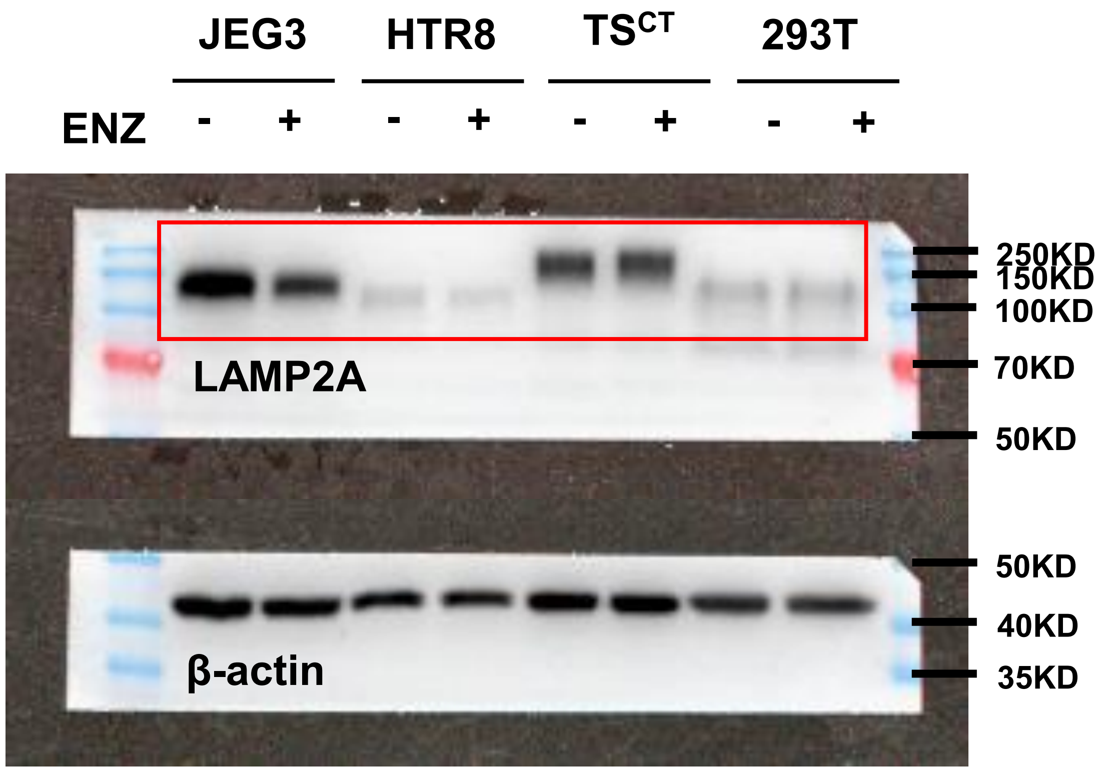


### The fourth repeat:

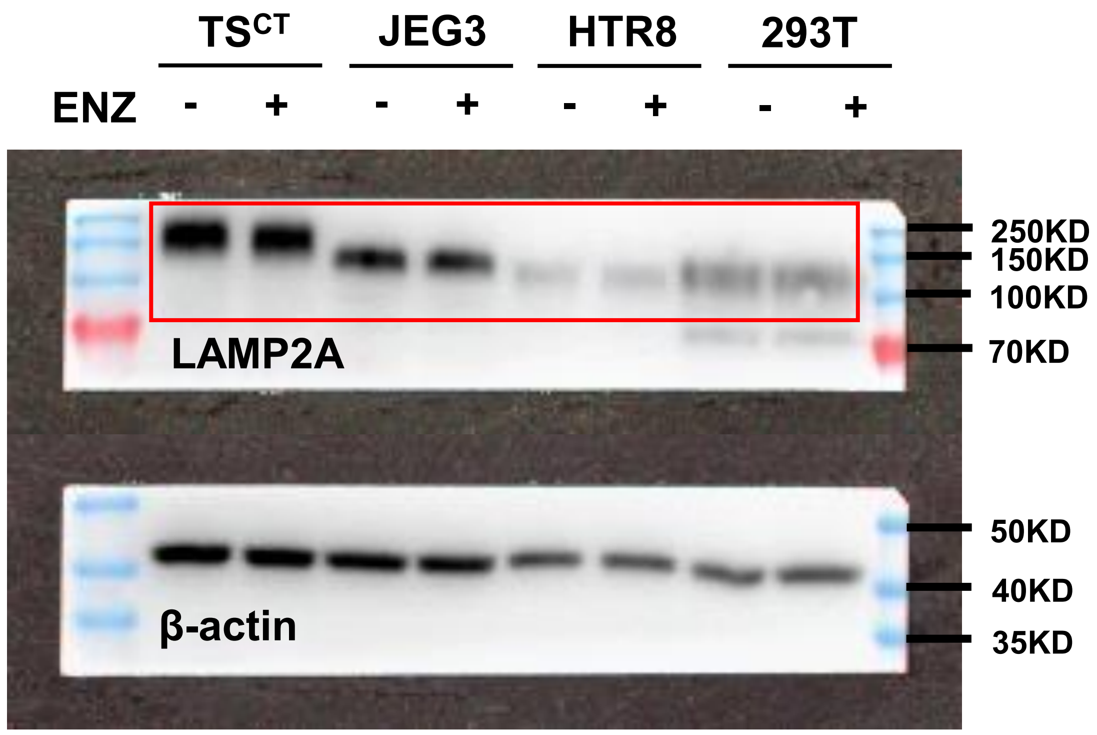


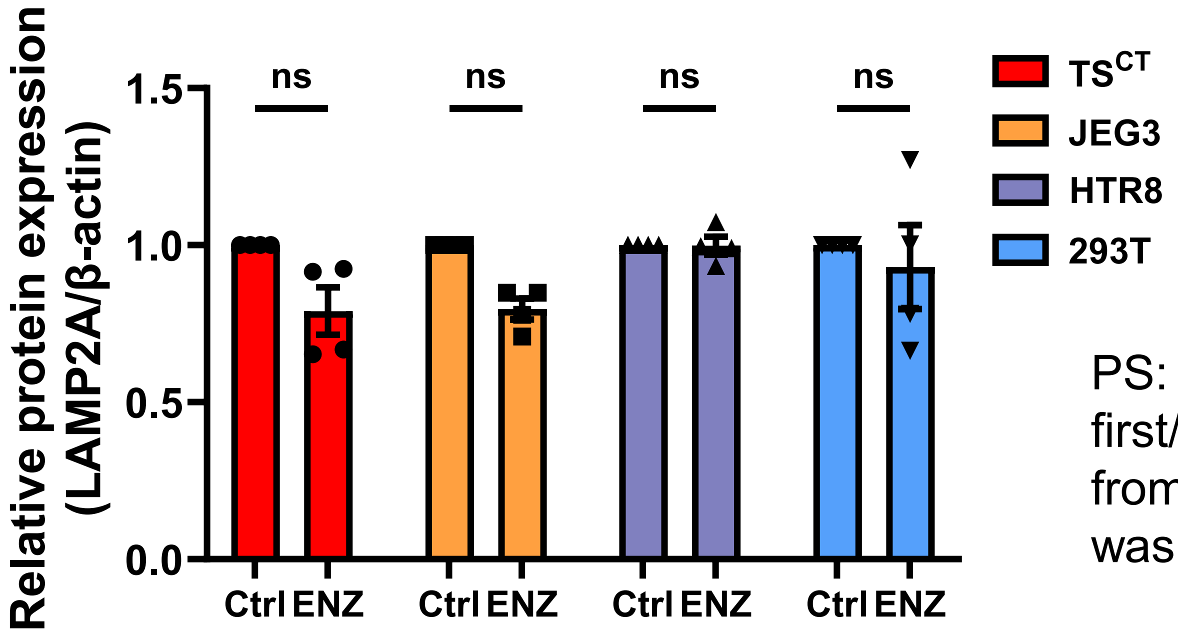


PS: The first/second repeat in Figure S6A and the first/second repeat in Figure S3A were derived from the same experiment, the WB membrane was washed with the stripping buffer.

## Figure S6C：

The first repeat:

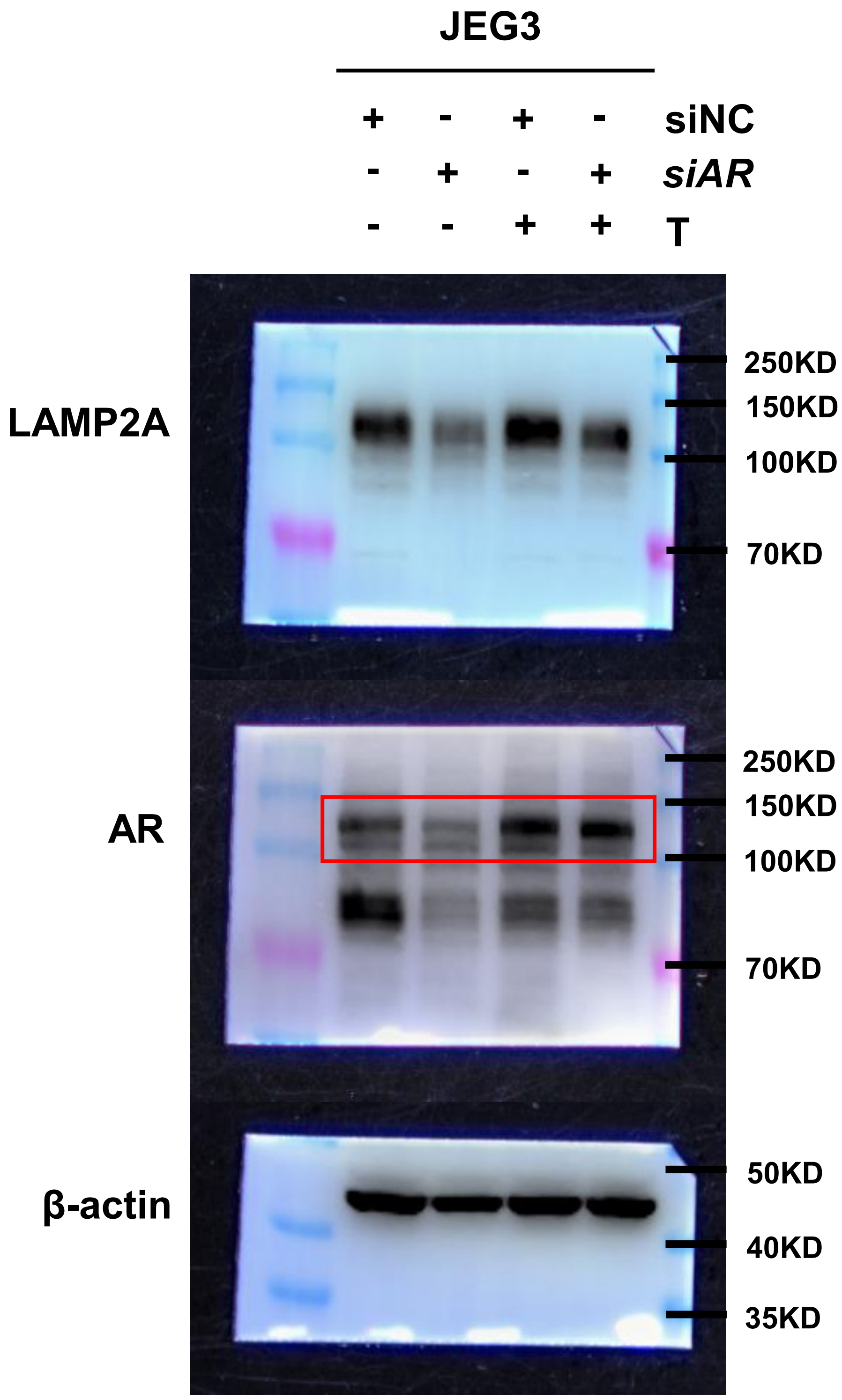


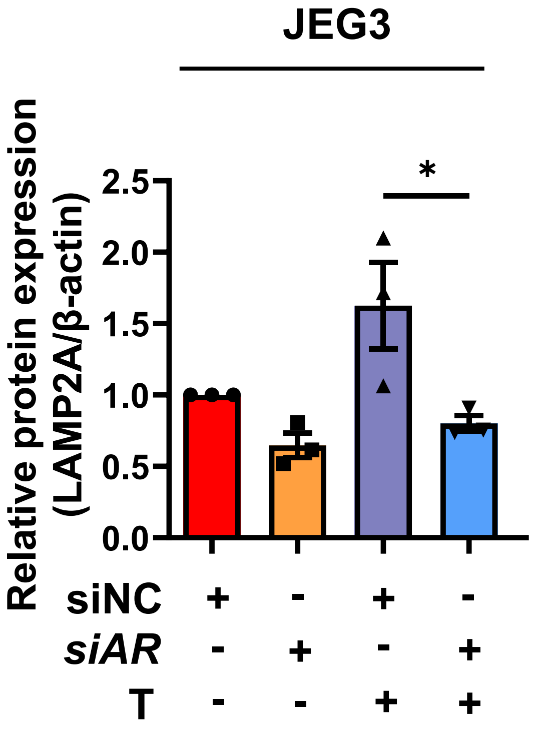


The second repeat:

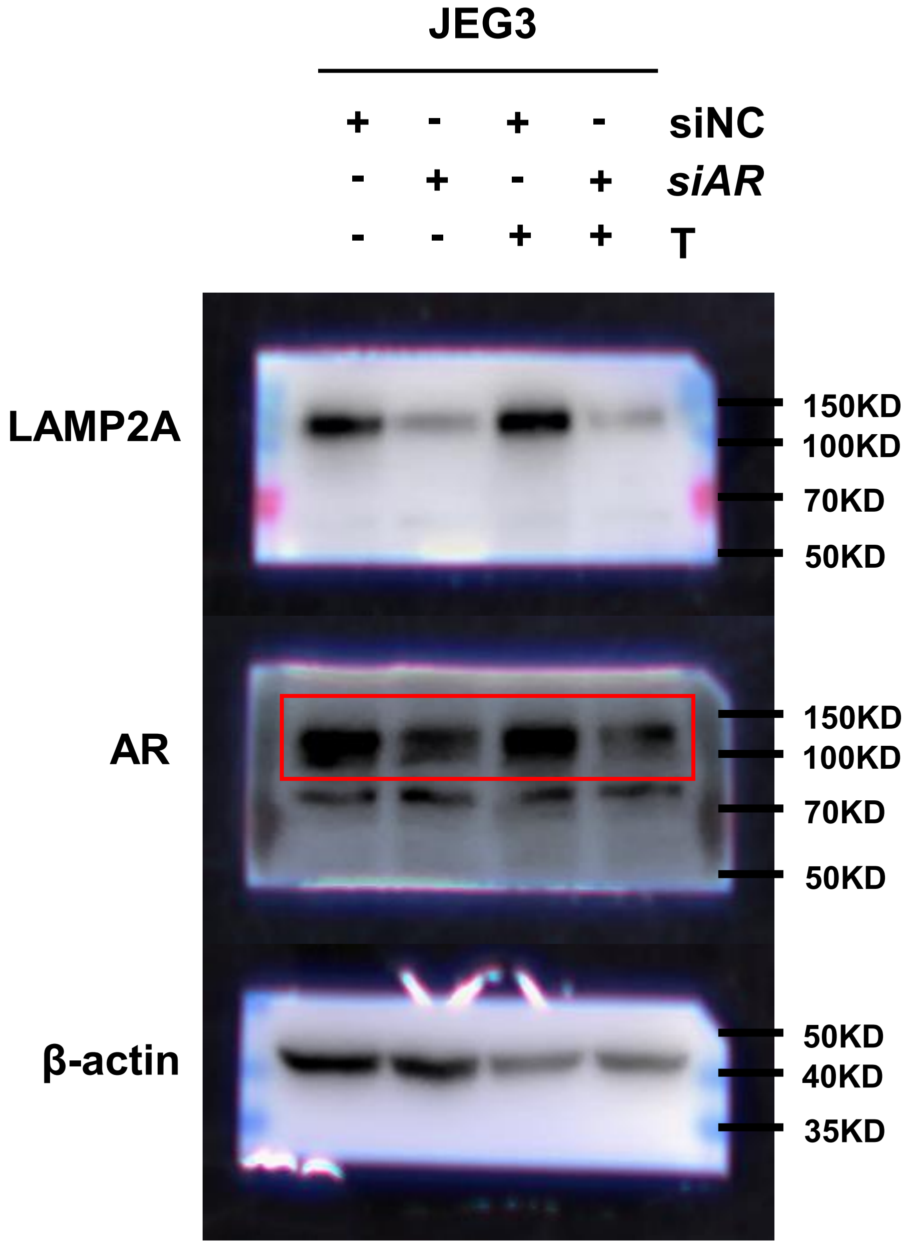


The third repeat:

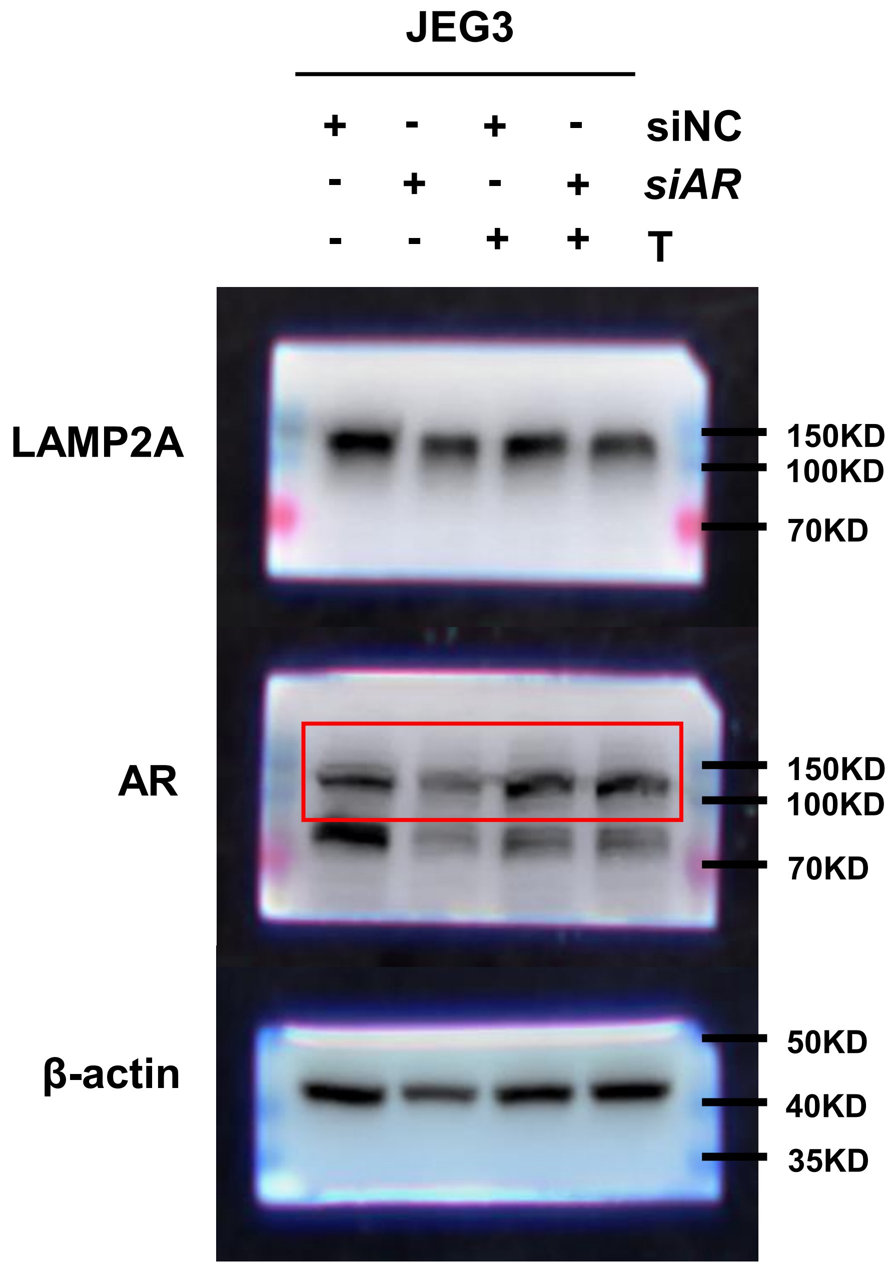

**Figure S6F:**

**The first repeat:**

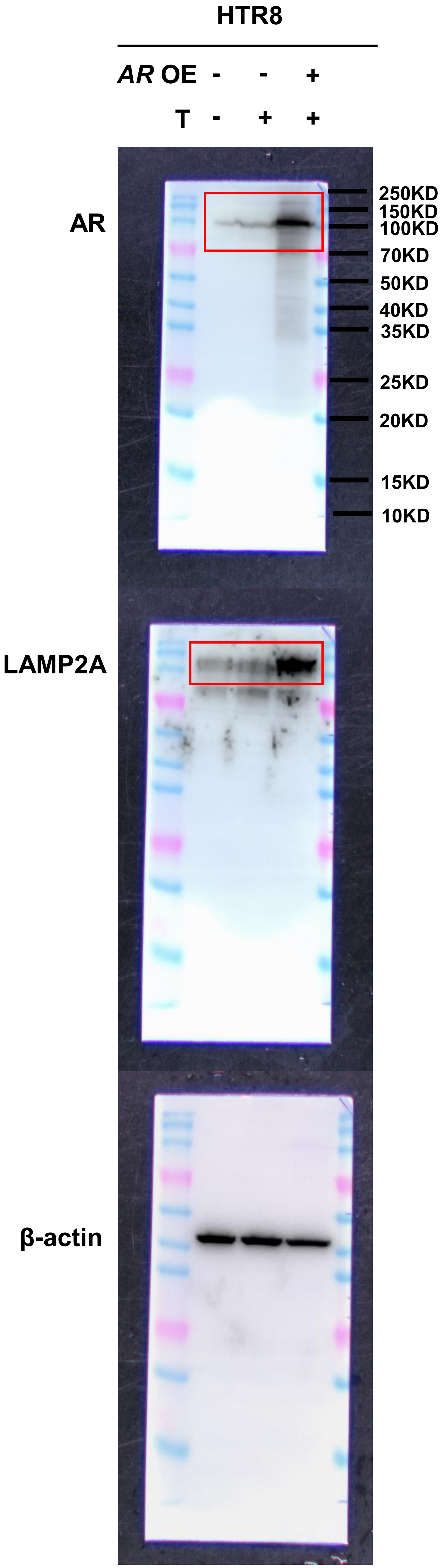


**The second repeat:**

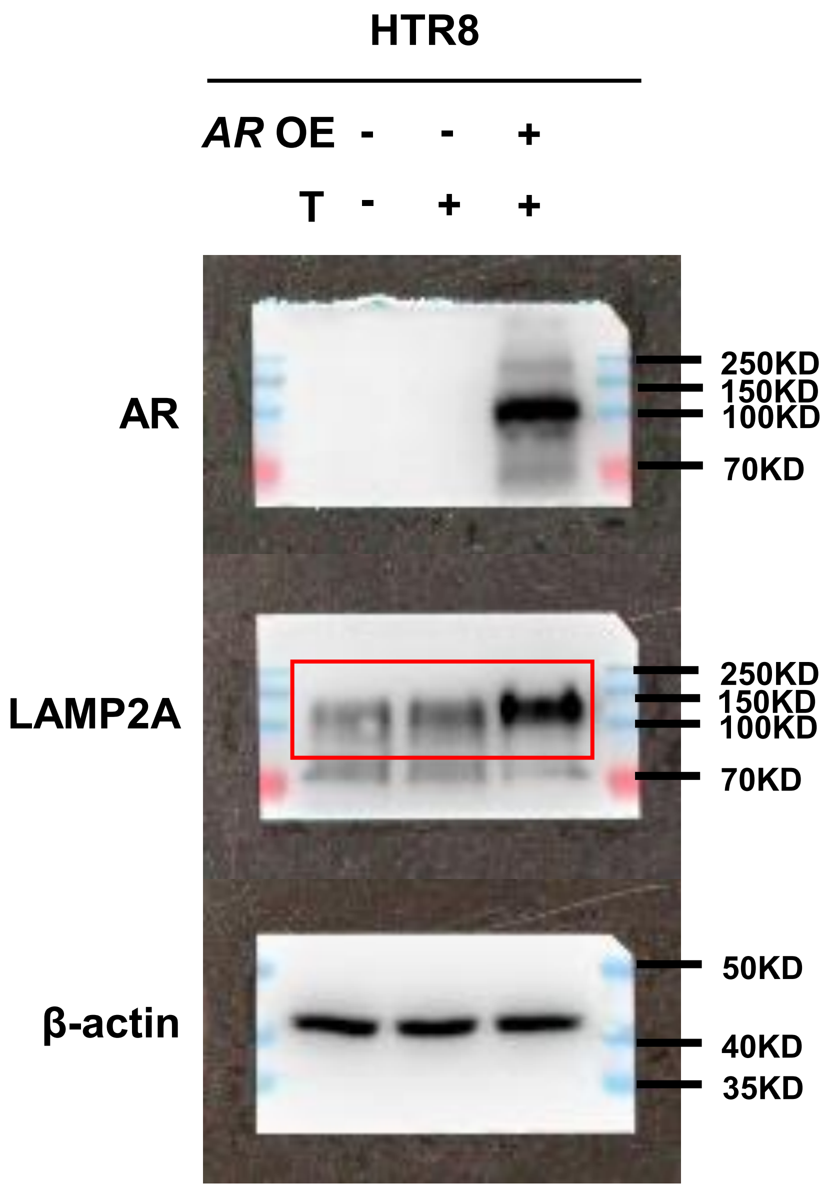


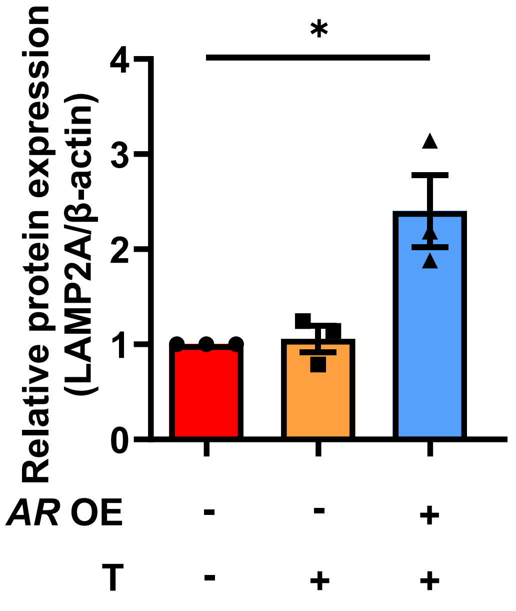


**The third repeat:**

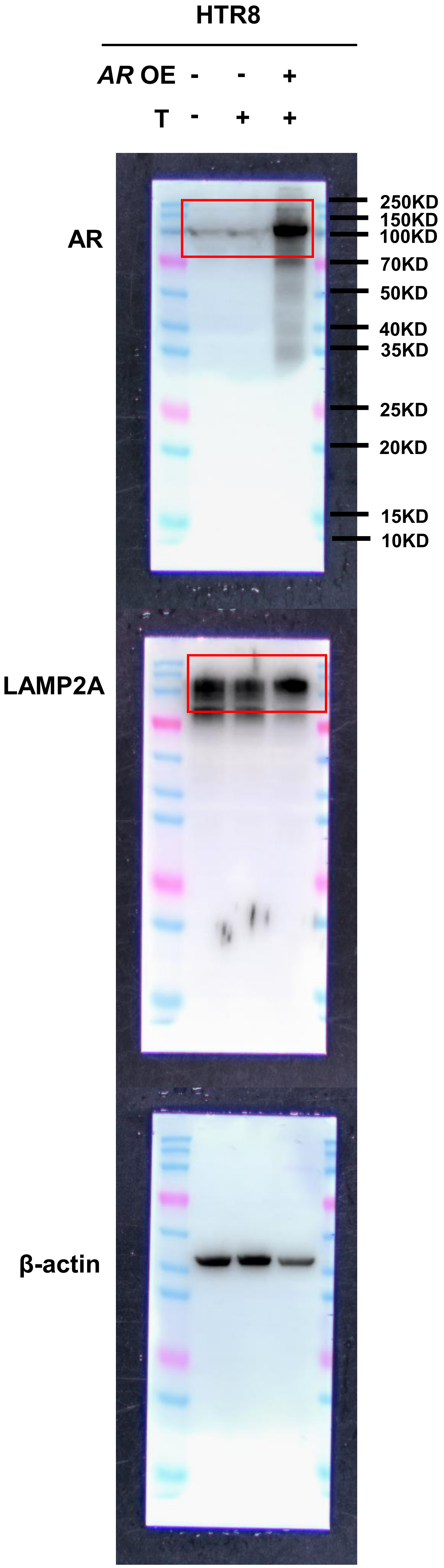

**Figure S9B：**

**The first repeat:**

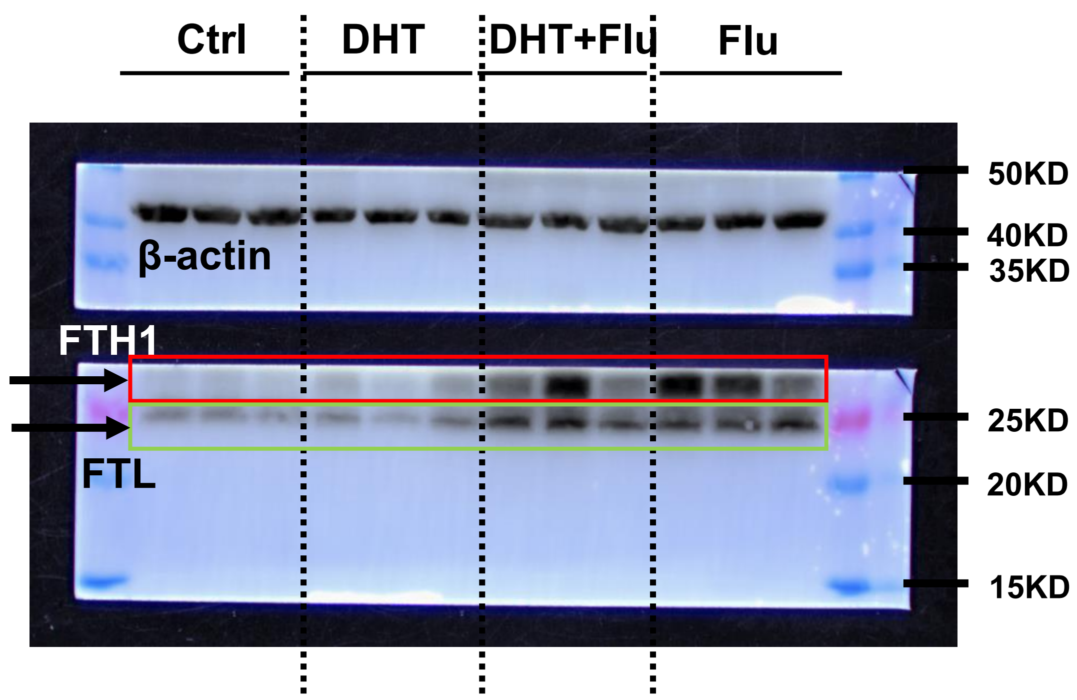


**The first repeat:**

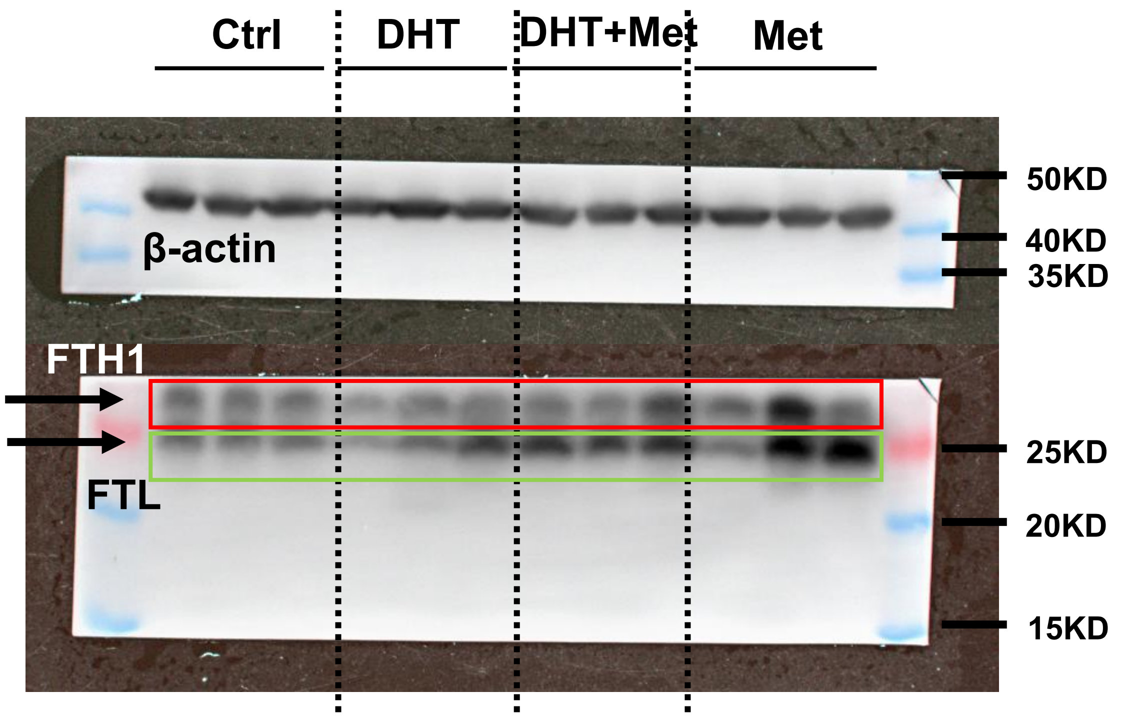


**The second repeat:**

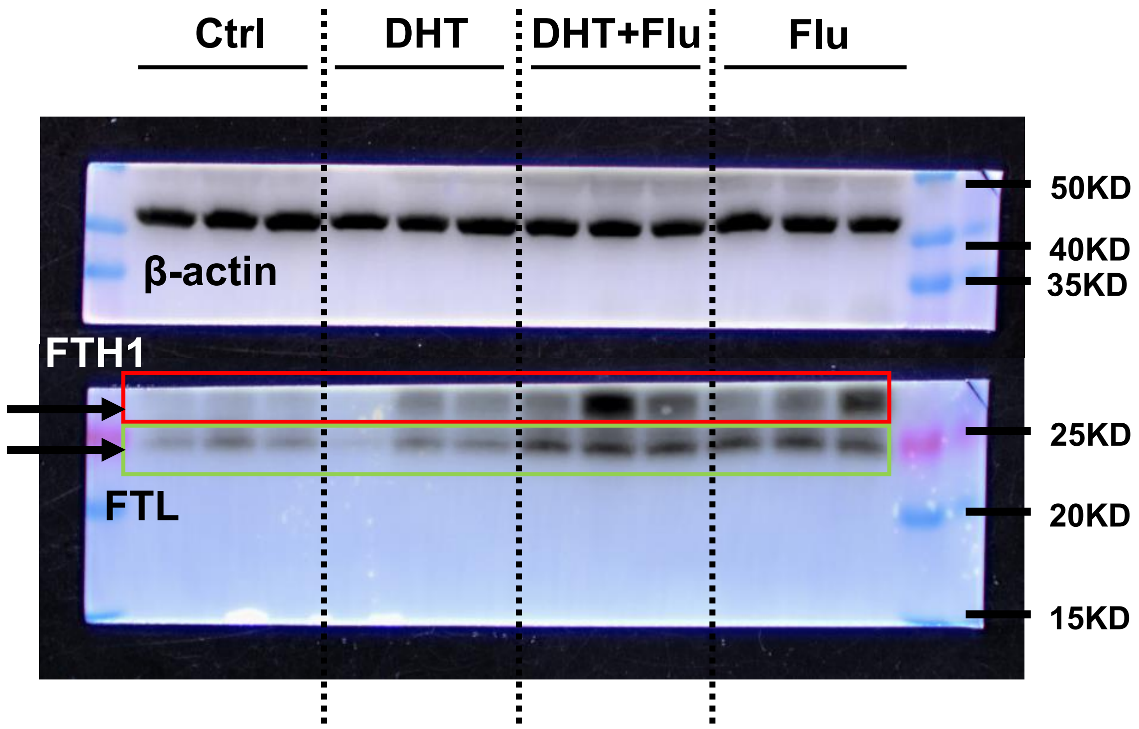


**The second repeat:**

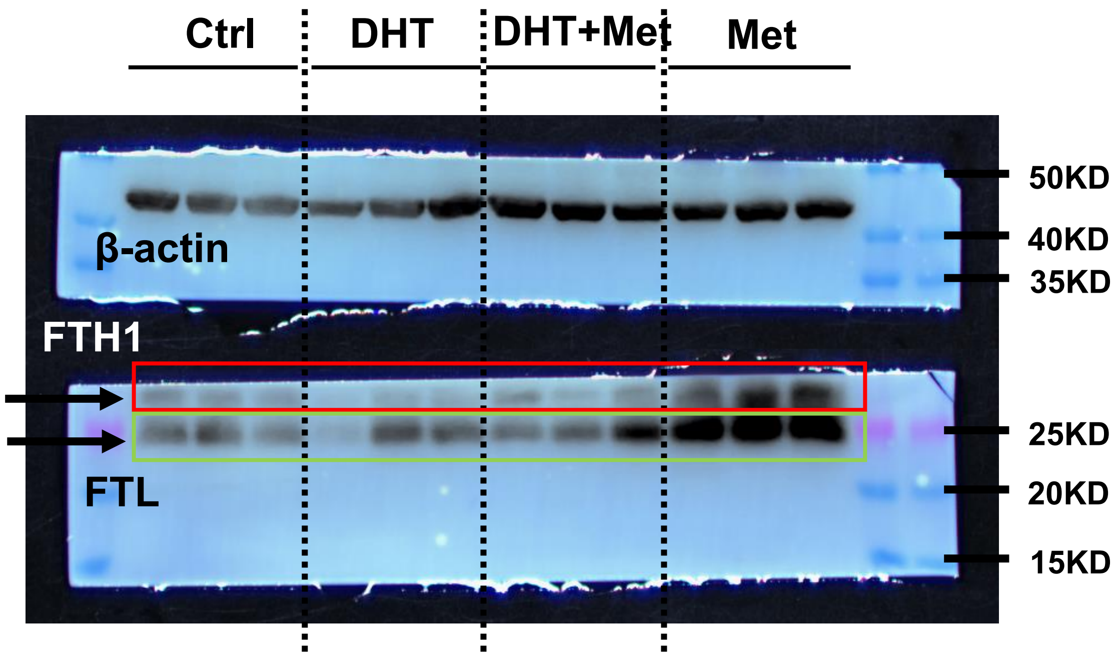


**The third repeat:**

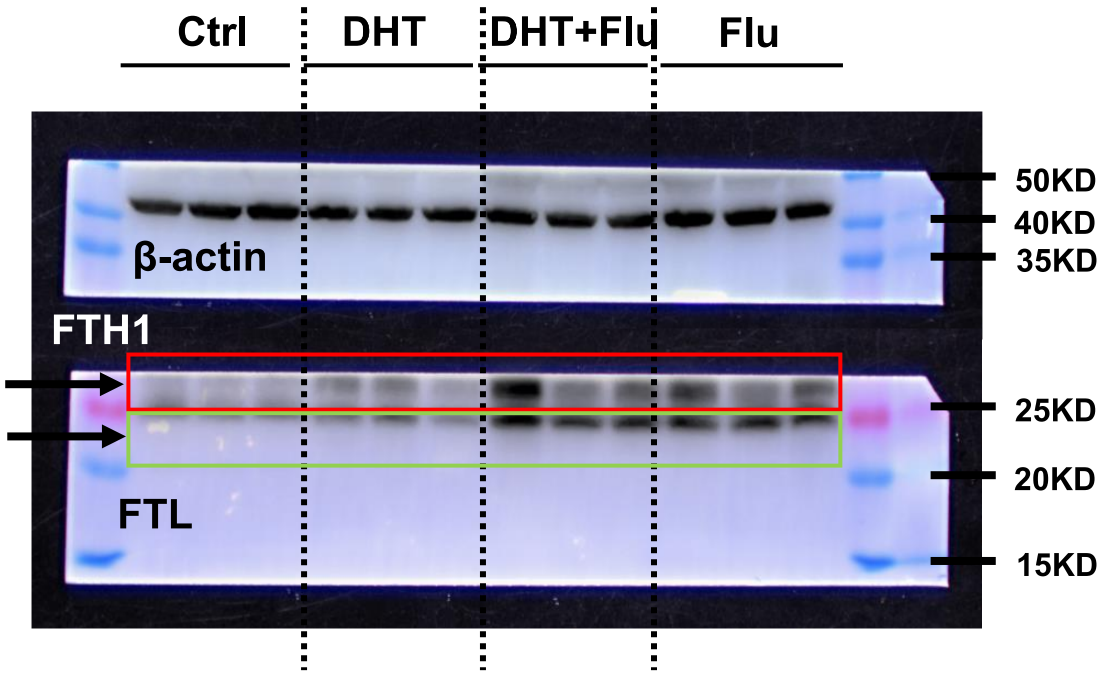


**The third repeat:**

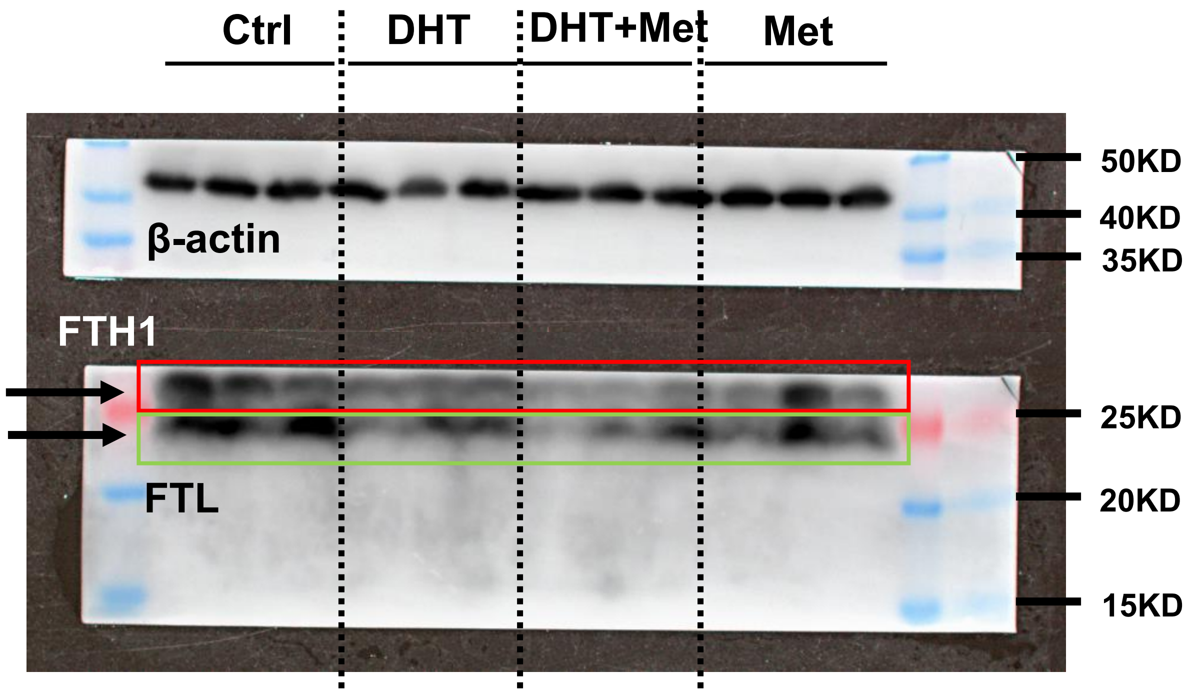

Supplement: Supplementary file 7 — Supporting Information [file ADVS-13-e06091-s004.pdf]
